# Immunoinformatic Design of a Multivalent Peptide Vaccine Against Mucormycosis: Targeting FTR1 Protein of Major Causative Fungi

**DOI:** 10.3389/fimmu.2022.863234

**Published:** 2022-05-26

**Authors:** Yusha Araf, Abu Tayab Moin, Vladimir I. Timofeev, Nairita Ahsan Faruqui, Syeda Afra Saiara, Nafisa Ahmed, Md. Sorwer Alam Parvez, Tanjim Ishraq Rahaman, Bishajit Sarkar, Md. Asad Ullah, Mohammad Jakir Hosen, Chunfu Zheng

**Affiliations:** ^1^Department of Genetic Engineering and Biotechnology, School of Life Sciences, Shahjalal University of Science and Technology, Sylhet, Bangladesh; ^2^Department of Immunology, School of Basic Medical Sciences, Fujian Medical University, Fuzhou, China; ^3^Department of Research and Development, Community of Biotechnology, Dhaka, Bangladesh; ^4^Department of Genetic Engineering and Biotechnology, Faculty of Biological Sciences, University of Chittagong, Chattogram, Bangladesh; ^5^Shubnikov Institute of Crystallography, Federal Scientific Research Centre, Crystallography and Photonics, Russian Academy of Sciences, Moscow, Russia; ^6^Biotechnology Program, Department of Mathematics and Natural Sciences, School of Data and Sciences, Brac University, Dhaka, Bangladesh; ^7^Department of Drug Discovery Medicine, Kyoto University Graduate School of Medicine, Kyoto, Japan; ^8^Department of Biotechnology and Genetic Engineering, Faculty of Life Sciences, Bangabandhu Sheikh Mujibur Rahman Science and Technology University, Gopalganj, Bangladesh; ^9^Department of Biotechnology and Genetic Engineering, Faculty of Biological Sciences, Jahangirnagar University, Dhaka, Bangladesh; ^10^Department of Microbiology, Immunology and Infectious Diseases, University of Calgary, Calgary, AB, Canada

**Keywords:** Mucormycosis, COVID-19, immunoinformatics, FTR1, SARS-CoV-2, vaccine

## Abstract

Mucormycosis is a potentially fatal illness that arises in immunocompromised people due to diabetic ketoacidosis, neutropenia, organ transplantation, and elevated serum levels of accessible iron. The sudden spread of mucormycosis in COVID-19 patients engendered massive concern worldwide. Comorbidities including diabetes, cancer, steroid-based medications, long-term ventilation, and increased ferritin serum concentration in COVID-19 patients trigger favorable fungi growth that in turn effectuate mucormycosis. The necessity of *FTR1* gene-encoded ferrous permease for host iron acquisition by fungi has been found in different studies recently. Thus, targeting the transit component could be a potential solution. Unfortunately, no appropriate antifungal vaccine has been constructed as of yet. To date, mucormycosis has been treated with antiviral therapy and surgical treatment only. Thus, in this study, the FTR1 protein has been targeted to design a convenient and novel epitope-based vaccine with the help of immunoinformatics against four different virulent fungal species. Furthermore, the vaccine was constructed using 8 CTL, 2 HTL, and 1 LBL epitopes that were found to be highly antigenic, non-allergenic, non-toxic, and fully conserved among the fungi under consideration. The vaccine has very reassuring stability due to its high pI value of 9.97, conclusive of a basic range. The vaccine was then subjected to molecular docking, molecular dynamics, and immune simulation studies to confirm the biological environment’s safety, efficacy, and stability. The vaccine constructs were found to be safe in addition to being effective. Finally, we used *in-silico* cloning to develop an effective strategy for vaccine mass production. The designed vaccine will be a potential therapeutic not only to control mucormycosis in COVID-19 patients but also be effective in general mucormycosis events. However, further *in vitro*, and *in vivo* testing is needed to confirm the vaccine’s safety and efficacy in controlling fungal infections. If successful, this vaccine could provide a low-cost and effective method of preventing the spread of mucormycosis worldwide.

## Introduction

Mucormycosis is a life-threatening but opportunistic fungal infection caused by a group of molds known as mucormycetes. Belonging to the class of mucormycetes (formerly known as Zygomycetes) of the Mucoraceae family, and from the order of Mucorales, their distinctive attribute to cause infections in humans are often only stimulated by a weakened immune system, making them opportunistic agents. *Rhizopus delemar* (*Rhizopus oryzae* strain 99-880), *Rhizopus oryzae*, *Rhizopus azygosporus*, *Rhizopus stolonifer*, and *Mucor circinelloides* have been reported to be the causative agents of most infections (1–4). Moreover, accounting for 70% of all cases, *Rhizopus delemar* is the most prevalent species isolated from patients with mucormycosis (5).

While countries across the globe suffer from the coronavirus disease 2019 (COVID-19), India’s state exacerbates as the simultaneous epidemic of “Black fungus (BF)”, colloquial term for mucormycosis, strikes the country majorly, and with several cases reported in some other regions as well (6, 7). However, while these countries showed only limited cases and few to no deaths from 5 May – 3 August 2021, India reported a total of 47,508 cases and 4,425 deaths attributable to their country-wide black fungus epidemic: with an overall 41,512 cases and 3,554 deaths worldwide in the same timeframe (8, 9).

**Figure 1:**
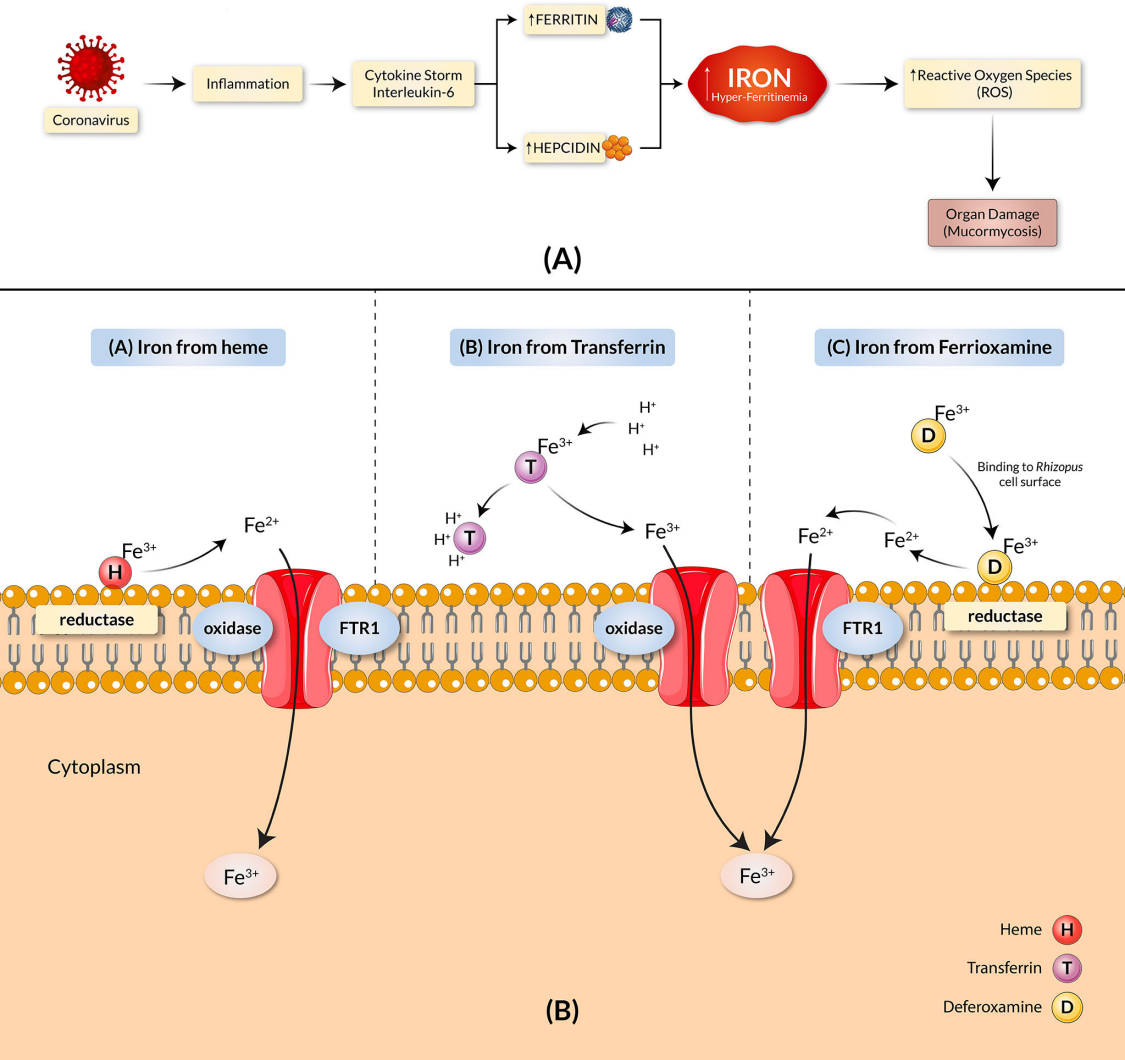
COVID-19 and mucormycosis correlated through the iron uptake mechanism. **(A)** Iron dysregulation and COVID-19. SARS-CoV-2 causes heightened inflammation and a pro-inflammatory cytokine storm, elevating hepcidin synthesis. The increase in hepcidin reduces the available Ferroportin and leads to intracellular iron overload, causing an increase in ferritin concentration. The result is hyper-ferritinemia. **(B)** Proposed pathogenic mechanisms of mucormycosis through iron uptake include the stripping of ferric ions (Fe3+) from heme groups (H), proton-mediated displacement of ferric ions from transferrin (T), and chelation of ferric irons by deferoxamine (D). The copper oxidase–iron permease (FTR1) complex transports iron across the cell membrane in all cases.

Mounting evidence exhibits implications of iron overload in the pathogenesis of COVID-19 through an increased ferritin serum concentration in COVID-19 patients (10–12), shown in a detailed manner in **Figure 1A**. Clinical and animal data demonstrated that the presence of elevated available serum iron predisposes the host to mucormycosis (13). The increased free unbound iron in COVID-19 patients causes oxidative damage to organs, further increasing their susceptibility to BF with an aided growth. Despite rigorous treatment efforts, including surgical debridement and antifungal therapy, the overall mortality rate of mucormycosis remains high (13). While iron remains a crucial element for cell growth, development, and the virulence of fungi, growing concern ensues as both the host iron availability and deferoxamine, termed as the iron chelator, are used to alleviate serum iron levels in COVID-19 patients which further predispose individuals to mucormycosis (14, 15). There are three main mechanisms by which fungi obtain iron from their host (**Figure 1B**). The first approach includes extracting the ferric iron through confiscation from the heme group by the fungi. The second approach involves the procurement of the ferric iron by the fungi through proton-mediated displacement methods from transferrin in internal acidic conditions, as seen in diabetic ketoacidosis (DKA). Thirdly, the iron chelator’s deferoxamine acts as a xenosiderophore and strips ferric iron from transferrin to form a complex with the iron, known as ferrioxamine (iron-deferoxamine complex). Following this, through the utilization of reductases on the surface of the fungi, the extracted ferric ions are reduced to soluble ferrous ions in all cases (9). The ferrous ion is then absorbed into the cytoplasm by the high-affinity transport complex containing an oxidase and a permease (FTR1) (16). All these mechanisms contribute to the uptake of iron and the pathogenesis of mucormycosis, which involve the transportation of iron across the cell membrane by the copper oxidase-iron permease (FTR1) complex. The proposed mechanistic pathways adapted by the fungi for iron acquisition confer potentially favorable conditions for mucormycosis. Host iron acquisition by fungi is mediated by utilizing high-affinity iron permeases or low-molecular-weight iron chelators (siderophores) (17). These high-affinity iron permeases are part of the fungi reductive system which contains redundant surface reductases to reduce ferric iron into the more soluble ferrous form. This solubilized ferrous iron is further collected by a protein complex consisting of a multicopper oxidase and a ferrous permease (18–20). The ferrous permease, part of the transit complex of many fungi (including R. oryzae), encoded by the FTR1 gene, was found required for iron transport in iron-dependent environments (13). In addition, disruption of the FTR1 gene revealed reduced virulence of fungi in a mouse model (13). A study by Ibrahim et al. reported the FTR1 gene as a potential key virulence factor for fungal pathogenesis (13). Therefore, targeting the transit component for iron acquisition may play a potential role in improved mucormycosis outcomes. Thus, in this study, we designed and constructed an effective and suitable polyvalent vaccine against the different virulent Rhizopus and Mucor fungus of mucormycosis, targeting the Ftr1 protein through the exploitation of immunoinformatic approaches.

## Methodology

**Figure 2:**
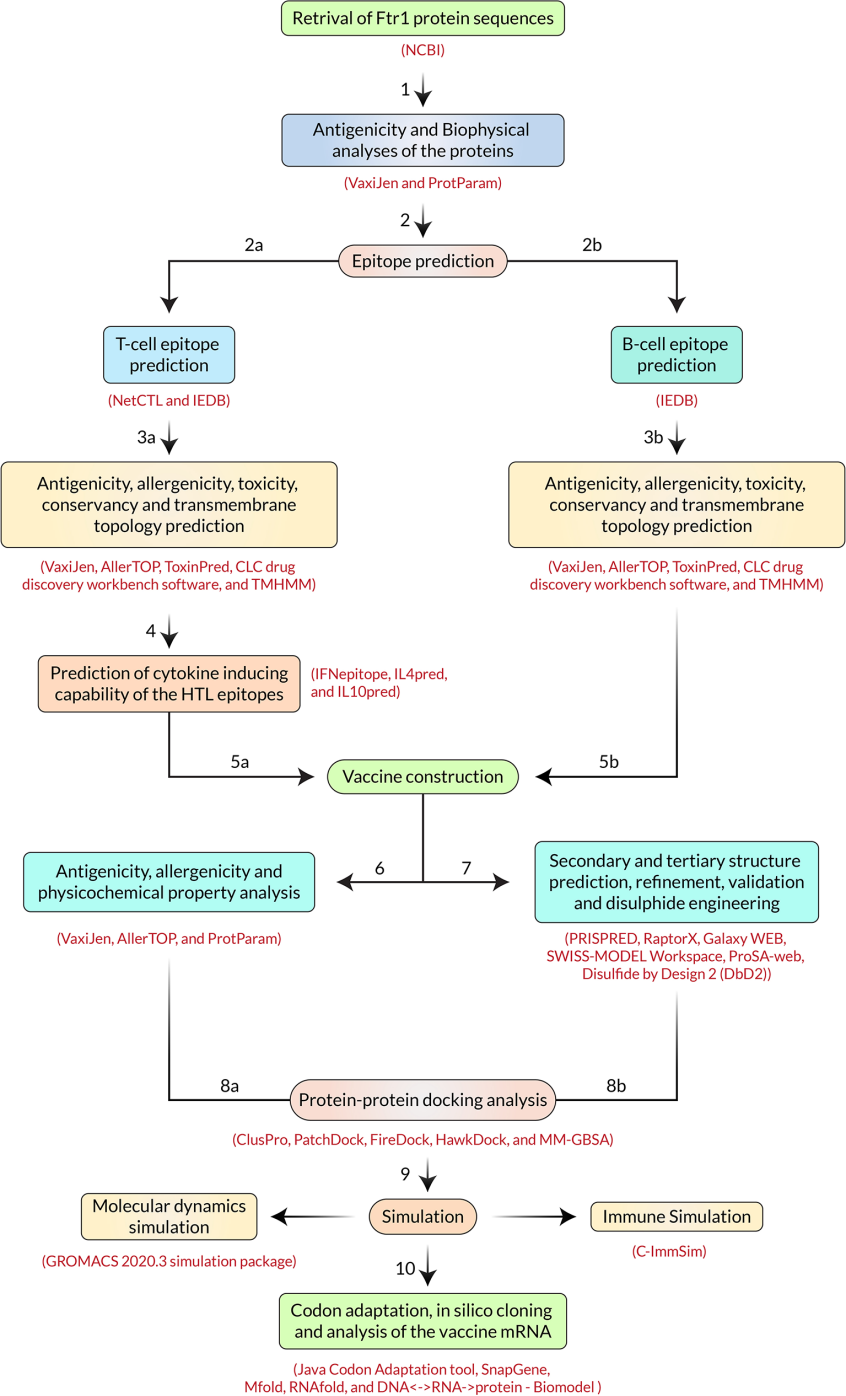
Step-by-step procedures used in the vaccine constructing experiment.

The extensive throughput vaccine designing immunoinformatic approaches utilized - including the FTR1 protein, are illustrated in **Figure 2**.

### Identification of Species and Retrieval of High-Affinity Iron Permease Protein Sequences

Sequences of FTR1 protein from four different fungi were retrieved in FASTA format from the NCBI (https://www.ncbi.nlm.nih.gov/) database.

### Epitope Mapping

MHC class-I or CD8+ cytotoxic T-lymphocytic (CTL) and MHC class-II or helper T-lymphocytic (HTL) epitopes were targeted to get an effective vaccine. The most antigenic CTL epitopes were screened using the NetCTL server (http://www.cbs.dtu.dk/services/NetCTL/) [33]. CTL alleles were grouped into 12 superfamilies (A1, A2, A3, A24, A26, B7, B8, B27, B39, B44, B58, B62). Targeted FTR1 protein was screened against each of the CTL superfamily members [kept threshold values as HLAI binding (epitope identification) >0.75, weight on proteasomal C-terminal cleavage = 0.15, and weight on TAP (transport efficiency) = 0.05]. Higher predicted scores from the NetCTL server (>0.75) correspond to higher specificity of the epitopes (21). Next, HTL epitope binding was predicted using the IEDB recommended 2.22 prediction method of the IEDB prediction server (22) with a low percentile rank (<1.00) as the selection criteria. In this case, a lower percentile rank signifies a higher affinity of the epitopes. Finally, the human allele reference sets were utilized to predict HTL binding (23, 24). The B-cell epitope structure can be classified into two types based on the spatial structure of the epitopes: continuous (linear) and discontinuous (conformational) epitopes (25). The BepiPred linear epitope prediction method 2.0 of the IEDB prediction server (http://tools.iedb.org/bcell/) was used to identify the linear B-cell lymphocyte epitopes (LBL), keeping all parameters set to default (22). The discontinuous (conformational) epitopes, on the other hand, were predicted using the ElliPro prediction tool (http://tools.iedb.org/ellipro/) of the IEDB server (22). This method uses solvent accessibility and flexibility to predict epitopes. The ElliPro prediction tool selected antigenic residues from the predicted three-dimensional (3D) structure. The lowest score and maximum distance (Angstrom) were calibrated using the default mode with 0.5 and 6, respectively (26). As the 3D structure of FTR1 was not available, its model was generated using the RaptorX web server (http://raptorx.uchicago.edu/). The server uses an excellent and efficient template-based technique to predict query proteins’ tertiary or 3D structure (27). The GalaxyRefine server (http://galaxy.seoklab.org/cgi-bin/submit.cgi?type=REFINE) was then used to refine the modeled tertiary structure. The tool uses dynamics modeling and a CASP10-tested refinement method to produce better-refined structures (28, 29). This refined 3D model of the FTR1 protein was eventually used to predict the conformational (discontinuous) B-cell epitope. In addition, the IEDB server’s Emini surface accessibility prediction tool was utilized to predict surface epitopes from the conserved region using the 1.0 default threshold value (30).

### Conservancy Prediction of the Epitopes

A conservation study of the predicted epitopes was performed using the selected fungal species with an accessible annotated sequence from NCBI. More comprehensive coverage and protection against all mentioned virulent rhizopus and mucor species were achieved by selecting epitopes in the conserved regions of the *R. oryzae* sequence. CLC drug discovery-workbench software version 3.0 (https://digitalinsights.qiagen.com/) was used to perform multiple sequence alignments of the selected proteins to identify epitopes that are conserved across all the targeted species. The conservancy test guarantees that the polyvalent vaccine has broad-spectrum activity against the targeted species or types of fungus.

### Analyses of Antigenicity, Allergenicity, Toxicity, and Cytokine Inducing Ability of the Epitopes

Epitopes that were highly antigenic, non-allergenic, non-toxic, 100% conserved among the desired species, and had the proper cytokine-inducing ability (for HTL epitopes) were considered the most promising epitopes for the vaccine development process. The antigenicity and allergenicity of the selected epitopes were predicted using the VaxiJen v2.0 (http://www.ddg-pharmfac.net/vaxijen/VaxiJen/VaxiJen.html) server (31) and the AllerTOP v2.0 (https://www.ddg-pharmfac.net/AllerTOP/) server, respectively (32). Afterward, the toxicity prediction of the epitopes was analyzed using the support vector machine (SVM) prediction method of the ToxinPred server (http://crdd.osdd.net/raghava/toxinpred/), with all parameters kept at default (33). The SVM method has great accuracy in distinguishing toxic and non-toxic epitopes. Finally, the TMHMM v2.0 server (http://www.cbs.dtu.dk/services/TMHMM/) was used to predict the transmembrane topology of all epitopes, with the parameters set to their default settings (34). The cytokine-inducing ability of the HTL epitopes is important as induced cytokines such as interferon-gamma (IFN-gamma), interleukin-4 (IL-4), and interleukin10 (IL-10) stimulate diverse immune cells, i.e., cytotoxic T-cells, macrophages, B-cells, and generate substantial immune responses (35). The IFN-gamma induction capability of the predicted HTL epitopes was determined using the Design Module and the Hybrid (Motif + SVM) prediction approach of the IFNepitope (http://crdd.osdd.net/raghava/ifnepitope/) server. Here, the Hybrid prediction approach is one of the highly accurate approaches for predicting the IFN-gamma inducing capability of the epitopes (36). Furthermore, the ability of the HTL epitopes to induce IL-4 and IL-10 was assessed using the IL4pred (https://webs.iiitd.edu.in/raghava/il4pred/index.php) and IL10pred (http://crdd.osdd.net/raghava/IL-10pred/) servers, respectively (37, 38). The SVM algorithm was utilized in both servers, with default threshold values of 0.2 and -0.3, respectively.

### Peptide Fusion for Construction of a Vaccine

Linker peptides were used to facilitate the conjugation of the most promising epitopes to create a fusion protein. The AAY, GPGPG, and KK linkers conjugate the CTL, HTL, and BCL epitopes. The EAAAK linkers allow partitioning domains in bi-functional fusion proteins, while the GPGPG linkers can avoid junctional epitope formation and improve immune processing and presentation (39, 40). The AAY linker is also commonly employed *in silico* vaccine designing due to its known ability to form effective epitope conjugation (41). In addition, the bi-lysine (KK) linkers are involved in independent immunological activities of the epitopes used in vaccines (42). Additionally, a Toll-like receptor 4 (TLR4) agonist RS09 (APPHALS) was incorporated at the N-terminus of the final vaccine design. The adjuvant and linker peptides were chosen based on the study previously conducted by Pandey et al. (43). RS09 is a synthetic version of lipopolysaccharide (LPS), which acts as a natural TLR4 ligand (44). Therefore, the presence of RS09 allows for TCR co-stimulation, resulting in a more robust immune activation. Synthetic adjuvants, such as RS09 and Freund’s adjuvant, are a safer alternative and are considered an upgrade over traditional vaccination methods (45). Further, the human beta-defensin-3 was also used as an adjuvant sequence and joined the EAAAK linker sequences. The adjuvant and epitopes were also conjugated with the pan HLA-DR epitope (PADRE) sequence. The PADRE sequence boosts the immunological response by increasing the capacity of CTL epitopes of the vaccines (46). Lastly, a TAT sequence (11aa) was appended to the C-terminal of the modeled vaccine to allow intracellular delivery (47).

### Antigenicity, Allergenicity, and Biophysical Analyses of the Vaccine Construct

The antigenicity of the vaccine construct was predicted by the VaxiJen v2.0 server, keeping a threshold of 0.5 (31). Thus, the constructed vaccine should be highly antigenic to induce a fast and efficient immune response. Afterward, the AllerTop v2.0 server was used to analyze the allergenicity of the vaccine construct. The biophysical study of the built vaccine was then performed using the ProtParam service (https://web.expasy.org/protparam/) (48). MHCII-NP (http://tools.iedb.org/mhciinp/) on the IEDB server and NetChop3.1 (http://www.cbs.dtu.dk/services/NetChop/) server were used to analyze proteasome cleavage of the final BF Vaccine (BFV) construct (49, 50).

### Secondary and Tertiary Structure Prediction of the Vaccine Construct

Following the biophysical analysis, secondary structure prediction of BFV was performed using the PRISPRED 4.0 prediction method of PRISPRED (http://bioinf.cs.ucl.ac.uk/psipred/) server, keeping all the parameters as default. The server efficiently predicts the proportion of amino acids in alpha-helix, beta-sheet, and coil structures (51–55). Next, the RaptorX webserver was used to predict the tertiary or 3D structure of the vaccine construct. The predicted tertiary structure of the BFV was then refined using the GalaxyRefine module of the GalaxyWEB server (http://galaxy.seoklab.org/). In addition, the SWISS-MODEL Workspace (https://swissmodel.expasy.org/assess) was used for the structural evaluation of the refined protein (56). For protein validation, another online tool, ProSA-web (https://prosa.services.came.sbg.ac.at/prosa.php), was utilized in conjunction with SWISS-MODEL workspace. The z-score generated by the server for each query protein represents the quality of the protein structures. A query protein with a z-score that falls within the range of the z-scores of all experimentally determined protein chains in the existing PDB database signifies the higher quality of the target protein (57).

### Protein Disulfide Engineering of the Vaccine

The vaccine protein disulfide engineering was performed to understand the conformational stability of folded proteins, using Disulfide by Design 2 (http://cptweb.cpt.wayne.edu/DbD2/) server (58). During the analysis, the χ3 angle was set at -87° or +97° 10 while the Cα-Cβ-Sγ angle was kept at its default value of 114.6° ± 10. Residue pairs that possess energy less than 2.2 Kcal/mol were selected and altered to cysteine residues to form disulfide bridges (59). Because 90% of native disulfide bonds have an energy value of less than 2.2 Kcal/mol, 2.2 Kcal/mol was used as the threshold for selecting the disulfide bonds (58).

### Protein-Protein Docking

When TLR proteins encounter vaccines, they induce strong immunological responses to produce immunity towards that particular vaccine, thus mimicking the initial immune response against pathogenic infections (60). Therefore, the constructed vaccines should have a binding affinity with the TLRs. In protein-protein docking, the developed vaccine was docked against numerous TLRs i.e., TLR-1 (PDB ID: 6NIH), TLR-2 (PDB ID: 3A7C), and TLR-4 (PDB ID: 4G8A). Three separate online tools were used to perform protein-protein docking to increase prediction accuracy. The docking was first done with ClusPro v2.0 (https://cluspro.bu.edu/login.php), where the lower the energy score, the better the binding affinity. The energy score is calculated by the ClusPro server using the equation *E* = 0.40*Erep* + (−0.40*Eatt*) + 600*Eelec* + 1.00 *EDARS* (61, 62). The docking was then conducted by the PatchDock server (https://bioinfo3d.cs.tau.ac.il/PatchDock/php.php) and refined by the FireDock server (http://bioinfo3d.cs.tau.ac.il/FireDock/php.php) (63–65). Finally, the third round of docking was executed by the HawkDock server (http://cadd.zju.edu.cn/hawkdock/) in conjunction with the Molecular Mechanics/Generalized Born Surface Area (MM-GBSA) study (66). Lower output scores in these servers translate to higher binding affinity and vice versa (67). Discovery Studio Visualizer was used to visualize the most effective vaccine-TLR combination (68).

### Conformational B-Cell Epitope Prediction

The conformational B-cell epitopes of the constructed vaccine were predicted using the IEDB ElliPro tool (http://tools.iedb.org/ellipro/), with all parameters set to default (22). In addition to cell-mediated immunity, humoral immunity is essential in fighting against infections inside the human body. The body’s humoral immunity depends on B-cells, which secrete antibodies in response to antigens. As a result, the vaccine construct should have effective conformational B-cell epitopes to boost humoral immunity.

### Molecular Dynamics Simulation

Molecular Dynamic (MD) simulations were performed using the GROMACS 2020.3 simulation package (69). The AMBER99SB-ILDN force field was combined with the TIP3P water model (70). Each system received 0.15 M KCl. Potential energy minimization with a step size of 1 fs to the maximum force of 1000.0 kJ/mol/nm was used to relax the structure and avoid steric clashes in subsequent simulations. The system’s pressure and temperature were equilibrated to 1 atm and 310K, respectively, by running NVT and NPT simulations (100-ps each). The system’s pressure and temperature were regulated using a modified Berendsen thermostat and a Parinello-Rahman barostat with time constants tau t = 0.1 ps and tau p = 2 ps, respectively (71, 72). Productive 200 ns MD simulations with a 2-fs time-step were performed in the isothermal-isobaric ensemble for all six systems. The LINCS algorithm constrains the hydrogen-atom bonds (73). The Particle-Mesh Ewald summation scheme was used to calculate long-range electrostatic interactions (74).

### Immune Simulation of Vaccine

Using the C-ImmSim server (https://kraken.iac.rm.cnr.it/C-IMMSIM/index.php), an immune simulation analysis of the vaccine construct was conducted to estimate its immunogenicity and immune response profile. The server uses machine learning algorithms and a position-specific scoring matrix (PSSM) to predict real-life-like immunological interactions (75). All parameters except the time steps were left at their default levels during the analysis. The number of simulation steps was set to 1050, while the time steps were kept at 1, 84, and 170. Because the recommended period between two subsequent doses of most commercial vaccinations has been proven to be four weeks, administration of three injections would need to be spaced four weeks apart (76). The figures were used to calculate the Simpson’s Diversity Index, D.

### Codon Adaptation and *In Silico* Cloning

Because an amino acid might be coded by more than one codon in different organisms, codon adaptation is used to predict the most appropriate codon that efficiently encodes the required amino acid in a particular organism. The Java Codon Adaptation tool (http://www.jcat.de/) was used to optimize the codon adaptation study of the vaccine protein, and the prokaryotic *Escherichia coli* (*E. coli*) strain K12 was chosen as the target organism for increasing the expression efficiency of the final vaccine protein (77). In the server, rho-independent transcription termination, prokaryotic ribosome binding site, and cleavage site of restriction enzymes were avoided to ensure the vaccine gene’s accurate translation. Instead, XhoI and BamHI restriction endonuclease sites were added to the vaccine’s N and C terminals, respectively. The modified DNA of the constructed vaccine was then incorporated between the *XhoI* and *BamHI* genes in the pET28a (+) vector using the SnapGene restriction cloning software (78). The ubiquitin-like modifier or SUMO tag and 6XHis-tag in the pETite vector plasmid aid in the recombinant protein’s solubilization and viable affinity purification (79).

### Analysis of the Vaccine mRNA Secondary Structure

Two online tools, Mfold version 2.3 (http://www.unafold.org/mfold/applications/rna-folding-form-v2.php) and RNAfold (http://rna.tbi.univie.ac.at/cgi-bin/RNAWebSuite/RNAfold.cgi) were used to predict the mRNA secondary structure prediction of the vaccine gene. Both of these servers thermodynamically predict mRNA secondary structures and report the least free energy (ΔG Kcal/mol) for each of the structures created. The folded mRNA is more stable when the minimum free energy is lower and vice versa (80–83). To analyze mRNA folding and secondary structure of the vaccine, the DNA<->RNA->Protein tool (http://biomodel.uah.es/en/lab/cybertory/analysis/trans.htm) was used to convert the newly adapted DNA sequences from the JCAt server into its probable RNA sequence. The generated RNA sequence was then copied and pasted into the Mfold and RNAfold servers for prediction, with all parameters set to default.

## Results

### Identification and Retrieval of the Protein Sequences

FTR1 protein of four different virulent funguses was selected after reviewing the literature from the NCBI database (**Supplementary Table S1**). The selected fungi were *Rhizopus oryzae, Rhizopus delemar RA* 99-880 (a reclassified *Rhizopus oryzae), Rhizopus azygosporus, Rhizopus stolonifer, and Mucor circinelloides 1006PhL.* The FTR1 protein sequence of the *Rhizopus delemar RA 99-880* was the only reviewed protein sequence in the Uniprot database (Entry name: FTR1_RHIO9) (https://www.uniprot.org/), considered as the model sequence to carry out the experiments.

### Epitope Mapping

For epitope mapping, the sequence of the FTR1 protein of *R. delemar* was evaluated, and a total of 309 epitopes were screened out for further research. After screening the FTR1 protein against all the CTL superfamilies in NetCTL-1.2 Server, epitopes were predicted based on the predicted scores. A total of 182 epitopes with higher scores (>1.00 signifies stronger binding affinity) were enlisted (**Supplementary Table S2**). Further, the top 122 HTL epitopes with low percentile scores (<1.00 represents better binding affinity) in the IEDB server were chosen (**Supplementary Table S3**). Finally, the LBL epitopes were predicted using the BepiPred linear epitope prediction method 2.0 of the IEDB prediction server. The binder’s protein score to B-cell was an average of 0.450, a minimum of 0.189, and a maximum of 0.693. Since all values were equal or higher than the default threshold of 0.500, all the epitopes were considered LBL epitopes (**Supplementary Figure S1**). Additionally, the average surface accessibility area of the FTR1 protein was found to be 1.000, a maximum of 5.900, and a minimum of 0.025 in Emini’s surface accessibility prediction test for a potent LBL epitope. Values ≥ the default threshold of 1.000 were selected (**Supplementary Table S3**). In the end, 5 LBL epitopes of 8 to 20 AA were chosen for further analysis (**Supplementary Table S4**). The ElliPro prediction server filters out antigenic residues from the 3D model of the FTR1 protein to predict the conformation of B-cell epitopes (**Table 1**). The lowest score and maximum distance (Angstrom) in the default mode were calibrated to 0.5 and 6, respectively.

**Table 1:** List of the promising conformational B-cell epitopes.

No.	Residues	Number of residues	Score
1	A:F304, A:F307, A:K310, A:R311, A:A312, A:A313, A:I314, A:R315, A:K316, A:A317, A:E318, A:A319, A:G320, A:E321, A:W322, A:D323, A:D324, A:G325, A:D326, A:E327, A:A328, A:E330, A:N331, A:Q334, A:Y335, A:G337, A:N338, A:D339, A:G340, A:E341, A:I343, A:V344, A:E345, A:D346, A:K347, A:E348, A:S349, A:D350, A:E351, A:E352, A:A353, A:N354, A:N355, A:H356, A:P357, A:K358, A:I361	47	0.765
2	A:F36, A:N37, A:T38, A:E39, A:S40, A:P41, A:V42, A:Y43, A:K44, A:R45, A:R47, A:N48, A:Q49, A:W51, A:I52, A:V118, A:K119, A:A121, A:K122, A:A123, A:M124, A:Q125, A:K126, A:S127, A:N128, A:S129, A:E130, A:K131, A:S132, A:S133, A:F134, A:K135, A:E136, A:K137, A:L138, A:Q139, A:K140	37	0.735
3	A:A66, A:A67, A:I69, A:A70, A:V71, A:Y72, A:Y73, A:T74, A:V75, A:L76, A:N77, A:D78, A:L79, A:W80, A:G81, A:N82, A:S83, A:I86, A:E228, A:Q229, A:N230, A:A231, A:W232, A:N233, A:Q234, A:V235, A:I236, A:G237, A:G238, A:E239, A:A240, A:A241, A:D242, A:V243, A:I244, A:S245, A:Y246, A:V248, A:S249, A:T250, A:A251, A:V252, A:W253, A:H254, A:V255, A:S256, A:W257, A:G258, A:D259, A:P260, A:E261, A:A262, A:N263, A:N264, A:D265, A:T266, A:S267, A:G268, A:N280, A:N281, A:T282, A:A283, A:T284, A:Y285, A:I289	65	0.732
4	A:M1, A:S2, A:Q3, A:D4, A:L5, A:F6, A:S166, A:L167, A:G168, A:I169, A:Q170, A:G171, A:K172, A:S173, A:I174, A:P175, A:I176, A:I179, A:M180	19	0.724
5	A: F59, A: L62, A: C63	3	0.61

### Selection of the Most Promising Epitopes

Fully conserved epitopes of the targeted fungal species were further used for the conservation analysis. Afterward, antigenicity, allergenicity, toxicity, cytokine inducing ability, and transmembrane topology of the identified epitopes were analyzed. Epitopes found to be highly antigenic (>0.5), non-allergenic, non-toxic, 100% conserved among selected species, and strong cytokine inducing ability (for HTL epitopes) was considered the most promising epitopes for the vaccine development process (**Table 2**).

**Table 2:** Promising CTL epitopes with their scores (>1.000), antigenicity, allergenicity toxicity, and conservancy.

HLA Supertype	Epitopes	Scores (>1.000)	Antigenicity (Threshold >0.5)	Allergenicity	Toxicity	Conservancy
A2	FIGGVSLGI	1.0756	Antigen	Non-allergen	Non-toxin	100%
A3	RMQEKWKVK	1.1218	Antigen	Non-allergen	Non-toxin	100%
A24	IQLRWFFVF	1.5956	Antigen	Non-allergen	Non-toxin	100%
B27	LRWFFVFST	1.0815	Antigen	Non-allergen	Non-toxin	100%
B39	MQEKWKVKL	1.5459	Antigen	Non-allergen	Non-toxin	100%
B44	RETTEAAII	1.0679	Antigen	Non-allergen	Non-toxin	100%
B62	IGAAFIAVY	1.2503	Antigen	Non-allergen	Non-toxin	100%

In addition, two HTL epitopes were found to have low percentile scores (less than 1.00) and appreciable cytokine-producing capability (IFN-gamma, IL-4, and IL-10) (**Table 3**).

**Table 3:** Promising HTL epitopes with their percentile rank, antigenicity, allergenicity, toxicity, conservancy, and cytokine inducing ability (IFN-gamma, IL-4, and IL-10).

Peptide	Allele	Percentile Rank (<1.00)	Antigenicity (>0,5)	Allergenicity	Toxicity	Conservancy	IFN-gamma inducing capability	IL-4 inducing capability	IL-10 inducing capability
AFIAVYYTVLNDLWG	HLA-DPA1*02:01/DPB1*01:01	0.66	Antigen	Non-allergen	Non-Toxin	100%	Inducer (Score: 0.14286936)	Inducer (Score: 0.38)	Inducer (Score: 0.423549064759)
FIAVYYTVLNDLWGN	HLA-DPA1*02:01/DPB1*01:01	0.78	Antigen	Non-allergen	Non-Toxin	100%	Inducer (Score: 0.0092719089)	Inducer (Score: 0.38)	Inducer (Score: 0.327536699979)

**Figure 3:**
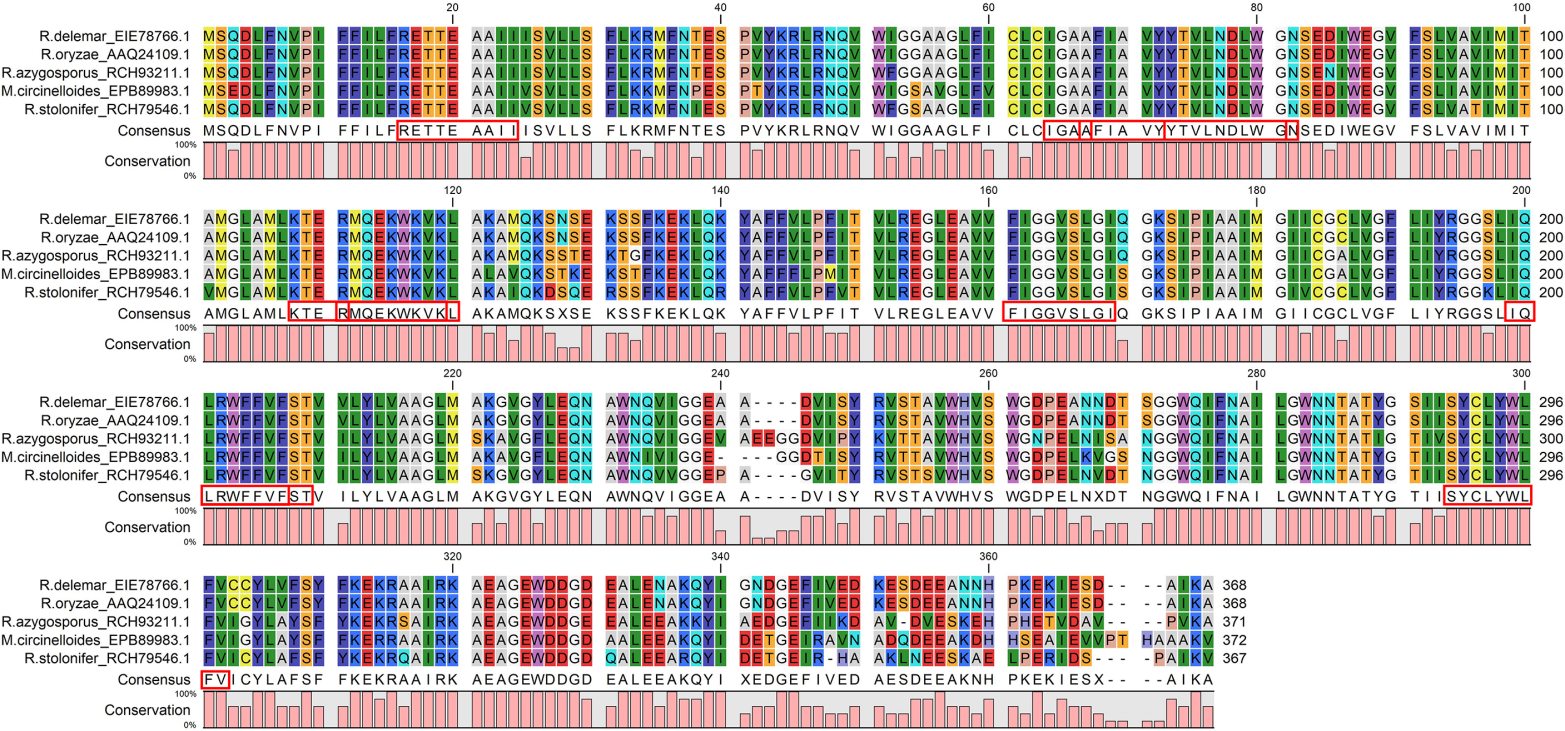
The CTL, HTL, and LBL epitopes were fully conserved across all the chosen virulent fungi and satisfied all the selection criteria: antigenicity, allergenicity, non-toxic, cytokine inducing ability. The red boxes indicate the epitopes.

The Emini score was considered for selecting LBL epitopes, which revealed KTERMQEKWKVK as the best sequence with a score of 3.077, highly antigenic, non-allergenic, and non-toxic. Therefore, we selected eleven CTL, HTL, and LBL epitopes as the most promising epitopes for the peptide fusion, fully conserved among the selected species (**Figure 3**).

### Peptide Fusion

**Figure 4:**
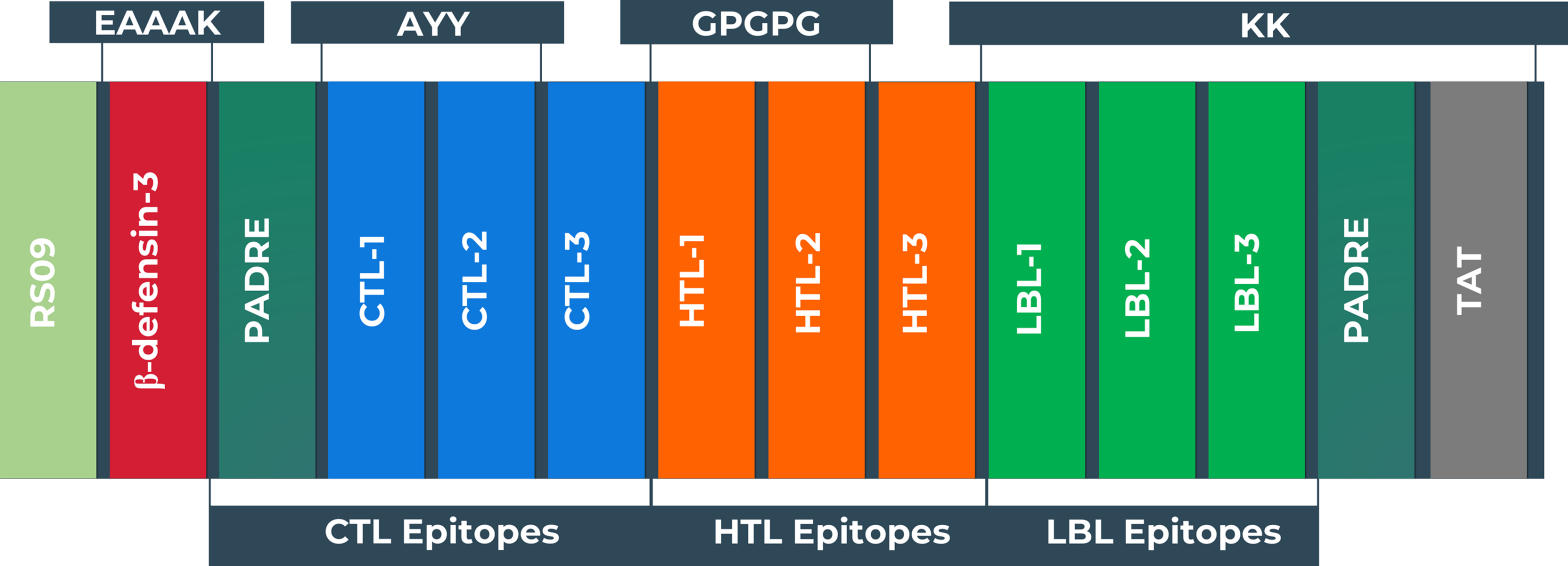
Schematic representation of the vaccine constructs with its linkers (EAAAK, AAY, GPGPG, KK), adjuvant (RS09 and human beta-defensin-3), PADRE, and TAT sequence, and epitopes (CTL, HTL, LBL) in a sequential manner.

The vaccine was constructed using the eleven most promising epitopes. RS09 and beta-defensin-3, appended at the N-terminal of the vaccine construct, were used as an adjuvant. In the C-terminal of the vaccine, the PADRE and TAT sequence were conjugated to act as strong immunity inducers. EAAAK, AAY, GPGPG, and KK linkers were employed in the correct positions to connect the epitopes. The newly developed vaccine is designated as the BF vaccine (BFV). **Figure 4** depicts a schematic representation of the constructed vaccine BFV.

### Antigenicity, Allergenicity and Biophysical Analyses of the Vaccine Construct

The newly constructed BFV was potent antigen and non-allergenic (**Supplementary Table S5**). The biophysical studies revealed that BFV possesses a high (primary) theoretical pI (9.97). The chemical formula of the vaccine protein can be denoted as C_1323_H_2034_N_342_O_322_S_10_, contains 4031 atoms, and has a molecular weight of 28203.40. It has a half-life of 4.4 hours in mammalian reticulocytes *in vitro*, >20 hours in yeast *in vivo*, and more than 10 hours in the *E. coli* cell culture system, which was satisfactory. Moreover, the GRAVY value of the vaccine design was 0.111, indicating that the protein is hydrophobic. The extinction coefficient was 80705 M^-1^ cm^-1^ at 280 nm measured in water. BFV was also shown to have a high solubility score of 0.640 in the Protein-sol server. Proteasome cleavage analysis using MHCII-NP from the IEDB server and NetChop3.1 server revealed that BFV was found to be cleaved by proteasomes to give rise to the predicted T- cell epitopes (**Supplementary Tables S6**, **S7**).

### Prediction of the Secondary and Tertiary Structure of the Vaccine Construct

**Figure 5:**
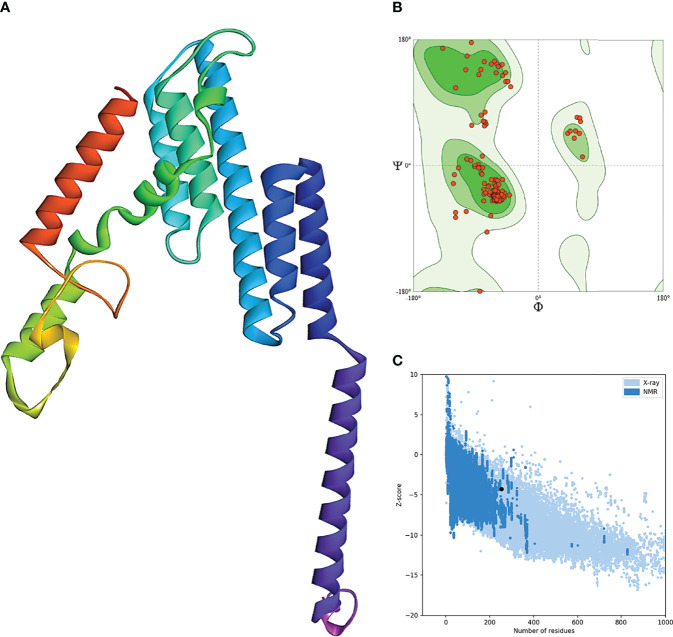
Refinement and validation of the modeled tertiary structure of BFV. **(A)** represents the refined 3D structure of BFV. **(B)** displays the Ramachandran plots of refining the 3D structure of BFV using the SWISS-MODEL Workspace. Finally, **(C)** depicts the Z-Score plot for the 3D structure of BFV, which contains the z-scores of all experimental protein chains determined by NMR spectroscopy (dark blue) and X-ray crystallography (light blue).

The secondary structure prediction of BFV displayed by the PSIPRED server revealed that its alpha-helix structure comprised the highest percentage of amino acids while the lowest proportion was discovered in the β-strand (**Supplementary Figure S3**). The RaptorX server was used to generate the 3D structure of BFV, which was then refined so that the 3D BFV protein could closely resemble the native protein structures. The refined 3D structure of BFV was further evaluated by the Ramachandran plot generated by the SWISS-MODEL Workspace and validated by the ProSA-web server. The Ramachandran plot analysis showed that BFV had a 96.43% favored region with 0.00% outliers and included A254 LYS A23 ARG in the Rotamer outliers (1.09%). In addition, BFV has a z-score of -4.38, which is within the range of all experimentally established X-ray crystal structures of proteins in the Protein Data Bank. Moreover, protein validation investigation confirmed the high quality of the revised structure (**Figure 5**).

### Disulfide Engineering of the Vaccine Protein

The Disulfide predicted the disulfide bonds of BFV by Design 2 server. Based on a selection criterion, the server determines the possible sites within a protein structure with the greater possibility of pairs of amino acids that can form disulfide bonds. Only amino acid pairs with a bond energy of less than 2.2 kcal/mol were chosen for this experiment. Four amino acid pairs in BFV protein were found to have bond energy less than 2.2 kcal/mol: 23 Arg and 59 Ala, 62 Ala and 65 Val, 119 Trp and 122 Val, and 213 Lys 216 Arg. These selected amino acid pairs were used to form the mutant version of the original BFV by forming disulfide bonds in the Disulfide by Design 2 server (**Supplementary Figure S4**).

### Protein-Protein Docking Analysis

**Figure 6:**
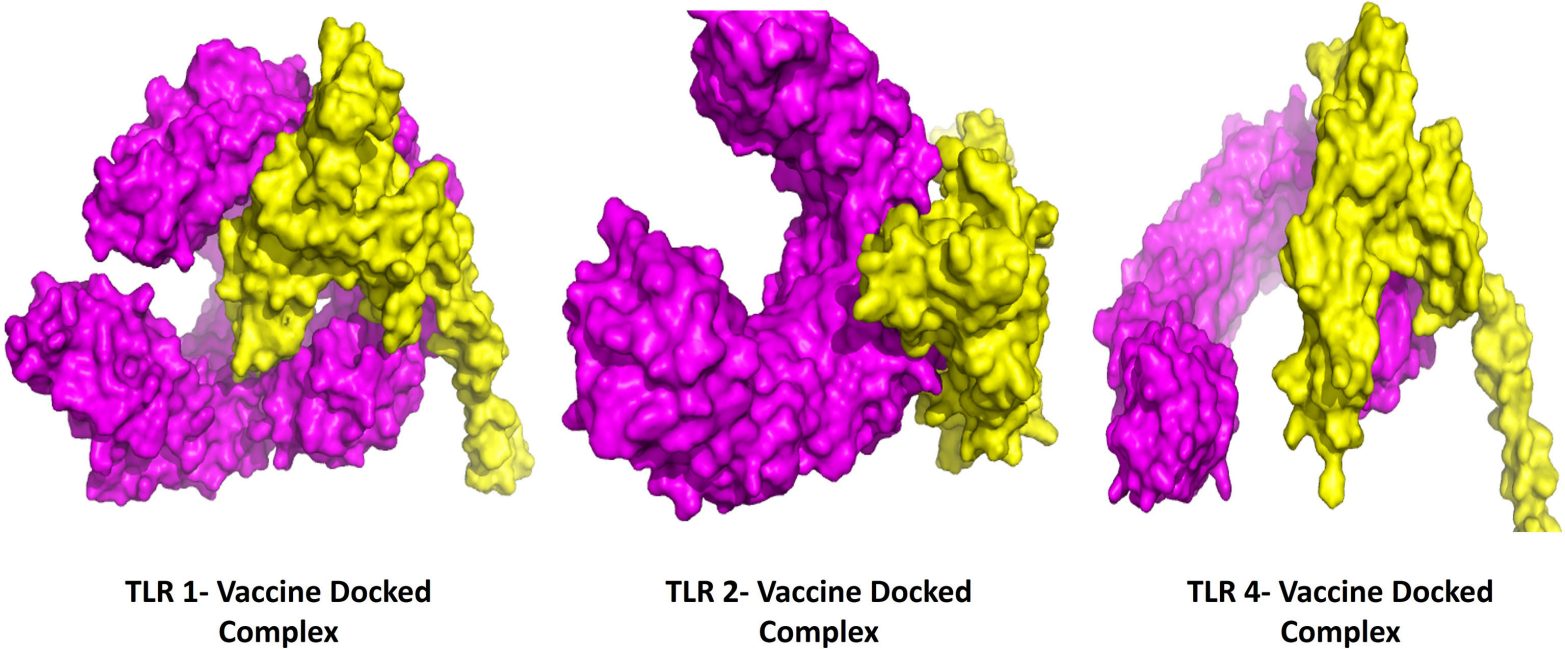
The interactions of the BFV with several TLRs using the Discovery Studio Visualizer.

The protein-protein docking study was performed to evaluate the ability of BFV to interact with different TLRs. Three online tools were used to perform protein-protein docking to improve prediction accuracy. When docked by ClusPro 2.0, it demonstrated very high binding affinities with all of its targets. In PatchDock and HawkDock, BFV has also shown excellent interaction with all of its targets. As a result, it may be argued that the BFV should elicit a strong and consistent immune response when administered (**Supplementary Table S8**). Finally, the discovery Studio Visualizer shows the interactions of the BFV with several TLRs (**Figure 6**).

### Conformational B-Cell Epitope Prediction of the Vaccine

**Figure 7:**
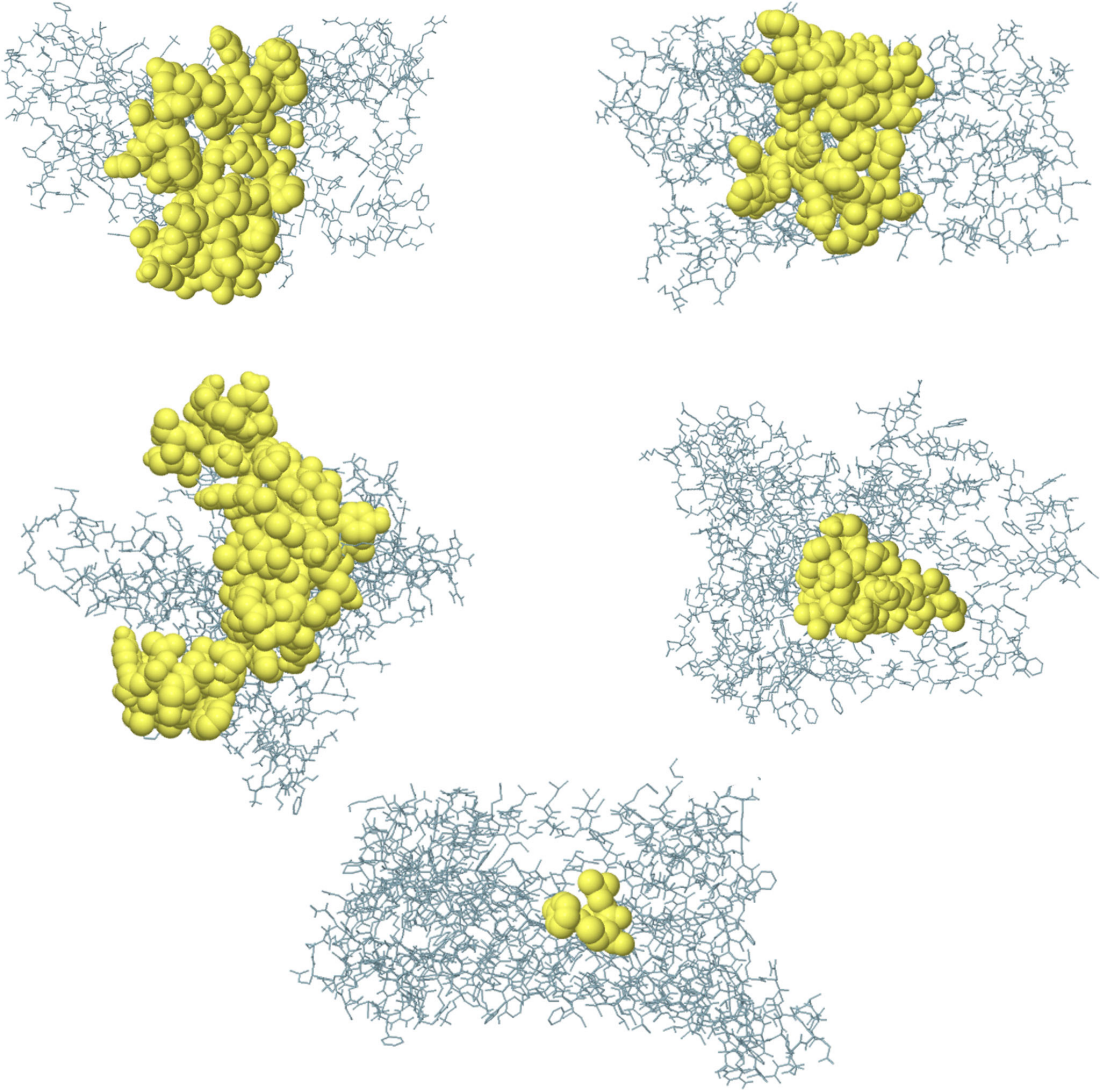
Three-dimensional representation of conformational B cell epitopes of BFV. In A-G, the bulk of the polyprotein is represented in grey sticks, and the yellow surfaces represent the conformational B cell epitopes.

The conformational B-cell epitope prediction of BFV revealed seven regions within the vaccine with scores ranging from 0.566 to 0.913, spanning a total of 120 amino acid residues (**Figure 7**). **Supplementary Table S9** shows the individual scores of each of the conformational B-cell epitopes.

### Molecular Dynamics Simulation

**Figure 8:**
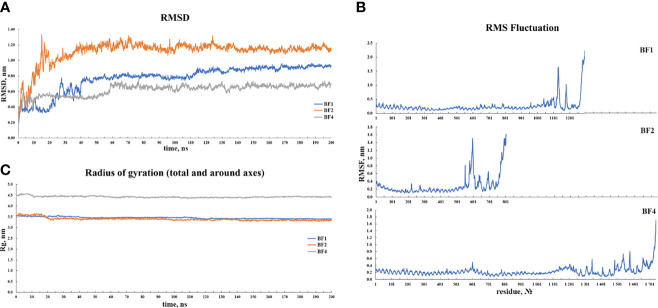
Results of the **(A)** RMSD, **(B)** RMSF and, **(C)** radius of gyration analyses of the three vaccine-TLR docked complexes.

For 200 ns simulations, trajectory analysis was performed. Since the periodic boundary conditions were met during the simulation, the protein molecules in the complex were re-centered and returned to the simulation cell. Following that, an initial analysis of the trajectories was performed, which included calculating the RMSD, radius of gyration, and RMSF. **Figure 8** depicts the corresponding graphs. **Figure 8A** depicts the RMSD changes. **Figure 8A** shows that the RMSD stabilizes around 0.8 for BF1, around 1.0 for BF2, and around 0.6 for BF4. Given the size of the simulated systems, this indicates that they are very stable. **Figure 8B** shows that the radius of gyration for each complex tends to be stable, indicating that the compactness of complexes does not change significantly during simulation. **Figure 8C** depicts the RMS fluctuations for the complexes’ C-alpha atoms. According to the graphs, the mobility of the receptor atoms is significantly lower than that of the vaccine atoms.

### Immune Stimulation

**Figure 9:**
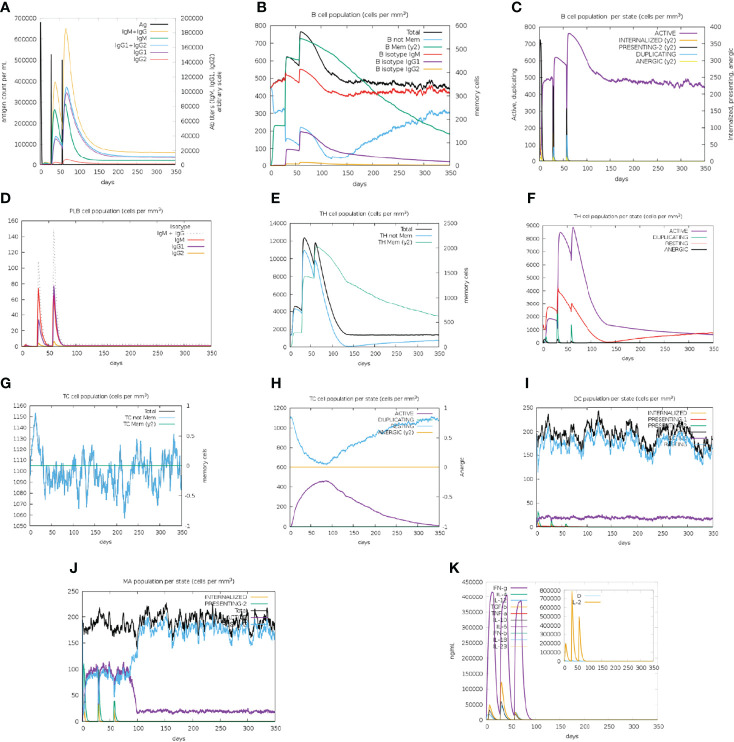
C-IMMSIMM representation of the immune simulation of BFV. **(A)** The immunoglobulin and immunocomplex response to BEV administration (lines colored in black) and the subclasses are depicted by colored lines, **(B)** Increment in the B-cell population throughout the three injections, **(C)** Rise in the B-cell population per state throughout vaccination, **(D)** Surge in the plasma B-cell population throughout the injections, **(E)** Rise in the helper T-cell population throughout the three injections, **(F)** Elevation in the helper T-cell population per state throughout the vaccination, **(G)** Enhancement in the cytotoxic T lymphocyte population throughout the injections, **(H)** Augmentation in the active cytotoxic T lymphocyte population per state throughout vaccination, **(I)** Increment in the active dendritic cell population per state throughout the three injections, **(J)** Increase in the macrophage population per state over the course of the injections, **(K)** Surge in the concentrations of different types of cytokines throughout the three injections.

The BFV elicited both robust primary and secondary immune responses. After administration of the three doses of BFV, increased levels of active B cells (**Figures 9B, C**), plasma B-cell (**Figure 9D**), helper T-cell (**Figure 9E, F**), and cytotoxic T-cell (**Figures 9G, H**) along with gradually elevated levels of different immunoglobulins (**Figure 9A**) were predicted. At the same time, the vaccine stimulates helper T-cells which eventually result in an improved adaptive immunity (84, 85). Again, improved immunological memory development, greater antigen clearance rate, increased dendritic cells, and macrophages suggest outstanding antigen presentation by the Antigen Presenting Cells, i.e., dendritic cells and macrophages (**Figures 9I, J**). Moreover, the vaccines induce diverse cytokines such as IFN-gamma, IL-23 (interleukin-23), IL-10, and IFN-beta (interferon-beta), that instigate immune response against pathogens and defend the body (86, 87). BFV was also shown to induce different types of cytokines (**Figure 9K**). Simpson’s Index (D) was measured as a nominal value, signifying that the vaccine has a more diverse effect in the analysis (88). Thus, the immune simulation study findings strongly suggest that BFV is a promising vaccine that stimulates a robust immunogenic response against FTR1 protein after its administration.

### Codon Adaptation, *In Silico* Cloning, and Analysis of the Vaccine mRNA Secondary Structure

**Figure 10:**
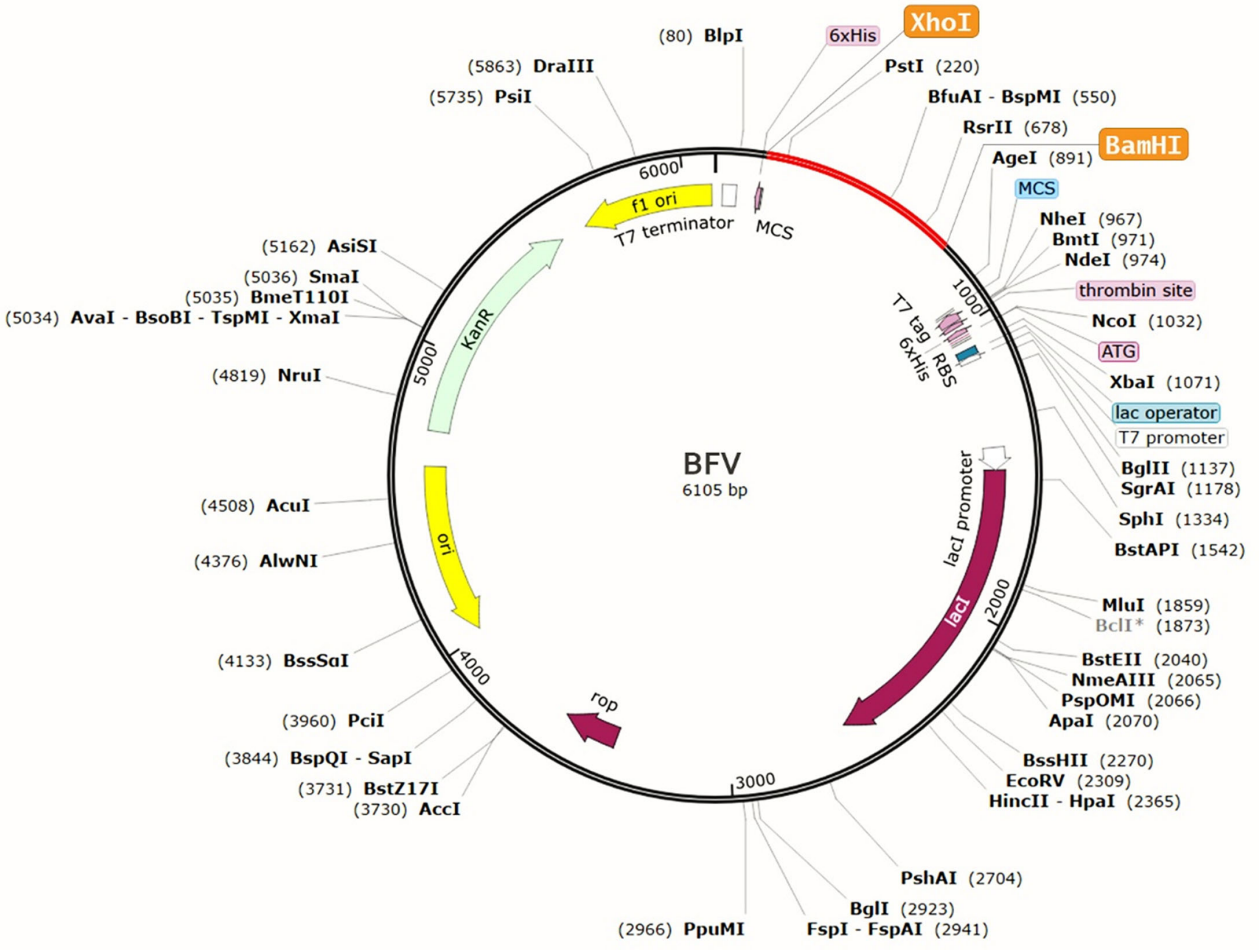
Recombinant pETite vector plasmid with the BFV vaccine inserted (marked in red color) between the XhoI and BamHI restriction enzyme sites.

The protein sequence of BFV was adapted in the JCat server for codon adaptation. BFV had a codon adaptation index (CAI) value of 0.798, suggesting that the DNA sequences of BFV contain a higher proportion of the codons that are expected to be used in the cellular machinery of the target organism *E. coli* strain K12 (**Supplementary Figure S5**). As a result, it was predicted that the *E. coli* strain K12 would produce the BFV efficiently (80, 81). Furthermore, the produced sequence had a GC content of 49.74%. Afterward, the predicted DNA sequence of BFV was introduced into the pETite vector plasmid between the XhoI and BamHI restriction sites. **Figure 10** depicts the newly created recombinant plasmid. SUMO tag and 6X His tag of the vector plasmid facilitate vaccine purification during the downstream processing (89). Following that, the Mfold and RNAfold servers predicted the secondary structure of the BFV mRNA. The Mfold server calculated a minimum free energy score of 199.10 kcal/mol, consistent with the RNAfold server’s -225.80 kcal/mol prediction. **Supplementary Figure S6** depicts the secondary structure of BFV mRNA.

## Discussion

Mucormycetes thrive in iron-rich environments, and therefore, the prevention of this condition can be considered a potential therapeutic approach. These iron-rich surroundings may be provided due to host iron availability, and through the use of deferoxamine, the iron chelator, which is also used to alleviate serum iron levels in COVID-19 patients. Acquisition of host iron by fungi involves a transit component, a part of which is encoded by the *FTR1* gene. Notably, all three mechanisms of fungi host iron uptake involve the transit component. With surgical debridement and antifungal therapy as the only treatment options, targeting this transit element or FTR1 protein may lead to improved outcomes of mucormycosis. Here, with the help of an immunoinformatics approach, we designed a potential polyvalent vaccine against the different virulent Rhizopus and Mucor fungus of mucormycosis, targeting the principal target component aiding the fungus iron entry. Apart from ensuring access to high throughput immunological analysis, immunoinformatic also eliminates finance-associated limitations and those concerning applications regarding vaccine constructions. By evaluating multiple factors, this experimental design assembles and represents a potential epitope-based polyvalent vaccine targeting the key virulent protein FTR1 of the three Rhizopus and one Mucor species selected for this experimentation. Both CTL epitopes and HTL epitopes were considered for prediction to produce a highly efficacious vaccine, which involved a stimulated humoral immune response, leading to a decline in antigen and antibody over time, and the cellular immune response, which comprises more robust and indefinite protection encompassing the cytokine secretion and targeted action - generating potentially the most effective multi-epitope subunit vaccine.

The B cell surface comprises antigenic B cell receptors (BCR), through which they recognize solvent-exposed antigens of B cell epitopes and further trigger the secretion of antibodies (90). The differential labels of B cell epitopes, either continuous linear or discontinuous, were identified with the IEDB prediction server using the BepiPred linear epitope prediction method and the ElliPro prediction tool. These led to the initial selection of 182 MHC Class-I epitopes, 122 MHC Class-I epitopes, and 5 B-cell epitopes, which were further evaluated through rigorous screening methods to identify all the antigenic, non-allergenic, non-toxic, and 100% conserved epitopes *via* VaxiJen v2.0, AllerTOP v2.0, ToxinPred and conservancy server, respectively. While these selection criteria enabled the construction of an effective and promising vaccine, the 100% conservancy ensured the broad-spectrum activity of the vaccines over the selected virulent fungi strains. Following this, the cytokine triggering factor of epitopes was evaluated through IFNepitope, and IL4pred and IL10pred servers were used for the determination of IFN-gamma induction, as well as to determine IL-4 and IL-10 inducing properties of the HTL epitopes, respectively, which was contributory to the role of cytokines, which are essential in establishing a communicative network among the cells of the immune system during an immunogenic response (35, 91).

Consequently, a total of 11 MHC-I, MHC-II, and B-cell epitopes were selected from the initial lot to construct the vaccine. These epitopes meet the specific screening criteria and potentially establish the most promising network as part of its immunogenic response. The ideally best-selected CTL, HTL, and BCL epitopes were combined utilizing different linkers - EAAAK, AAY, GPGPG, and KK at varying positions. Incorporating the EAAAK linker at the vaccine’s start or terminal eliminates its degradation (91). Here, RS09 and beta-defensin-3 were used as adjuvants, contributing to increased antigenicity, immunogenicity, stability, and longevity of the constructed vaccines (92). The vaccine constructs also included PADRE and TAT sequences, which induce strong immunogenic responses. These combinatorial use of adjuvants and linkers and the best-selected epitopes stabilized the vaccine construct and would promisingly have increased the antigenicity, immunogenicity, and longevity. The final construct was labeled “BF Vaccine (BFV).”

Subsequently, the crucial properties concerning the BF vaccine, including antigenicity, allergenicity, and biophysical properties, were determined. Apart from showing antigenic characteristics, the vaccine was also found to be non-allergenic, ideal to its trait, for inducing a robust and highly immunogenic response without the causation of an allergic reaction. Moreover, the construct exhibits greatly reassuring stability due to its high pI value of 9.97, a conclusive basic range, the theoretical pI or pH at which the protein contains no integrated charge, and from the analyzed pI value, this was deemed quite achievable. Alongside, the aliphatic index of a protein which is indicative of the relative volume of aliphatic amino acids occupied in its side chains, as well as a thermally stable state measure, was observed to be extensively high for this construct; representing its thermostable nature (92–94). Moreover, the vaccine possibly procured a half-life of 4.4 hours in mammalian reticulocytes *in vitro*, more than 10 hours in E.coli cell culture systems, and more than 20 hours in yeast, *in vivo*, which signifies the potentially constructive mass production effort of the vaccine in E.coli cell culture systems, which is considered to be the ideal preference for mass production while ensuring the emergence of stable recombinant proteins.

Furthermore, the extinction coefficients were found to be 80705 M-1 cm-1 at 280 nm measured in water, and the vaccine was also shown to be highly soluble in the Protein-sol server acquiring an overall solubility score of 0.640. A proteasome cleavage analysis of the BF vaccine construct using NetChop3.1, and MHC II-NP on the IEDB server further showed proteasomes generating predicted T- cell epitopes. The secondary structure analysis of BFV enabled the generation of the Ramachandran plot and the z-score using the ProSA-web server. The refined structure obtained from the protein validation study has suggestively complied, representing a potentially ideal structure and indicating the robustness of the vaccine construct. The z score of the vaccine is -4.38, which resides within the range of all experimentally proven X-ray crystal structures of proteins from the Protein Data Bank. Notably, the maximum number of amino acids was present in the best locations.

When the BFV protein was checked for possible Disulfide bond-forming amino acid pairs to generate the mutant version, four amino acid pairs meeting the selection criteria of bond energies less than 2.2 kcal/mol were found. These pairs included - 23 Arg and 59 Ala, 62 Ala and 65 Val, 119 Trp and 122 Val, and 213 Lys and 216 Arg. Following this, additional docking efforts of the BFV protein with different TLRs using the ClusPro 2.0 and PatchDock servers exhibited a high binding affinity across all targets. The high affinity for docking represents the BFV binding with TLRs during an immune response within the body. Additional confirmative results were observed in conformational B-cell epitope prediction of the BF vaccine construct; exhibited seven potential regions capable of acting as conformational B-cell epitopes to the antibodies, with an exceptional score ranging from 0.566 to 0.913, covering a total of 120 amino acid residues. While these results exhibited potentially good interactions and efficacy for the BF vaccine, further MD simulation was carried out to provide a view of the dynamic evolution of the system. MD simulation represents a biological environment, which allows the analysis and evaluation of physical movements and interactions of the atoms of a protein complex with environment molecules for a specified duration. Results obtained from MD simulation exhibit the vaccine stability through varying environmental conditions, including - changing pressure, temperature, and motion. The initial trajectory evaluations, including the calculation of RMSD, the radius of gyration, and RMSF, all correspond to the high stability of the vaccine construct in a biological environment. During the immune simulation analysis, the vaccine triggered a robust primary and secondary immune response. The vaccine administration may have initially mediated the primary immune response followed by the secondary response later. The vaccine-mediated a combined humoral and cell-mediated immune response is indicated by increased cytotoxic T-cells, helper T-cells, memory B-cells, plasma B-cells, and different antibodies (84, 85). A measure of the potentially outstanding antigen presentation was also shown by a rise in the concentration of APCs, including macrophages and dendritic cells. The proof of a robust and effective immune response against the fungus was also presented with additional accretion of the cytokine profile.

Moreover, the limited Simpson index (D) also indicated a diversified immune response of BFV. While the major attributive feature of immune response was found to be potentially effective, the final step, including codon adaptation and *in silico* cloning, was performed to design a recombinant plasmid for the mass production efforts of BFV in the E. coli strain K12. The codon adaptation study also represented results that are considered satisfactory and close to the threshold count; a CAI value of 0.798 and a GC content of 49.74% were found, where any CAI value over 0.80 and GC content within 30-70% are considered to be good scores. After that, the DNA sequence of the BF vaccine was introduced into the pETite plasmid. Additionally, the Mfold server generated a positive, and the RNAfold server-generated negative but much lower minimal free energy of 199.10 kcal/mol and -225.80 kcal/mol, respectively. These values indicate the stability of the vaccine mRNA secondary structure, ensuring significantly consistent stability upon *in vivo* transcription.

These differential outcomes from multiple evaluating servers potentially ensure a productive and robust character of the BF vaccine construct against the virulent Rhizopus and Mucor spp. strains. The construct is assumed to serve a therapeutic purpose against the rare and deadly mucor afflicted fungal infection, which came to much attention amidst the current pandemic owing to its rapid transmission. All evaluative measures of the vaccine model indicate a possible trigger of a strong immunogenic response within the human body. However, further wet lab-based studies on the designed vaccine promise intriguing results which will provide confirmative results concerning its safety, efficacy, and potentiality. In immunology and vaccinology, the current emerging topics include these genome-based technologies for the development of vaccines, which show robust results against infectious diseases and include minimal harmful effects. Utilizing these immunoinformatics approaches and delivering efficacious vaccine constructs in recent times have led to market access and mass acceptability among populations of two developed vaccines using such methods. Our study, including the BF vaccine, is another step towards the field of immunology and vaccinology, as it represents a potential vaccine construct against mucormycosis. Our evaluative measures in verifying our vaccine construct have ensured the possibility of a safe and effective vaccine.

## Conclusion

This study evaluated and targeted the mechanistic entry complex on the host cell, FTR1, for the fungi to develop a potentially robust vaccine construct in varying conditions and species. This high throughput and accurate immunological analyses ensure the construction of a majorly robust and efficacious vaccine - selecting the most highly antigenic (for the vaccine to generate a robust immune response), non-allergenic (no harmful reaction within the body caused by the vaccine), non-toxic, non-human homolog, and 100% conserved epitopes (vaccine ensuring effectivity against different isolates/variants around the world). The vaccine construct will not only serve its role against the rising cases of mucormycosis exhibited amidst the COVID-19 pandemic but is also presumed to be effective against generalized mucormycosis cases. Moreover, alongside efficacy, the vaccine constructs designed through these methods were found to be safe; however, wet-lab-based studies remain a priority. Approval, followed by mass production and availability of this vaccine, may provide a cheap and effective option for the worldwide prevention of the mucormycosis epidemic.

## Data Availability Statement

The original contributions presented in the study are included in the article/**Supplementary Material**. Further inquiries can be directed to the corresponding authors.

## Author Contributions

YA conceived the study. YA and MH designed the study. MH and CZ supervised the study. YA, AM, VT, SS, NF, NA, MP, BS, and MU did the formal analysis. YA, NF, AM, and NA wrote the draft manuscript. YA, MH, and CZ edited and revised the manuscript. All the authors approved the final version of the manuscript.

## Conflict of Interest

The authors declare that the research was conducted in the absence of any commercial or financial relationships that could be construed as a potential conflict of interest.

## Publisher’s Note

All claims expressed in this article are solely those of the authors and do not necessarily represent those of their affiliated organizations, or those of the publisher, the editors and the reviewers. Any product that may be evaluated in this article, or claim that may be made by its manufacturer, is not guaranteed or endorsed by the publisher.
