# Supplementary material for: Immunoinformatic Design of a Multivalent Peptide Vaccine Against Mucormycosis: Targeting FTR1 Protein of Major Causative Fungi
Source: Front Immunol. 2022 May 26;13:863234. doi: 10.3389/fimmu.2022.863234 (PMC9204303; doi:10.3389/fimmu.2022.863234)
Supplement: Supplementary file 1 [file Image_1.pdf]

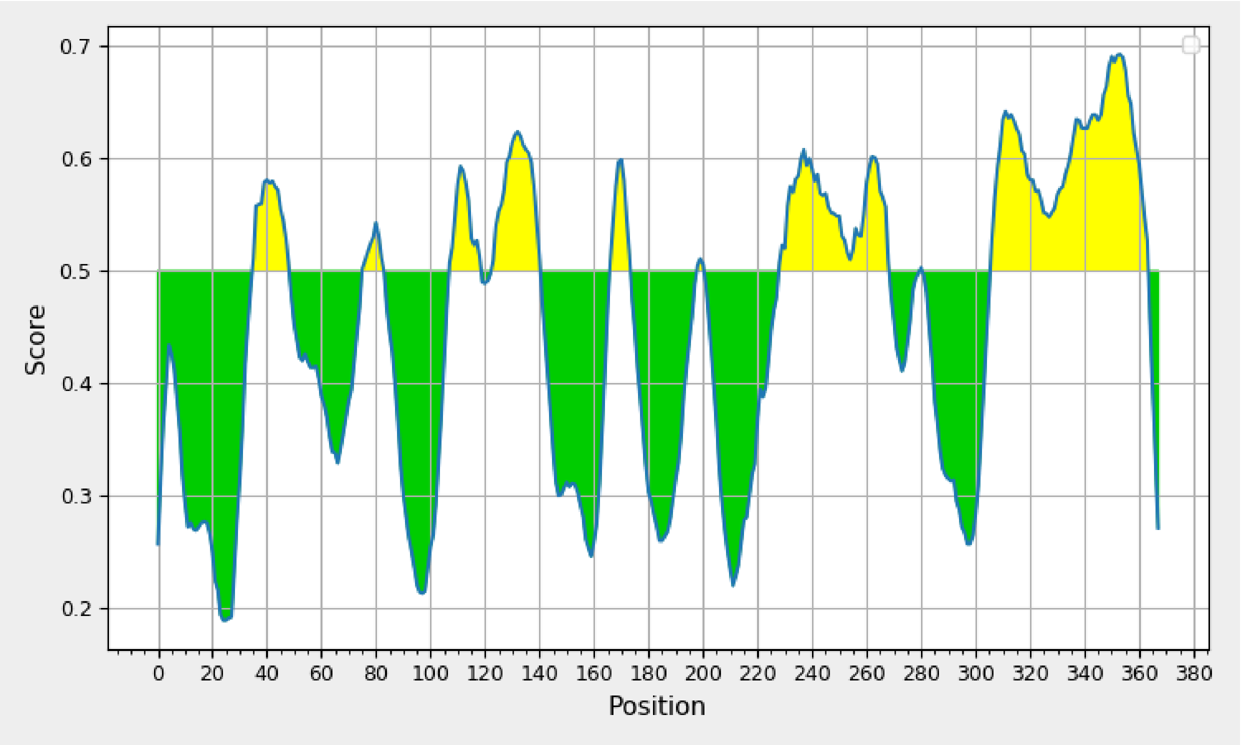

Figure S1. LBL epitopes were predicted using Bepipred linear epitope prediction. Scores above the default threshold of 0.500 denoted by yellow color represent B-cell epitopes.
