# Supplementary material for: Immunoinformatic Design of a Multivalent Peptide Vaccine Against Mucormycosis: Targeting FTR1 Protein of Major Causative Fungi
Source: Front Immunol. 2022 May 26;13:863234. doi: 10.3389/fimmu.2022.863234 (PMC9204303; doi:10.3389/fimmu.2022.863234)
Supplement: Supplementary file 2 [file Image_2.pdf]

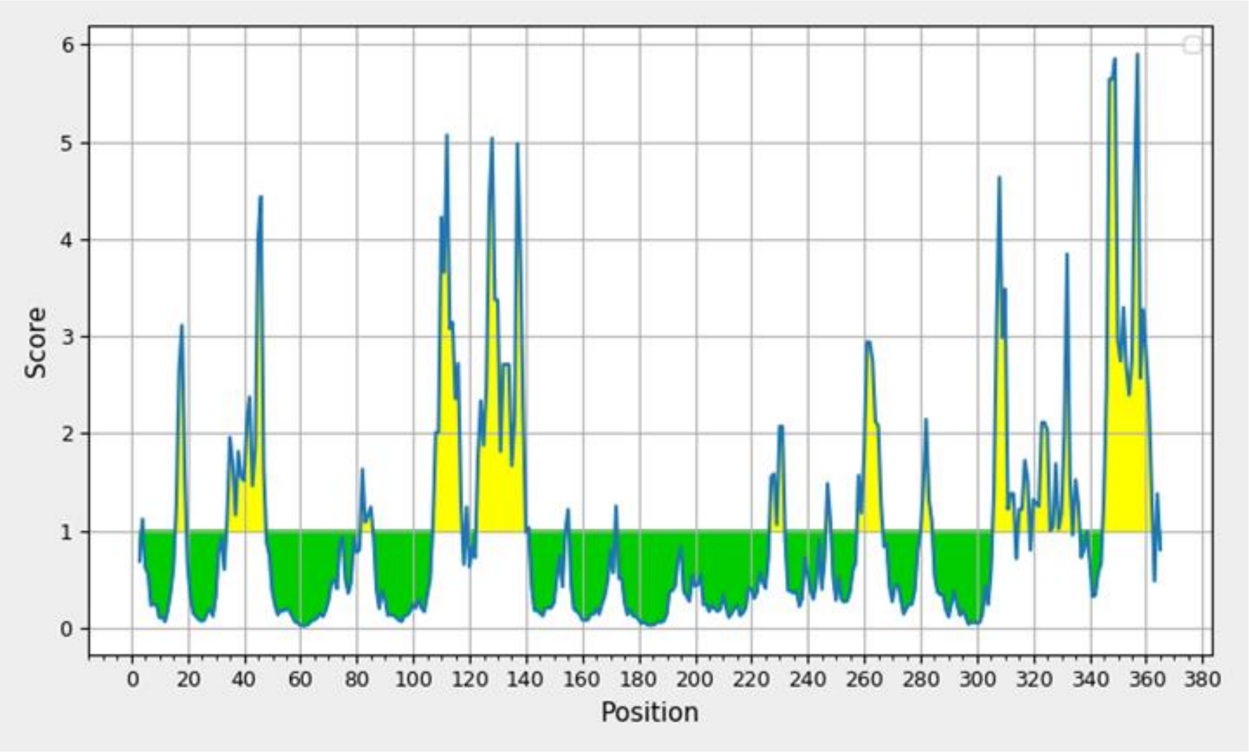

Figure S2: Surface LBL epitopes from Emini's surface accessibility prediction test. Scores above the default threshold of 1.000 denoted by yellow color represent potent LBL epitopes.
