# Supplementary material for: Immunoinformatic Design of a Multivalent Peptide Vaccine Against Mucormycosis: Targeting FTR1 Protein of Major Causative Fungi
Source: Front Immunol. 2022 May 26;13:863234. doi: 10.3389/fimmu.2022.863234 (PMC9204303; doi:10.3389/fimmu.2022.863234)
Supplement: Supplementary file 3 [file Image_3.pdf]

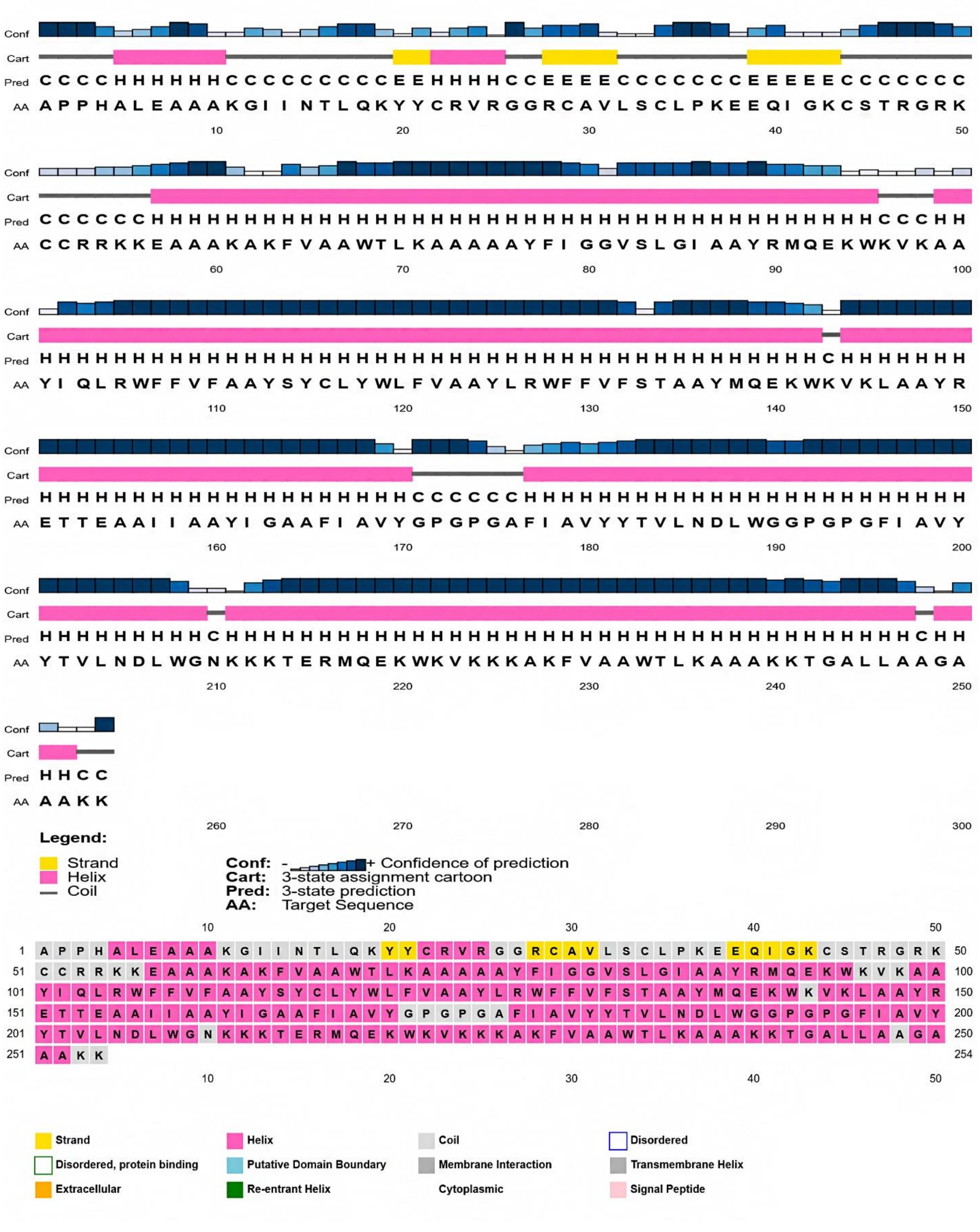

Figure S3. Secondary structure prediction of BFV in PRISPRED prediction server, displaying the β-strand, α-helix, and coil structure of BFV.
