# Supplementary material for: Immunoinformatic Design of a Multivalent Peptide Vaccine Against Mucormycosis: Targeting FTR1 Protein of Major Causative Fungi
Source: Front Immunol. 2022 May 26;13:863234. doi: 10.3389/fimmu.2022.863234 (PMC9204303; doi:10.3389/fimmu.2022.863234)
Supplement: Supplementary file 4 [file Image_4.pdf]

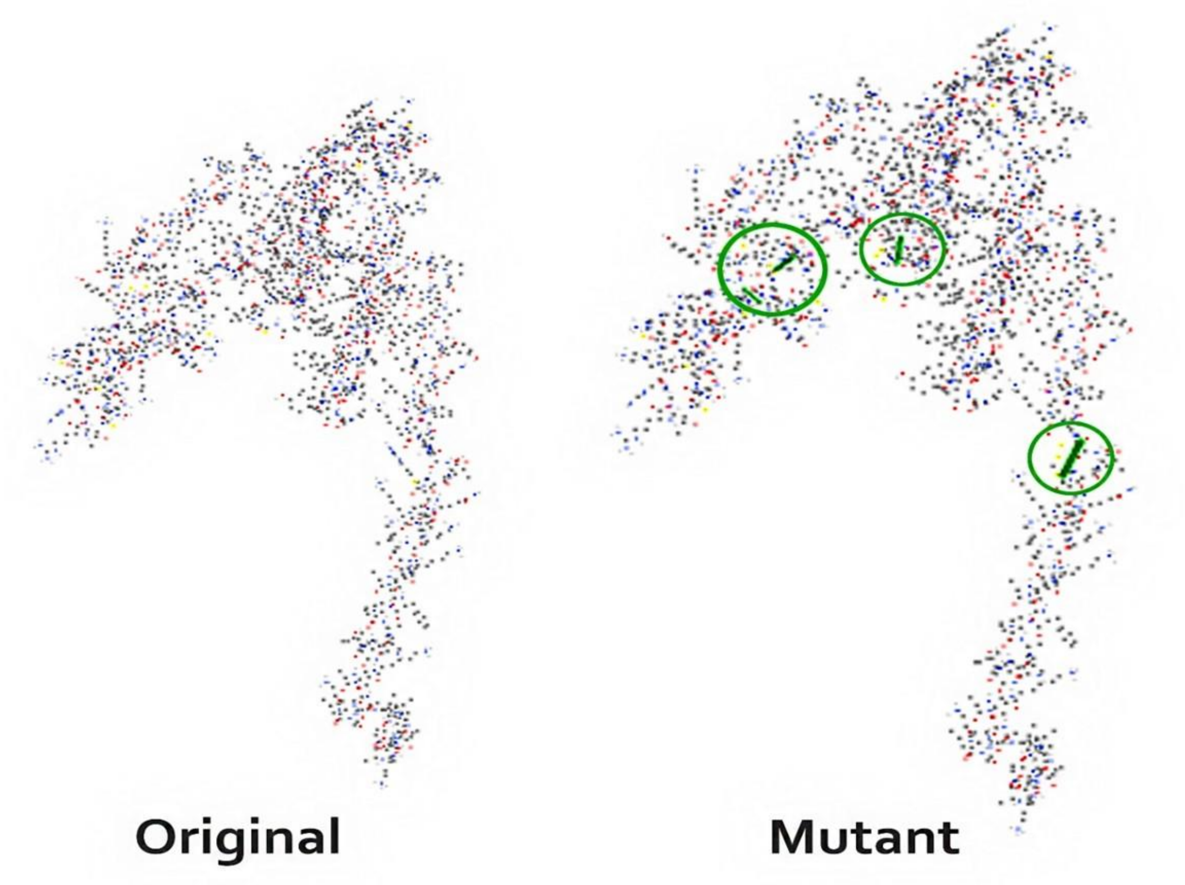

Figure S4. The original BFV is displayed on the left. The mutant BFV after disulfide engineering has been displayed on the right. The disulfide bonds in the mutant vaccine are represented inside the green circles.
