# Supplementary figures and images for: Immunoinformatic Design of a Multivalent Peptide Vaccine Against Mucormycosis: Targeting FTR1 Protein of Major Causative Fungi
Source: Front Immunol. 2022 May 26;13:863234. doi: 10.3389/fimmu.2022.863234 (PMC9204303; doi:10.3389/fimmu.2022.863234)

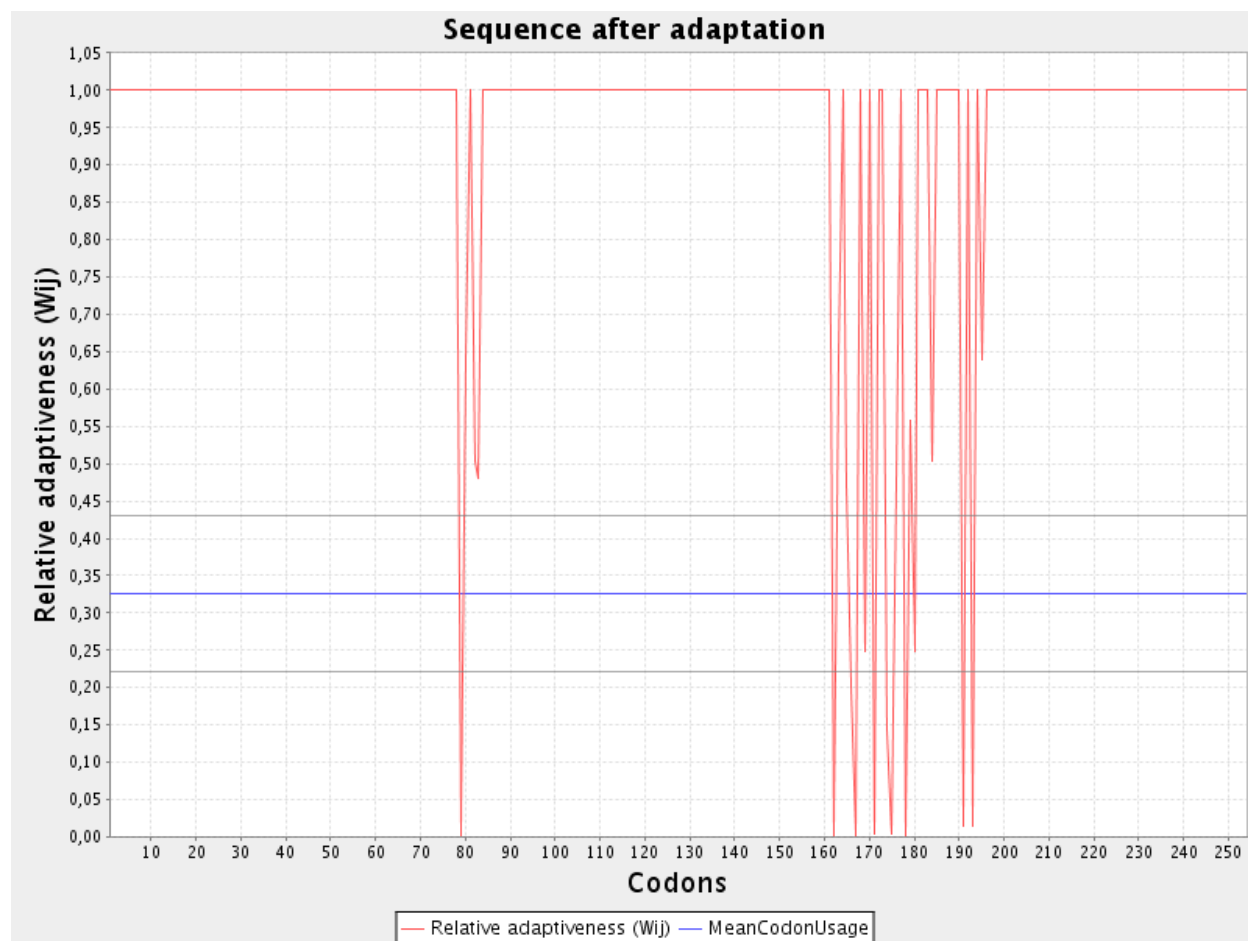

**Figure S5.** Codon adaptation graph of BFV.

Supplement: Supplementary file 5 [file Image_5.pdf]

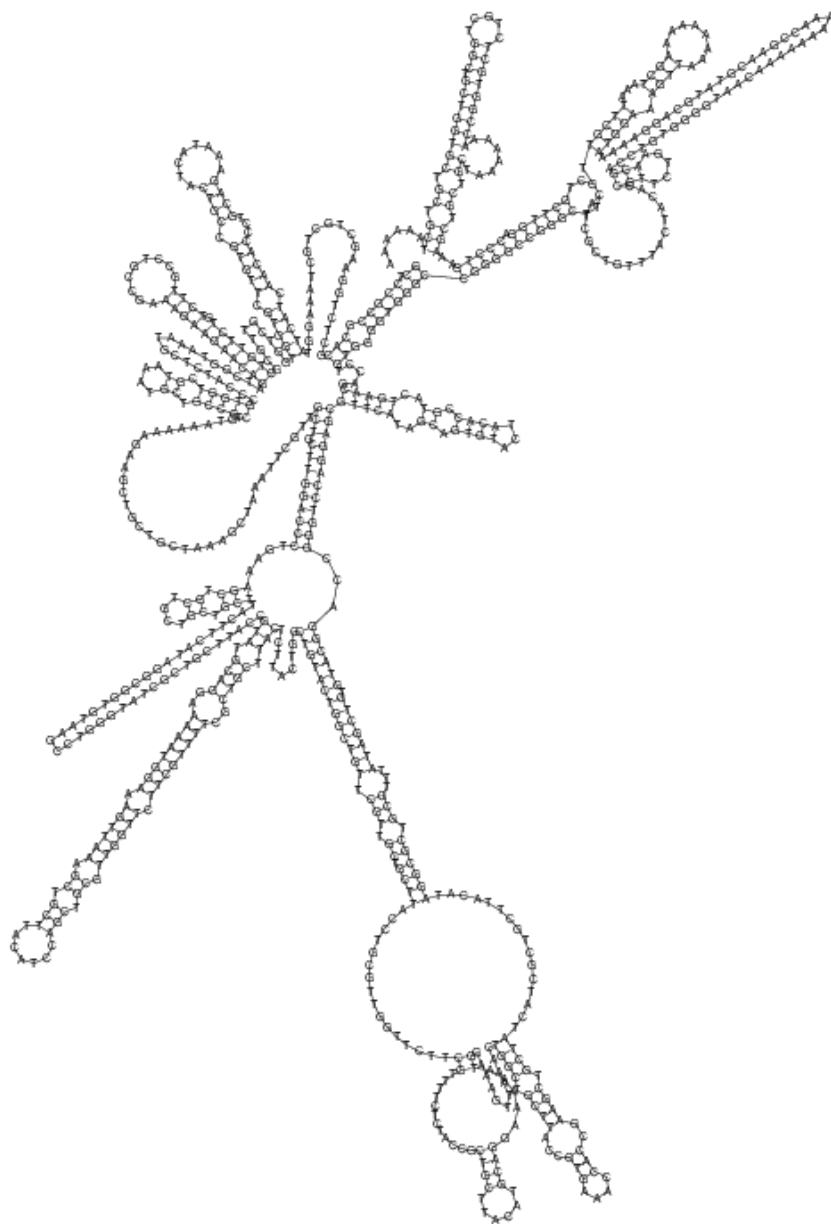

**Figure S6.** The mRNA secondary structure of the BFV was retrieved from the RNAfold server.

Supplement: Supplementary file 6 [file Image_6.pdf]
