# Supplementary material for: Immunoinformatic Design of a Multivalent Peptide Vaccine Against Mucormycosis: Targeting FTR1 Protein of Major Causative Fungi
Source: Front Immunol. 2022 May 26;13:863234. doi: 10.3389/fimmu.2022.863234 (PMC9204303; doi:10.3389/fimmu.2022.863234)
Supplement: Supplementary file 7 [file Table_1.pdf]

Table S1. Selected FTR1 proteins from four different virulent fungus species

Name of the Fungi	GeneBank accession number of the protein	Protein sequence length (aa)
Rhizopus delemar RA 99-880	EIE78766.1	368
Rhizopus azygosporus	RCH93211.1	371
Rhizopus oryzae	AAQ24109.1	368
Rhizopus stolonifer	RCH79546.1	367
Mucor circinelloides 1006PhL	EPB89983.1	372
