# Supplementary material for: Immunoinformatic Design of a Multivalent Peptide Vaccine Against Mucormycosis: Targeting FTR1 Protein of Major Causative Fungi
Source: Front Immunol. 2022 May 26;13:863234. doi: 10.3389/fimmu.2022.863234 (PMC9204303; doi:10.3389/fimmu.2022.863234)
Supplement: Supplementary file 8 [file Table_2.pdf]

Table S2. List of the predicted CTL epitopes of the Ftr1 protein, their predicted scores (>1.00), and their antigenicity, allergenicity, toxicity, and topology analyses. TT; Topology.

HLA Supertype	Epitopes	Scores (>1.000)	Antigenicity (Threshold: 0.5)	Allergenicity	Toxicity	TT
**A1**	GSIISYCLY	1.8699	Non-antigen	Allergen	Non-toxin	Inside
	ATYGSIISY	1.3453	Antigen	Allergen	Non-toxin	Inside
	FVFSTVVLY	1.3408	Non-antigen	Non-allergen	Non-toxin	Outside
	GAAFIAVYY	1.1633	Non-antigen	Non-allergen	Non-toxin	Inside
	NTATYGSII	0.9800	Non-antigen	Allergen	Non-toxin	Inside
	ESDEEANNH	0.9503	Non-antigen	Non-allergen	Non-toxin	Outside
	ISYCLYWLF	0.8936	Antigen	Allergen	Non-toxin	Outside
	RVSTAVWHV	0.7650	Non-antigen	Non-allergen	Non-toxin	Inside
**A2**	KLQKYAFFV	1.3303	Non-antigen	Allergen	Non-toxin	Inside
	FVLPFITVL	1.3184	Non-antigen	Non-allergen	Non-toxin	Outside
	RMFNTESPV	1.2116	Antigen	Allergen	Non-toxin	Inside
	WLFVCCYLV	1.1862	Non-antigen	Allergen	Toxin	Outside
	FIAVYYTVL	1.1308	Non-antigen	Non-allergen	Non-toxin	Inside
	YAFFVLPFI	1.0930	Non-antigen	Allergen	Non-toxin	Outside
	FIGGVSLGI	1.0756	Antigen	Non-allergen	Non-toxin	Outside
	IISYCLYWL	1.0672	Non-antigen	Allergen	Non-toxin	Outside
	FSTVVLYLV	1.0243	Non-antigen	Non-allergen	Non-toxin	Outside
	LMAKGVGYL	1.0108	Non-antigen	Non-allergen	Non-toxin	Outside
	YLVAAGLMA	1.0102	Non-antigen	Non-allergen	Non-toxin	Outside
	RVSTAVWHV	0.9869	Non-antigen	Non-allergen	Non-toxin	Inside
	VLYLVAAGL	0.9266	Non-antigen	Non-allergen	Non-toxin	Outside
	ILFRETTEA	0.9071	Non-antigen	Non-allergen	Non-toxin	Inside
	FLIYRGGSL	0.8688	Non-antigen	Non-allergen	Non-toxin	Outside
	DIWEGVFSL	0.8495	Non-antigen	Non-allergen	Non-toxin	Outside
	AADVISYRV	0.8063	Antigen	Allergen	Non-toxin	Inside
	AILGWNNTA	0.7978	Non-antigen	Allergen	Non-toxin	Inside
	VLREGLEAV	0.7968	Non-antigen	Allergen	Non-toxin	Outside
	VLLSFLKRM	0.7836	Non-antigen	Allergen	Non-toxin	Outside
	AFIAVYYTV	0.7557	Non-antigen	Non-allergen	Non-toxin	Inside
**A3**	TAMGLAMLK	1.4302	Non-antigen	Non-allergen	Non-toxin	Inside
	ATYGSIISY	1.4193	Antigen	Allergen	Non-toxin	Inside
	ISVLLSFLK	1.4022	Antigen	Allergen	Non-toxin	Outside
	LVFSYFKEK	1.3840	Non-antigen	Non-allergen	Non-toxin	Outside
	LVAAGLMAK	1.3794	Non-antigen	Non-allergen	Non-toxin	Outside
	FVFSTVVLY	1.2418	Non-antigen	Non-allergen	Non-toxin	Outside
	SSFKEKLQK	1.2107	Non-antigen	Non-allergen	Non-toxin	Inside
	SVLLSFLKR	1.2107	Non-antigen	Allergen	Non-toxin	Outside
	VLPFITVLR	1.1794	Non-antigen	Non-allergen	Non-toxin	Outside
	AMQKSNSEK	1.1457	Non-antigen	Allergen	Non-toxin	Inside
	RMQEKWKVK	1.1218	Antigen	Non-allergen	Non-toxin	Inside
	GLMAKGVGY	1.0896	Non-antigen	Allergen	Non-toxin	Outside
	GAAFIAVYY	0.9873	Non-antigen	Non-allergen	Non-toxin	Inside
	GVSLGIQGK	0.9678	Antigen	Non-allergen	Non-toxin	Outside

	CLVGFLIYR	0.9560	Non-antigen	Allergen	Non-toxin	Outside
**A24**	KYAFFVLPF	2.0609	Non-antigen	Non-allergen	Non-toxin	Outside
	VYYTVLNDL	1.6596	Non-antigen	Allergen	Non-toxin	Outside
	IQLRWFFVF	1.5956	Antigen	Non-allergen	Non-toxin	Outside
	QYIGNDGEF	1.4374	Antigen	Allergen	Non-toxin	Outside
	VFSTVVLYL	1.4206	Non-antigen	Non-allergen	Non-toxin	Outside
	ISYCLYWLF	1.3863	Antigen	Allergen	Non-toxin	Outside
	LFVCCYLVF	1.3786	Antigen	Allergen	Toxin	Outside
	YYTVLNDLW	1.3709	Non-antigen	Non-allergen	Non-toxin	Outside
	YWLFVCCYL	1.3319	Antigen	Allergen	Toxin	Outside
	AFIAVYYTV	1.2884	Non-antigen	Non-allergen	Non-toxin	Inside
	RWFFVFSTV	1.2198	Non-antigen	Non-allergen	Non-toxin	Outside
	CYLVFSYFK	1.1582	Non-antigen	Non-allergen	Non-toxin	Inside
	NVPIFFILF	1.1367	Non-antigen	Non-allergen	Non-toxin	Outside
	SYCLYWLFV	1.1361	Antigen	Non-allergen	Non-toxin	Outside
	LYLVAAGLM	1.0834	Non-antigen	Non-allergen	Non-toxin	Outside
	SYRVSTAVW	1.0233	Non-antigen	Allergen	Non-toxin	Inside
	IIISVLLSF	1.0060	Non-antigen	Non-allergen	Non-toxin	Outside
	IWEGVFSLV	0.9840	Non-antigen	Non-allergen	Non-toxin	Outside
	VYKRLRNQV	0.9560	Non-antigen	Non-allergen	Non-toxin	Inside
	LYWLFVCCY	0.9076	Antigen	Allergen	Toxin	Outside
	LFNVPIFFI	0.9024	Non-antigen	Non-allergen	Non-toxin	Outside
	FFVFSTVVL	0.8010	Non-antigen	Non-allergen	Non-toxin	Outside
	SLIQLRWFF	0.7750	Antigen	Allergen	Non-toxin	Outside
**A26**	FVFSTVVLY	1.9361	Non-antigen	Non-allergen	Non-toxin	Outside
	ATYGSIISY	1.7985	Antigen	Allergen	Non-toxin	Inside
	EALENAKQY	1.4207	Non-antigen	Allergen	Non-toxin	Inside
	DTSGGWQIF	1.3005	Antigen	Allergen	Non-toxin	Outside
	DIWEGVFSL	1.1927	Non-antigen	Non-allergen	Non-toxin	Outside
	SLIQLRWFF	1.1775	Antigen	Allergen	Non-toxin	Outside
	IIISVLLSF	1.1399	Non-antigen	Non-allergen	Non-toxin	Outside
	SFKEKLQKY	1.1253	Antigen	Non-allergen	Non-toxin	Inside
	DLFNVPIFF	1.0454	Non-antigen	Non-allergen	Non-toxin	Outside
	NVPIFFILF	0.9390	Non-antigen	Non-allergen	Non-toxin	Outside
	EAVVFIGGV	0.8733	Non-antigen	Non-allergen	Non-toxin	Outside
	QIFNAILGW	0.8628	Non-antigen	Allergen	Non-toxin	Outside
	EAADVISYR	0.8013	Antigen	Allergen	Non-toxin	Inside
	GSIISYCLY	0.7574	Non-antigen	Allergen	Non-toxin	Inside
**B7**	VAVIMITAM	0.8951	Non-antigen	Non-allergen	Non-toxin	Inside
	MITAMGLAM	0.8564	Non-antigen	Non-allergen	Non-toxin	Inside
	FLIYRGGSL	0.8533	Non-antigen	Non-allergen	Non-toxin	Outside
	VVFIGGVSL	0.8064	Non-antigen	Allergen	Non-toxin	Outside
**B8**	YFKEKRAAI	2.1600	Antigen	Allergen	Non-toxin	Inside
	FIAVYYTVL	1.8805	Non-antigen	Non-allergen	Non-toxin	Inside
	FLIYRGGSL	1.6357	Non-antigen	Non-allergen	Non-toxin	Outside
	KEKLQKYAF	1.4184	Non-antigen	Non-allergen	Non-toxin	Inside
	FITVLREGL	1.2857	Non-antigen	Non-allergen	Non-toxin	Outside
	YKRLRNQVW	1.2062	Non-antigen	Non-allergen	Non-toxin	Inside
	FVLPFITVL	1.1879	Non-antigen	Non-allergen	Non-toxin	Outside
	VYKRLRNQV	1.0850	Non-antigen	Non-allergen	Non-toxin	Inside
	ISYRVSTAV	1.0201	Non-antigen	Non-allergen	Non-toxin	Inside

	FLKRMFNTE	0.9901	Antigen	Non-allergen	Non-toxin	Inside
	VLYLVAAGL	0.9477	Non-antigen	Non-allergen	Non-toxin	Outside
	WKVKLAKAM	0.9443	Antigen	Allergen	Non-toxin	Inside
	FFVFSTVVL	0.8760	Non-antigen	Non-allergen	Non-toxin	Outside
	LQKYAFFVL	0.8636	Non-antigen	Non-allergen	Non-toxin	Outside
	IQLRWFFVF	0.8556	Antigen	Allergen	Non-toxin	Outside
	EAAIIISVL	0.8430	Non-antigen	Non-allergen	Non-toxin	Outside
	VLREGLEAV	0.7915	Non-antigen	Allergen	Non-toxin	Outside
	FFILFRETT	0.7817	Non-antigen	Non-allergen	Non-toxin	Outside
	MITAMGLAM	0.7605	Non-antigen	Non-allergen	Non-toxin	Inside
**B27**	YRGGSLIQL	1.4697	Non-antigen	Allergen	Non-toxin	Outside
	KRLRNQVWI	1.4279	Non-antigen	Non-allergen	Non-toxin	Inside
	YRVSTAVWH	1.1015	Non-antigen	Non-allergen	Non-toxin	Inside
	LRWFFVFST	1.0815	Antigen	Non-allergen	Non-toxin	Outside
	KRAAIRKAE	0.9498	Non-antigen	Allergen	Non-toxin	Inside
	IQLRWFFVF	0.9424	Antigen	Allergen	Non-toxin	Outside
	EKWKVKLAK	0.8732	Antigen	Allergen	Non-toxin	Inside
	FRETTEAAI	0.8483	Non-antigen	Non-allergen	Non-toxin	Inside
	KRMFNTESP	0.8433	Antigen	Allergen	Non-toxin	Inside
	LREGLEAVV	0.8036	Non-antigen	Non-allergen	Non-toxin	Outside
**B39**	FFVFSTVVL	1.8345	Non-antigen	Non-allergen	Non-toxin	Outside
	FIAVYYTVL	1.7774	Non-antigen	Non-allergen	Non-toxin	Inside
	YRGGSLIQL	1.7750	Non-antigen	Allergen	Non-toxin	Outside
	FNVPIFFIL	1.5723	Non-antigen	Non-allergen	Non-toxin	Outside
	MQEKWKVKL	1.5459	Antigen	Non-allergen	Non-toxin	Inside
	IQLRWFFVF	1.3264	Antigen	Allergen	Non-toxin	Outside
	FVLPFITVL	1.0401	Non-antigen	Non-allergen	Non-toxin	Outside
	YGSIISYCL	1.0191	Antigen	Allergen	Non-toxin	Inside
	EAAIIISVL	1.0154	Non-antigen	Non-allergen	Non-toxin	Outside
	LQKYAFFVL	1.0094	Non-antigen	Non-allergen	Non-toxin	Outside
	EWDDGDEAL	0.9879	Non-antigen	Non-allergen	Non-toxin	Outside
	FRETTEAAI	0.9778	Non-antigen	Non-allergen	Non-toxin	Inside
	SQDLFNVPI	0.8782	Non-antigen	Non-allergen	Non-toxin	Outside
	STAVWHVSW	0.8178	Non-antigen	Allergen	Non-toxin	Outside
**B44**	REGLEAVVF	1.3730	Non-antigen	Non-allergen	Non-toxin	Outside
	TESPVYKRL	1.3554	Non-antigen	Non-allergen	Non-toxin	Inside
	TEAAIIISV	1.1876	Antigen	Non-allergen	Non-toxin	Inside
	RETTEAAII	1.0679	Antigen	Non-allergen	Non-toxin	Inside
	LEQNAWNQV	1.0248	Non-antigen	Non-allergen	Non-toxin	Inside
	KEKLQKYAF	0.9491	Non-antigen	Non-allergen	Non-toxin	Inside
	GEAADVISY	0.9133	Antigen	Allergen	Non-toxin	Outside
	LQKYAFFVL	0.8019	Non-antigen	Non-allergen	Non-toxin	Outside
	FNVPIFFIL	0.7725	Non-antigen	Non-allergen	Non-toxin	Outside
**B58**	STAVWHVSW	2.0157	Non-antigen	Allergen	Non-toxin	Outside
	ISYCLYWLF	1.7134	Antigen	Allergen	Non-toxin	Outside
	KSNSEKSSF	1.7028	Non-antigen	Allergen	Non-toxin	Inside
	KTERMQEKW	1.6697	Non-antigen	Non-allergen	Non-toxin	Inside
	SIISYCLYW	1.5907	Non-antigen	Non-allergen	Non-toxin	Inside
	KSIPIAAIM	1.5855	Non-antigen	Non-allergen	Non-toxin	Inside
	IIISVLLSF	1.5511	Non-antigen	Non-allergen	Non-toxin	Outside
	GGSLIQLRW	1.5108	Antigen	Non-allergen	Non-toxin	Outside

	QIFNAILGW	1.4824	Non-antigen	Allergen	Non-toxin	Outside
	VGYLEQNAW	1.3677	Non-antigen	Allergen	Non-toxin	Outside
	IGAAFIAVY	1.2060	Antigen	Non-allergen	Non-toxin	Outside
	GSIISYCLY	1.1460	Non-antigen	Allergen	Non-toxin	Inside
	GAAFIAVYY	1.0624	Non-antigen	Non-allergen	Non-toxin	Inside
	KYAFFVLPF	1.0206	Non-antigen	Non-allergen	Non-toxin	Outside
	ISYRVSTAV	0.9702	Non-antigen	Non-allergen	Non-toxin	Inside
	IQLRWFFVF	0.9691	Antigen	Allergen	Non-toxin	Outside
	ATYGSIISY	0.9351	Antigen	Allergen	Non-toxin	Inside
	RVSTAVWHV	0.8337	Non-antigen	Non-allergen	Non-toxin	Inside
	SLIQLRWFF	0.8047	Antigen	Allergen	Non-toxin	Outside
**B62**	ATYGSIISY	1.4651	Antigen	Allergen	Non-toxin	Inside
	IQLRWFFVF	1.3738	Antigen	Allergen	Non-toxin	Outside
	GLMAKGVGY	1.3670	Non-antigen	Allergen	Non-toxin	Outside
	FVFSTVVLY	1.3385	Non-antigen	Non-allergen	Non-toxin	Outside
	IGAAFIAVY	1.2503	Antigen	Non-allergen	Non-toxin	Outside
	MFNTESPVY	1.1278	Non-antigen	Allergen	Non-toxin	Outside
	IIISVLLSF	1.1266	Non-antigen	Non-allergen	Non-toxin	Outside
	VVFIGGVSL	1.1016	Non-antigen	Allergen	Non-toxin	Outside
	RMFNTESPV	1.0976	Antigen	Allergen	Non-toxin	Inside
	LMAKGVGYL	1.0888	Non-antigen	Non-allergen	Non-toxin	Outside
	FLIYRGGSL	1.0451	Non-antigen	Non-allergen	Non-toxin	Outside
	LQKYAFFVL	1.0022	Non-antigen	Non-allergen	Non-toxin	Outside
	LGWNNTATY	1.0006	Non-antigen	Allergen	Non-toxin	Inside
	GAAFIAVYY	0.9786	Non-antigen	Non-allergen	Non-toxin	Inside
	GEAADVISY	0.9592	Antigen	Allergen	Non-toxin	Outside
	EQNAWNQVI	0.9432	Non-antigen	Non-allergen	Non-toxin	Inside
	KSNSEKSSF	0.9415	Non-antigen	Allergen	Non-toxin	Inside
	GSIISYCLY	0.9253	Non-antigen	Allergen	Non-toxin	Inside
	KYAFFVLPF	0.9154	Non-antigen	Non-allergen	Non-toxin	Outside
	FVLPFITVL	0.8924	Non-antigen	Non-allergen	Non-toxin	Outside
	MITAMGLAM	0.8891	Non-antigen	Non-allergen	Non-toxin	Inside
	SLIQLRWFF	0.8519	Antigen	Allergen	Non-toxin	Outside
	LLSFLKRMF	0.8487	Non-antigen	Non-allergen	Non-toxin	Outside
	SFKEKLQKY	0.8387	Antigen	Non-allergen	Non-toxin	Inside
	WIGGAAGLF	0.7726	Antigen	Non-allergen	Non-toxin	Outside
	KSIPIAAIM	0.7544	Non-antigen	Non-allergen	Non-toxin	Inside
