# Supplementary material for: Immunoinformatic Design of a Multivalent Peptide Vaccine Against Mucormycosis: Targeting FTR1 Protein of Major Causative Fungi
Source: Front Immunol. 2022 May 26;13:863234. doi: 10.3389/fimmu.2022.863234 (PMC9204303; doi:10.3389/fimmu.2022.863234)
Supplement: Supplementary file 9 [file Table_3.pdf]

Table S3. List of the predicted HTL epitopes of the Ftr1 protein, their percentile rank (<1.00) and their antigenicity, allergenicity, toxicity, and topology analyses.

Allele	Epitopes	Percentile Rank <1.00	Start	End	Antigenicity (Threshold: 0.5)	Allergenicity	Toxicity	Topology
HLA-DPA1*01:03/DPB1*04:01	KLQKYAFFVLPFITV	0.12	137	151	Non-antigen	Non-allergen	Non-toxin	Outside
HLA-DPA1*01:03/DPB1*04:01	EKLQKYAFFVLPFIT	0.17	136	150	Non-antigen	Non-allergen	Non-toxin	Outside
HLA-DPA1*01:03/DPB1*04:01	LQKYAFFVLPFITVL	0.18	138	152	Non-antigen	Non-allergen	Non-toxin	Outside
HLA-DPA1*01:03/DPB1*02:01	LFVCCYLVFSYFKEK	0.19	296	310	Non-antigen	Allergen	Toxin	Outside
HLA-DPA1*01:03/DPB1*02:01	FVCCYLVFSYFKEKR	0.2	297	311	Antigen	Allergen	Toxin	Inside
HLA-DPA1*01:03/DPB1*04:01	KEKLQKYAFFVLPFI	0.21	135	149	Non-antigen	Non-allergen	Non-toxin	Inside
HLA-DPA1*01:03/DPB1*04:01	VCCYLVFSYFKEKRA	0.21	298	312	Antigen	Non-allergen	Toxin	Inside
HLA-DPA1*01:03/DPB1*04:01	QKYAFFVLPFITVLR	0.23	139	153	Non-antigen	Non-allergen	Non-toxin	Outside
HLA-DRB1*01:01	FSTVVLYLVAAGLMA	0.24	207	221	Non-antigen	Non-allergen	Non-toxin	Outside
HLA-DPA1*02:01/DPB1*01:01	LRWFFVFSTVVLYLV	0.24	201	215	Non-antigen	Non-allergen	Non-toxin	Outside
HLA-DPA1*02:01/DPB1*01:01	RWFFVFSTVVLYLVA	0.24	202	216	Non-antigen	Non-allergen	Non-toxin	Outside
HLA-DRB1*01:01	STVVLYLVAAGLMAK	0.24	208	222	Non-antigen	Non-allergen	Non-toxin	Outside
HLA-DRB1*01:01	TVVLYLVAAGLMAKG	0.24	209	223	Non-antigen	Non-allergen	Non-toxin	Outside
HLA-DPA1*01:03/DPB1*04:01	CCYLVFSYFKEKRAA	0.25	299	313	Antigen	Non-allergen	Toxin	Inside
HLA-DPA1*01:03/DPB1*04:01	FVCCYLVFSYFKEKR	0.25	297	311	Antigen	Allergen	Toxin	Inside
HLA-DPA1*02:01/DPB1*14:01	VISYRVSTAVWHVSW	0.25	243	257	Non-antigen	Non-allergen	Non-toxin	Inside

HLA-DPA1*02:01/DPB1*01:01	QLRWFFVFSTVVLYL	0.26	200	214	Non-antigen	Non-allergen	Non-toxin	Outside
HLA-DRB5*01:01	VFSYFKEKRAAIRKA	0.26	303	317	Non-antigen	Non-allergen	Non-toxin	Inside
HLA-DPA1*01:03/DPB1*04:01	FKEKLQKYAFFVLPF	0.28	134	148	Non-antigen	Non-allergen	Non-toxin	Inside
HLA-DPA1*03:01/DPB1*04:02	KYAFFVLPFITVLRE	0.28	140	154	Non-antigen	Non-allergen	Non-toxin	Outside
HLA-DPA1*01:03/DPB1*04:01	LFVCCYLVFSYFKEK	0.28	296	310	Non-antigen	Allergen	Toxin	Outside
HLA-DPA1*03:01/DPB1*04:02	QKYAFFVLPFITVLR	0.28	139	153	Non-antigen	Non-allergen	Non-toxin	Outside
HLA-DPA1*03:01/DPB1*04:02	LQKYAFFVLPFITVL	0.29	138	152	Non-antigen	Non-allergen	Non-toxin	Outside
HLA-DPA1*03:01/DPB1*04:02	RWFFVFSTVVLYLVA	0.29	202	216	Non-antigen	Non-allergen	Non-toxin	Outside
HLA-DPA1*02:01/DPB1*01:01	WFFVFSTVVLYLVAA	0.29	203	217	Non-antigen	Non-allergen	Non-toxin	Outside
HLA-DPA1*01:03/DPB1*02:01	WLFVCCYLVFSYFKE	0.29	295	309	Non-antigen	Allergen	Toxin	Outside
HLA-DPA1*03:01/DPB1*04:02	YAFFVLPFITVLREG	0.29	141	155	Non-antigen	Non-allergen	Non-toxin	Outside
HLA-DPA1*02:01/DPB1*01:01	FFVFSTVVLYLVAAG	0.3	204	218	Non-antigen	Non-allergen	Non-toxin	Outside
HLA-DPA1*03:01/DPB1*04:02	LRWFFVFSTVVLYLV	0.31	201	215	Non-antigen	Non-allergen	Non-toxin	Outside
HLA-DRB5*01:01	FSYFKEKRAAIRKAE	0.32	304	318	Non-antigen	Non-allergen	Non-toxin	Inside

HLA-DPA1*01:03/DPB1*02:01	LQKYAFFVLPFITVL	0.32	138	152	Non-antigen	Non-allergen	Non-toxin	Outside
HLA-DPA1*02:01/DPB1*14:01	ISYRVSTAVWHVSWG	0.33	244	258	Antigen	Non-allergen	Non-toxin	Inside
HLA-DPA1*03:01/DPB1*04:02	WFFVFSTVVLYLVAA	0.34	203	217	Non-antigen	Non-allergen	Non-toxin	Outside
HLA-DPA1*01:03/DPB1*04:01	CYLVFSYFKEKRAAI	0.35	300	314	Antigen	Non-allergen	Non-toxin	Inside
HLA-DRB5*01:01	EKWKVKLAKAMQKSN	0.35	114	128	Antigen	Allergen	Non-toxin	Inside
HLA-DRB5*01:01	KWKVKLAKAMQKSNS	0.35	115	129	Antigen	Allergen	Non-toxin	Inside
HLA-DPA1*01:03/DPB1*04:01	KYAFFVLPFITVLRE	0.35	140	154	Non-antigen	Non-allergen	Non-toxin	Outside
HLA-DRB5*01:01	MQEKWKVKLAKAMQK	0.35	112	126	Antigen	Allergen	Non-toxin	Inside
HLA-DRB5*01:01	QEKWKVKLAKAMQKS	0.35	113	127	Antigen	Allergen	Non-toxin	Inside
HLA-DPA1*03:01/DPB1*04:02	FFVFSTVVLYLVAAG	0.36	204	218	Non-antigen	Non-allergen	Non-toxin	Outside
HLA-DPA1*01:03/DPB1*02:01	CCYLVFSYFKEKRAA	0.37	299	313	Antigen	Non-allergen	Toxin	Inside
HLA-DRB1*15:01	GCLVGFLIYRGGSLI	0.37	185	199	Non-antigen	Non-allergen	Non-toxin	Outside
HLA-DRB1*15:01	LVGFLIYRGGSLIQL	0.37	187	201	Non-antigen	Non-allergen	Non-toxin	Outside
HLA-DRB5*01:01	WKVKLAKAMQKSNSE	0.37	116	130	Antigen	Allergen	Non-toxin	Inside
HLA-DPA1*03:01/DPB1*04:02	KLQKYAFFVLPFITV	0.39	137	151	Non-antigen	Non-allergen	Non-toxin	Outside
HLA-DRB5*01:01	SYFKEKRAAIRKAEA	0.39	305	319	Non-antigen	Allergen	Non-toxin	Inside
HLA-DPA1*03:01/DPB1*04:02	QLRWFFVFSTVVLYL	0.42	200	214	Non-antigen	Non-allergen	Non-toxin	Outside
HLA-DPA1*02:01/DPB1*14:01	DVISYRVSTAVWHVS	0.44	242	256	Non-antigen	Non-allergen	Non-toxin	Inside
HLA-DRB1*15:01	CLVGFLIYRGGSLIQ	0.46	186	200	Non-antigen	Non-allergen	Non-toxin	Outside
HLA-DPA1*02:01/DPB1*05:01	FKEKLQKYAFFVLPF	0.49	134	148	Non-antigen	Non-allergen	Non-toxin	Inside

HLA-DPA1*02:01/DPB1*05:01	KEKLQKYAFFVLPFI	0.49	135	149	Non-antigen	Non-allergen	Non-toxin	Inside
HLA-DRB5*01:01	LVFSYFKEKRAAIRK	0.49	302	316	Non-antigen	Non-allergen	Non-toxin	Inside
HLA-DRB1*15:01	VGFLIYRGGSLIQLR	0.49	188	202	Non-antigen	Non-allergen	Non-toxin	Outside
HLA-DQA1*01:01/DQB1*05:01	GSIISYCLYWLFVCC	0.51	286	300	Antigen	Allergen	Toxin	Outside
HLA-DPA1*01:03/DPB1*04:01	WLFVCCYLVFSYFKE	0.52	295	309	Non-antigen	Allergen	Toxin	Outside
HLA-DPA1*02:01/DPB1*14:01	KWKVKLAKAMQKSNS	0.54	115	129	Antigen	Allergen	Non-toxin	Inside
HLA-DPA1*01:03/DPB1*02:01	CYLVFSYFKEKRAAI	0.55	300	314	Antigen	Non-allergen	Non-toxin	Inside
HLA-DRB1*01:01	VFSTVVLYLVAAGLM	0.56	206	220	Non-antigen	Non-allergen	Non-toxin	Outside
HLA-DRB1*01:01	VVLYLVAAGLMAKGV	0.56	210	224	Non-antigen	Non-allergen	Non-toxin	Outside
HLA-DPA1*02:01/DPB1*01:01	AAFIAVYYTVLNDLW	0.57	66	80	Non-antigen	Non-allergen	Non-toxin	Inside
HLA-DPA1*02:01/DPB1*01:01	GAAFIAVYYTVLNDL	0.57	65	79	Non-antigen	Non-allergen	Non-toxin	Outside
HLA-DPA1*02:01/DPB1*01:01	IGAAFIAVYYTVLND	0.57	64	78	Non-antigen	Non-allergen	Non-toxin	Outside
HLA-DPA1*03:01/DPB1*04:02	AFFVLPFITVLREGL	0.6	142	156	Non-antigen	Non-allergen	Non-toxin	Outside
HLA-DPA1*02:01/DPB1*14:01	EKWKVKLAKAMQKSN	0.61	114	128	Antigen	Allergen	Non-toxin	Inside
HLA-DPA1*01:03/DPB1*04:01	QDLFNVPIFFILFRE	0.64	3	17	Non-antigen	Non-allergen	Non-toxin	Outside
HLA-DPA1*02:01/DPB1*14:01	QEKWKVKLAKAMQKS	0.64	113	127	Antigen	Allergen	Non-toxin	Inside
HLA-DPA1*02:01/DPB1*01:01	AFIAVYYTVLNDLWG	0.66	67	81	Antigen	Non-allergen	Non-toxin	Outside
HLA-DRB1*01:01	FVFSTVVLYLVAAGL	0.67	205	219	Non-antigen	Non-allergen	Non-toxin	Outside
HLA-DPA1*02:01/DPB1*01:01	QKYAFFVLPFITVLR	0.67	139	153	Non-antigen	Non-allergen	Non-toxin	Outside
HLA-DRB1*01:01	VLYLVAAGLMAKGVG	0.67	211	225	Non-antigen	Non-allergen	Non-toxin	Outside

HLA-DQA1*05:01/DQB1*02:01	AAFIAVYYTVLNDLW	0.68	66	80	Non-antigen	Non-allergen	Non-toxin	Inside
HLA-DPA1*01:03/DPB1*02:01	DLFNVPIFFILFRET	0.69	4	18	Non-antigen	Non-allergen	Non-toxin	Outside
HLA-DPA1*02:01/DPB1*05:01	EKLQKYAFFVLPFIT	0.69	136	150	Non-antigen	Non-allergen	Non-toxin	Outside
HLA-DPA1*03:01/DPB1*04:02	GAAFIAVYYTVLNDL	0.7	65	79	Non-antigen	Non-allergen	Non-toxin	Outside
HLA-DPA1*02:01/DPB1*01:01	KYAFFVLPFITVLRE	0.71	140	154	Non-antigen	Non-allergen	Non-toxin	Outside
HLA-DPA1*02:01/DPB1*01:01	LQKYAFFVLPFITVL	0.72	138	152	Non-antigen	Non-allergen	Non-toxin	Outside
HLA-DPA1*01:03/DPB1*04:01	IQLRWFFVFSTVVLY	0.73	199	213	Non-antigen	Non-allergen	Non-toxin	Outside
HLA-DPA1*02:01/DPB1*14:01	SYRVSTAVWHVSWGD	0.73	245	259	Non-antigen	Allergen	Non-toxin	Inside
HLA-DPA1*02:01/DPB1*01:01	EKLQKYAFFVLPFIT	0.74	136	150	Non-antigen	Non-allergen	Non-toxin	Outside
HLA-DPA1*02:01/DPB1*01:01	KEKLQKYAFFVLPFI	0.74	135	149	Non-antigen	Non-allergen	Non-toxin	Inside
HLA-DPA1*02:01/DPB1*01:01	KLQKYAFFVLPFITV	0.74	137	151	Non-antigen	Non-allergen	Non-toxin	Outside
HLA-DPA1*03:01/DPB1*04:02	FFVLPFITVLREGLE	0.75	143	157	Non-antigen	Non-allergen	Non-toxin	Outside
HLA-DRB4*01:01	LVAVIMITAMGLAML	0.75	93	107	Non-antigen	Non-allergen	Non-toxin	Outside
HLA-DRB1*09:01	QEKWKVKLAKAMQKS	0.75	113	127	Antigen	Allergen	Non-toxin	Inside
HLA-DRB5*01:01	YFKEKRAAIRKAEAG	0.75	306	320	Antigen	Non-allergen	Non-toxin	Inside
HLA-DPA1*01:03/DPB1*02:01	GSLIQLRWFFVFSTV	0.77	196	210	Non-antigen	Non-allergen	Non-toxin	Outside
HLA-DPA1*02:01/DPB1*01:01	FIAVYYTVLNDLWGN	0.78	68	82	Antigen	Non-allergen	Non-toxin	Outside
HLA-DPA1*02:01/DPB1*05:01	KLQKYAFFVLPFITV	0.81	137	151	Non-antigen	Non-allergen	Non-toxin	Outside
HLA-DPA1*01:03/DPB1*02:01	SLIQLRWFFVFSTVV	0.82	197	211	Non-antigen	Non-allergen	Non-toxin	Outside

HLA-DPA1*03:01/DPB1*04:02	AAFIAVYYTVLNDLW	0.83	66	80	Non-antigen	Non-allergen	Non-toxin	Inside
HLA-DPA1*02:01/DPB1*01:01	FKEKLQKYAFFVLPF	0.83	134	148	Non-antigen	Non-allergen	Non-toxin	Inside
HLA-DRB1*07:01	DIWEGVFSLVAVIMI	0.84	85	99	Non-antigen	Non-allergen	Non-toxin	Outside
HLA-DRB1*07:01	EGVFSLVAVIMITAM	0.84	88	102	Non-antigen	Non-allergen	Non-toxin	Outside
HLA-DRB1*07:01	GVFSLVAVIMITAMG	0.84	89	103	Non-antigen	Non-allergen	Non-toxin	Outside
HLA-DRB1*07:01	IWEGVFSLVAVIMIT	0.84	86	100	Non-antigen	Non-allergen	Non-toxin	Outside
HLA-DRB4*01:01	VAVIMITAMGLAMLK	0.84	94	108	Non-antigen	Non-allergen	Non-toxin	Inside
HLA-DRB1*07:01	WEGVFSLVAVIMITA	0.84	87	101	Non-antigen	Non-allergen	Non-toxin	Outside
HLA-DPA1*01:03/DPB1*04:01	QLRWFFVFSTVVLYL	0.87	200	214	Non-antigen	Non-allergen	Non-toxin	Outside
HLA-DPA1*02:01/DPB1*01:01	SFKEKLQKYAFFVLP	0.87	133	147	Non-antigen	Non-allergen	Non-toxin	Inside
HLA-DRB1*09:01	EKWKVKLAKAMQKSN	0.88	114	128	Antigen	Allergen	Non-toxin	Inside
HLA-DQA1*01:01/DQB1*05:01	IISYCLYWLFVCCYL	0.9	288	302	Antigen	Allergen	Toxin	Outside
HLA-DPA1*02:01/DPB1*05:01	SFKEKLQKYAFFVLP	0.9	133	147	Non-antigen	Non-allergen	Non-toxin	Inside
HLA-DPA1*01:03/DPB1*02:01	EKLQKYAFFVLPFIT	0.91	136	150	Non-antigen	Non-allergen	Non-toxin	Outside
HLA-DPA1*01:03/DPB1*02:01	QKYAFFVLPFITVLR	0.91	139	153	Non-antigen	Non-allergen	Non-toxin	Outside
HLA-DRB1*04:05	SVLLSFLKRMFNTES	0.92	26	40	Antigen	Non-allergen	Non-toxin	Outside
HLA-DPA1*02:01/DPB1*01:01	SSFKEKLQKYAFFVL	0.93	132	146	Non-antigen	Non-allergen	Non-toxin	Inside
HLA-DQA1*01:01/DQB1*05:01	YGSIISYCLYWLFVC	0.94	285	299	Non-antigen	Allergen	Non-toxin	Outside
HLA-DPA1*01:03/DPB1*04:01	GAAFIAVYYTVLNDL	0.95	65	79	Non-antigen	Non-allergen	Non-toxin	Outside
HLA-DPA1*01:03/DPB1*04:01	AAFIAVYYTVLNDLW	0.96	66	80	Non-antigen	Non-allergen	Non-toxin	Inside
HLA-DPA1*01:03/DPB1*02:01	KEKLQKYAFFVLPFI	0.97	135	149	Non-antigen	Non-allergen	Non-toxin	Inside

HLA-DRB1*04:05	LLSFLKRMFNTESPV	0.97	28	42	Antigen	Non-allergen	Non-toxin	Outside
HLA-DRB1*04:05	LSFLKRMFNTESPVY	0.98	29	43	Antigen	Allergen	Non-toxin	Outside
HLA-DPA1*02:01/DPB1*01:01	QDLFNVPIFFILFRE	0.98	3	17	Non-antigen	Non-allergen	Non-toxin	Outside
HLA-DPA1*01:03/DPB1*02:01	RGGSLIQLRWFFVFS	0.98	194	208	Antigen	Allergen	Non-toxin	Outside
HLA-DPA1*01:03/DPB1*04:01	DLFNVPIFFILFRET	0.99	4	18	Non-antigen	Non-allergen	Non-toxin	Outside
