# Supplementary material for: Immunoinformatic Design of a Multivalent Peptide Vaccine Against Mucormycosis: Targeting FTR1 Protein of Major Causative Fungi
Source: Front Immunol. 2022 May 26;13:863234. doi: 10.3389/fimmu.2022.863234 (PMC9204303; doi:10.3389/fimmu.2022.863234)
Supplement: Supplementary file 10 [file Table_4.pdf]

Table S4. List of the predicted epitopes of the Ftr1 protein, their surface scores (<1.00), their antigenicity, allergenicity, toxicity and topology analyses.

**Peptide**	**Start**	**End**	**Length**	**Surface score** (Emini's surface threshold = 1.000)	**Antigenicity (Threshold**: 0.5**)**	**Allergenicity**	**Toxicity**	**Topology**
FNTESPVYKRLRNQ	36	49	14	2.376	Non-antigen	Allergen	Non-Toxin	Inside
LNDLWGNS	76	83	8	0.933	Antigen	Allergen	Non-Toxin	Outside
KTERMQEKWKVK	108	119	12	3.077	Antigen	Non-allergen	Non-Toxin	Inside
MQKSNSEKSSFKEKLQKY	124	141	18	2.711	Non-antigen	Allergen	Non-Toxin	Inside
LGIQGKSI	167	174	8	0.802	Antigen	Non-allergen	Non-Toxin	Outside
