# Supplementary material for: Immunoinformatic Design of a Multivalent Peptide Vaccine Against Mucormycosis: Targeting FTR1 Protein of Major Causative Fungi
Source: Front Immunol. 2022 May 26;13:863234. doi: 10.3389/fimmu.2022.863234 (PMC9204303; doi:10.3389/fimmu.2022.863234)
Supplement: Supplementary file 11 [file Table_5.pdf]

Table S5. Antigenicity, allergenicity, and biophysical analyses of BFV.

Name of the protein	AN (threshold 0.5)	AG	pI	Number of positively charged amino acids	Number of negatively charged amino acids	Extinction coefficients (in M^{-1} cm^{-1})	Estimated half-life	II	AI	GRAVY
BFV	Antigenic	Non-allergenic	9.97	42	12	80705	4.4 hours (mammalian reticulocytes, in vitro), >20 hours in yeast, in vivo and >10 h in *E. coli* cell culture system	Stable (25.15)	84.41	0.111
