# Supplementary material for: Immunoinformatic Design of a Multivalent Peptide Vaccine Against Mucormycosis: Targeting FTR1 Protein of Major Causative Fungi
Source: Front Immunol. 2022 May 26;13:863234. doi: 10.3389/fimmu.2022.863234 (PMC9204303; doi:10.3389/fimmu.2022.863234)
Supplement: Supplementary file 12 [file Table_6.pdf]

Table S6. The proteasomal cleavage analysis of BFV using MHCII-NP server.

Start	End	Length	Peptide	N motif	C motif	Score	Percentile Rank
162	175	14	IGAAFIAVYGPGPG	YIG	PGA	0.70633	0.0
35	50	16	LPKEEQIGKCSTRGRK	CLP	RKC	0.6686	0.04
191	205	15	GPGPGFIAVYYTVLN	GGP	LND	0.55423	0.08
35	48	14	LPKEEQIGKCSTRG	CLP	RGR	0.53698	0.12
35	49	15	LPKEEQIGKCSTRGR	CLP	GRK	0.51019	0.15
41	55	15	IGKCSTRGRKCCRRK	QIG	RKK	0.50695	0.19
171	186	16	GPGPGAFIAVYYTVLN	YGP	LND	0.50134	0.23
26	40	15	GGRCAVLSCLPKEEQ	RGG	EQI	0.44459	0.27
171	185	15	GPGPGAFIAVYYTVL	YGP	VLN	0.44337	0.31
191	206	16	GPGPGFIAVYYTVLND	GGP	NDL	0.42422	0.35
161	175	15	YIGAAFIAVYGPGPG	AYI	PGA	0.41657	0.39
41	54	14	IGKCSTRGRKCCRR	QIG	RRK	0.41448	0.43
26	39	14	GGRCAVLSCLPKEE	RGG	EEQ	0.41319	0.46
191	204	14	GPGPGFIAVYYTVL	GGP	VLN	0.40404	0.5
75	90	16	AAYFIGGVSLGIAAYR	AAA	YRM	0.38225	0.54
41	57	17	IGKCSTRGRKCCRRKKE	QIG	KEA	0.3795	0.58
41	56	16	IGKCSTRGRKCCRRKK	QIG	KKE	0.36558	0.62
24	37	14	VRGGRCAVLSCLPK	RVR	PKE	0.35157	0.66
47	61	15	RGRKCCRRKKEAAAK	TRG	AKA	0.34946	0.7
10	25	16	AKGIINTLQKYYCRVR	AAK	VRG	0.34752	0.74
149	163	15	YRETTEAAIIAAYIG	AYR	IGA	0.34348	0.77
26	42	17	GGRCAVLSCLPKEEQIG	RGG	IGK	0.33844	0.81
182	195	14	YTVLNDLWGGPGPG	YYT	PGF	0.32957	0.85
89	103	15	YRMQEKWKVKAAYIQ	AYR	IQL	0.32257	0.89
191	207	17	GPGPGFIAVYYTVLNDL	GGP	DLW	0.3178	0.93
46	61	16	TRGRKCCRRKKEAAAK	STR	AKA	0.3107	0.97
48	61	14	GRKCCRRKKEAAAK	RGR	AKA	0.30308	1.01
214	228	15	TERMQEKWKVKKKAK	KTE	AKF	0.30105	1.04
171	183	13	GPGPGAFIAVYYT	YGP	YTV	0.30091	1.08
35	47	13	LPKEEQIGKCSTR	CLP	TRG	0.29854	1.12
211	224	14	KKKTERMQEKWKVK	NKK	VKK	0.29294	1.16
60	74	15	AKAKFVAAWTLKAAA	AAK	AAA	0.28923	1.2
12	25	14	GIINTLQKYYCRVR	KGI	VRG	0.28731	1.24
171	187	17	GPGPGAFIAVYYTVLND	YGP	NDL	0.28602	1.28
42	57	16	GKCSTRGRKCCRRKKE	IGK	KEA	0.28395	1.32
6	19	14	LEAAAKGIINTLQK	ALE	QKY	0.27779	1.35
85	98	14	GIAAYRMQEKWKVK	LGI	VKA	0.27763	1.39
237	251	15	AAAKKTGALLAAGAA	KAA	AAA	0.27721	1.43
202	216	15	TVLNDLWGNKKKTER	YTV	ERM	0.27704	1.47
56	71	16	KEAAAKAKFVAAWTLK	KKE	LKA	0.27631	1.51
133	147	15	STAAYMQEKWKVKLA	FST	LAA	0.27485	1.55
211	226	16	KKKTERMQEKWKVKKK	NKK	KKA	0.27481	1.59
203	216	14	VLNDLWGNKKKTER	TVL	ERM	0.27295	1.62
133	145	13	STAAYMQEKWKVK	FST	VKL	0.27245	1.66
60	73	14	AKAKFVAAWTLKAA	AAK	AAA	0.26357	1.7

49	63	15	RKCCRRKKEAAAKAK	GRK	AKF	0.26315	1.74
154	168	15	EAAIIAAYIGAAFIA	TEA	IAV	0.263	1.78
77	90	14	YFIGGVSLGIAAYR	AYF	YRM	0.26234	1.82
59	73	15	AAKAKFVAAWTLKAA	AAA	AAA	0.26225	1.86
60	75	16	AKAKFVAAWTLKAAAA	AAK	AAA	0.26162	1.9
167	181	15	IAVYGPGPGAFIAVY	FIA	VYY	0.2616	1.93
209	224	16	GNKKKTERMQEKWKVK	WGN	VKK	0.26111	1.97
82	96	15	VSLGIAAYRMQEKWK	GVS	WKV	0.26027	2.01
23	37	15	RVRGGRCAVLSCLPK	CRV	PKE	0.25979	2.05
48	63	16	GRKCCRRKKEAAAKAK	RGR	AKF	0.25917	2.09
239	253	15	AKKTGALLAAGAAAK	AAK	AKK	0.25881	2.13
74	90	17	AAAYFIGGVSLGIAAYR	AAA	YRM	0.25772	2.17
160	175	16	AYIGAAFIAVYGPGPG	AAY	PGA	0.25176	2.21
180	195	16	VYYTVLNDLWGGPGPG	AVY	PGF	0.25176	2.21
201	216	16	YTVLNDLWGNKKKTER	YYT	ERM	0.2506	2.28
81	96	16	GVSLGIAAYRMQEKWK	GGV	WKV	0.24997	2.32
97	111	15	VKAAYIQLRWFFVFA	KVK	FAA	0.2494	2.36
212	226	15	KKTERMQEKWKVKKK	KKK	KKA	0.24902	2.4
11	25	15	KGIINTLQKYYCRVR	AKG	VRG	0.24811	2.44
79	93	15	IGGVSLGIAAYRMQE	FIG	QEK	0.24297	2.48
57	71	15	EAAAKAKFVAAWTLK	KEA	LKA	0.24248	2.51
140	155	16	EKWKVKLAAYRETTEA	QEK	EAA	0.24213	2.55
238	251	14	AAKKTGALLAAGAA	AAA	AAA	0.23899	2.59
209	222	14	GNKKKTERMQEKWK	WGN	WKV	0.23896	2.63
59	74	16	AAKAKFVAAWTLKAAA	AAA	AAA	0.23723	2.67
86	98	13	IAAYRMQEKWKVK	GIA	VKA	0.23688	2.71
76	90	15	AYFIGGVSLGIAAYR	AAY	YRM	0.23562	2.75
227	241	15	AKFVAAWTLKAAAKK	KAK	KKT	0.23532	2.79
46	59	14	TRGRKCCRRKKEAA	STR	AAA	0.2349	2.82
84	98	15	LGIAAYRMQEKWKVK	SLG	VKA	0.23474	2.86
42	55	14	GKCSTRGRKCCRRK	IGK	RKK	0.23305	2.9
183	195	13	TVLNDLWGGPGPG	YTV	PGF	0.22909	2.94
82	98	17	VSLGIAAYRMQEKWKVK	GVS	VKA	0.22637	2.98
135	150	16	AAYMQEKWKVKLAAYR	TAA	YRE	0.21934	3.02
171	184	14	GPGPGAFIAVYYTV	YGP	TVL	0.21893	3.06
187	200	14	DLWGGPGPGFIAVY	NDL	VYY	0.21606	3.09
239	252	14	AKKTGALLAAGAAA	AAK	AAK	0.21604	3.13
238	252	15	AAKKTGALLAAGAAA	AAA	AAK	0.21496	3.17
61	75	15	KAKFVAAWTLKAAAA	AKA	AAA	0.21454	3.21
213	228	16	KTERMQEKWKVKKKAK	KKT	AKF	0.21272	3.25
74	88	15	AAAYFIGGVSLGIAA	AAA	AAY	0.21248	3.29
9	25	17	AAKGIINTLQKYYCRVR	AAA	VRG	0.21246	3.33
238	253	16	AAKKTGALLAAGAAAK	AAA	AKK	0.21227	3.37
133	148	16	STAAYMQEKWKVKLAA	FST	AAY	0.21197	3.4
236	249	14	KAAAKKTGALLAAG	LKA	AGA	0.2118	3.44
46	60	15	TRGRKCCRRKKEAAA	STR	AAK	0.21128	3.48
62	75	14	AKFVAAWTLKAAAA	KAK	AAA	0.21091	3.52
143	156	14	KVKLAAYRETTEAA	WKV	AAI	0.20961	3.56
58	73	16	AAAKAKFVAAWTLKAA	EAA	AAA	0.20935	3.6

223	238	16	VKKKAKFVAAWTLKAA	KVK	AAA	0.20935	3.6
155	168	14	AAIIAAYIGAAFIA	EAA	IAV	0.20932	3.68
225	238	14	KKAKFVAAWTLKAA	KKK	AAA	0.20917	3.71
98	111	14	KAAYIQLRWFFVFA	VKA	FAA	0.20818	3.75
186	200	15	NDLWGGPGPGFIAVY	LND	VYY	0.20753	3.79
179	195	17	AVYYYTVLNDLWGGPGPG	IAV	PGF	0.20739	3.83
78	90	13	FIGGVSLGIAAYR	YFI	YRM	0.20725	3.87
79	94	16	IGGVSLGIAAYRMQEK	FIG	EKW	0.20722	3.91
206	220	15	DLWGNKKKTERMQEK	NDL	EKW	0.20706	3.95
26	38	13	GGRCAVLSCLPKE	RGG	KEE	0.20565	3.98
237	252	16	AAAKKTGALLAAGAAA	KAA	AAK	0.20553	4.02
58	71	14	AAAKAKFVAAWTLK	EAA	LKA	0.2049	4.06
223	236	14	VKKKAKFVAAWTLK	KVK	LKA	0.2049	4.06
27	42	16	GRCAVLSCLPKEEQIG	GGR	IGK	0.20478	4.14
39	54	16	EQIGKCSTRGRKCCRR	EEQ	RRK	0.20478	4.14
99	112	14	AAYIQLRWFFVFAA	KAA	AAY	0.20467	4.22
42	56	15	GKCSTRGRKCCRRKK	IGK	KKE	0.20388	4.26
184	198	15	VLNDLWGGPGPGFIA	TVL	IAV	0.20388	4.26
86	100	15	IAAYRMQEKWKVKAA	GIA	AAY	0.20374	4.33
213	226	14	KTERMQEKWKVKKK	KKT	KKA	0.20254	4.37
178	195	18	IAVYYYTVLNDLWGGPGPG	FIA	PGF	0.20205	4.41
131	145	15	VFSTAAYMQEKWKVK	FVF	VKL	0.20127	4.45
72	85	14	AAAAAYFIGGVSLG	KAA	LGI	0.20052	4.49
206	219	14	DLWGNKKKTERMQE	NDL	QEK	0.20013	4.53
210	224	15	NKKKTERMQEKWKVK	GNK	VKK	0.20004	4.56
13	25	13	IINTLQKYYCRVR	GII	VRG	0.19971	4.6
38	50	13	EEQIGKCSTRGRK	KEE	RKC	0.19905	4.64
237	249	13	AAAKKTGALLAAG	KAA	AGA	0.19612	4.68
47	60	14	RGRKCCRRKKEAAA	TRG	AAK	0.19589	4.72
61	74	14	KAKFVAAWTLKAAA	AKA	AAA	0.19551	4.76
151	163	13	ETTEAAIIAAYIG	RET	IGA	0.19535	4.8
75	88	14	AAYFIGGVSLGIAA	AAA	AAY	0.19363	4.84
93	105	13	EKWKVKAAYIQLR	QEK	LRW	0.19363	4.87
49	61	13	RKCCRRKKEAAAK	GRK	AKA	0.1935	4.91
205	219	15	NDLWGNKKKTERMQE	LND	QEK	0.19223	4.95
73	88	16	AAAAYFIGGVSLGIAA	AAA	AAY	0.1922	4.99
140	156	17	EKWKVKLAAYRETTEAA	QEK	AAI	0.19144	5.03
96	111	16	KVKAAYIQLRWFFVFA	WKV	FAA	0.19017	5.07
211	225	15	KKKTERMQEKWKVKK	NKK	KKK	0.18996	5.11
222	236	15	KVKKKAKFVAAWTLK	WKV	LKA	0.18954	5.15
181	195	15	YYTVLNDLWGGPGPG	VYY	PGF	0.18941	5.18
99	111	13	AAYIQLRWFFVFA	KAA	FAA	0.18922	5.22
227	240	14	AKFVAAWTLKAAAK	KAK	AKK	0.18873	5.26
197	211	15	IAVYYYTVLNDLWGNK	FIA	NKK	0.18853	5.3
236	251	16	KAAAKKTGALLAAGAA	LKA	AAA	0.18823	5.34
225	239	15	KKAKFVAAWTLKAAA	KKK	AAK	0.18814	5.38
167	179	13	IAVYGPGPGAFIA	FIA	IAV	0.18801	5.42
62	76	15	AKFVAAWTLKAAAAA	KAK	AAY	0.18752	5.45
225	240	16	KKAKFVAAWTLKAAAK	KKK	AKK	0.18579	5.49

197	213	17	IAVYYTVLNDLWGNKKK	FIA	KKT	0.18546	5.53
178	192	15	IAVYYTVLNDLWGGP	FIA	GPG	0.18514	5.57
171	188	18	GPGPGAFIAVYYTVLNDL	YGP	DLW	0.18495	5.61
79	92	14	IGGVSLGIAAYRMQ	FIG	MQE	0.18479	5.65
13	28	16	IINTLQKYYCRVRGGR	GII	GRC	0.18448	5.69
81	94	14	GVSLGIAAYRMQEK	GGV	EKW	0.18448	5.73
40	55	16	QIGKCSTRGRKCCRRK	EQI	RKK	0.18438	5.76
135	148	14	AAYMQEKWKVKLAA	TAA	AAY	0.18428	5.8
47	63	17	RGRKCCRRKKEAAAKAK	TRG	AKF	0.1836	5.84
164	179	16	AAFIAVYGPGPGAFIA	GAA	IAV	0.18358	5.88
197	212	16	IAVYYTVLNDLWGNKK	FIA	KKK	0.18352	5.92
239	251	13	AKKTGALLAAGAA	AAK	AAA	0.18321	5.96
40	54	15	QIGKCSTRGRKCCRR	EQI	RRK	0.18288	6.0
156	168	13	AIIAAYIGAAFIA	AAI	IAV	0.18182	6.03
140	154	15	EKWKVKLAAYRETTE	QEK	TEA	0.18124	6.07
113	127	15	YSYCLYWLFVAAYLR	AYS	LRW	0.18123	6.11
130	145	16	FVFSTAAYMQEKWKVK	FFV	VKL	0.18077	6.15
89	105	17	YRMQEKWKVKAAYIQLR	AYR	LRW	0.18049	6.19
214	226	13	TERMQEKWKVKKK	KTE	KKA	0.18022	6.23
73	87	15	AAAAYFIGGVSLGIA	AAA	IAA	0.17788	6.27
159	175	17	AAYIGAAFIAVYGPGPG	IAA	PGA	0.17763	6.31
148	163	16	AYRETTEAAIIAAYIG	AAY	IGA	0.17626	6.34
229	243	15	FVAAWTLKAAAKKTG	KFV	TGA	0.17538	6.38
144	156	13	VKLAAYRETTEAA	KVK	AAI	0.17284	6.42
142	156	15	WKVKLAAYRETTEAA	KWK	AAI	0.1724	6.46
206	222	17	DLWGNKKKTERMQEKWK	NDL	WKV	0.17239	6.5
38	54	17	EEQIGKCSTRGRKCCRR	KEE	RRK	0.17195	6.54
226	241	16	KAKFVAAWTLKAAAKK	KKA	KKT	0.17179	6.58
98	112	15	KAAYIQLRWFFVFAA	VKA	AAY	0.17176	6.62
216	231	16	RMQEKWKVKKKAKFVA	ERM	VAA	0.17146	6.65
129	143	15	FFVFSTAAYMQEKWK	WFF	WKV	0.17136	6.69
208	222	15	WGNKKKTERMQEKWK	LWG	WKV	0.17074	6.73
183	198	16	TVLNDLWGGPGPGFIA	YTV	IAV	0.17058	6.77
72	87	16	AAAAAYFIGGVSLGIA	KAA	IAA	0.17008	6.81
97	112	16	VKAAYIQLRWFFVFAA	KVK	AAY	0.16962	6.85
166	179	14	FIAVYGPGPGAFIA	AFI	IAV	0.16882	6.89
48	62	15	GRKCCRRKKEAAAKA	RGR	KAK	0.16877	6.92
153	168	16	TEAAIIAAYIGAAFIA	TTE	IAV	0.16768	6.96
219	231	13	EKWKVKKKAKFVA	QEK	VAA	0.16738	7.0
24	39	16	VRGGRCAVLSCLPKEE	RVR	EEQ	0.16732	7.04
226	240	15	KAKFVAAWTLKAAAK	KKA	AKK	0.16714	7.08
212	224	13	KKTERMQEKWKVK	KKK	VKK	0.1669	7.12
150	163	14	RETTEAAIIAAYIG	YRE	IGA	0.16618	7.16
47	59	13	RGRKCCRRKKEAA	TRG	AAA	0.16612	7.2
88	103	16	AYRMQEKWKVKAAYIQ	AAY	IQL	0.16553	7.23
27	40	14	GRCAVLSCLPKEEQ	GGR	EQI	0.16529	7.27
71	85	15	KAAAAAYFIGGVSLG	LKA	LGI	0.16518	7.31
219	233	15	EKWKVKKKAKFVAAW	QEK	AWT	0.16482	7.35
224	238	15	KKKAKFVAAWTLKAA	VKK	AAA	0.16433	7.39

198	213	16	AVYYTVLNDLWGNKKK	IAV	KKT	0.16431	7.43
46	58	13	TRGRKCCRRKKEA	STR	EAA	0.16416	7.47
92	105	14	QEKWKVKAAYIQLR	MQE	LRW	0.16404	7.5
135	151	17	AAYMQEKWKVKLAAYRE	TAA	RET	0.16403	7.54
205	220	16	NDLWGNKKKTERMQEK	LND	EKW	0.16395	7.58
90	103	14	RMQEKWKVKAAYIQ	YRM	IQL	0.16364	7.62
59	72	14	AAKAKFVAAWTLKA	AAA	KAA	0.16268	7.66
235	249	15	LKAAAKKTGALLAAG	TLK	AGA	0.1623	7.7
74	87	14	AAAYFIGGVSLGIA	AAA	IAA	0.1621	7.74
214	227	14	TERMQEKWKVKKKA	KTE	KAK	0.1616	7.78
59	71	13	AAKAKFVAAWTLK	AAA	LKA	0.16139	7.81
212	228	17	KKTERMQEKWKVKKKAK	KKK	AKF	0.1607	7.85
47	62	16	RGRKCCRRKKEAAAKA	TRG	KAK	0.16041	7.89
59	75	17	AAKAKFVAAWTLKAAAA	AAA	AAA	0.15995	7.93
25	37	13	RGGRCAVLSCLPK	VRG	PKE	0.15984	7.97
162	174	13	IGAAFIAVYGPGP	YIG	GPG	0.15968	8.01
91	105	15	MQEKWKVKAAYIQLR	RMQ	LRW	0.1587	8.05
234	249	16	TLKAAAKKTGALLAAG	WTL	AGA	0.15779	8.09
58	72	15	AAAKAKFVAAWTLKA	EAA	KAA	0.15754	8.12
223	237	15	VKKKAKFVAAWTLKA	KVK	KAA	0.15754	8.12
12	26	15	GIINTLQKYYCRVRG	KGI	RGG	0.15743	8.2
61	76	16	KAKFVAAWTLKAAAAA	AKA	AAY	0.15724	8.24
162	177	16	IGAAFIAVYGPGPGAF	YIG	AFI	0.15657	8.28
86	99	14	IAAYRMQEKWKVKA	GIA	KAA	0.15599	8.32
122	136	15	VAAYLRWFFVFSTAA	FVA	AAY	0.15564	8.36
7	19	13	EAAAKGIINTLQK	LEA	QKY	0.15559	8.39
197	210	14	IAVYYTVLNDLWGN	FIA	GNK	0.15559	8.43
240	253	14	KKTGALLAAGAAAK	AKK	AKK	0.15494	8.47
39	55	17	EQIGKCSTRGRKCCRRK	EEQ	RKK	0.15389	8.51
57	72	16	EAAAKAKFVAAWTLKA	KEA	KAA	0.15368	8.55
89	104	16	YRMQEKWKVKAAYIQL	AYR	QLR	0.1525	8.59
57	73	17	EAAAKAKFVAAWTLKAA	KEA	AAA	0.15222	8.63
191	203	13	GPGPGFIAVYYTV	GGP	TVL	0.15218	8.67
112	127	16	AYSYCLYWLFVAAYLR	AAY	LRW	0.15186	8.7
151	165	15	ETTEAAIIAAYIGAA	RET	AAF	0.15166	8.74
237	253	17	AAAKKTGALLAAGAAAK	KAA	AKK	0.15128	8.78
230	243	14	VAAWTLKAAAKKTG	FVA	TGA	0.1512	8.82
135	147	13	AAYMQEKWKVKLA	TAA	LAA	0.15024	8.86
85	100	16	GIAAYRMQEKWKVKAA	LGI	AAY	0.15014	8.9
73	90	18	AAAAYFIGGVSLGIAAYR	AAA	YRM	0.14999	8.94
75	89	15	AAYFIGGVSLGIAAY	AAA	AYR	0.14923	8.97
186	198	13	NDLWGGPGPGFIA	LND	IAV	0.14916	9.01
129	145	17	FFVFSTAAYMQEKWKVK	WFF	VKL	0.14904	9.05
90	105	16	RMQEKWKVKAAYIQLR	YRM	LRW	0.14902	9.09
78	93	16	FIGGVSLGIAAYRMQE	YFI	QEK	0.14881	9.13
199	213	15	VYYTVLNDLWGNKKK	AVY	KKT	0.14868	9.17
166	181	16	FIAVYGPGPGAFIAVY	AFI	VYY	0.14769	9.21
163	175	13	GAAFIAVYGPGPG	IGA	PGA	0.14735	9.25
62	74	13	AKFVAAWTLKAAA	KAK	AAA	0.1466	9.28

45	59	15	STRGRKCCRRKKEAA	CST	AAA	0.14623	9.32
205	218	14	NDLWGNKKKTERMQ	LND	MQE	0.1462	9.36
127	141	15	RWFFVFSTAAYMQEK	LRW	EKW	0.1459	9.4
141	156	16	KWKVKLAAYRETTEAA	EKW	AAI	0.14556	9.44
42	54	13	GKCSTRGRKCCRR	IGK	RRK	0.14534	9.48
41	58	18	IGKCSTRGRKCCRRKKEA	QIG	EAA	0.14532	9.52
165	179	15	AFIAVYGPGPGAFIA	AAF	IAV	0.14422	9.56
13	26	14	IINTLQKYYCRVRG	GII	RGG	0.14346	9.59
162	179	18	IGAAFIAVYGPGPGAFIA	YIG	IAV	0.14345	9.63
215	228	14	ERMQEKWKVKKKAK	TER	AKF	0.14316	9.67
60	76	17	AKAKFVAAWTLKAAAAA	AAK	AAY	0.14292	9.71
209	223	15	GNKKKTERMQEKWKV	WGN	KVK	0.14268	9.75
136	151	16	AYMQEKWKVKLAAYRE	AAY	RET	0.14254	9.79
168	181	14	AVYGPGPGAFIAVY	IAV	VYY	0.14241	9.83
225	241	17	KKAKFVAAWTLKAAAKK	KKK	KKT	0.14233	9.86
136	150	15	AYMQEKWKVKLAAYR	AAY	YRE	0.14207	9.9
135	149	15	AAYMQEKWKVKLAAY	TAA	AYR	0.14202	9.94
144	159	16	VKLAAYRETTEAAIIA	KVK	IAA	0.142	9.98
212	225	14	KKTERMQEKWKVKK	KKK	KKK	0.1419	10.02
111	124	14	AAYSYCLYWLFVAA	FAA	AAY	0.14183	10.06
218	231	14	QEKWKVKKKAKFVA	MQE	VAA	0.1418	10.1
228	241	14	KFVAAWTLKAAAKK	AKF	KKT	0.14178	10.14
147	163	17	AAYRETTEAAIIAAYIG	LAA	IGA	0.14162	10.17
45	57	13	STRGRKCCRRKKE	CST	KEA	0.14154	10.21
178	193	16	IAVYYYTVLNDLWGGPG	FIA	PGP	0.14151	10.25
49	62	14	RKCCRRKKEAAAKA	GRK	KAK	0.14126	10.29
58	74	17	AAAKAKFVAAWTLKAAA	EAA	AAA	0.14115	10.33
127	140	14	RWFFVFSTAAYMQE	LRW	QEK	0.14102	10.37
114	127	14	SYCLYWLFVAAYLR	YSY	LRW	0.1406	10.41
212	227	16	KKTERMQEKWKVKKKA	KKK	KAK	0.14039	10.44
19	33	15	KYYCRVRGGRCAVLS	QKY	LSC	0.14034	10.48
81	98	18	GVSLGIAAYRMQEKWKVK	GGV	VKA	0.13988	10.52
140	152	13	EKWKVKLAAYRET	QEK	ETT	0.13966	10.56
226	239	14	KAKFVAAWTLKAAA	KKA	AAK	0.13952	10.6
85	99	15	GIAAYRMQEKWKVKA	LGI	KAA	0.13945	10.64
133	146	14	STAAYMQEKWKVKL	FST	KLA	0.13937	10.68
216	228	13	RMQEKWKVKKKAK	ERM	AKF	0.13888	10.72
190	205	16	GGPGPGFIAVYYYTVLN	WGG	LND	0.13864	10.75
213	227	15	KTERMQEKWKVKKKA	KKT	KAK	0.13852	10.79
78	92	15	FIGGVSLGIAAYRMQ	YFI	MQE	0.1373	10.83
141	155	15	KWKVKLAAYRETTEA	EKW	EAA	0.13722	10.87
217	231	15	MQEKWKVKKKAKFVA	RMQ	VAA	0.13719	10.91
72	88	17	AAAAAYFIGGVSLGIAA	KAA	AAY	0.13698	10.95
134	147	14	TAAYMQEKWKVKLA	STA	LAA	0.1367	10.99
132	145	14	FSTAAYMQEKWKVK	VFS	VKL	0.13622	11.03
81	93	13	GVSLGIAAYRMQE	GGV	QEK	0.136	11.06
61	73	13	KAKFVAAWTLKAA	AKA	AAA	0.1359	11.1
193	207	15	GPGFIAVYYYTVLNDL	PGP	DLW	0.13544	11.14
234	247	14	TLKAAAKKTGALLA	WTL	LAA	0.13526	11.18

74	89	16	AAAYFIGGVSLGIAAY	AAA	AYR	0.13498	11.22
14	28	15	INTLQKYYCRVRGGR	IIN	GRC	0.13472	11.26
122	135	14	VAAYLRWFFVFSTA	FVA	TAA	0.13453	11.3
111	127	17	AAYSYCLYWLFVAAYLR	FAA	LRW	0.13451	11.33
109	123	15	VFAAYSYCLYWLFVA	FVF	VAA	0.13432	11.37
93	106	14	EKWKVKAAYIQLRW	QEK	RWF	0.13427	11.41
45	58	14	STRGRKCCRRKKEA	CST	EAA	0.13397	11.45
142	155	14	WKVKLAAYRETTEA	KWK	EAA	0.13397	11.45
26	43	18	GGRCAVLSCLPKEEQIGK	RGG	GKC	0.13315	11.53
93	108	16	EKWKVKAAYIQLRWFF	QEK	FFV	0.1331	11.57
24	38	15	VRGGRCAVLSCLPKE	RVR	KEE	0.13245	11.61
201	215	15	YTVLNDLWGNKKKTE	YYT	TER	0.13219	11.64
73	85	13	AAAAYFIGGVSLG	AAA	LGI	0.13186	11.68
219	232	14	EKWKVKKKAKFVAA	QEK	AAW	0.13156	11.72
173	186	14	GPGAFIAVYYTVLN	PGP	LND	0.13128	11.76
229	241	13	FVAAWTLKAAAKK	KFV	KKT	0.13077	11.8
56	72	17	KEAAAKAKFVAAWTLKA	KKE	KAA	0.13053	11.84
233	247	15	WTLKAAAKKTGALLA	AWT	LAA	0.12971	11.88
48	60	13	GRKCCRRKKEAAA	RGR	AAK	0.12959	11.91
147	160	14	AAYRETTEAAIIAA	LAA	AAY	0.12867	11.95
221	236	16	WKVKKKAKFVAAWTLK	KWK	LKA	0.12851	11.99
82	97	16	VSLGIAAYRMQEKWKV	GVS	KVK	0.1281	12.03
97	110	14	VKAAYIQLRWFFVF	KVK	VFA	0.12806	12.07
237	250	14	AAAKKTGALLAAGA	KAA	GAA	0.12795	12.11
134	148	15	TAAYMQEKWKVKLAA	STA	AAY	0.12789	12.15
130	143	14	FVFSTAAYMQEKWK	FFV	WKV	0.12771	12.19
83	98	16	SLGIAAYRMQEKWKVK	VSL	VKA	0.12767	12.22
187	202	16	DLWGGPGPGFIAVYYT	NDL	YTV	0.1276	12.26
69	83	15	TLKAAAAAYFIGGVS	WTL	VSL	0.12654	12.3
42	58	17	GKCSTRGRKCCRRKKEA	IGK	EAA	0.12596	12.34
203	218	16	VLNDLWGNKKKTERMQ	TVL	MQE	0.12565	12.38
4	19	16	HALEAAAKGIINTLQK	PHA	QKY	0.12551	12.42
26	41	16	GGRCAVLSCLPKEEQI	RGG	QIG	0.12525	12.46
53	66	14	RRKKEAAAKAKFVA	CRR	VAA	0.12502	12.5
60	72	13	AKAKFVAAWTLKA	AAK	KAA	0.12471	12.53
140	153	14	EKWKVKLAAYRETT	QEK	TTE	0.12466	12.57
12	28	17	GIINTLQKYYCRVRGGR	KGI	GRC	0.12438	12.61
158	175	18	IAAYIGAAFIAVYGPGPG	IIA	PGA	0.12426	12.65
143	155	13	KVKLAAYRETTEA	WKV	EAA	0.12424	12.69
191	209	19	GPGPGFIAVYYTVLNDLWG	GGP	WGN	0.1242	12.73
13	27	15	IINTLQKYYCRVRGG	GII	GGR	0.12415	12.77
161	174	14	YIGAAFIAVYGPGP	AYI	GPG	0.12347	12.8
56	68	13	KEAAAKAKFVAAW	KKE	AWT	0.12329	12.84
65	80	16	VAAWTLKAAAAAYFIG	FVA	IGG	0.12325	12.88
190	204	15	GGPGPGFIAVYYTVL	WGG	VLN	0.12261	12.92
173	188	16	GPGAFIAVYYTVLNDL	PGP	DLW	0.12252	12.96
201	213	13	YTVLNDLWGNKKK	YYT	KKT	0.12238	13.0
227	243	17	AKFVAAWTLKAAAKKTG	KAK	TGA	0.12191	13.04
173	187	15	GPGAFIAVYYTVLND	PGP	NDL	0.1219	13.08

224	239	16	KKKAKFVAAWTLKAAA	VKK	AAK	0.12184	13.11
127	143	17	RWFFVFSTAAYMQEKWK	LRW	WKV	0.12147	13.15
198	212	15	AVYYTVLNDLWGNKK	IAV	KKK	0.12119	13.19
25	39	15	RGGRCAVLSCLPKEE	VRG	EEQ	0.12099	13.23
82	94	13	VSLGIAAYRMQEK	GVS	EKW	0.12077	13.27
111	123	13	AAYSYCLYWLFVA	FAA	VAA	0.12076	13.31
108	123	16	FVFAAYSYCLYWLFVA	FFV	VAA	0.12064	13.35
202	215	14	TVLNDLWGNKKKTE	YTV	TER	0.12047	13.38
227	239	13	AKFVAAWTLKAAA	KAK	AAK	0.12017	13.42
214	229	16	TERMQEKWKVKKKAKF	KTE	KFV	0.12015	13.46
222	237	16	KVKKKAKFVAAWTLKA	WKV	KAA	0.12013	13.5
80	94	15	GGVSLGIAAYRMQEK	IGG	EKW	0.11977	13.54
228	243	16	KFVAAWTLKAAAKKTG	AKF	TGA	0.11955	13.58
197	209	13	IAVYYTVLNDLWG	FIA	WGN	0.11942	13.62
62	77	16	AKFVAAWTLKAAAAAY	KAK	AYF	0.11912	13.66
227	242	16	AKFVAAWTLKAAAKKT	KAK	KTG	0.11912	13.66
222	238	17	KVKKKAKFVAAWTLKAA	WKV	AAA	0.11899	13.73
45	61	17	STRGRKCCRRKKEAAAK	CST	AKA	0.11884	13.77
96	110	15	KVKAAYIQLRWFFVF	WKV	VFA	0.11846	13.81
226	238	13	KAKFVAAWTLKAA	KKA	AAA	0.11832	13.85
139	152	14	QEKWKVKLAAYRET	MQE	ETT	0.11831	13.89
149	164	16	YRETTEAAIIAAYIGA	AYR	GAA	0.1181	13.93
203	217	15	VLNDLWGNKKKTERM	TVL	RMQ	0.11727	13.97
27	39	13	GRCAVLSCLPKEE	GGR	EEQ	0.11717	14.0
10	26	17	AKGIINTLQKYYCRVRG	AAK	RGG	0.117	14.04
87	103	17	AAYRMQEKWKVKAAYIQ	IAA	IQL	0.11679	14.08
196	209	14	FIAVYYTVLNDLWG	GFI	WGN	0.11664	14.12
241	253	13	KTGALLAAGAAAK	KKT	AKK	0.11612	14.16
206	218	13	DLWGNKKKTERMQ	NDL	MQE	0.1161	14.2
6	18	13	LEAAAKGIINTLQ	ALE	LQK	0.11605	14.24
196	210	15	FIAVYYTVLNDLWGN	GFI	GNK	0.11591	14.27
196	211	16	FIAVYYTVLNDLWGNK	GFI	NKK	0.11578	14.31
80	93	14	GGVSLGIAAYRMQE	IGG	QEK	0.11576	14.35
223	239	17	VKKKAKFVAAWTLKAAA	KVK	AAK	0.1157	14.39
156	170	15	AIIAAYIGAAFIAVY	AAI	VYG	0.1156	14.43
211	227	17	KKKTERMQEKWKVKKKA	NKK	KAK	0.11548	14.47
210	226	17	NKKKTERMQEKWKVKKK	GNK	KKA	0.1153	14.51
210	222	13	NKKKTERMQEKWK	GNK	WKV	0.11511	14.55
182	198	17	YTVLNDLWGGPGPGFIA	YYT	IAV	0.11501	14.58
208	224	17	WGNKKKTERMQEKWKVK	LWG	VKK	0.11463	14.62
144	160	17	VKLAAYRETTEAAIIAA	KVK	AAY	0.11436	14.66
211	228	18	KKKTERMQEKWKVKKKAK	NKK	AKF	0.1141	14.7
232	247	16	AWTLKAAAKKTGALLA	AAW	LAA	0.11396	14.74
158	172	15	IAAYIGAAFIAVYGP	IIA	GPG	0.11386	14.78
56	70	15	KEAAAKAKFVAAWTL	KKE	TLK	0.11352	14.82
157	172	16	IIAAYIGAAFIAVYGP	AII	GPG	0.1135	14.85
63	76	14	KFVAAWTLKAAAAA	AKF	AAY	0.11298	14.89
87	100	14	AAYRMQEKWKVKAA	IAA	AAY	0.11298	14.89
178	194	17	IAVYYTVLNDLWGGPGP	FIA	GPG	0.11292	14.97

75	87	13	AAYFIGGVSLGIA	AAA	IAA	0.11267	15.01
64	78	15	FVAAWTLKAAAAAYF	KFV	YFI	0.11237	15.05
12	27	16	GIINTLQKYYCRVRGG	KGI	GGR	0.1123	15.09
11	26	16	KGIINTLQKYYCRVRG	AKG	RGG	0.11207	15.13
118	132	15	YWLFVAAYLRWFFVF	LYW	VFS	0.11179	15.16
86	103	18	IAAYRMQEKWKVKAAYIQ	GIA	IQL	0.11169	15.2
56	73	18	KEAAAKAKFVAAWTLKAA	KKE	AAA	0.1116	15.24
38	55	18	EEQIGKCSTRGRKCCRRK	KEE	RKK	0.11153	15.28
193	206	14	GPGFIAVYYTVLND	PGP	NDL	0.11109	15.32
79	96	18	IGGVSLGIAAYRMQEKWK	FIG	WKV	0.111	15.36
24	40	17	VRGGRCAVLSCLPKEEQ	RVR	EQI	0.11062	15.4
96	109	14	KVKAAYIQLRWFFV	WKV	FVF	0.11006	15.44
162	176	15	IGAAFIAVYGPGPGA	YIG	GAF	0.10994	15.47
146	160	15	LAAYRETTEAAIIAA	KLA	AAY	0.10966	15.51
184	200	17	VLNDLWGGPGPGFIAVY	TVL	VYY	0.10959	15.55
91	103	13	MQEKWKVKAAYIQ	RMQ	IQL	0.10958	15.59
77	92	16	YFIGGVSLGIAAYRMQ	AYF	MQE	0.10928	15.63
156	172	17	AIIAAYIGAAFIAVYGP	AAI	GPG	0.1092	15.67
8	25	18	AAAKGIINTLQKYYCRVR	EAA	VRG	0.10912	15.71
167	180	14	IAVYGPGPGAFIAV	FIA	AVY	0.10893	15.74
79	91	13	IGGVSLGIAAYRM	FIG	RMQ	0.10844	15.78
45	60	16	STRGRKCCRRKKEAAA	CST	AAK	0.10842	15.82
15	28	14	NTLQKYYCRVRGGR	INT	GRC	0.10824	15.86
123	136	14	AAYLRWFFVFSTAA	VAA	AAY	0.10797	15.9
25	40	16	RGGRCAVLSCLPKEEQ	VRG	EQI	0.10731	15.94
70	83	14	LKAAAAAYFIGGVS	TLK	VSL	0.10729	15.98
176	190	15	AFIAVYYTVLNDLWG	GAF	WGG	0.1071	16.02
139	151	13	QEKWKVKLAAYRE	MQE	RET	0.10702	16.05
210	225	16	NKKKTERMQEKWKVKK	GNK	KKK	0.10693	16.09
151	164	14	ETTEAAIIAAYIGA	RET	GAA	0.10682	16.13
71	83	13	KAAAAAYFIGGVS	LKA	VSL	0.1068	16.17
46	62	17	TRGRKCCRRKKEAAAKA	STR	KAK	0.1063	16.21
99	113	15	AAYIQLRWFFVFAAY	KAA	AYS	0.10625	16.25
230	244	15	VAAWTLKAAAKKTGA	FVA	GAL	0.10616	16.29
78	91	14	FIGGVSLGIAAYRM	YFI	RMQ	0.10563	16.32
234	248	15	TLKAAAKKTGALLAA	WTL	AAG	0.10561	16.36
230	245	16	VAAWTLKAAAKKTGAL	FVA	ALL	0.1055	16.4
54	66	13	RKKEAAAKAKFVA	RRK	VAA	0.10541	16.44
236	250	15	KAAAKKTGALLAAGA	LKA	GAA	0.1054	16.48
156	169	14	AIIAAYIGAAFIAV	AAI	AVY	0.10535	16.52
64	77	14	FVAAWTLKAAAAAY	KFV	AYF	0.10529	16.56
229	242	14	FVAAWTLKAAAKKT	KFV	KTG	0.10529	16.56
49	64	16	RKCCRRKKEAAAKAKF	GRK	KFV	0.10503	16.63
46	63	18	TRGRKCCRRKKEAAAKAK	STR	AKF	0.10502	16.67
120	134	15	LFVAAYLRWFFVFST	WLF	STA	0.10489	16.71
5	19	15	ALEAAAKGIINTLQK	HAL	QKY	0.10458	16.75
177	192	16	FIAVYYTVLNDLWGGP	AFI	GPG	0.10453	16.79
70	85	16	LKAAAAAYFIGGVSLG	TLK	LGI	0.10434	16.83
84	96	13	LGIAAYRMQEKWK	SLG	WKV	0.10427	16.87

64	76	13	FVAAWTLKAAAAA	KFV	AAY	0.10421	16.91
209	225	17	GNKKKTERMQEKWKVKK	WGN	KKK	0.10404	16.94
236	252	17	KAAAKKTGALLAAGAAA	LKA	AAK	0.10403	16.98
178	190	13	IAVYYTVLNDLWG	FIA	WGG	0.104	17.02
54	68	15	RKKEAAAKAKFVAAW	RRK	AWT	0.1038	17.06
66	80	15	AAWTLKAAAAAYFIG	VAA	IGG	0.10372	17.1
17	32	16	LQKYYCRVRGGRCAVL	TLQ	VLS	0.10334	17.14
149	165	17	YRETTEAAIIAAYIGAA	AYR	AAF	0.10302	17.18
198	211	14	AVYYTVLNDLWGNK	IAV	NKK	0.10263	17.21
99	114	16	AAYIQLRWFFVFAAYS	KAA	YSY	0.1022	17.25
77	91	15	YFIGGVSLGIAAYRM	AYF	RMQ	0.10199	17.29
224	237	14	KKKAKFVAAWTLKA	VKK	KAA	0.10194	17.33
121	135	15	FVAAYLRWFFVFSTA	LFV	TAA	0.10188	17.37
203	219	17	VLNDLWGNKKKTERMQE	TVL	QEK	0.10151	17.41
229	244	16	FVAAWTLKAAAKKTGA	KFV	GAL	0.10151	17.45
56	69	14	KEAAAKAKFVAAWT	KKE	WTL	0.10144	17.49
2	15	14	PPHALEAAAKGIIN	APP	INT	0.10134	17.52
224	236	13	KKKAKFVAAWTLK	VKK	LKA	0.10113	17.56
118	133	16	YWLFVAAYLRWFFVFS	LYW	FST	0.10112	17.6
179	192	14	AVYYTVLNDLWGGP	IAV	GPG	0.10079	17.64
211	223	13	KKKTERMQEKWKV	NKK	KVK	0.10065	17.68
93	109	17	EKWKVKAAYIQLRWFFV	QEK	FVF	0.10052	17.72
133	149	17	STAAYMQEKWKVKLAAY	FST	AYR	0.10037	17.76
67	81	15	AWTLKAAAAAYFIGG	AAW	GGV	0.1001	17.79
179	194	16	AVYYTVLNDLWGGPGP	IAV	GPG	0.10004	17.83
68	83	16	WTLKAAAAAYFIGGVS	AWT	VSL	0.10003	17.87
66	81	16	AAWTLKAAAAAYFIGG	VAA	GGV	0.09987	17.91
133	150	18	STAAYMQEKWKVKLAAYR	FST	YRE	0.09973	17.95
80	96	17	GGVSLGIAAYRMQEKWK	IGG	WKV	0.09971	17.99
17	31	15	LQKYYCRVRGGRCAV	TLQ	AVL	0.09951	18.03
107	123	17	FFVFAAYSYCLYWLFVA	WFF	VAA	0.09946	18.07
111	126	16	AAYSYCLYWLFVAAYL	FAA	YLR	0.09919	18.1
109	124	16	VFAAYSYCLYWLFVAA	FVF	AAY	0.09919	18.14
40	56	17	QIGKCSTRGRKCCRRKK	EQI	KKE	0.09911	18.18
126	140	15	LRWFFVFSTAAYMQE	YLR	QEK	0.09902	18.22
30	42	13	AVLSCLPKEEQIG	CAV	IGK	0.09899	18.26
225	237	13	KKAKFVAAWTLKA	KKK	KAA	0.09897	18.3
17	33	17	LQKYYCRVRGGRCAVLS	TLQ	LSC	0.09887	18.34
240	252	13	KKTGALLAAGAAA	AKK	AAK	0.09866	18.38
202	217	16	TVLNDLWGNKKKTERM	YTV	RMQ	0.09812	18.41
84	99	16	LGIAAYRMQEKWKVKA	SLG	KAA	0.09719	18.45
121	136	16	FVAAYLRWFFVFSTAA	LFV	AAY	0.09716	18.49
63	75	13	KFVAAWTLKAAAA	AKF	AAA	0.09693	18.53
120	133	14	LFVAAYLRWFFVFS	WLF	FST	0.0969	18.57
65	78	14	VAAWTLKAAAAAYF	FVA	YFI	0.09687	18.61
209	226	18	GNKKKTERMQEKWKVKKK	WGN	KKA	0.09683	18.65
139	154	16	QEKWKVKLAAYRETTE	MQE	TEA	0.09654	18.68
72	90	19	AAAAAYFIGGVSLGIAAYR	KAA	YRM	0.09642	18.72
96	112	17	KVKAAYIQLRWFFVFAA	WKV	AAY	0.0964	18.76

30	43	14	AVLSCLPKEEQIGK	CAV	GKC	0.09628	18.8
139	155	17	QEKWKVKLAAYRETTEA	MQE	EAA	0.09613	18.84
95	111	17	WKVKAAYIQLRWFFVFA	KWK	FAA	0.0961	18.88
154	169	16	EAAIIAAYIGAAFIAV	TEA	AVY	0.09581	18.92
128	143	16	WFFVFSTAAYMQEKWK	RWF	WKV	0.09512	18.96
167	183	17	IAVYGPGPGAFIAVYYT	FIA	YTV	0.09493	18.99
152	168	17	TTEAAIIAAYIGAAFIA	ETT	IAV	0.0949	19.03
120	135	16	LFVAAYLRWFFVFSTA	WLF	TAA	0.09488	19.07
78	94	17	FIGGVSLGIAAYRMQEK	YFI	EKW	0.0946	19.11
24	36	13	VRGGRCAVLSCLP	RVR	LPK	0.09401	19.15
68	81	14	WTLKAAAAAYFIGG	AWT	GGV	0.09392	19.19
90	104	15	RMQEKWKVKAAYIQL	YRM	QLR	0.09385	19.23
35	54	20	LPKEEQIGKCSTRGRKCCRR	CLP	RRK	0.09364	19.26
152	165	14	TTEAAIIAAYIGAA	ETT	AAF	0.09363	19.3
134	150	17	TAAYMQEKWKVKLAAYR	STA	YRE	0.09353	19.34
122	134	13	VAAYLRWFFVFST	FVA	STA	0.09351	19.38
179	193	15	AVYYTVLNDLWGGPG	IAV	PGP	0.09345	19.42
177	190	14	FIAVYYTVLNDLWG	AFI	WGG	0.09339	19.46
27	43	17	GRCAVLSCLPKEEQIGK	GGR	GKC	0.09334	19.5
233	249	17	WTLKAAAKKTGALLAAG	AWT	AGA	0.09298	19.54
121	134	14	FVAAYLRWFFVFST	LFV	STA	0.09284	19.57
155	169	15	AAIIAAYIGAAFIAV	EAA	AVY	0.0925	19.61
214	231	18	TERMQEKWKVKKKAKFVA	KTE	VAA	0.09238	19.65
200	213	14	YYTVLNDLWGNKKK	VYY	KKT	0.09221	19.69
219	236	18	EKWKVKKKAKFVAAWTLK	QEK	LKA	0.09181	19.73
162	178	17	IGAAFIAVYGPGPGAFI	YIG	FIA	0.09181	19.77
81	97	17	GVSLGIAAYRMQEKWKV	GGV	KVK	0.0917	19.81
75	91	17	AAYFIGGVSLGIAAYRM	AAA	RMQ	0.09131	19.85
193	205	13	GPGFIAVYYYTVLN	PGP	LND	0.09125	19.88
193	209	17	GPGFIAVYYYTVLNDLWG	PGP	WGN	0.09122	19.92
23	36	14	RVRGGRCAVLSCLP	CRV	LPK	0.09107	19.96
73	89	17	AAAAYFIGGVSLGIAAY	AAA	AYR	0.09101	20.0
110	123	14	FAAYSYCLYWLFVA	VFA	VAA	0.09091	20.04
97	109	13	VKAAYIQLRWFFV	KVK	FVF	0.09075	20.08
203	215	13	VLNDLWGNKKKTE	TVL	TER	0.09053	20.12
95	109	15	WKVKAAYIQLRWFFV	KWK	FVF	0.09052	20.15
160	174	15	AYIGAAFIAVYGPGP	AAY	GPG	0.09052	20.15
180	194	15	VYYTVLNDLWGGPGP	AVY	GPG	0.09052	20.15
199	212	14	VYYTVLNDLWGNKK	AVY	KKK	0.0904	20.27
83	96	14	SLGIAAYRMQEKWK	VSL	WKV	0.09019	20.31
19	32	14	KYYCRVRGGRCAVL	QKY	VLS	0.09012	20.35
210	223	14	NKKKTERMQEKWKV	GNK	KVK	0.09011	20.39
224	240	17	KKKAKFVAAWTLKAAAK	VKK	AKK	0.08968	20.43
235	248	14	LKAAAKKTGALLAA	TLK	AAG	0.08954	20.46
207	222	16	LWGNKKKTERMQEKWK	DLW	WKV	0.08952	20.5
124	139	16	AYLRWFFVFSTAAYMQ	AAY	MQE	0.08944	20.54
131	143	13	VFSTAAYMQEKWK	FVF	WKV	0.0894	20.58
20	33	14	YYCRVRGGRCAVLS	KYY	LSC	0.08931	20.62
154	167	14	EAAIIAAYIGAAFI	TEA	FIA	0.08927	20.66

236	248	13	KAAAKKTGALLAA	LKA	AAG	0.08913	20.7
151	166	16	ETTEAAIIAAYIGAAF	RET	AFI	0.08899	20.74
40	57	18	QIGKCSTRGRKCCRRKKE	EQI	KEA	0.0888	20.77
231	245	15	AAWTLKAAAKKTGAL	VAA	ALL	0.08879	20.81
144	158	15	VKLAAYRETTEAAII	KVK	IIA	0.08871	20.85
235	251	17	LKAAAKKTGALLAAGAA	TLK	AAA	0.08862	20.89
57	74	18	EAAAKAKFVAAWTLKAAA	KEA	AAA	0.08859	20.93
200	216	17	YYTVLNDLWGNKKKTER	VYY	ERM	0.08849	20.97
65	81	17	VAAWTLKAAAAAYFIGG	FVA	GGV	0.08846	21.01
77	93	17	YFIGGVSLGIAAYRMQE	AYF	QEK	0.08828	21.04
216	229	14	RMQEKWKVKKKAKF	ERM	KFV	0.08816	21.08
213	225	13	KTERMQEKWKVKK	KKT	KKK	0.08803	21.12
105	118	14	RWFFVFAAYSYCLY	LRW	LYW	0.088	21.16
41	59	19	IGKCSTRGRKCCRRKKEAA	QIG	AAA	0.0879	21.2
64	80	17	FVAAWTLKAAAAAYFIG	KFV	IGG	0.08784	21.24
205	222	18	NDLWGNKKKTERMQEKWK	LND	WKV	0.08782	21.28
218	233	16	QEKWKVKKKAKFVAAW	MQE	AWT	0.08779	21.32
235	247	13	LKAAAKKTGALLA	TLK	LAA	0.08747	21.35
63	77	15	KFVAAWTLKAAAAAY	AKF	AYF	0.08707	21.39
228	242	15	KFVAAWTLKAAAKKT	AKF	KTG	0.08707	21.39
158	173	16	IAAYIGAAFIAVYGPG	IIA	PGP	0.08703	21.47
111	125	15	AAYSYCLYWLFVAAY	FAA	AYL	0.08684	21.51
92	106	15	QEKWKVKAAYIQLRW	MQE	RWF	0.08676	21.55
228	240	13	KFVAAWTLKAAAK	AKF	AKK	0.08673	21.59
186	199	14	NDLWGGPGPGFIAV	LND	AVY	0.08642	21.62
132	147	16	FSTAAYMQEKWKVKLA	VFS	LAA	0.0864	21.66
159	172	14	AAYIGAAFIAVYGP	IAA	GPG	0.08632	21.7
71	87	17	KAAAAAYFIGGVSLGIA	LKA	IAA	0.08608	21.74
205	217	13	NDLWGNKKKTERM	LND	RMQ	0.0858	21.78
10	24	15	AKGIINTLQKYYCRV	AAK	RVR	0.08577	21.82
67	80	14	AWTLKAAAAAYFIG	AAW	IGG	0.08569	21.86
159	174	16	AAYIGAAFIAVYGPGP	IAA	GPG	0.08569	21.9
219	234	16	EKWKVKKKAKFVAAWT	QEK	WTL	0.08526	21.93
218	232	15	QEKWKVKKKAKFVAA	MQE	AAW	0.08501	21.97
20	37	18	YYCRVRGGRCAVLSCLPK	KYY	PKE	0.08498	22.01
231	247	17	AAWTLKAAAKKTGALLA	VAA	LAA	0.08475	22.05
141	154	14	KWKVKLAAYRETTE	EKW	TEA	0.08467	22.09
126	141	16	LRWFFVFSTAAYMQEK	YLR	EKW	0.08445	22.13
129	144	16	FFVFSTAAYMQEKWKV	WFF	KVK	0.08434	22.17
2	18	17	PPHALEAAAKGIINTLQ	APP	LQK	0.08418	22.21
238	250	13	AAKKTGALLAAGA	AAA	GAA	0.08414	22.24
201	214	14	YTVLNDLWGNKKKT	YYT	KTE	0.08411	22.28
168	183	16	AVYGPGPGAFIAVYYT	IAV	YTV	0.0841	22.32
208	223	16	WGNKKKTERMQEKWKV	LWG	KVK	0.08404	22.36
196	212	17	FIAVYYTVLNDLWGNKK	GFI	KKK	0.08401	22.4
155	170	16	AAIIAAYIGAAFIAVY	EAA	VYG	0.08368	22.44
146	159	14	LAAYRETTEAAIIA	KLA	IAA	0.08366	22.48
233	248	16	WTLKAAAKKTGALLAA	AWT	AAG	0.08348	22.51
112	126	15	AYSYCLYWLFVAAYL	AAY	YLR	0.08348	22.55

4	18	15	HALEAAAKGIINTLQ	PHA	LQK	0.08339	22.59
157	175	19	IIAAYIGAAFIAVYGPGPG	AII	PGA	0.08328	22.63
190	202	13	GGPGPGFIAVYYT	WGG	YTV	0.08321	22.67
54	67	14	RKKEAAAKAKFVAA	RRK	AAW	0.08285	22.71
215	231	17	ERMQEKWKVKKKAKFVA	TER	VAA	0.08283	22.75
216	230	15	RMQEKWKVKKKAKFV	ERM	FVA	0.08266	22.79
91	104	14	MQEKWKVKAAYIQL	RMQ	QLR	0.08239	22.82
58	75	18	AAAKAKFVAAWTLKAAAA	EAA	AAA	0.08215	22.86
57	70	14	EAAAKAKFVAAWTL	KEA	TLK	0.08212	22.9
113	126	14	YSYCLYWLFVAAYL	AYS	YLR	0.08212	22.94
127	139	13	RWFFVFSTAAYMQ	LRW	MQE	0.08181	22.98
84	97	14	LGIAAYRMQEKWKV	SLG	KVK	0.08162	23.02
98	110	13	KAAYIQLRWFFVF	VKA	VFA	0.08154	23.06
151	168	18	ETTEAAIIAAYIGAAFIA	RET	IAV	0.08153	23.09
2	19	18	PPHALEAAAKGIINTLQK	APP	QKY	0.08152	23.13
110	124	15	FAAYSYCLYWLFVAA	VFA	AAY	0.08144	23.17
101	114	14	YIQLRWFFVFAAYS	AYI	YSY	0.08129	23.21
134	149	16	TAAYMQEKWKVKLAAY	STA	AYR	0.08125	23.25
150	165	16	RETTEAAIIAAYIGAA	YRE	AAF	0.08112	23.29
55	71	17	KKEAAAKAKFVAAWTLK	RKK	LKA	0.08109	23.33
169	181	13	VYGPGPGAFIAVY	AVY	VYY	0.08102	23.37
118	131	14	YWLFVAAYLRWFFV	LYW	FVF	0.08095	23.4
220	236	17	KWKVKKKAKFVAAWTLK	EKW	LKA	0.08087	23.44
86	101	16	IAAYRMQEKWKVKAAY	GIA	AYI	0.08084	23.48
49	66	18	RKCCRRKKEAAAKAKFVA	GRK	VAA	0.08075	23.52
143	159	17	KVKLAAYRETTEAAIIA	WKV	IAA	0.08071	23.56
23	38	16	RVRGGRCAVLSCLPKE	CRV	KEE	0.08068	23.6
139	153	15	QEKWKVKLAAYRETT	MQE	TTE	0.08055	23.64
95	110	16	WKVKAAYIQLRWFFVF	KWK	VFA	0.08031	23.68
159	173	15	AAYIGAAFIAVYGPG	IAA	PGP	0.08004	23.71
231	243	13	AAWTLKAAAKKTG	VAA	TGA	0.08	23.75
129	141	13	FFVFSTAAYMQEK	WFF	EKW	0.07952	23.79
208	220	13	WGNKKKTERMQEK	LWG	EKW	0.07923	23.83
164	177	14	AAFIAVYGPGPGAF	GAA	AFI	0.07921	23.87
190	206	17	GGPGPGFIAVYYTVLND	WGG	NDL	0.0791	23.91
113	128	16	YSYCLYWLFVAAYLRW	AYS	RWF	0.07902	23.95
25	38	14	RGGRCAVLSCLPKE	VRG	KEE	0.07894	23.98
16	28	13	TLQKYYCRVRGGR	NTL	GRC	0.07867	24.02
131	147	17	VFSTAAYMQEKWKVKLA	FVF	LAA	0.07844	24.06
93	107	15	EKWKVKAAYIQLRWF	QEK	WFF	0.07844	24.1
202	218	17	TVLNDLWGNKKKTERMQ	YTV	MQE	0.07836	24.14
62	78	17	AKFVAAWTLKAAAAAYF	KAK	YFI	0.07811	24.18
84	100	17	LGIAAYRMQEKWKVKAA	SLG	AAY	0.078	24.22
37	50	14	KEEQIGKCSTRGRK	PKE	RKC	0.07781	24.26
156	175	20	AIIAAYIGAAFIAVYGPGPG	AAI	PGA	0.07773	24.29
189	202	14	WGGPGPGFIAVYYT	LWG	YTV	0.07765	24.33
102	118	17	IQLRWFFVFAAYSYCLY	YIQ	LYW	0.07754	24.37
153	165	13	TEAAIIAAYIGAA	TTE	AAF	0.07753	24.41
165	181	17	AFIAVYGPGPGAFIAVY	AAF	VYY	0.07752	24.45

53	68	16	RRKKEAAAKAKFVAAW	CRR	AWT	0.07741	24.49
55	68	14	KKEAAAKAKFVAAW	RKK	AWT	0.0772	24.53
48	64	17	GRKCCRRKKEAAAKAKF	RGR	KFV	0.0771	24.56
220	233	14	KWKVKKKAKFVAAW	EKW	AWT	0.077	24.6
177	195	19	FIAVYYTVLNDLWGGPGPG	AFI	PGF	0.07669	24.64
63	78	16	KFVAAWTLKAAAAAYF	AKF	YFI	0.0766	24.68
94	109	16	KWKVKAAYIQLRWFFV	EKW	FVF	0.07643	24.72
130	144	15	FVFSTAAYMQEKWKV	FFV	KVK	0.07625	24.76
169	183	15	VYGPGPGAFIAVYYT	AVY	YTV	0.0761	24.8
23	39	17	RVRGGRCAVLSCLPKEE	CRV	EEQ	0.07597	24.84
204	216	13	LNDLWGNKKKTER	VLN	ERM	0.07595	24.87
76	89	14	AYFIGGVSLGIAAY	AAY	AYR	0.07583	24.91
136	149	14	AYMQEKWKVKLAAY	AAY	AYR	0.07583	24.91
199	216	18	VYYTVLNDLWGNKKKTER	AVY	ERM	0.07567	24.99
69	85	17	TLKAAAAAYFIGGVSLG	WTL	LGI	0.07561	25.03
164	178	15	AAFIAVYGPGPGAFI	GAA	FIA	0.0756	25.07
76	91	16	AYFIGGVSLGIAAYRM	AAY	RMQ	0.07551	25.11
94	108	15	KWKVKAAYIQLRWFF	EKW	FFV	0.07543	25.15
59	76	18	AAKAKFVAAWTLKAAAAA	AAA	AAY	0.07542	25.18
126	139	14	LRWFFVFSTAAYMQ	YLR	MQE	0.07531	25.22
186	202	17	NDLWGGPGPGFIAVYYT	LND	YTV	0.07531	25.26
229	245	17	FVAAWTLKAAAKKTGAL	KFV	ALL	0.07519	25.3
76	88	13	AYFIGGVSLGIAA	AAY	AAY	0.07505	25.34
88	100	13	AYRMQEKWKVKAA	AAY	AAY	0.07505	25.34
100	112	13	AYIQLRWFFVFAA	AAY	AAY	0.07505	25.34
112	124	13	AYSYCLYWLFVAA	AAY	AAY	0.07505	25.34
124	136	13	AYLRWFFVFSTAA	AAY	AAY	0.07505	25.34
136	148	13	AYMQEKWKVKLAA	AAY	AAY	0.07505	25.34
148	160	13	AYRETTEAAIIAA	AAY	AAY	0.07505	25.34
53	67	15	RRKKEAAAKAKFVAA	CRR	AAW	0.07495	25.61
147	159	13	AAYRETTEAAIIA	LAA	IAA	0.07487	25.65
14	27	14	INTLQKYYCRVRGG	IIN	GGR	0.07473	25.69
69	81	13	TLKAAAAAYFIGG	WTL	GGV	0.0747	25.73
166	180	15	FIAVYGPGPGAFIAV	AFI	AVY	0.07461	25.76
182	194	13	YTVLNDLWGGPGP	YYT	GPG	0.07451	25.8
61	77	17	KAKFVAAWTLKAAAAAY	AKA	AYF	0.07445	25.84
185	198	14	LNDLWGGPGPGFIA	VLN	IAV	0.07438	25.88
114	128	15	SYCLYWLFVAAYLRW	YSY	RWF	0.07437	25.92
184	199	16	VLNDLWGGPGPGFIAV	TVL	AVY	0.07427	25.96
19	37	19	KYYCRVRGGRCAVLSCLPK	QKY	PKE	0.07401	26.0
163	179	17	GAAFIAVYGPGPGAFIA	IGA	IAV	0.07398	26.03
136	152	17	AYMQEKWKVKLAAYRET	AAY	ETT	0.07385	26.07
157	170	14	IIAAYIGAAFIAVY	AII	VYG	0.07383	26.11
176	192	17	AFIAVYYTVLNDLWGGP	GAF	GPG	0.07366	26.15
95	108	14	WKVKAAYIQLRWFF	KWK	FFV	0.07365	26.19
231	244	14	AAWTLKAAAKKTGA	VAA	GAL	0.07365	26.19
85	97	13	GIAAYRMQEKWKV	LGI	KVK	0.07363	26.27
191	210	20	GPGPGFIAVYYTVLNDLWGN	GGP	GNK	0.07358	26.31
223	240	18	VKKKAKFVAAWTLKAAAK	KVK	AKK	0.07351	26.34

98	113	16	KAAYIQLRWFFVFAAY	VKA	AYS	0.0735	26.38
135	152	18	AAYMQEKWKVKLAAYRET	TAA	ETT	0.07336	26.42
232	245	14	AWTLKAAAKKTGAL	AAW	ALL	0.07336	26.46
196	213	18	FIAVYYTVLNDLWGNKKK	GFI	KKT	0.07328	26.5
173	185	13	GPGAFIAVYYTVL	PGP	VLN	0.073	26.54
145	160	16	KLAAYRETTEAAIIAA	VKL	AAY	0.07293	26.58
42	59	18	GKCSTRGRKCCRRKKEAA	IGK	AAA	0.07292	26.62
161	173	13	YIGAAFIAVYGPG	AYI	PGP	0.07252	26.65
153	166	14	TEAAIIAAYIGAAF	TTE	AFI	0.07235	26.69
124	140	17	AYLRWFFVFSTAAYMQE	AAY	QEK	0.07226	26.73
189	204	16	WGGPGPGFIAVYYTVL	LWG	VLN	0.07194	26.77
6	20	15	LEAAAKGIINTLQKY	ALE	KYY	0.0715	26.81
39	56	18	EQIGKCSTRGRKCCRRKK	EEQ	KKE	0.0714	26.85
154	166	13	EAAIIAAYIGAAF	TEA	AFI	0.07135	26.89
187	201	15	DLWGGPGPGFIAVYY	NDL	YYT	0.07134	26.92
167	182	16	IAVYGPGPGAFIAVYY	FIA	YYT	0.0712	26.96
123	135	13	AAYLRWFFVFSTA	VAA	TAA	0.07118	27.0
171	190	20	GPGPGAFIAVYYTVLNDLWG	YGP	WGG	0.07076	27.04
176	188	13	AFIAVYYTVLNDL	GAF	DLW	0.07055	27.08
9	24	16	AAKGIINTLQKYYCRV	AAA	RVR	0.07035	27.12
183	197	15	TVLNDLWGGPGPGFI	YTV	FIA	0.07024	27.16
128	141	14	WFFVFSTAAYMQEK	RWF	EKW	0.0702	27.2
117	132	16	LYWLFVAAYLRWFFVF	CLY	VFS	0.07013	27.23
131	144	14	VFSTAAYMQEKWKV	FVF	KVK	0.06998	27.27
81	95	15	GVSLGIAAYRMQEKW	GGV	KWK	0.06975	27.31
160	173	14	AYIGAAFIAVYGPG	AAY	PGP	0.0697	27.35
180	193	14	VYYTVLNDLWGGPG	AVY	PGP	0.0697	27.35
138	151	14	MQEKWKVKLAAYRE	YMQ	RET	0.06953	27.43
158	174	17	IAAYIGAAFIAVYGPGP	IIA	GPG	0.06944	27.47
102	115	14	IQLRWFFVFAAYSY	YIQ	SYC	0.06942	27.5
150	164	15	RETTEAAIIAAYIGA	YRE	GAA	0.06931	27.54
215	229	15	ERMQEKWKVKKKAKF	TER	KFV	0.06931	27.54
65	77	13	VAAWTLKAAAAAY	FVA	AYF	0.06924	27.62
230	242	13	VAAWTLKAAAKKT	FVA	KTG	0.06924	27.62
214	230	17	TERMQEKWKVKKKAKFV	KTE	FVA	0.06922	27.7
184	197	14	VLNDLWGGPGPGFI	TVL	FIA	0.0692	27.74
91	106	16	MQEKWKVKAAYIQLRW	RMQ	RWF	0.0692	27.78
153	167	15	TEAAIIAAYIGAAFI	TTE	FIA	0.06905	27.81
187	199	13	DLWGGPGPGFIAV	NDL	AVY	0.06863	27.85
96	108	13	KVKAAYIQLRWFF	WKV	FFV	0.0683	27.89
195	209	15	GFIAVYYTVLNDLWG	PGF	WGN	0.06798	27.93
104	118	15	LRWFFVFAAYSYCLY	QLR	LYW	0.06794	27.97
56	74	19	KEAAAKAKFVAAWTLKAAA	KKE	AAA	0.06787	28.01
217	232	16	MQEKWKVKKKAKFVAA	RMQ	AAW	0.0678	28.05
143	158	16	KVKLAAYRETTEAAII	WKV	IIA	0.06764	28.09
145	159	15	KLAAYRETTEAAIIA	VKL	IAA	0.0675	28.12
120	136	17	LFVAAYLRWFFVFSTAA	WLF	AAY	0.06745	28.16
120	132	13	LFVAAYLRWFFVF	WLF	VFS	0.06736	28.2
133	151	19	STAAYMQEKWKVKLAAYRE	FST	RET	0.06728	28.24

118	134	17	YWLFVAAYLRWFFVFST	LYW	STA	0.06725	28.28
206	224	19	DLWGNKKKTERMQEKWKVK	NDL	VKK	0.06717	28.32
80	92	13	GGVSLGIAAYRMQ	IGG	MQE	0.06715	28.36
39	57	19	EQIGKCSTRGRKCCRRKKE	EEQ	KEA	0.06686	28.39
152	166	15	TTEAAIIAAYIGAAF	ETT	AFI	0.06664	28.43
235	250	16	LKAAAKKTGALLAAGA	TLK	GAA	0.06658	28.47
108	124	17	FVFAAYSYCLYWLFVAA	FFV	AAY	0.06641	28.51
35	55	21	LPKEEQIGKCSTRGRKCCRRK	CLP	RKK	0.06637	28.55
101	115	15	YIQLRWFFVFAAYSY	AYI	SYC	0.06631	28.59
201	217	17	YTVLNDLWGNKKKTERM	YYT	RMQ	0.06615	28.63
236	253	18	KAAAKKTGALLAAGAAAK	LKA	AKK	0.06609	28.67
207	220	14	LWGNKKKTERMQEK	DLW	EKW	0.06607	28.7
87	99	13	AAYRMQEKWKVKA	IAA	KAA	0.06598	28.74
14	26	13	INTLQKYYCRVRG	IIN	RGG	0.06587	28.78
139	156	18	QEKWKVKLAAYRETTEAA	MQE	AAI	0.06561	28.82
67	83	17	AWTLKAAAAAYFIGGVS	AAW	VSL	0.06551	28.86
89	102	14	YRMQEKWKVKAAYI	AYR	YIQ	0.06546	28.9
121	133	13	FVAAYLRWFFVFS	LFV	FST	0.06542	28.94
185	200	16	LNDLWGGPGPGFIAVY	VLN	VYY	0.06507	28.97
92	104	13	QEKWKVKAAYIQL	MQE	QLR	0.06495	29.01
102	114	13	IQLRWFFVFAAYS	YIQ	YSY	0.06492	29.05
226	242	17	KAKFVAAWTLKAAAKKT	KKA	KTG	0.06482	29.09
230	247	18	VAAWTLKAAAKKTGALLA	FVA	LAA	0.06479	29.13
144	157	14	VKLAAYRETTEAAI	KVK	AII	0.06476	29.17
131	146	16	VFSTAAYMQEKWKVKL	FVF	KLA	0.06474	29.21
157	173	17	IIAAYIGAAFIAVYGPG	AII	PGP	0.06466	29.25
198	210	13	AVYYTVLNDLWGN	IAV	GNK	0.0646	29.28
154	170	17	EAAIIAAYIGAAFIAVY	TEA	VYG	0.0646	29.32
206	221	16	DLWGNKKKTERMQEKW	NDL	KWK	0.06453	29.36
77	89	13	YFIGGVSLGIAAY	AYF	AYR	0.06439	29.4
107	122	16	FFVFAAYSYCLYWLFV	WFF	FVA	0.06433	29.44
199	214	16	VYYTVLNDLWGNKKKT	AVY	KTE	0.06425	29.48
222	235	14	KVKKKAKFVAAWTL	WKV	TLK	0.06419	29.52
146	163	18	LAAYRETTEAAIIAAYIG	KLA	IGA	0.06401	29.56
182	197	16	YTVLNDLWGGPGPGFI	YYT	FIA	0.06354	29.59
164	181	18	AAFIAVYGPGPGAFIAVY	GAA	VYY	0.06349	29.63
137	151	15	YMQEKWKVKLAAYRE	AYM	RET	0.06334	29.67
213	229	17	KTERMQEKWKVKKKAKF	KKT	KFV	0.06328	29.71
216	232	17	RMQEKWKVKKKAKFVAA	ERM	AAW	0.06316	29.75
142	154	13	WKVKLAAYRETTE	KWK	TEA	0.06305	29.79
107	121	15	FFVFAAYSYCLYWLF	WFF	LFV	0.06298	29.83
79	98	20	IGGVSLGIAAYRMQEKWKVK	FIG	VKA	0.06297	29.86
75	92	18	AAYFIGGVSLGIAAYRMQ	AAA	MQE	0.06294	29.9
200	215	16	YYTVLNDLWGNKKKTE	VYY	TER	0.06263	29.94
16	31	16	TLQKYYCRVRGGRCAV	NTL	AVL	0.06252	29.98
199	215	17	VYYTVLNDLWGNKKKTE	AVY	TER	0.06205	30.02
93	111	19	EKWKVKAAYIQLRWFFVFA	QEK	FAA	0.06193	30.06
147	161	15	AAYRETTEAAIIAAY	LAA	AYI	0.06193	30.1
9	26	18	AAKGIINTLQKYYCRVRG	AAA	RGG	0.06174	30.14

117	131	15	LYWLFVAAYLRWFFV	CLY	FVF	0.0616	30.17
197	216	20	IAVYYTVLNDLWGNKKKTER	FIA	ERM	0.06157	30.21
122	137	16	VAAYLRWFFVFSTAAY	FVA	AYM	0.06152	30.25
68	80	13	WTLKAAAAAYFIG	AWT	IGG	0.06133	30.29
127	145	19	RWFFVFSTAAYMQEKWKVK	LRW	VKL	0.06131	30.33
31	47	17	VLSCLPKEEQIGKCSTR	AVL	TRG	0.0609	30.37
221	237	17	WKVKKKAKFVAAWTLKA	KWK	KAA	0.06071	30.41
189	205	17	WGGPGPGFIAVYYTVLN	LWG	LND	0.06063	30.44
190	203	14	GGPGPGFIAVYYTV	WGG	TVL	0.06054	30.48
49	65	17	RKCCRRKKEAAAKAKFV	GRK	FVA	0.06051	30.52
134	151	18	TAAYMQEKWKVKLAAYRE	STA	RET	0.06037	30.56
76	92	17	AYFIGGVSLGIAAYRMQ	AAY	MQE	0.0603	30.6
123	139	17	AAYLRWFFVFSTAAYMQ	VAA	MQE	0.0603	30.6
82	99	18	VSLGIAAYRMQEKWKVKA	GVS	KAA	0.0603	30.68
204	219	16	LNDLWGNKKKTERMQE	VLN	QEK	0.06027	30.72
112	125	14	AYSYCLYWLFVAAY	AAY	AYL	0.06024	30.75
31	43	13	VLSCLPKEEQIGK	AVL	GKC	0.06017	30.79
143	157	15	KVKLAAYRETTEAAI	WKV	AII	0.0599	30.83
82	95	14	VSLGIAAYRMQEKW	GVS	KWK	0.05986	30.87
71	88	18	KAAAAAYFIGGVSLGIAA	LKA	AAY	0.05984	30.91
182	196	15	YTVLNDLWGGPGPGF	YYT	GFI	0.05981	30.95
2	16	15	PPHALEAAAKGIINT	APP	NTL	0.05981	30.99
170	183	14	YGPGPGAFIAVYYT	VYG	YTV	0.05966	31.03
100	114	15	AYIQLRWFFVFAAYS	AAY	YSY	0.0596	31.06
88	105	18	AYRMQEKWKVKAAYIQLR	AAY	LRW	0.05959	31.1
11	27	17	KGIINTLQKYYCRVRGG	AKG	GGR	0.05959	31.14
177	193	17	FIAVYYTVLNDLWGGPG	AFI	PGP	0.05955	31.18
105	120	16	RWFFVFAAYSYCLYWL	LRW	WLF	0.05937	31.22
224	241	18	KKKAKFVAAWTLKAAAKK	VKK	KKT	0.0593	31.26
31	46	16	VLSCLPKEEQIGKCST	AVL	STR	0.05922	31.3
183	200	18	TVLNDLWGGPGPGFIAVY	YTV	VYY	0.05899	31.33
178	191	14	IAVYYTVLNDLWGG	FIA	GGP	0.05868	31.37
138	152	15	MQEKWKVKLAAYRET	YMQ	ETT	0.05863	31.41
219	235	17	EKWKVKKKAKFVAAWTL	QEK	TLK	0.05863	31.45
215	227	13	ERMQEKWKVKKKA	TER	KAK	0.05862	31.49
202	214	13	TVLNDLWGNKKKT	YTV	KTE	0.05846	31.53
60	77	18	AKAKFVAAWTLKAAAAAY	AAK	AYF	0.05841	31.57
199	211	13	VYYTVLNDLWGNK	AVY	NKK	0.05839	31.61
88	104	17	AYRMQEKWKVKAAYIQL	AAY	QLR	0.05833	31.64
108	122	15	FVFAAYSYCLYWLFV	FFV	FVA	0.05816	31.68
89	101	13	YRMQEKWKVKAAY	AYR	AYI	0.05803	31.72
149	161	13	YRETTEAAIIAAY	AYR	AYI	0.05803	31.72
18	33	16	QKYYCRVRGGRCAVLS	LQK	LSC	0.05795	31.8
54	71	18	RKKEAAAKAKFVAAWTLK	RRK	LKA	0.05782	31.84
93	110	18	EKWKVKAAYIQLRWFFVF	QEK	VFA	0.05738	31.88
160	172	13	AYIGAAFIAVYGP	AAY	GPG	0.05734	31.91
180	192	13	VYYTVLNDLWGGP	AVY	GPG	0.05734	31.91
221	233	13	WKVKKKAKFVAAW	KWK	AWT	0.05734	31.91
5	18	14	ALEAAAKGIINTLQ	HAL	LQK	0.05727	32.03

226	243	18	KAKFVAAWTLKAAAKKTG	KKA	TGA	0.05726	32.07
11	28	18	KGIINTLQKYYCRVRGGR	AKG	GRC	0.05697	32.11
222	239	18	KVKKKAKFVAAWTLKAAA	WKV	AAK	0.05676	32.15
161	177	17	YIGAAFIAVYGPGPGAF	AYI	AFI	0.05674	32.19
27	41	15	GRCAVLSCLPKEEQI	GGR	QIG	0.05649	32.22
186	201	16	NDLWGGPGPGFIAVYY	LND	YYT	0.05649	32.26
232	246	15	AWTLKAAAKKTGALL	AAW	LLA	0.05635	32.3
191	208	18	GPGPGFIAVYYTVLNDLW	GGP	LWG	0.05624	32.34
198	216	19	AVYYTVLNDLWGNKKKTER	IAV	ERM	0.05623	32.38
231	246	16	AAWTLKAAAKKTGALL	VAA	LLA	0.05622	32.42
20	36	17	YYCRVRGGRCAVLSCLP	KYY	LPK	0.05618	32.46
181	194	14	YYTVLNDLWGGPGP	VYY	GPG	0.05614	32.5
143	160	18	KVKLAAYRETTEAAIIAA	WKV	AAY	0.0561	32.53
72	89	18	AAAAAYFIGGVSLGIAAY	KAA	AYR	0.05599	32.57
57	69	13	EAAAKAKFVAAWT	KEA	WTL	0.05597	32.61
71	84	14	KAAAAAYFIGGVSL	LKA	SLG	0.05587	32.65
203	220	18	VLNDLWGNKKKTERMQEK	TVL	EKW	0.0557	32.69
195	210	16	GFIAVYYTVLNDLWGN	PGF	GNK	0.05569	32.73
204	218	15	LNDLWGNKKKTERMQ	VLN	MQE	0.05561	32.77
234	251	18	TLKAAAKKTGALLAAGAA	WTL	AAA	0.05544	32.8
85	103	19	GIAAYRMQEKWKVKAAYIQ	LGI	IQL	0.05534	32.84
170	185	16	YGPGPGAFIAVYYTVL	VYG	VLN	0.05527	32.88
232	248	17	AWTLKAAAKKTGALLAA	AAW	AAG	0.05467	32.92
202	219	18	TVLNDLWGNKKKTERMQE	YTV	QEK	0.05464	32.96
206	223	18	DLWGNKKKTERMQEKWKV	NDL	KVK	0.05459	33.0
19	31	13	KYYCRVRGGRCAV	QKY	AVL	0.05457	33.04
217	230	14	MQEKWKVKKKAKFV	RMQ	FVA	0.05452	33.08
183	196	14	TVLNDLWGGPGPGF	YTV	GFI	0.05451	33.11
38	57	20	EEQIGKCSTRGRKCCRRKKE	KEE	KEA	0.05446	33.15
64	81	18	FVAAWTLKAAAAAYFIGG	KFV	GGV	0.05442	33.19
87	101	15	AAYRMQEKWKVKAAY	IAA	AYI	0.05438	33.23
155	167	13	AAIIAAYIGAAFI	EAA	FIA	0.05419	33.27
97	113	17	VKAAYIQLRWFFVFAAY	KVK	AYS	0.0541	33.31
38	56	19	EEQIGKCSTRGRKCCRRKK	KEE	KKE	0.05408	33.35
57	75	19	EAAAKAKFVAAWTLKAAAA	KEA	AAA	0.05388	33.38
83	97	15	SLGIAAYRMQEKWKV	VSL	KVK	0.05385	33.42
54	69	16	RKKEAAAKAKFVAAWT	RRK	WTL	0.0537	33.46
10	27	18	AKGIINTLQKYYCRVRGG	AAK	GGR	0.0537	33.5
156	173	18	AIIAAYIGAAFIAVYGPG	AAI	PGP	0.0537	33.5
10	28	19	AKGIINTLQKYYCRVRGGR	AAK	GRC	0.05365	33.58
215	230	16	ERMQEKWKVKKKAKFV	TER	FVA	0.05357	33.62
48	66	19	GRKCCRRKKEAAAKAKFVA	RGR	VAA	0.05347	33.66
161	176	16	YIGAAFIAVYGPGPGA	AYI	GAF	0.05345	33.69
109	122	14	VFAAYSYCLYWLFV	FVF	FVA	0.05338	33.73
128	145	18	WFFVFSTAAYMQEKWKVK	RWF	VKL	0.05323	33.77
132	146	15	FSTAAYMQEKWKVKL	VFS	KLA	0.05315	33.81
74	91	18	AAAYFIGGVSLGIAAYRM	AAA	RMQ	0.05314	33.85
101	113	13	YIQLRWFFVFAAY	AYI	AYS	0.05314	33.89
58	70	13	AAAKAKFVAAWTL	EAA	TLK	0.05293	33.93

223	235	13	VKKKAKFVAAWTL	KVK	TLK	0.05293	33.93
198	214	17	AVYYTVLNDLWGNKKKT	IAV	KTE	0.05293	34.0
134	146	13	TAAYMQEKWKVKL	STA	KLA	0.05287	34.04
233	246	14	WTLKAAAKKTGALL	AWT	LLA	0.05287	34.08
92	108	17	QEKWKVKAAYIQLRWFF	MQE	FFV	0.05285	34.12
124	138	15	AYLRWFFVFSTAAYM	AAY	YMQ	0.05279	34.16
221	235	15	WKVKKKAKFVAAWTL	KWK	TLK	0.05279	34.16
98	114	17	KAAYIQLRWFFVFAAYS	VKA	YSY	0.0527	34.24
188	200	13	LWGGPGPGFIAVY	DLW	VYY	0.05258	34.27
25	42	18	RGGRCAVLSCLPKEEQIG	VRG	IGK	0.05256	34.31
232	249	18	AWTLKAAAKKTGALLAAG	AAW	AGA	0.05256	34.31
165	180	16	AFIAVYGPGPGAFIAV	AAF	AVY	0.05254	34.39
7	20	14	EAAAKGIINTLQKY	LEA	KYY	0.05251	34.43
233	245	13	WTLKAAAKKTGAL	AWT	ALL	0.0525	34.47
176	195	20	AFIAVYYTVLNDLWGGPGPG	GAF	PGF	0.05243	34.51
152	167	16	TTEAAIIAAYIGAAFI	ETT	FIA	0.05243	34.55
94	111	18	KWKVKAAYIQLRWFFVFA	EKW	FAA	0.0522	34.58
217	233	17	MQEKWKVKKKAKFVAAW	RMQ	AWT	0.05219	34.62
151	167	17	ETTEAAIIAAYIGAAFI	RET	FIA	0.05218	34.66
137	150	14	YMQEKWKVKLAAYR	AYM	YRE	0.05204	34.7
163	177	15	GAAFIAVYGPGPGAF	IGA	AFI	0.05195	34.74
221	238	18	WKVKKKAKFVAAWTLKAA	KWK	AAA	0.0519	34.78
125	140	16	YLRWFFVFSTAAYMQE	AYL	QEK	0.05189	34.82
175	188	14	GAFIAVYYTVLNDL	PGA	DLW	0.05177	34.85
123	137	15	AAYLRWFFVFSTAAY	VAA	AYM	0.05177	34.89
128	140	13	WFFVFSTAAYMQE	RWF	QEK	0.05176	34.93
72	84	13	AAAAAYFIGGVSL	KAA	SLG	0.05173	34.97
191	211	21	GPGPGFIAVYYTVLNDLWGNK	GGP	NKK	0.05167	35.01
228	244	17	KFVAAWTLKAAAKKTGA	AKF	GAL	0.05158	35.05
197	214	18	IAVYYTVLNDLWGNKKKT	FIA	KTE	0.05156	35.09
107	120	14	FFVFAAYSYCLYWL	WFF	WLF	0.05146	35.13
157	169	13	IIAAYIGAAFIAV	AII	AVY	0.05132	35.16
66	78	13	AAWTLKAAAAAYF	VAA	YFI	0.05126	35.2
32	47	16	LSCLPKEEQIGKCSTR	VLS	TRG	0.05126	35.24
99	115	17	AAYIQLRWFFVFAAYSY	KAA	SYC	0.05122	35.28
190	207	18	GGPGPGFIAVYYTVLNDL	WGG	DLW	0.05115	35.32
100	113	14	AYIQLRWFFVFAAY	AAY	AYS	0.05108	35.36
155	172	18	AAIIAAYIGAAFIAVYGP	EAA	GPG	0.05085	35.4
223	241	19	VKKKAKFVAAWTLKAAAKK	KVK	KKT	0.0508	35.44
89	106	18	YRMQEKWKVKAAYIQLRW	AYR	RWF	0.05063	35.47
94	110	17	KWKVKAAYIQLRWFFVF	EKW	VFA	0.05054	35.51
11	24	14	KGIINTLQKYYCRV	AKG	RVR	0.05047	35.55
8	20	13	AAAKGIINTLQKY	EAA	KYY	0.05038	35.59
80	98	19	GGVSLGIAAYRMQEKWKVK	IGG	VKA	0.05033	35.63
152	164	13	TTEAAIIAAYIGA	ETT	GAA	0.0503	35.67
162	181	20	IGAAFIAVYGPGPGAFIAVY	YIG	VYY	0.0503	35.71
105	119	15	RWFFVFAAYSYCLYW	LRW	YWL	0.05027	35.74
118	130	13	YWLFVAAYLRWFF	LYW	FFV	0.05023	35.78
117	130	14	LYWLFVAAYLRWFF	CLY	FFV	0.05012	35.82

164	180	17	AAFIAVYGPGPGAFIAV	GAA	AVY	0.04985	35.86
230	246	17	VAAWTLKAAAKKTGALL	FVA	LLA	0.0498	35.9
132	148	17	FSTAAYMQEKWKVKLAA	VFS	AAY	0.04967	35.94
175	190	16	GAFIAVYYTVLNDLWG	PGA	WGG	0.04942	35.98
188	202	15	LWGGPGPGFIAVYYT	DLW	YTV	0.04939	36.02
112	128	17	AYSYCLYWLFVAAYLRW	AAY	RWF	0.04935	36.05
165	178	14	AFIAVYGPGPGAFI	AAF	FIA	0.04895	36.09
24	42	19	VRGGRCAVLSCLPKEEQIG	RVR	IGK	0.04887	36.13
41	61	21	IGKCSTRGRKCCRRKKEAAAK	QIG	AKA	0.04873	36.17
207	219	13	LWGNKKKTERMQE	DLW	QEK	0.04871	36.21
114	126	13	SYCLYWLFVAAYL	YSY	YLR	0.04859	36.25
55	69	15	KKEAAAKAKFVAAWT	RKK	WTL	0.04845	36.29
77	94	18	YFIGGVSLGIAAYRMQEK	AYF	EKW	0.04844	36.32
90	106	17	RMQEKWKVKAAYIQLRW	YRM	RWF	0.04843	36.36
16	32	17	TLQKYYCRVRGGRCAVL	NTL	VLS	0.04839	36.4
8	21	14	AAAKGIINTLQKYY	EAA	YYC	0.04836	36.44
200	214	15	YYTVLNDLWGNKKKT	VYY	KTE	0.04834	36.48
220	234	15	KWKVKKKAKFVAAWT	EKW	WTL	0.04832	36.52
234	250	17	TLKAAAKKTGALLAAGA	WTL	GAA	0.04825	36.56
79	95	17	IGGVSLGIAAYRMQEKW	FIG	KWK	0.04814	36.6
7	25	19	EAAAKGIINTLQKYYCRVR	LEA	VRG	0.04808	36.63
193	210	18	GPGFIAVYYYTVLNDLWGN	PGP	GNK	0.04807	36.67
125	139	15	YLRWFFVFSTAAYMQ	AYL	MQE	0.04787	36.71
94	106	13	KWKVKAAYIQLRW	EKW	RWF	0.04785	36.75
123	138	16	AAYLRWFFVFSTAAYM	VAA	YMQ	0.04776	36.79
88	101	14	AYRMQEKWKVKAAY	AAY	AYI	0.04736	36.83
148	161	14	AYRETTEAAIIAAY	AAY	AYI	0.04736	36.83
71	90	20	KAAAAAYFIGGVSLGIAAYR	LKA	YRM	0.04735	36.91
117	133	17	LYWLFVAAYLRWFFVFS	CLY	FST	0.04728	36.94
107	124	18	FFVFAAYSYCLYWLFVAA	WFF	AAY	0.04726	36.98
124	137	14	AYLRWFFVFSTAAY	AAY	AYM	0.04718	37.02
221	234	14	WKVKKKAKFVAAWT	KWK	WTL	0.04718	37.02
47	64	18	RGRKCCRRKKEAAAKAKF	TRG	KFV	0.04715	37.1
41	60	20	IGKCSTRGRKCCRRKKEAAA	QIG	AAK	0.04713	37.14
65	79	15	VAAWTLKAAAAAYFI	FVA	FIG	0.04706	37.18
168	182	15	AVYGPGPGAFIAVYY	IAV	YYT	0.04702	37.21
55	67	13	KKEAAAKAKFVAA	RKK	AAW	0.047	37.25
108	121	14	FVFAAYSYCLYWLF	FFV	LFV	0.04694	37.29
161	179	19	YIGAAFIAVYGPGPGAFIA	AYI	IAV	0.04689	37.33
220	232	13	KWKVKKKAKFVAA	EKW	AAW	0.04688	37.37
19	36	18	KYYCRVRGGRCAVLSCLP	QKY	LPK	0.04682	37.41
187	204	18	DLWGGPGPGFIAVYYTVL	NDL	VLN	0.04673	37.45
170	186	17	YGPGPGAFIAVYYTVLN	VYG	LND	0.04658	37.49
158	170	13	IAAYIGAAFIAVY	IIA	VYG	0.04657	37.52
232	244	13	AWTLKAAAKKTGA	AAW	GAL	0.04641	37.56
225	242	18	KKAKFVAAWTLKAAAKKT	KKK	KTG	0.04636	37.6
183	199	17	TVLNDLWGGPGPGFIAV	YTV	AVY	0.04632	37.64
173	190	18	GPGAFIAVYYTVLNDLWG	PGP	WGG	0.04623	37.68
122	139	18	VAAYLRWFFVFSTAAYMQ	FVA	MQE	0.0461	37.72

75	93	19	AAYFIGGVSLGIAAYRMQE	AAA	QEK	0.04587	37.76
15	27	13	NTLQKYYCRVRGG	INT	GGR	0.0458	37.79
119	133	15	WLFVAAYLRWFFVFS	YWL	FST	0.04579	37.83
78	96	19	FIGGVSLGIAAYRMQEKWK	YFI	WKV	0.04571	37.87
201	218	18	YTVLNDLWGNKKKTERMQ	YYT	MQE	0.0456	37.91
153	169	17	TEAAIIAAYIGAAFIAV	TTE	AVY	0.04553	37.95
127	142	16	RWFFVFSTAAYMQEKW	LRW	KWK	0.04547	37.99
156	171	16	AIIAAYIGAAFIAVYG	AAI	YGP	0.04541	38.03
227	244	18	AKFVAAWTLKAAAKKTGA	KAK	GAL	0.0454	38.07
130	147	18	FVFSTAAYMQEKWKVKLA	FFV	LAA	0.04533	38.1
114	130	17	SYCLYWLFVAAYLRWFF	YSY	FFV	0.0453	38.14
126	143	18	LRWFFVFSTAAYMQEKWK	YLR	WKV	0.04524	38.18
168	180	13	AVYGPGPGAFIAV	IAV	AVY	0.04523	38.22
113	125	13	YSYCLYWLFVAAY	AYS	AYL	0.0452	38.26
148	164	17	AYRETTEAAIIAAYIGA	AAY	GAA	0.04517	38.3
18	32	15	QKYYCRVRGGRCAVL	LQK	VLS	0.04515	38.34
64	79	16	FVAAWTLKAAAAAYFI	KFV	FIG	0.045	38.38
197	215	19	IAVYYTVLNDLWGNKKKTE	FIA	TER	0.04491	38.41
55	70	16	KKEAAAKAKFVAAWTL	RKK	TLK	0.04469	38.45
105	121	17	RWFFVFAAYSYCLYWLF	LRW	LFV	0.04465	38.49
12	24	13	GIINTLQKYYCRV	KGI	RVR	0.04458	38.53
220	235	16	KWKVKKKAKFVAAWTL	EKW	TLK	0.04458	38.57
157	174	18	IIAAYIGAAFIAVYGPGP	AII	GPG	0.04454	38.61
141	153	13	KWKVKLAAYRETT	EKW	TTE	0.04442	38.65
30	46	17	AVLSCLPKEEQIGKCST	CAV	STR	0.04441	38.68
85	101	17	GIAAYRMQEKWKVKAAY	LGI	AYI	0.0444	38.72
56	75	20	KEAAAKAKFVAAWTLKAAAA	KKE	AAA	0.0444	38.76
217	229	13	MQEKWKVKKKAKF	RMQ	KFV	0.04435	38.8
31	48	18	VLSCLPKEEQIGKCSTRG	AVL	RGR	0.04431	38.84
198	215	18	AVYYTVLNDLWGNKKKTE	IAV	TER	0.04412	38.88
137	152	16	YMQEKWKVKLAAYRET	AYM	ETT	0.04403	38.92
213	231	19	KTERMQEKWKVKKKAKFVA	KKT	VAA	0.04389	38.96
222	234	13	KVKKKAKFVAAWT	WKV	WTL	0.04375	38.99
20	32	13	YYCRVRGGRCAVL	KYY	VLS	0.04374	39.03
166	178	13	FIAVYGPGPGAFI	AFI	FIA	0.04371	39.07
82	100	19	VSLGIAAYRMQEKWKVKAA	GVS	AAY	0.04365	39.11
138	150	13	MQEKWKVKLAAYR	YMQ	YRE	0.04357	39.15
144	163	20	VKLAAYRETTEAAIIAAYIG	KVK	IGA	0.04355	39.19
187	203	17	DLWGGPGPGFIAVYYTV	NDL	TVL	0.04351	39.23
146	161	16	LAAYRETTEAAIIAAY	KLA	AYI	0.04351	39.26
23	40	18	RVRGGRCAVLSCLPKEEQ	CRV	EQI	0.04335	39.3
130	146	17	FVFSTAAYMQEKWKVKL	FFV	KLA	0.04334	39.34
169	185	17	VYGPGPGAFIAVYYTVL	AVY	VLN	0.04332	39.38
32	48	17	LSCLPKEEQIGKCSTRG	VLS	RGR	0.04321	39.42
210	228	19	NKKKTERMQEKWKVKKKAK	GNK	AKF	0.04318	39.46
6	21	16	LEAAAKGIINTLQKYY	ALE	YYC	0.04316	39.5
189	203	15	WGGPGPGFIAVYYTV	LWG	TVL	0.04309	39.54
218	230	13	QEKWKVKKKAKFV	MQE	FVA	0.04298	39.57
70	84	15	LKAAAAAYFIGGVSL	TLK	SLG	0.04281	39.61

225	243	19	KKAKFVAAWTLKAAAKKTG	KKK	TGA	0.04279	39.65
204	217	14	LNDLWGNKKKTERM	VLN	RMQ	0.04278	39.69
200	212	13	YYTVLNDLWGNKK	VYY	KKK	0.04276	39.73
86	102	17	IAAYRMQEKWKVKAAYI	GIA	YIQ	0.04274	39.77
122	138	17	VAAYLRWFFVFSTAAYM	FVA	YMQ	0.0423	39.81
235	252	18	LKAAAKKTGALLAAGAAA	TLK	AAK	0.04228	39.85
61	78	18	KAKFVAAWTLKAAAAAYF	AKA	YFI	0.04214	39.88
2	17	16	PPHALEAAAKGIINTL	APP	TLQ	0.04206	39.92
66	83	18	AAWTLKAAAAAYFIGGVS	VAA	VSL	0.04205	39.96
76	93	18	AYFIGGVSLGIAAYRMQE	AAY	QEK	0.04205	39.96
95	112	18	WKVKAAYIQLRWFFVFAA	KWK	AAY	0.04205	39.96
123	140	18	AAYLRWFFVFSTAAYMQE	VAA	QEK	0.04205	39.96
234	246	13	TLKAAAKKTGALL	WTL	LLA	0.04205	40.12
216	233	18	RMQEKWKVKKKAKFVAAW	ERM	AWT	0.04197	40.15
209	221	13	GNKKKTERMQEKW	WGN	KWK	0.04192	40.19
8	24	17	AAAKGIINTLQKYYCRV	EAA	RVR	0.04186	40.23
210	227	18	NKKKTERMQEKWKVKKKA	GNK	KAK	0.04182	40.27
92	107	16	QEKWKVKAAYIQLRWF	MQE	WFF	0.04178	40.31
119	132	14	WLFVAAYLRWFFVF	YWL	VFS	0.04173	40.35
229	247	19	FVAAWTLKAAAKKTGALLA	KFV	LAA	0.04165	40.39
69	84	16	TLKAAAAAYFIGGVSL	WTL	SLG	0.04162	40.43
195	211	17	GFIAVYYYTVLNDLWGNK	PGF	NKK	0.04146	40.46
212	229	18	KKTERMQEKWKVKKKAKF	KKK	KFV	0.04126	40.5
110	125	16	FAAYSYCLYWLFVAAY	VFA	AYL	0.0411	40.54
177	194	18	FIAVYYYTVLNDLWGGPGP	AFI	GPG	0.04101	40.58
184	196	13	VLNDLWGGPGPGF	TVL	GFI	0.04096	40.62
110	127	18	FAAYSYCLYWLFVAAYLR	VFA	LRW	0.04096	40.66
105	123	19	RWFFVFAAYSYCLYWLFVA	LRW	VAA	0.04092	40.7
163	178	16	GAAFIAVYGPGPGAFI	IGA	FIA	0.04087	40.74
119	134	16	WLFVAAYLRWFFVFST	YWL	STA	0.04086	40.77
86	105	20	IAAYRMQEKWKVKAAYIQLR	GIA	LRW	0.04077	40.81
209	228	20	GNKKKTERMQEKWKVKKKAK	WGN	AKF	0.04076	40.85
88	102	15	AYRMQEKWKVKAAYI	AAY	YIQ	0.04075	40.89
142	157	16	WKVKLAAYRETTEAAI	KWK	AII	0.04061	40.93
58	76	19	AAAKAKFVAAWTLKAAAAA	EAA	AAY	0.04048	40.97
15	31	17	NTLQKYYCRVRGGRCAV	INT	AVL	0.04032	41.01
177	191	15	FIAVYYYTVLNDLWGG	AFI	GGP	0.04019	41.04
100	115	16	AYIQLRWFFVFAAYSY	AAY	SYC	0.04007	41.08
16	33	18	TLQKYYCRVRGGRCAVLS	NTL	LSC	0.03997	41.12
124	141	18	AYLRWFFVFSTAAYMQEK	AAY	EKW	0.03965	41.16
155	175	21	AAIIAAYIGAAFIAVYGPGPG	EAA	PGA	0.03956	41.2
208	225	18	WGNKKKTERMQEKWKVKK	LWG	KKK	0.03942	41.24
30	47	18	AVLSCLPKEEQIGKCSTR	CAV	TRG	0.03942	41.28
129	142	14	FFVFSTAAYMQEKW	WFF	KWK	0.03941	41.32
83	99	17	SLGIAAYRMQEKWKVKA	VSL	KAA	0.0394	41.35
101	118	18	YIQLRWFFVFAAYSYCLY	AYI	LYW	0.03928	41.39
208	221	14	WGNKKKTERMQEKW	LWG	KWK	0.03927	41.43
118	135	18	YWLFVAAYLRWFFVFSTA	LYW	TAA	0.03914	41.47
165	177	13	AFIAVYGPGPGAF	AAF	AFI	0.03912	41.51

219	237	19	EKWKVKKKAKFVAAWTLKA	QEK	KAA	0.03912	41.55
6	25	20	LEAAAKGIINTLQKYYCRVR	ALE	VRG	0.03903	41.59
195	207	13	GFIAVYYTVLNDL	PGF	DLW	0.039	41.62
81	99	19	GVSLGIAAYRMQEKWKVKA	GGV	KAA	0.03894	41.66
131	148	18	VFSTAAYMQEKWKVKLAA	FVF	AAY	0.03892	41.7
149	166	18	YRETTEAAIIAAYIGAAF	AYR	AFI	0.03889	41.74
207	224	18	LWGNKKKTERMQEKWKVK	DLW	VKK	0.03867	41.78
42	61	20	GKCSTRGRKCCRRKKEAAAK	IGK	AKA	0.03865	41.82
156	174	19	AIIAAYIGAAFIAVYGPGP	AAI	GPG	0.03865	41.86
87	102	16	AAYRMQEKWKVKAAYI	IAA	YIQ	0.03857	41.9
63	80	18	KFVAAWTLKAAAAAYFIG	AKF	IGG	0.03852	41.93
127	144	18	RWFFVFSTAAYMQEKWKV	LRW	KVK	0.03847	41.97
205	224	20	NDLWGNKKKTERMQEKWKVK	LND	VKK	0.03846	42.01
68	85	18	WTLKAAAAAYFIGGVSLG	AWT	LGI	0.03846	42.05
7	21	15	EAAAKGIINTLQKYY	LEA	YYC	0.03845	42.09
208	226	19	WGNKKKTERMQEKWKVKKK	LWG	KKA	0.03834	42.13
48	65	18	GRKCCRRKKEAAAKAKFV	RGR	FVA	0.03834	42.17
204	220	17	LNDLWGNKKKTERMQEK	VLN	EKW	0.03831	42.21
74	92	19	AAAYFIGGVSLGIAAYRMQ	AAA	MQE	0.03828	42.24
9	21	13	AAKGIINTLQKYY	AAA	YYC	0.03809	42.28
205	221	17	NDLWGNKKKTERMQEKW	LND	KWK	0.03809	42.32
176	191	16	AFIAVYYTVLNDLWGG	GAF	GGP	0.03799	42.36
87	105	19	AAYRMQEKWKVKAAYIQLR	IAA	LRW	0.03792	42.4
53	65	13	RRKKEAAAKAKFV	CRR	FVA	0.0379	42.44
111	128	18	AAYSYCLYWLFVAAYLRW	FAA	RWF	0.03773	42.48
222	240	19	KVKKKAKFVAAWTLKAAAK	WKV	AKK	0.03768	42.51
219	238	20	EKWKVKKKAKFVAAWTLKAA	QEK	AAA	0.0376	42.55
72	86	15	AAAAAYFIGGVSLGI	KAA	GIA	0.03749	42.59
37	49	13	KEEQIGKCSTRGR	PKE	GRK	0.03733	42.63
80	95	16	GGVSLGIAAYRMQEKW	IGG	KWK	0.03733	42.67
109	125	17	VFAAYSYCLYWLFVAAY	FVF	AYL	0.03732	42.71
193	208	16	GPGFIAVYYTVLNDLW	PGP	LWG	0.03725	42.75
32	46	15	LSCLPKEEQIGKCST	VLS	STR	0.03715	42.79
54	70	17	RKKEAAAKAKFVAAWTL	RRK	TLK	0.03692	42.82
47	66	20	RGRKCCRRKKEAAAKAKFVA	TRG	VAA	0.03675	42.86
79	97	19	IGGVSLGIAAYRMQEKWKV	FIG	KVK	0.03673	42.9
209	227	19	GNKKKTERMQEKWKVKKKA	WGN	KAK	0.0367	42.94
94	107	14	KWKVKAAYIQLRWF	EKW	WFF	0.03664	42.98
42	60	19	GKCSTRGRKCCRRKKEAAA	IGK	AAK	0.03635	43.02
45	63	19	STRGRKCCRRKKEAAAKAK	CST	AKF	0.03623	43.06
176	193	18	AFIAVYYTVLNDLWGGPG	GAF	PGP	0.03622	43.09
132	144	13	FSTAAYMQEKWKV	VFS	KVK	0.03613	43.13
149	168	20	YRETTEAAIIAAYIGAAFIA	AYR	IAV	0.03612	43.17
182	200	19	YTVLNDLWGGPGPGFIAVY	YYT	VYY	0.03588	43.21
18	31	14	QKYYCRVRGGRCAV	LQK	AVL	0.03584	43.25
114	129	16	SYCLYWLFVAAYLRWF	YSY	WFF	0.03581	43.29
10	23	14	AKGIINTLQKYYCR	AAK	CRV	0.03565	43.33
17	37	21	LQKYYCRVRGGRCAVLSCLPK	TLQ	PKE	0.03557	43.37
40	58	19	QIGKCSTRGRKCCRRKKEA	EQI	EAA	0.03553	43.4

187	205	19	DLWGGPGPGFIAVYYTVLN	NDL	LND	0.03553	43.44
106	123	18	WFFVFAAYSYCLYWLFVA	RWF	VAA	0.03552	43.48
87	104	18	AAYRMQEKWKVKAAYIQL	IAA	QLR	0.03552	43.52
86	104	19	IAAYRMQEKWKVKAAYIQL	GIA	QLR	0.0355	43.56
150	166	17	RETTEAAIIAAYIGAAF	YRE	AFI	0.03548	43.6
9	23	15	AAKGIINTLQKYYCR	AAA	CRV	0.03547	43.64
62	80	19	AKFVAAWTLKAAAAAYFIG	KAK	IGG	0.03544	43.68
154	172	19	EAAIIAAYIGAAFIAVYGP	TEA	GPG	0.03541	43.71
231	249	19	AAWTLKAAAKKTGALLAAG	VAA	AGA	0.03526	43.75
142	159	18	WKVKLAAYRETTEAAIIA	KWK	IAA	0.0352	43.79
157	171	15	IIAAYIGAAFIAVYG	AII	YGP	0.03518	43.83
162	180	19	IGAAFIAVYGPGPGAFIAV	YIG	AVY	0.03513	43.87
45	62	18	STRGRKCCRRKKEAAAKA	CST	KAK	0.0351	43.91
231	248	18	AAWTLKAAAKKTGALLAA	VAA	AAG	0.0351	43.91
169	182	14	VYGPGPGAFIAVYY	AVY	YYT	0.03507	43.98
181	198	18	YYTVLNDLWGGPGPGFIA	VYY	IAV	0.03505	44.02
110	126	17	FAAYSYCLYWLFVAAYL	VFA	YLR	0.03499	44.06
36	50	15	PKEEQIGKCSTRGRK	LPK	RKC	0.03499	44.1
70	87	18	LKAAAAAYFIGGVSLGIA	TLK	IAA	0.03498	44.14
164	176	13	AAFIAVYGPGPGA	GAA	GAF	0.03497	44.18
106	121	16	WFFVFAAYSYCLYWLF	RWF	LFV	0.03496	44.22
128	144	17	WFFVFSTAAYMQEKWKV	RWF	KVK	0.0349	44.26
41	53	13	IGKCSTRGRKCCR	QIG	CRR	0.03489	44.29
169	184	16	VYGPGPGAFIAVYYTV	AVY	TVL	0.03481	44.33
145	158	14	KLAAYRETTEAAII	VKL	IIA	0.03476	44.37
106	120	15	WFFVFAAYSYCLYWL	RWF	WLF	0.03465	44.41
60	78	19	AKAKFVAAWTLKAAAAAYF	AAK	YFI	0.03455	44.45
166	183	18	FIAVYGPGPGAFIAVYYT	AFI	YTV	0.03448	44.49
92	109	18	QEKWKVKAAYIQLRWFFV	MQE	FVF	0.03445	44.53
171	189	19	GPGPGAFIAVYYTVLNDLW	YGP	LWG	0.0342	44.56
142	158	17	WKVKLAAYRETTEAAII	KWK	IIA	0.03418	44.6
148	165	18	AYRETTEAAIIAAYIGAA	AAY	AAF	0.03401	44.64
218	234	17	QEKWKVKKKAKFVAAWT	MQE	WTL	0.03385	44.68
133	152	20	STAAYMQEKWKVKLAAYRET	FST	ETT	0.03382	44.72
129	147	19	FFVFSTAAYMQEKWKVKLA	WFF	LAA	0.03371	44.76
65	83	19	VAAWTLKAAAAAYFIGGVS	FVA	VSL	0.0336	44.8
122	140	19	VAAYLRWFFVFSTAAYMQE	FVA	QEK	0.0336	44.8
109	127	19	VFAAYSYCLYWLFVAAYLR	FVF	LRW	0.03354	44.87
97	114	18	VKAAYIQLRWFFVFAAYS	KVK	YSY	0.03348	44.91
201	219	19	YTVLNDLWGNKKKTERMQE	YYT	QEK	0.03323	44.95
8	26	19	AAAKGIINTLQKYYCRVRG	EAA	RGG	0.03314	44.99
170	184	15	YGPGPGAFIAVYYTV	VYG	TVL	0.03311	45.03
55	72	18	KKEAAAKAKFVAAWTLKA	RKK	KAA	0.03306	45.07
125	141	17	YLRWFFVFSTAAYMQEK	AYL	EKW	0.03299	45.11
228	245	18	KFVAAWTLKAAAKKTGAL	AKF	ALL	0.03298	45.15
220	237	18	KWKVKKKAKFVAAWTLKA	EKW	KAA	0.03298	45.18
181	193	13	YYTVLNDLWGGPG	VYY	PGP	0.03297	45.22
138	153	16	MQEKWKVKLAAYRETT	YMQ	TTE	0.0329	45.26
218	236	19	QEKWKVKKKAKFVAAWTLK	MQE	LKA	0.03288	45.3

185	199	15	LNDLWGGPGPGFIAV	VLN	AVY	0.03287	45.34
109	121	13	VFAAYSYCLYWLF	FVF	LFV	0.03286	45.38
146	158	13	LAAYRETTEAAII	KLA	IIA	0.03286	45.38
207	223	17	LWGNKKKTERMQEKWKV	DLW	KVK	0.03284	45.45
191	213	23	GPGPGFIAVYYTVLNDLWGNKKK	GGP	KKT	0.03269	45.49
66	79	14	AAWTLKAAAAAYFI	VAA	FIG	0.03265	45.53
175	187	13	GAFIAVYYTVLND	PGA	NDL	0.03239	45.57
73	86	14	AAAAYFIGGVSLGI	AAA	GIA	0.03232	45.61
73	91	19	AAAAYFIGGVSLGIAAYRM	AAA	RMQ	0.03232	45.65
106	118	13	WFFVFAAYSYCLY	RWF	LYW	0.0323	45.69
193	211	19	GPGFIAVYYTVLNDLWGNK	PGP	NKK	0.03228	45.73
59	77	19	AAKAKFVAAWTLKAAAAAY	AAA	AYF	0.03221	45.76
212	231	20	KKTERMQEKWKVKKKAKFVA	KKK	VAA	0.03217	45.8
104	119	16	LRWFFVFAAYSYCLYW	QLR	YWL	0.03199	45.84
35	57	23	LPKEEQIGKCSTRGRKCCRRKKE	CLP	KEA	0.03196	45.88
191	212	22	GPGPGFIAVYYTVLNDLWGNKK	GGP	KKK	0.03183	45.92
9	28	20	AAKGIINTLQKYYCRVRGGR	AAA	GRC	0.03182	45.96
149	162	14	YRETTEAAIIAAYI	AYR	YIG	0.03177	46.0
80	97	18	GGVSLGIAAYRMQEKWKV	IGG	KVK	0.03158	46.03
169	186	18	VYGPGPGAFIAVYYTVLN	AVY	LND	0.03151	46.07
213	230	18	KTERMQEKWKVKKKAKFV	KKT	FVA	0.03147	46.11
167	185	19	IAVYGPGPGAFIAVYYTVL	FIA	VLN	0.03136	46.15
202	220	19	TVLNDLWGNKKKTERMQEK	YTV	EKW	0.03133	46.19
147	164	18	AAYRETTEAAIIAAYIGA	LAA	GAA	0.03133	46.23
180	198	19	VYYTVLNDLWGGPGPGFIA	AVY	IAV	0.03133	46.27
113	130	18	YSYCLYWLFVAAYLRWFF	AYS	FFV	0.03096	46.31
38	53	16	EEQIGKCSTRGRKCCR	KEE	CRR	0.03088	46.34
176	189	14	AFIAVYYTVLNDLW	GAF	LWG	0.03086	46.38
129	146	18	FFVFSTAAYMQEKWKVKL	WFF	KLA	0.03084	46.42
168	185	18	AVYGPGPGAFIAVYYTVL	IAV	VLN	0.0308	46.46
214	232	19	TERMQEKWKVKKKAKFVAA	KTE	AAW	0.03069	46.5
229	246	18	FVAAWTLKAAAKKTGALL	KFV	LLA	0.03063	46.54
211	229	19	KKKTERMQEKWKVKKKAKF	NKK	KFV	0.03062	46.58
93	112	20	EKWKVKAAYIQLRWFFVFAA	QEK	AAY	0.03046	46.62
227	245	19	AKFVAAWTLKAAAKKTGAL	KAK	ALL	0.03033	46.65
81	100	20	GVSLGIAAYRMQEKWKVKAA	GGV	AAY	0.03032	46.69
230	249	20	VAAWTLKAAAKKTGALLAAG	FVA	AGA	0.0303	46.73
35	56	22	LPKEEQIGKCSTRGRKCCRRKK	CLP	KKE	0.03029	46.77
203	222	20	VLNDLWGNKKKTERMQEKWK	TVL	WKV	0.03025	46.81
43	57	15	KCSTRGRKCCRRKKE	GKC	KEA	0.03025	46.85
2	14	13	PPHALEAAAKGII	APP	IIN	0.03009	46.89
163	176	14	GAAFIAVYGPGPGA	IGA	GAF	0.03007	46.92
74	93	20	AAAYFIGGVSLGIAAYRMQE	AAA	QEK	0.03	46.96
30	48	19	AVLSCLPKEEQIGKCSTRG	CAV	RGR	0.02998	47.0
166	182	17	FIAVYGPGPGAFIAVYY	AFI	YYT	0.02996	47.04
189	206	18	WGGPGPGFIAVYYTVLND	LWG	NDL	0.02986	47.08
53	69	17	RRKKEAAAKAKFVAAWT	CRR	WTL	0.02985	47.12
9	27	19	AAKGIINTLQKYYCRVRGG	AAA	GGR	0.02961	47.16
55	73	19	KKEAAAKAKFVAAWTLKAA	RKK	AAA	0.02954	47.2

114	131	18	SYCLYWLFVAAYLRWFFV	YSY	FVF	0.02953	47.23
220	238	19	KWKVKKKAKFVAAWTLKAA	EKW	AAA	0.02946	47.27
233	251	19	WTLKAAAKKTGALLAAGAA	AWT	AAA	0.02946	47.27
138	154	17	MQEKWKVKLAAYRETTE	YMQ	TEA	0.02939	47.35
105	122	18	RWFFVFAAYSYCLYWLFV	LRW	FVA	0.02934	47.39
150	168	19	RETTEAAIIAAYIGAAFIA	YRE	IAV	0.02932	47.43
108	120	13	FVFAAYSYCLYWL	FFV	WLF	0.02925	47.47
144	161	18	VKLAAYRETTEAAIIAAY	KVK	AYI	0.02919	47.5
158	171	14	IAAYIGAAFIAVYG	IIA	YGP	0.02909	47.54
205	223	19	NDLWGNKKKTERMQEKWKV	LND	KVK	0.02906	47.58
171	192	22	GPGPGAFIAVYYTVLNDLWGGP	YGP	GPG	0.02905	47.62
140	157	18	EKWKVKLAAYRETTEAAI	QEK	AII	0.02902	47.66
53	71	19	RRKKEAAAKAKFVAAWTLK	CRR	LKA	0.02899	47.7
39	58	20	EQIGKCSTRGRKCCRRKKEA	EEQ	EAA	0.02877	47.74
161	178	18	YIGAAFIAVYGPGPGAFI	AYI	FIA	0.02872	47.78
168	184	17	AVYGPGPGAFIAVYYTV	IAV	TVL	0.02868	47.81
145	163	19	KLAAYRETTEAAIIAAYIG	VKL	IGA	0.02862	47.85
121	137	17	FVAAYLRWFFVFSTAAY	LFV	AYM	0.02862	47.89
57	76	20	EAAAKAKFVAAWTLKAAAAA	KEA	AAY	0.02855	47.93
113	129	17	YSYCLYWLFVAAYLRWF	AYS	WFF	0.02836	47.97
181	196	16	YYTVLNDLWGGPGPGF	VYY	GFI	0.02834	48.01
8	23	16	AAAKGIINTLQKYYCR	EAA	CRV	0.02831	48.05
75	94	20	AAYFIGGVSLGIAAYRMQEK	AAA	EKW	0.02829	48.09
134	152	19	TAAYMQEKWKVKLAAYRET	STA	ETT	0.02822	48.12
46	64	19	TRGRKCCRRKKEAAAKAKF	STR	KFV	0.02818	48.16
104	120	17	LRWFFVFAAYSYCLYWL	QLR	WLF	0.02816	48.2
188	204	17	LWGGPGPGFIAVYYTVL	DLW	VLN	0.02811	48.24
180	196	17	VYYTVLNDLWGGPGPGF	AVY	GFI	0.02807	48.28
235	253	19	LKAAAKKTGALLAAGAAAK	TLK	AKK	0.02807	48.32
230	248	19	VAAWTLKAAAKKTGALLAA	FVA	AAG	0.02804	48.36
222	241	20	KVKKKAKFVAAWTLKAAAKK	WKV	KKT	0.02801	48.39
126	138	13	LRWFFVFSTAAYM	YLR	YMQ	0.02795	48.43
167	184	18	IAVYGPGPGAFIAVYYTV	FIA	TVL	0.02794	48.47
84	103	20	LGIAAYRMQEKWKVKAAYIQ	SLG	IQL	0.02789	48.51
135	153	19	AAYMQEKWKVKLAAYRETT	TAA	TTE	0.02768	48.55
234	252	19	TLKAAAKKTGALLAAGAAA	WTL	AAK	0.02763	48.59
110	122	13	FAAYSYCLYWLFV	VFA	FVA	0.02756	48.63
119	135	17	WLFVAAYLRWFFVFSTA	YWL	TAA	0.02755	48.67
109	126	18	VFAAYSYCLYWLFVAAYL	FVF	YLR	0.02742	48.7
39	53	15	EQIGKCSTRGRKCCR	EEQ	CRR	0.02741	48.74
107	119	13	FFVFAAYSYCLYW	WFF	YWL	0.0274	48.78
189	201	13	WGGPGPGFIAVYY	LWG	YYT	0.0273	48.82
83	100	18	SLGIAAYRMQEKWKVKAA	VSL	AAY	0.02729	48.86
95	107	13	WKVKAAYIQLRWF	KWK	WFF	0.02729	48.9
37	54	18	KEEQIGKCSTRGRKCCRR	PKE	RRK	0.02719	48.94
117	134	18	LYWLFVAAYLRWFFVFST	CLY	STA	0.02714	48.97
91	108	18	MQEKWKVKAAYIQLRWFF	RMQ	FFV	0.02712	49.01
78	98	21	FIGGVSLGIAAYRMQEKWKVK	YFI	VKA	0.02712	49.05
182	199	18	YTVLNDLWGGPGPGFIAV	YYT	AVY	0.02696	49.09

15	32	18	NTLQKYYCRVRGGRCAVL	INT	VLS	0.02694	49.13
49	67	19	RKCCRRKKEAAAKAKFVAA	GRK	AAW	0.02683	49.17
136	153	18	AYMQEKWKVKLAAYRETT	AAY	TTE	0.02667	49.21
178	198	21	IAVYYTVLNDLWGGPGPGFIA	FIA	IAV	0.02665	49.25
106	122	17	WFFVFAAYSYCLYWLFV	RWF	FVA	0.02662	49.28
14	31	18	INTLQKYYCRVRGGRCAV	IIN	AVL	0.02661	49.32
196	216	21	FIAVYYYTVLNDLWGNKKKTER	GFI	ERM	0.02658	49.36
128	142	15	WFFVFSTAAYMQEKW	RWF	KWK	0.02654	49.4
96	113	18	KVKAAYIQLRWFFVFAAY	WKV	AYS	0.02654	49.44
153	170	18	TEAAIIAAYIGAAFIAVY	TTE	VYG	0.0265	49.48
206	226	21	DLWGNKKKTERMQEKWKVKKK	NDL	KKA	0.02641	49.52
215	232	18	ERMQEKWKVKKKAKFVAA	TER	AAW	0.02634	49.56
77	96	20	YFIGGVSLGIAAYRMQEKWK	AYF	WKV	0.02631	49.59
155	173	19	AAIIAAYIGAAFIAVYGPG	EAA	PGP	0.02613	49.63
176	194	19	AFIAVYYTVLNDLWGGPGP	GAF	GPG	0.02607	49.67
195	212	18	GFIAVYYYTVLNDLWGNKK	PGF	KKK	0.02597	49.71
206	225	20	DLWGNKKKTERMQEKWKVKK	NDL	KKK	0.02596	49.75
154	175	22	EAAIIAAYIGAAFIAVYGPGPG	TEA	PGA	0.02593	49.79
221	239	19	WKVKKKAKFVAAWTLKAAA	KWK	AAK	0.02587	49.83
126	145	20	LRWFFVFSTAAYMQEKWKVK	YLR	VKL	0.02566	49.86
167	186	20	IAVYGPGPGAFIAVYYTVLN	FIA	LND	0.02564	49.9
4	17	14	HALEAAAKGIINTL	PHA	TLQ	0.0256	49.94
142	160	19	WKVKLAAYRETTEAAIIAA	KWK	AAY	0.02557	49.98
141	157	17	KWKVKLAAYRETTEAAI	EKW	AII	0.02556	50.02
71	89	19	KAAAAAYFIGGVSLGIAAY	LKA	AYR	0.02556	50.06
140	159	20	EKWKVKLAAYRETTEAAIIA	QEK	IAA	0.0255	50.1
71	86	16	KAAAAAYFIGGVSLGI	LKA	GIA	0.02546	50.14
70	88	19	LKAAAAAYFIGGVSLGIAA	TLK	AAY	0.02541	50.17
90	102	13	RMQEKWKVKAAYI	YRM	YIQ	0.02533	50.21
138	155	18	MQEKWKVKLAAYRETTEA	YMQ	EAA	0.02526	50.25
173	189	17	GPGAFIAVYYYTVLNDLW	PGP	LWG	0.02512	50.29
118	136	19	YWLFVAAYLRWFFVFSTAA	LYW	AAY	0.0251	50.33
73	92	20	AAAAYFIGGVSLGIAAYRMQ	AAA	MQE	0.02504	50.37
179	198	20	AVYYYTVLNDLWGGPGPGFIA	IAV	IAV	0.02504	50.41
207	221	15	LWGNKKKTERMQEKW	DLW	KWK	0.02498	50.44
211	231	21	KKKTERMQEKWKVKKKAKFVA	NKK	VAA	0.02496	50.48
63	81	19	KFVAAWTLKAAAAAYFIGG	AKF	GGV	0.02494	50.52
186	204	19	NDLWGGPGPGFIAVYYYTVL	LND	VLN	0.02488	50.56
91	107	17	MQEKWKVKAAYIQLRWF	RMQ	WFF	0.02484	50.6
62	81	20	AKFVAAWTLKAAAAAYFIGG	KAK	GGV	0.02468	50.64
147	165	19	AAYRETTEAAIIAAYIGAA	LAA	AAF	0.02465	50.68
54	72	19	RKKEAAAKAKFVAAWTLKA	RRK	KAA	0.02464	50.72
233	250	18	WTLKAAAKKTGALLAAGA	AWT	GAA	0.02454	50.75
68	84	17	WTLKAAAAAYFIGGVSL	AWT	SLG	0.02452	50.79
155	171	17	AAIIAAYIGAAFIAVYG	EAA	YGP	0.0245	50.83
13	31	19	IINTLQKYYCRVRGGRCAV	GII	AVL	0.0245	50.87
47	65	19	RGRKCCRRKKEAAAKAKFV	TRG	FVA	0.0245	50.91
179	191	13	AVYYTVLNDLWGG	IAV	GGP	0.02436	50.95
106	119	14	WFFVFAAYSYCLYW	RWF	YWL	0.02419	50.99

76	94	19	AYFIGGVSLGIAAYRMQEK	AAY	EKW	0.02411	51.03
123	141	19	AAYLRWFFVFSTAAYMQEK	VAA	EKW	0.02411	51.03
160	176	17	AYIGAAFIAVYGPGPGA	AAY	GAF	0.02408	51.1
135	154	20	AAYMQEKWKVKLAAYRETTE	TAA	TEA	0.02399	51.14
94	112	19	KWKVKAAYIQLRWFFVFAA	EKW	AAY	0.02387	51.18
92	111	20	QEKWKVKAAYIQLRWFFVFA	MQE	FAA	0.02386	51.22
54	73	20	RKKEAAAKAKFVAAWTLKAA	RRK	AAA	0.02368	51.26
195	213	19	GFIAVYYTVLNDLWGNKKK	PGF	KKT	0.02367	51.3
130	148	19	FVFSTAAYMQEKWKVKLAA	FFV	AAY	0.0235	51.33
102	119	18	IQLRWFFVFAAYSYCLYW	YIQ	YWL	0.02349	51.37
168	186	19	AVYGPGPGAFIAVYYTVLN	IAV	LND	0.02342	51.41
143	163	21	KVKLAAYRETTEAAIIAAYIG	WKV	IGA	0.02334	51.45
31	49	19	VLSCLPKEEQIGKCSTRGR	AVL	GRK	0.02333	51.49
125	138	14	YLRWFFVFSTAAYM	AYL	YMQ	0.02329	51.53
15	33	19	NTLQKYYCRVRGGRCAVLS	INT	LSC	0.02325	51.57
64	83	20	FVAAWTLKAAAAAYFIGGVS	KFV	VSL	0.02323	51.61
26	46	21	GGRCAVLSCLPKEEQIGKCST	RGG	STR	0.02322	51.64
40	59	20	QIGKCSTRGRKCCRRKKEAA	EQI	AAA	0.02312	51.68
184	202	19	VLNDLWGGPGPGFIAVYYT	TVL	YTV	0.02308	51.72
163	181	19	GAAFIAVYGPGPGAFIAVY	IGA	VYY	0.02308	51.76
119	131	13	WLFVAAYLRWFFV	YWL	FVF	0.02305	51.8
46	66	21	TRGRKCCRRKKEAAAKAKFVA	STR	VAA	0.02297	51.84
170	187	18	YGPGPGAFIAVYYTVLND	VYG	NDL	0.02294	51.88
214	233	20	TERMQEKWKVKKKAKFVAAW	KTE	AWT	0.02292	51.91
4	16	13	HALEAAAKGIINT	PHA	NTL	0.02289	51.95
56	76	21	KEAAAKAKFVAAWTLKAAAAA	KKE	AAY	0.02288	51.99
69	87	19	TLKAAAAAYFIGGVSLGIA	WTL	IAA	0.02287	52.03
63	79	17	KFVAAWTLKAAAAAYFI	AKF	FIG	0.02286	52.07
98	115	18	KAAYIQLRWFFVFAAYSY	VKA	SYC	0.0228	52.11
38	58	21	EEQIGKCSTRGRKCCRRKKEA	KEE	EAA	0.02279	52.15
188	201	14	LWGGPGPGFIAVYY	DLW	YYT	0.02276	52.19
193	212	20	GPGFIAVYYTVLNDLWGNKK	PGP	KKK	0.02273	52.22
67	85	19	AWTLKAAAAAYFIGGVSLG	AAW	LGI	0.02272	52.26
114	132	19	SYCLYWLFVAAYLRWFFVF	YSY	VFS	0.0226	52.3
188	203	16	LWGGPGPGFIAVYYTV	DLW	TVL	0.02259	52.34
135	155	21	AAYMQEKWKVKLAAYRETTEA	TAA	EAA	0.02253	52.38
25	41	17	RGGRCAVLSCLPKEEQI	VRG	QIG	0.02253	52.42
190	209	20	GGPGPGFIAVYYTVLNDLWG	WGG	WGN	0.02247	52.46
74	86	13	AAAYFIGGVSLGI	AAA	GIA	0.02247	52.5
181	197	17	YYTVLNDLWGGPGPGFI	VYY	FIA	0.02244	52.53
130	142	13	FVFSTAAYMQEKW	FFV	KWK	0.0224	52.57
72	91	20	AAAAAYFIGGVSLGIAAYRM	KAA	RMQ	0.02234	52.61
196	208	13	FIAVYYTVLNDLW	GFI	LWG	0.02233	52.65
226	244	19	KAKFVAAWTLKAAAKKTGA	KKA	GAL	0.02228	52.69
152	169	18	TTEAAIIAAYIGAAFIAV	ETT	AVY	0.02224	52.73
5	20	16	ALEAAAKGIINTLQKY	HAL	KYY	0.02219	52.77
186	203	18	NDLWGGPGPGFIAVYYTV	LND	TVL	0.02216	52.8
31	50	20	VLSCLPKEEQIGKCSTRGRK	AVL	RKC	0.02211	52.84
18	37	20	QKYYCRVRGGRCAVLSCLPK	LQK	PKE	0.0221	52.88

160	177	18	AYIGAAFIAVYGPGPGAF	AAY	AFI	0.02206	52.92
140	158	19	EKWKVKLAAYRETTEAAII	QEK	IIA	0.02203	52.96
36	49	14	PKEEQIGKCSTRGR	LPK	GRK	0.02201	53.0
175	192	18	GAFIAVYYTVLNDLWGGP	PGA	GPG	0.02187	53.04
105	124	20	RWFFVFAAYSYCLYWLFVAA	LRW	AAY	0.02185	53.08
108	127	20	FVFAAYSYCLYWLFVAAYLR	FFV	LRW	0.02179	53.11
32	49	18	LSCLPKEEQIGKCSTRGR	VLS	GRK	0.02177	53.15
193	213	21	GPGFIAVYYTVLNDLWGNKKK	PGP	KKT	0.02166	53.19
24	43	20	VRGGRCAVLSCLPKEEQIGK	RVR	GKC	0.02161	53.23
25	43	19	RGGRCAVLSCLPKEEQIGK	VRG	GKC	0.02161	53.27
145	161	17	KLAAYRETTEAAIIAAY	VKL	AYI	0.02157	53.31
108	125	18	FVFAAYSYCLYWLFVAAY	FFV	AYL	0.02156	53.35
124	143	20	AYLRWFFVFSTAAYMQEKWK	AAY	WKV	0.02153	53.38
23	42	20	RVRGGRCAVLSCLPKEEQIG	CRV	IGK	0.02153	53.42
17	36	20	LQKYYCRVRGGRCAVLSCLP	TLQ	LPK	0.02151	53.46
61	80	20	KAKFVAAWTLKAAAAAYFIG	AKA	IGG	0.02149	53.5
136	154	19	AYMQEKWKVKLAAYRETTE	AAY	TEA	0.02149	53.54
212	230	19	KKTERMQEKWKVKKKAKFV	KKK	FVA	0.02144	53.58
147	162	16	AAYRETTEAAIIAAYI	LAA	YIG	0.02132	53.62
196	214	19	FIAVYYTVLNDLWGNKKKT	GFI	KTE	0.02129	53.66
137	149	13	YMQEKWKVKLAAY	AYM	AYR	0.02119	53.69
85	105	21	GIAAYRMQEKWKVKAAYIQLR	LGI	LRW	0.02112	53.73
113	131	19	YSYCLYWLFVAAYLRWFFV	AYS	FVF	0.02109	53.77
132	150	19	FSTAAYMQEKWKVKLAAYR	VFS	YRE	0.02108	53.81
70	90	21	LKAAAAAYFIGGVSLGIAAYR	TLK	YRM	0.02103	53.85
170	182	13	YGPGPGAFIAVYY	VYG	YYT	0.02097	53.89
136	155	20	AYMQEKWKVKLAAYRETTEA	AAY	EAA	0.02076	53.93
173	192	20	GPGAFIAVYYTVLNDLWGGP	PGP	GPG	0.02074	53.97
122	141	20	VAAYLRWFFVFSTAAYMQEK	FVA	EKW	0.02072	54.0
50	63	14	KCCRRKKEAAAKAK	RKC	AKF	0.02071	54.04
67	79	13	AWTLKAAAAAYFI	AAW	FIG	0.02057	54.08
149	167	19	YRETTEAAIIAAYIGAAFI	AYR	FIA	0.02057	54.12
92	110	19	QEKWKVKAAYIQLRWFFVF	MQE	VFA	0.02055	54.16
228	247	20	KFVAAWTLKAAAKKTGALLA	AKF	LAA	0.02053	54.2
177	189	13	FIAVYYTVLNDLW	AFI	LWG	0.02052	54.24
201	220	20	YTVLNDLWGNKKKTERMQEK	YYT	EKW	0.0205	54.27
59	78	20	AAKAKFVAAWTLKAAAAAYF	AAA	YFI	0.02049	54.31
160	179	20	AYIGAAFIAVYGPGPGAFIA	AAY	IAV	0.02049	54.35
188	205	18	LWGGPGPGFIAVYYTVLN	DLW	LND	0.02045	54.39
229	249	21	FVAAWTLKAAAKKTGALLAAG	KFV	AGA	0.02037	54.43
186	205	20	NDLWGGPGPGFIAVYYTVLN	LND	LND	0.02034	54.47
178	196	19	IAVYYTVLNDLWGGPGPGF	FIA	GFI	0.02032	54.51
132	149	18	FSTAAYMQEKWKVKLAAY	VFS	AYR	0.0203	54.55
26	47	22	GGRCAVLSCLPKEEQIGKCSTR	RGG	TRG	0.02027	54.58
85	102	18	GIAAYRMQEKWKVKAAYI	LGI	YIQ	0.02026	54.62
155	174	20	AAIIAAYIGAAFIAVYGPGP	EAA	GPG	0.02023	54.66
224	242	19	KKKAKFVAAWTLKAAAKKT	VKK	KTG	0.02018	54.7
40	53	14	QIGKCSTRGRKCCR	EQI	CRR	0.02018	54.74
189	207	19	WGGPGPGFIAVYYTVLNDL	LWG	DLW	0.02018	54.78

200	217	18	YYTVLNDLWGNKKKTERM	VYY	RMQ	0.02016	54.82
62	79	18	AKFVAAWTLKAAAAAYFI	KAK	FIG	0.02012	54.85
89	108	20	YRMQEKWKVKAAYIQLRWFF	AYR	FFV	0.02012	54.89
218	235	18	QEKWKVKKKAKFVAAWTL	MQE	TLK	0.02009	54.93
24	41	18	VRGGRCAVLSCLPKEEQI	RVR	QIG	0.02005	54.97
224	243	20	KKKAKFVAAWTLKAAAKKTG	VKK	TGA	0.02004	55.01
49	68	20	RKCCRRKKEAAAKAKFVAAW	GRK	AWT	0.02004	55.05
141	159	19	KWKVKLAAYRETTEAAIIA	EKW	IAA	0.01998	55.09
151	169	19	ETTEAAIIAAYIGAAFIAV	RET	AVY	0.01997	55.13
179	196	18	AVYYTVLNDLWGGPGPGF	IAV	GFI	0.01996	55.16
196	215	20	FIAVYYTVLNDLWGNKKKTE	GFI	TER	0.01995	55.2
84	101	18	LGIAAYRMQEKWKVKAAY	SLG	AYI	0.01991	55.24
4	20	17	HALEAAAKGIINTLQKY	PHA	KYY	0.01985	55.28
99	118	20	AAYIQLRWFFVFAAYSYCLY	KAA	LYW	0.01979	55.32
148	162	15	AYRETTEAAIIAAYI	AAY	YIG	0.01978	55.36
227	247	21	AKFVAAWTLKAAAKKTGALLA	KAK	LAA	0.01975	55.4
234	253	20	TLKAAAKKTGALLAAGAAAK	WTL	AKK	0.01973	55.44
187	206	20	DLWGGPGPGFIAVYYTVLND	NDL	NDL	0.01966	55.47
126	142	17	LRWFFVFSTAAYMQEKW	YLR	KWK	0.01962	55.51
219	239	21	EKWKVKKKAKFVAAWTLKAAA	QEK	AAK	0.0196	55.55
154	173	20	EAAIIAAYIGAAFIAVYGPG	TEA	PGP	0.01957	55.59
229	248	20	FVAAWTLKAAAKKTGALLAA	KFV	AAG	0.01939	55.63
140	160	21	EKWKVKLAAYRETTEAAIIAA	QEK	AAY	0.01937	55.67
145	157	13	KLAAYRETTEAAI	VKL	AII	0.01936	55.71
121	139	19	FVAAYLRWFFVFSTAAYMQ	LFV	MQE	0.01935	55.74
55	74	20	KKEAAAKAKFVAAWTLKAAA	RKK	AAA	0.01932	55.78
185	197	13	LNDLWGGPGPGFI	VLN	FIA	0.01926	55.82
69	82	14	TLKAAAAAYFIGGV	WTL	GVS	0.01925	55.86
184	201	18	VLNDLWGGPGPGFIAVYY	TVL	YYT	0.01919	55.9
180	197	18	VYYTVLNDLWGGPGPGFI	AVY	FIA	0.01919	55.94
32	50	19	LSCLPKEEQIGKCSTRGRK	VLS	RKC	0.01918	55.98
48	67	20	GRKCCRRKKEAAAKAKFVAA	RGR	AAW	0.01911	56.02
73	93	21	AAAAYFIGGVSLGIAAYRMQE	AAA	QEK	0.01908	56.05
78	95	18	FIGGVSLGIAAYRMQEKW	YFI	KWK	0.01897	56.09
217	236	20	MQEKWKVKKKAKFVAAWTLK	RMQ	LKA	0.01896	56.13
85	104	20	GIAAYRMQEKWKVKAAYIQL	LGI	QLR	0.01892	56.17
104	123	20	LRWFFVFAAYSYCLYWLFVA	QLR	VAA	0.01883	56.21
129	148	20	FFVFSTAAYMQEKWKVKLAA	WFF	AAY	0.0188	56.25
232	251	20	AWTLKAAAKKTGALLAAGAA	AAW	AAA	0.01872	56.29
102	120	19	IQLRWFFVFAAYSYCLYWL	YIQ	WLF	0.01865	56.32
58	77	20	AAAKAKFVAAWTLKAAAAAY	EAA	AYF	0.0186	56.36
223	242	20	VKKKAKFVAAWTLKAAAKKT	KVK	KTG	0.0186	56.36
14	32	19	INTLQKYYCRVRGGRCAVL	IIN	VLS	0.01858	56.44
117	129	13	LYWLFVAAYLRWF	CLY	WFF	0.01857	56.48
141	158	18	KWKVKLAAYRETTEAAII	EKW	IIA	0.01857	56.52
131	150	20	VFSTAAYMQEKWKVKLAAYR	FVF	YRE	0.01857	56.56
204	222	19	LNDLWGNKKKTERMQEKWK	VLN	WKV	0.01851	56.6
221	240	20	WKVKKKAKFVAAWTLKAAAK	KWK	AKK	0.01848	56.63
91	109	19	MQEKWKVKAAYIQLRWFFV	RMQ	FVF	0.01847	56.67

68	82	15	WTLKAAAAAYFIGGV	AWT	GVS	0.01846	56.71
37	55	19	KEEQIGKCSTRGRKCCRRK	PKE	RKK	0.01843	56.75
60	80	21	AKAKFVAAWTLKAAAAAYFIG	AAK	IGG	0.01842	56.79
137	153	17	YMQEKWKVKLAAYRETT	AYM	TTE	0.01842	56.83
13	32	20	IINTLQKYYCRVRGGRCAVL	GII	VLS	0.0184	56.87
79	99	21	IGGVSLGIAAYRMQEKWKVKA	FIG	KAA	0.01833	56.91
215	233	19	ERMQEKWKVKKKAKFVAAW	TER	AWT	0.01829	56.94
104	121	18	LRWFFVFAAYSYCLYWLF	QLR	LFV	0.01828	56.98
39	59	21	EQIGKCSTRGRKCCRRKKEAA	EEQ	AAA	0.0182	57.02
199	217	19	VYYTVLNDLWGNKKKTERM	AVY	RMQ	0.01802	57.06
138	156	19	MQEKWKVKLAAYRETTEAA	YMQ	AAI	0.01802	57.1
43	58	16	KCSTRGRKCCRRKKEA	GKC	EAA	0.018	57.14
74	94	21	AAAYFIGGVSLGIAAYRMQEK	AAA	EKW	0.01799	57.18
150	167	18	RETTEAAIIAAYIGAAFI	YRE	FIA	0.01796	57.21
223	243	21	VKKKAKFVAAWTLKAAAKKTG	KVK	TGA	0.01795	57.25
225	244	20	KKAKFVAAWTLKAAAKKTGA	KKK	GAL	0.01791	57.29
43	56	14	KCSTRGRKCCRRKK	GKC	KKE	0.0179	57.33
69	88	20	TLKAAAAAYFIGGVSLGIAA	WTL	AAY	0.01787	57.37
8	28	21	AAAKGIINTLQKYYCRVRGGR	EAA	GRC	0.01786	57.41
202	222	21	TVLNDLWGNKKKTERMQEKWK	YTV	WKV	0.01779	57.45
20	38	19	YYCRVRGGRCAVLSCLPKE	KYY	KEE	0.01774	57.49
53	70	18	RRKKEAAAKAKFVAAWTL	CRR	TLK	0.01771	57.52
3	19	17	PHALEAAAKGIINTLQK	PPH	QKY	0.01768	57.56
36	48	13	PKEEQIGKCSTRG	LPK	RGR	0.01767	57.6
7	24	18	EAAAKGIINTLQKYYCRV	LEA	RVR	0.01765	57.64
171	191	21	GPGPGAFIAVYYTVLNDLWGG	YGP	GGP	0.01765	57.68
106	124	19	WFFVFAAYSYCLYWLFVAA	RWF	AAY	0.01764	57.72
88	106	19	AYRMQEKWKVKAAYIQLRW	AAY	RWF	0.01747	57.76
112	130	19	AYSYCLYWLFVAAYLRWFF	AAY	FFV	0.01744	57.79
26	48	23	GGRCAVLSCLPKEEQIGKCSTRG	RGG	RGR	0.01737	57.83
217	234	18	MQEKWKVKKKAKFVAAWT	RMQ	WTL	0.01737	57.87
113	132	20	YSYCLYWLFVAAYLRWFFVF	AYS	VFS	0.01736	57.91
163	180	18	GAAFIAVYGPGPGAFIAV	IGA	AVY	0.01734	57.95
35	53	19	LPKEEQIGKCSTRGRKCCR	CLP	CRR	0.01733	57.99
175	189	15	GAFIAVYYTVLNDLW	PGA	LWG	0.01727	58.03
14	33	20	INTLQKYYCRVRGGRCAVLS	IIN	LSC	0.01725	58.07
161	181	21	YIGAAFIAVYGPGPGAFIAVY	AYI	VYY	0.01719	58.1
96	114	19	KVKAAYIQLRWFFVFAAYS	WKV	YSY	0.01717	58.14
120	137	18	LFVAAYLRWFFVFSTAAY	WLF	AYM	0.01715	58.18
90	108	19	RMQEKWKVKAAYIQLRWFF	YRM	FFV	0.01712	58.22
211	230	20	KKKTERMQEKWKVKKKAKFV	NKK	FVA	0.01711	58.26
8	27	20	AAAKGIINTLQKYYCRVRGG	EAA	GGR	0.01709	58.3
195	208	14	GFIAVYYTVLNDLW	PGF	LWG	0.01706	58.34
175	195	21	GAFIAVYYTVLNDLWGGPGPG	PGA	PGF	0.01701	58.38
121	138	18	FVAAYLRWFFVFSTAAYM	LFV	YMQ	0.01699	58.41
30	49	20	AVLSCLPKEEQIGKCSTRGR	CAV	GRK	0.01698	58.45
107	127	21	FFVFAAYSYCLYWLFVAAYLR	WFF	LRW	0.01694	58.49
119	136	18	WLFVAAYLRWFFVFSTAA	YWL	AAY	0.0169	58.53
208	228	21	WGNKKKTERMQEKWKVKKKAK	LWG	AKF	0.01688	58.57

72	92	21	AAAAAYFIGGVSLGIAAYRMQ	KAA	MQE	0.01683	58.61
159	171	13	AAYIGAAFIAVYG	IAA	YGP	0.01682	58.65
171	193	23	GPGPGAFIAVYYTVLNDLWGGPG	YGP	PGP	0.01682	58.68
216	236	21	RMQEKWKVKKKAKFVAAWTLK	ERM	LKA	0.01666	58.72
131	149	19	VFSTAAYMQEKWKVKLAAY	FVF	AYR	0.01662	58.76
19	38	20	KYYCRVRGGRCAVLSCLPKE	QKY	KEE	0.01662	58.8
13	33	21	IINTLQKYYCRVRGGRCAVLS	GII	LSC	0.0166	58.84
108	126	19	FVFAAYSYCLYWLFVAAYL	FFV	YLR	0.01656	58.88
117	135	19	LYWLFVAAYLRWFFVFSTA	CLY	TAA	0.01651	58.92
41	63	23	IGKCSTRGRKCCRRKKEAAAKAK	QIG	AKF	0.01647	58.96
89	107	19	YRMQEKWKVKAAYIQLRWF	AYR	WFF	0.01639	58.99
66	85	20	AAWTLKAAAAAYFIGGVSLG	VAA	LGI	0.01639	59.03
153	172	20	TEAAIIAAYIGAAFIAVYGP	TTE	GPG	0.01633	59.07
154	171	18	EAAIIAAYIGAAFIAVYG	TEA	YGP	0.01633	59.11
165	183	19	AFIAVYGPGPGAFIAVYYT	AAF	YTV	0.01632	59.15
127	147	21	RWFFVFSTAAYMQEKWKVKLA	LRW	LAA	0.0163	59.19
78	97	20	FIGGVSLGIAAYRMQEKWKV	YFI	KVK	0.01627	59.23
67	82	16	AWTLKAAAAAYFIGGV	AAW	GVS	0.01622	59.26
169	187	19	VYGPGPGAFIAVYYTVLND	AVY	NDL	0.01622	59.3
20	39	20	YYCRVRGGRCAVLSCLPKEE	KYY	EEQ	0.01621	59.34
82	103	22	VSLGIAAYRMQEKWKVKAAYIQ	GVS	IQL	0.01606	59.38
107	125	19	FFVFAAYSYCLYWLFVAAY	WFF	AYL	0.01604	59.42
12	31	20	GIINTLQKYYCRVRGGRCAV	KGI	AVL	0.01603	59.46
226	245	20	KAKFVAAWTLKAAAKKTGAL	KKA	ALL	0.01601	59.5
11	23	13	KGIINTLQKYYCR	AKG	CRV	0.016	59.54
100	118	19	AYIQLRWFFVFAAYSYCLY	AAY	LYW	0.01596	59.57
125	143	19	YLRWFFVFSTAAYMQEKWK	AYL	WKV	0.01594	59.61
173	195	23	GPGAFIAVYYTVLNDLWGGPGPG	PGP	PGF	0.01591	59.65
125	137	13	YLRWFFVFSTAAY	AYL	AYM	0.01588	59.69
83	95	13	SLGIAAYRMQEKW	VSL	KWK	0.01582	59.73
220	239	20	KWKVKKKAKFVAAWTLKAAA	EKW	AAK	0.0158	59.77
233	252	20	WTLKAAAKKTGALLAAGAAA	AWT	AAK	0.0158	59.77
3	18	16	PHALEAAAKGIINTLQ	PPH	LQK	0.01576	59.85
46	65	20	TRGRKCCRRKKEAAAKAKFV	STR	FVA	0.01575	59.88
7	26	20	EAAAKGIINTLQKYYCRVRG	LEA	RGG	0.0157	59.92
213	232	20	KTERMQEKWKVKKKAKFVAA	KKT	AAW	0.01568	59.96
30	50	21	AVLSCLPKEEQIGKCSTRGRK	CAV	RKC	0.01564	60.0
208	227	20	WGNKKKTERMQEKWKVKKKA	LWG	KAK	0.01563	60.04
141	160	20	KWKVKLAAYRETTEAAIIAA	EKW	AAY	0.01562	60.08
178	197	20	IAVYYTVLNDLWGGPGPGFI	FIA	FIA	0.01561	60.12
43	55	13	KCSTRGRKCCRRK	GKC	RKK	0.01561	60.15
22	37	16	CRVRGGRCAVLSCLPK	YCR	PKE	0.01561	60.19
170	188	19	YGPGPGAFIAVYYTVLNDL	VYG	DLW	0.0155	60.23
136	156	21	AYMQEKWKVKLAAYRETTEAA	AAY	AAI	0.01548	60.27
197	217	21	IAVYYTVLNDLWGNKKKTERM	FIA	RMQ	0.01533	60.31
132	151	20	FSTAAYMQEKWKVKLAAYRE	VFS	RET	0.01529	60.35
112	129	18	AYSYCLYWLFVAAYLRWF	AAY	WFF	0.01529	60.39
185	202	18	LNDLWGGPGPGFIAVYYT	VLN	YTV	0.01519	60.43
121	140	20	FVAAYLRWFFVFSTAAYMQE	LFV	QEK	0.01517	60.46

97	115	19	VKAAYIQLRWFFVFAAYSY	KVK	SYC	0.01514	60.5
135	156	22	AAYMQEKWKVKLAAYRETTEAA	TAA	AAI	0.01513	60.54
80	99	20	GGVSLGIAAYRMQEKWKVKA	IGG	KAA	0.01507	60.58
218	237	20	QEKWKVKKKAKFVAAWTLKA	MQE	KAA	0.01507	60.58
54	74	21	RKKEAAAKAKFVAAWTLKAAA	RRK	AAA	0.01506	60.66
164	183	20	AAFIAVYGPGPGAFIAVYYT	GAA	YTV	0.01503	60.7
90	107	18	RMQEKWKVKAAYIQLRWF	YRM	WFF	0.01501	60.74
111	130	20	AAYSYCLYWLFVAAYLRWFF	FAA	FFV	0.01499	60.77
126	144	19	LRWFFVFSTAAYMQEKWKV	YLR	KVK	0.01497	60.81
143	161	19	KVKLAAYRETTEAAIIAAY	WKV	AYI	0.01497	60.85
27	46	20	GRCAVLSCLPKEEQIGKCST	GGR	STR	0.0149	60.89
17	30	14	LQKYYCRVRGGRCA	TLQ	CAV	0.01484	60.93
146	164	19	LAAYRETTEAAIIAAYIGA	KLA	GAA	0.0148	60.97
114	133	20	SYCLYWLFVAAYLRWFFVFS	YSY	FST	0.01478	61.01
19	39	21	KYYCRVRGGRCAVLSCLPKEE	QKY	EEQ	0.01476	61.04
154	174	21	EAAIIAAYIGAAFIAVYGPGP	TEA	GPG	0.01473	61.08
159	176	18	AAYIGAAFIAVYGPGPGA	IAA	GAF	0.01466	61.12
216	234	19	RMQEKWKVKKKAKFVAAWT	ERM	WTL	0.0146	61.16
61	81	21	KAKFVAAWTLKAAAAAYFIGG	AKA	GGV	0.01455	61.2
207	226	20	LWGNKKKTERMQEKWKVKKK	DLW	KKA	0.01454	61.24
200	218	19	YYTVLNDLWGNKKKTERMQ	VYY	MQE	0.01453	61.28
232	250	19	AWTLKAAAKKTGALLAAGA	AAW	GAA	0.01449	61.32
205	225	21	NDLWGNKKKTERMQEKWKVKK	LND	KKK	0.01445	61.35
27	47	21	GRCAVLSCLPKEEQIGKCSTR	GGR	TRG	0.01445	61.39
198	217	20	AVYYTVLNDLWGNKKKTERM	IAV	RMQ	0.0144	61.43
89	109	21	YRMQEKWKVKAAYIQLRWFFV	AYR	FVF	0.01433	61.47
127	146	20	RWFFVFSTAAYMQEKWKVKL	LRW	KLA	0.01426	61.51
179	197	19	AVYYTVLNDLWGGPGPGFI	IAV	FIA	0.01426	61.55
137	154	18	YMQEKWKVKLAAYRETTE	AYM	TEA	0.0142	61.59
41	62	22	IGKCSTRGRKCCRRKKEAAAKA	QIG	KAK	0.01415	61.62
16	37	22	TLQKYYCRVRGGRCAVLSCLPK	NTL	PKE	0.01414	61.66
218	238	21	QEKWKVKKKAKFVAAWTLKAA	MQE	AAA	0.01408	61.7
75	96	22	AAYFIGGVSLGIAAYRMQEKWK	AAA	WKV	0.01406	61.74
159	177	19	AAYIGAAFIAVYGPGPGAF	IAA	AFI	0.01404	61.78
228	246	19	KFVAAWTLKAAAKKTGALL	AKF	LLA	0.01404	61.78
183	202	20	TVLNDLWGGPGPGFIAVYYT	YTV	YTV	0.01396	61.86
199	218	20	VYYTVLNDLWGNKKKTERMQ	AVY	MQE	0.01396	61.9
207	225	19	LWGNKKKTERMQEKWKVKK	DLW	KKK	0.0139	61.93
187	207	21	DLWGGPGPGFIAVYYTVLNDL	NDL	DLW	0.01389	61.97
227	246	20	AKFVAAWTLKAAAKKTGALL	KAK	LLA	0.01389	62.01
48	68	21	GRKCCRRKKEAAAKAKFVAAW	RGR	AWT	0.01387	62.05
67	84	18	AWTLKAAAAAYFIGGVSL	AAW	SLG	0.01386	62.09
7	23	17	EAAAKGIINTLQKYYCR	LEA	CRV	0.01383	62.13
167	187	21	IAVYGPGPGAFIAVYYTVLND	FIA	NDL	0.0138	62.17
76	96	21	AYFIGGVSLGIAAYRMQEKWK	AAY	WKV	0.01369	62.21
123	143	21	AAYLRWFFVFSTAAYMQEKWK	VAA	WKV	0.01369	62.21
65	85	21	VAAWTLKAAAAAYFIGGVSLG	FVA	LGI	0.01369	62.28
77	98	22	YFIGGVSLGIAAYRMQEKWKVK	AYF	VKA	0.01366	62.32
159	179	21	AAYIGAAFIAVYGPGPGAFIA	IAA	IAV	0.01364	62.36

102	121	20	IQLRWFFVFAAYSYCLYWLF	YIQ	LFV	0.01361	62.4
165	182	18	AFIAVYGPGPGAFIAVYY	AAF	YYT	0.01357	62.44
128	147	20	WFFVFSTAAYMQEKWKVKLA	RWF	LAA	0.01353	62.48
152	170	19	TTEAAIIAAYIGAAFIAVY	ETT	VYG	0.01353	62.51
148	166	19	AYRETTEAAIIAAYIGAAF	AAY	AFI	0.01342	62.55
5	17	13	ALEAAAKGIINTL	HAL	TLQ	0.01341	62.59
91	111	21	MQEKWKVKAAYIQLRWFFVFA	RMQ	FAA	0.01338	62.63
221	241	21	WKVKKKAKFVAAWTLKAAAKK	KWK	KKT	0.01335	62.67
133	153	21	STAAYMQEKWKVKLAAYRETT	FST	TTE	0.01334	62.71
6	24	19	LEAAAKGIINTLQKYYCRV	ALE	RVR	0.01332	62.75
53	72	20	RRKKEAAAKAKFVAAWTLKA	CRR	KAA	0.01329	62.79
107	126	20	FFVFAAYSYCLYWLFVAAYL	WFF	YLR	0.01325	62.82
205	226	22	NDLWGNKKKTERMQEKWKVKKK	LND	KKA	0.01323	62.86
185	201	17	LNDLWGGPGPGFIAVYY	VLN	YYT	0.0132	62.9
231	251	21	AAWTLKAAAKKTGALLAAGAA	VAA	AAA	0.01313	62.94
131	151	21	VFSTAAYMQEKWKVKLAAYRE	FVF	RET	0.0131	62.98
68	87	20	WTLKAAAAAYFIGGVSLGIA	AWT	IAA	0.01307	63.02
175	191	17	GAFIAVYYTVLNDLWGG	PGA	GGP	0.01307	63.06
151	170	20	ETTEAAIIAAYIGAAFIAVY	RET	VYG	0.01306	63.09
120	139	20	LFVAAYLRWFFVFSTAAYMQ	WLF	MQE	0.01303	63.13
148	168	21	AYRETTEAAIIAAYIGAAFIA	AAY	IAV	0.01303	63.17
18	36	19	QKYYCRVRGGRCAVLSCLP	LQK	LPK	0.013	63.21
38	59	22	EEQIGKCSTRGRKCCRRKKEAA	KEE	AAA	0.01298	63.25
168	187	20	AVYGPGPGAFIAVYYTVLND	IAV	NDL	0.01296	63.29
190	210	21	GGPGPGFIAVYYTVLNDLWGN	WGG	GNK	0.01294	63.33
69	90	22	TLKAAAAAYFIGGVSLGIAAYR	WTL	YRM	0.01294	63.37
102	123	22	IQLRWFFVFAAYSYCLYWLFVA	YIQ	VAA	0.01283	63.4
166	185	20	FIAVYGPGPGAFIAVYYTVL	AFI	VLN	0.0128	63.44
112	131	20	AYSYCLYWLFVAAYLRWFFV	AAY	FVF	0.01278	63.48
47	67	21	RGRKCCRRKKEAAAKAKFVAA	TRG	AAW	0.01277	63.52
137	155	19	YMQEKWKVKLAAYRETTEA	AYM	EAA	0.01276	63.56
57	77	21	EAAAKAKFVAAWTLKAAAAAY	KEA	AYF	0.01275	63.6
104	122	19	LRWFFVFAAYSYCLYWLFV	QLR	FVA	0.01255	63.64
90	109	20	RMQEKWKVKAAYIQLRWFFV	YRM	FVF	0.01254	63.68
82	101	20	VSLGIAAYRMQEKWKVKAAY	GVS	AYI	0.01252	63.71
153	175	23	TEAAIIAAYIGAAFIAVYGPGPG	TTE	PGA	0.01252	63.75
146	165	20	LAAYRETTEAAIIAAYIGAA	KLA	AAF	0.01252	63.79
225	245	21	KKAKFVAAWTLKAAAKKTGAL	KKK	ALL	0.01252	63.83
86	106	21	IAAYRMQEKWKVKAAYIQLRW	GIA	RWF	0.0125	63.87
79	100	22	IGGVSLGIAAYRMQEKWKVKAA	FIG	AAY	0.01249	63.91
42	62	21	GKCSTRGRKCCRRKKEAAAKA	IGK	KAK	0.01247	63.95
210	229	20	NKKKTERMQEKWKVKKKAKF	GNK	KFV	0.01246	63.98
70	82	13	LKAAAAAYFIGGV	TLK	GVS	0.01245	64.02
101	119	19	YIQLRWFFVFAAYSYCLYW	AYI	YWL	0.01243	64.06
53	73	21	RRKKEAAAKAKFVAAWTLKAA	CRR	AAA	0.01241	64.1
6	26	21	LEAAAKGIINTLQKYYCRVRG	ALE	RGG	0.01239	64.14
161	180	20	YIGAAFIAVYGPGPGAFIAV	AYI	AVY	0.01235	64.18
181	200	20	YYTVLNDLWGGPGPGFIAVY	VYY	VYY	0.01229	64.22
55	75	21	KKEAAAKAKFVAAWTLKAAAA	RKK	AAA	0.01229	64.26

219	240	22	EKWKVKKKAKFVAAWTLKAAAK	QEK	AKK	0.01225	64.29
111	129	19	AAYSYCLYWLFVAAYLRWF	FAA	WFF	0.01222	64.33
42	63	22	GKCSTRGRKCCRRKKEAAAKAK	IGK	AKF	0.01212	64.37
203	224	22	VLNDLWGNKKKTERMQEKWKVK	TVL	VKK	0.01212	64.37
95	113	19	WKVKAAYIQLRWFFVFAAY	KWK	AYS	0.0121	64.45
35	51	17	LPKEEQIGKCSTRGRKC	CLP	KCC	0.01209	64.49
43	59	17	KCSTRGRKCCRRKKEAA	GKC	AAA	0.01207	64.53
66	82	17	AAWTLKAAAAAYFIGGV	VAA	GVS	0.01206	64.56
40	60	21	QIGKCSTRGRKCCRRKKEAAA	EQI	AAK	0.01205	64.6
110	128	19	FAAYSYCLYWLFVAAYLRW	VFA	RWF	0.01201	64.64
70	86	17	LKAAAAAYFIGGVSLGI	TLK	GIA	0.01199	64.68
50	66	17	KCCRRKKEAAAKAKFVA	RKC	VAA	0.01198	64.72
87	106	20	AAYRMQEKWKVKAAYIQLRW	IAA	RWF	0.01196	64.76
91	110	20	MQEKWKVKAAYIQLRWFFVF	RMQ	VFA	0.01185	64.8
169	188	20	VYGPGPGAFIAVYYTVLNDL	AVY	DLW	0.01179	64.84
130	150	21	FVFSTAAYMQEKWKVKLAAYR	FFV	YRE	0.01172	64.87
150	162	13	RETTEAAIIAAYI	YRE	YIG	0.01172	64.91
12	32	21	GIINTLQKYYCRVRGGRCAVL	KGI	VLS	0.0117	64.95
81	103	23	GVSLGIAAYRMQEKWKVKAAYIQ	GGV	IQL	0.01168	64.99
70	89	20	LKAAAAAYFIGGVSLGIAAY	TLK	AYR	0.01168	65.03
203	221	19	VLNDLWGNKKKTERMQEKW	TVL	KWK	0.01167	65.07
160	178	19	AYIGAAFIAVYGPGPGAFI	AAY	FIA	0.01167	65.11
2	20	19	PPHALEAAAKGIINTLQKY	APP	KYY	0.01163	65.15
164	182	19	AAFIAVYGPGPGAFIAVYY	GAA	YYT	0.01162	65.18
72	93	22	AAAAAYFIGGVSLGIAAYRMQE	KAA	QEK	0.01155	65.22
128	146	19	WFFVFSTAAYMQEKWKVKL	RWF	KLA	0.01151	65.26
58	78	21	AAAKAKFVAAWTLKAAAAAYF	EAA	YFI	0.0115	65.3
180	200	21	VYYTVLNDLWGGPGPGFIAVY	AVY	VYY	0.01149	65.34
103	118	16	QLRWFFVFAAYSYCLY	IQL	LYW	0.01146	65.38
134	153	20	TAAYMQEKWKVKLAAYRETT	STA	TTE	0.01145	65.42
209	229	21	GNKKKTERMQEKWKVKKKAKF	WGN	KFV	0.01144	65.45
102	117	16	IQLRWFFVFAAYSYCL	YIQ	CLY	0.01141	65.49
80	100	21	GGVSLGIAAYRMQEKWKVKAA	IGG	AAY	0.01141	65.53
92	112	21	QEKWKVKAAYIQLRWFFVFAA	MQE	AAY	0.01141	65.53
213	233	21	KTERMQEKWKVKKKAKFVAAW	KKT	AWT	0.01139	65.61
117	136	20	LYWLFVAAYLRWFFVFSTAA	CLY	AAY	0.01139	65.65
200	219	20	YYTVLNDLWGNKKKTERMQE	VYY	QEK	0.01139	65.65
61	79	19	KAKFVAAWTLKAAAAAYFI	AKA	FIG	0.01135	65.73
16	30	15	TLQKYYCRVRGGRCA	NTL	CAV	0.01131	65.76
175	193	19	GAFIAVYYTVLNDLWGGPG	PGA	PGP	0.01124	65.8
60	81	22	AKAKFVAAWTLKAAAAAYFIGG	AAK	GGV	0.01123	65.84
40	61	22	QIGKCSTRGRKCCRRKKEAAAK	EQI	AKA	0.01122	65.88
124	145	22	AYLRWFFVFSTAAYMQEKWKVK	AAY	VKL	0.01118	65.92
212	232	21	KKTERMQEKWKVKKKAKFVAA	KKK	AAW	0.01118	65.96
146	162	17	LAAYRETTEAAIIAAYI	KLA	YIG	0.01116	66.0
173	193	21	GPGAFIAVYYTVLNDLWGGPG	PGP	PGP	0.01114	66.03
63	83	21	KFVAAWTLKAAAAAYFIGGVS	AKF	VSL	0.01113	66.07
113	133	21	YSYCLYWLFVAAYLRWFFVFS	AYS	FST	0.01104	66.11
158	177	20	IAAYIGAAFIAVYGPGPGAF	IIA	AFI	0.01104	66.15

173	191	19	GPGAFIAVYYTVLNDLWGG	PGP	GGP	0.01103	66.19
172	186	15	PGPGAFIAVYYTVLN	GPG	LND	0.011	66.23
220	240	21	KWKVKKKAKFVAAWTLKAAAK	EKW	AKK	0.01097	66.27
233	253	21	WTLKAAAKKTGALLAAGAAAK	AWT	AKK	0.01097	66.27
206	228	23	DLWGNKKKTERMQEKWKVKKKAK	NDL	AKF	0.01096	66.34
186	206	21	NDLWGGPGPGFIAVYYTVLND	LND	NDL	0.01095	66.38
162	183	22	IGAAFIAVYGPGPGAFIAVYYT	YIG	YTV	0.01089	66.42
198	218	21	AVYYTVLNDLWGNKKKTERMQ	IAV	MQE	0.01085	66.46
130	149	20	FVFSTAAYMQEKWKVKLAAY	FFV	AYR	0.0108	66.5
183	201	19	TVLNDLWGGPGPGFIAVYY	YTV	YYT	0.0108	66.54
217	235	19	MQEKWKVKKKAKFVAAWTL	RMQ	TLK	0.01077	66.58
158	176	19	IAAYIGAAFIAVYGPGPGA	IIA	GAF	0.01072	66.62
111	131	21	AAYSYCLYWLFVAAYLRWFFV	FAA	FVF	0.01068	66.65
71	91	21	KAAAAAYFIGGVSLGIAAYRM	LKA	RMQ	0.01067	66.69
83	103	21	SLGIAAYRMQEKWKVKAAYIQ	VSL	IQL	0.01066	66.73
199	219	21	VYYTVLNDLWGNKKKTERMQE	AVY	QEK	0.01064	66.77
120	138	19	LFVAAYLRWFFVFSTAAYM	WLF	YMQ	0.01064	66.81
101	120	20	YIQLRWFFVFAAYSYCLYWL	AYI	WLF	0.01062	66.85
166	184	19	FIAVYGPGPGAFIAVYYTV	AFI	TVL	0.01061	66.89
109	128	20	VFAAYSYCLYWLFVAAYLRW	FVF	RWF	0.01058	66.92
144	164	21	VKLAAYRETTEAAIIAAYIGA	KVK	GAA	0.01053	66.96
188	206	19	LWGGPGPGFIAVYYTVLND	DLW	NDL	0.01053	67.0
203	223	21	VLNDLWGNKKKTERMQEKWKV	TVL	KVK	0.01047	67.04
147	166	20	AAYRETTEAAIIAAYIGAAF	LAA	AFI	0.01046	67.08
45	64	20	STRGRKCCRRKKEAAAKAKF	CST	KFV	0.01046	67.12
231	250	20	AAWTLKAAAKKTGALLAAGA	VAA	GAA	0.01046	67.12
197	218	22	IAVYYTVLNDLWGNKKKTERMQ	FIA	MQE	0.0104	67.2
139	157	19	QEKWKVKLAAYRETTEAAI	MQE	AII	0.01039	67.23
27	48	22	GRCAVLSCLPKEEQIGKCSTRG	GGR	RGR	0.01034	67.27
174	188	15	PGAFIAVYYTVLNDL	GPG	DLW	0.01034	67.31
194	209	16	PGFIAVYYTVLNDLWG	GPG	WGN	0.01033	67.35
73	94	22	AAAAYFIGGVSLGIAAYRMQEK	AAA	EKW	0.0103	67.39
122	143	22	VAAYLRWFFVFSTAAYMQEKWK	FVA	WKV	0.0103	67.43
133	155	23	STAAYMQEKWKVKLAAYRETTEA	FST	EAA	0.01024	67.47
112	132	21	AYSYCLYWLFVAAYLRWFFVF	AAY	VFS	0.01023	67.5
201	222	22	YTVLNDLWGNKKKTERMQEKWK	YYT	WKV	0.01019	67.54
166	186	21	FIAVYGPGPGAFIAVYYTVLN	AFI	LND	0.01018	67.58
77	95	19	YFIGGVSLGIAAYRMQEKW	AYF	KWK	0.01015	67.62
133	154	22	STAAYMQEKWKVKLAAYRETTE	FST	TEA	0.01012	67.66
20	40	21	YYCRVRGGRCAVLSCLPKEEQ	KYY	EQI	0.01011	67.7
50	64	15	KCCRRKKEAAAKAKF	RKC	KFV	0.01003	67.74
192	205	14	PGPGFIAVYYTVLN	GPG	LND	0.01002	67.78
142	163	22	WKVKLAAYRETTEAAIIAAYIG	KWK	IGA	0.01002	67.81
60	79	20	AKAKFVAAWTLKAAAAAYFI	AAK	FIG	0.01	67.85
6	23	18	LEAAAKGIINTLQKYYCR	ALE	CRV	0.00999	67.89
5	21	17	ALEAAAKGIINTLQKYY	HAL	YYC	0.00998	67.93
222	242	21	KVKKKAKFVAAWTLKAAAKKT	WKV	KTG	0.00997	67.97
68	88	21	WTLKAAAAAYFIGGVSLGIAA	AWT	AAY	0.00993	68.01
120	140	21	LFVAAYLRWFFVFSTAAYMQE	WLF	QEK	0.00993	68.01

230	251	22	VAAWTLKAAAKKTGALLAAGAA	FVA	AAA	0.00987	68.09
15	30	16	NTLQKYYCRVRGGRCA	INT	CAV	0.00979	68.12
137	156	20	YMQEKWKVKLAAYRETTEAA	AYM	AAI	0.00979	68.16
89	111	23	YRMQEKWKVKAAYIQLRWFFVFA	AYR	FAA	0.00978	68.2
104	124	21	LRWFFVFAAYSYCLYWLFVAA	QLR	AAY	0.00978	68.24
102	122	21	IQLRWFFVFAAYSYCLYWLFV	YIQ	FVA	0.00977	68.28
232	252	21	AWTLKAAAKKTGALLAAGAAA	AAW	AAK	0.00976	68.32
216	235	20	RMQEKWKVKKKAKFVAAWTL	ERM	TLK	0.00974	68.36
134	154	21	TAAYMQEKWKVKLAAYRETTE	STA	TEA	0.00965	68.39
62	83	22	AKFVAAWTLKAAAAAYFIGGVS	KAK	VSL	0.00964	68.43
74	96	23	AAAYFIGGVSLGIAAYRMQEKWK	AAA	WKV	0.00963	68.47
98	118	21	KAAYIQLRWFFVFAAYSYCLY	VKA	LYW	0.00962	68.51
162	182	21	IGAAFIAVYGPGPGAFIAVYY	YIG	YYT	0.00962	68.55
37	56	20	KEEQIGKCSTRGRKCCRRKK	PKE	KKE	0.00961	68.59
59	80	22	AAKAKFVAAWTLKAAAAAYFIG	AAA	IGG	0.00956	68.63
139	159	21	QEKWKVKLAAYRETTEAAIIA	MQE	IAA	0.00955	68.67
219	241	23	EKWKVKKKAKFVAAWTLKAAAKK	QEK	KKT	0.00954	68.7
177	198	22	FIAVYYTVLNDLWGGPGPGFIA	AFI	IAV	0.00952	68.74
39	61	23	EQIGKCSTRGRKCCRRKKEAAAK	EEQ	AKA	0.00951	68.78
12	33	22	GIINTLQKYYCRVRGGRCAVLS	KGI	LSC	0.00951	68.82
16	36	21	TLQKYYCRVRGGRCAVLSCLP	NTL	LPK	0.0095	68.86
84	102	19	LGIAAYRMQEKWKVKAAYI	SLG	YIQ	0.0095	68.9
190	208	19	GGPGPGFIAVYYTVLNDLW	WGG	LWG	0.00946	68.94
125	145	21	YLRWFFVFSTAAYMQEKWKVK	AYL	VKL	0.00945	68.97
206	227	22	DLWGNKKKTERMQEKWKVKKKA	NDL	KAK	0.00942	69.01
194	207	14	PGFIAVYYTVLNDL	GPG	DLW	0.00942	69.05
37	57	21	KEEQIGKCSTRGRKCCRRKKE	PKE	KEA	0.00941	69.09
158	179	22	IAAYIGAAFIAVYGPGPGAFIA	IIA	IAV	0.00938	69.13
192	207	16	PGPGFIAVYYTVLNDL	GPG	DLW	0.00935	69.17
84	105	22	LGIAAYRMQEKWKVKAAYIQLR	SLG	LRW	0.00932	69.21
192	206	15	PGPGFIAVYYTVLND	GPG	NDL	0.0093	69.25
66	84	19	AAWTLKAAAAAYFIGGVSL	VAA	SLG	0.0093	69.28
228	248	21	KFVAAWTLKAAAKKTGALLAA	AKF	AAG	0.00929	69.32
114	134	21	SYCLYWLFVAAYLRWFFVFST	YSY	STA	0.00927	69.36
84	104	21	LGIAAYRMQEKWKVKAAYIQL	SLG	QLR	0.00927	69.4
15	37	23	NTLQKYYCRVRGGRCAVLSCLPK	INT	PKE	0.00927	69.44
189	209	21	WGGPGPGFIAVYYTVLNDLWG	LWG	WGN	0.00927	69.48
23	43	21	RVRGGRCAVLSCLPKEEQIGK	CRV	GKC	0.00925	69.52
149	169	21	YRETTEAAIIAAYIGAAFIAV	AYR	AVY	0.00925	69.56
65	82	18	VAAWTLKAAAAAYFIGGV	FVA	GVS	0.00922	69.59
178	200	23	IAVYYTVLNDLWGGPGPGFIAVY	FIA	VYY	0.00922	69.63
56	77	22	KEAAAKAKFVAAWTLKAAAAAY	KKE	AYF	0.0092	69.67
93	113	21	EKWKVKAAYIQLRWFFVFAAY	QEK	AYS	0.00916	69.71
168	188	21	AVYGPGPGAFIAVYYTVLNDL	IAV	DLW	0.00916	69.75
226	247	22	KAKFVAAWTLKAAAKKTGALLA	KKA	LAA	0.00912	69.79
227	249	23	AKFVAAWTLKAAAKKTGALLAAG	KAK	AGA	0.00911	69.83
77	97	21	YFIGGVSLGIAAYRMQEKWKV	AYF	KVK	0.0091	69.86
121	141	21	FVAAYLRWFFVFSTAAYMQEK	LFV	EKW	0.00909	69.9
214	236	23	TERMQEKWKVKKKAKFVAAWTLK	KTE	LKA	0.00898	69.94

184	204	21	VLNDLWGGPGPGFIAVYYTVL	TVL	VLN	0.00896	69.98
3	15	13	PHALEAAAKGIIN	PPH	INT	0.00889	70.02
147	168	22	AAYRETTEAAIIAAYIGAAFIA	LAA	IAV	0.00889	70.06
210	231	22	NKKKTERMQEKWKVKKKAKFVA	GNK	VAA	0.00889	70.06
182	202	21	YTVLNDLWGGPGPGFIAVYYT	YYT	YTV	0.00888	70.14
209	231	23	GNKKKTERMQEKWKVKKKAKFVA	WGN	VAA	0.00879	70.17
228	249	22	KFVAAWTLKAAAKKTGALLAAG	AKF	AGA	0.00879	70.21
167	188	22	IAVYGPGPGAFIAVYYTVLNDL	FIA	DLW	0.00878	70.25
153	173	21	TEAAIIAAYIGAAFIAVYGPG	TTE	PGP	0.00877	70.29
230	250	21	VAAWTLKAAAKKTGALLAAGA	FVA	GAA	0.00874	70.33
152	172	21	TTEAAIIAAYIGAAFIAVYGP	ETT	GPG	0.00872	70.37
175	194	20	GAFIAVYYTVLNDLWGGPGP	PGA	GPG	0.0087	70.41
222	243	22	KVKKKAKFVAAWTLKAAAKKTG	WKV	TGA	0.00866	70.44
54	75	22	RKKEAAAKAKFVAAWTLKAAAA	RRK	AAA	0.00862	70.48
181	199	19	YYTVLNDLWGGPGPGFIAV	VYY	AVY	0.00859	70.52
39	60	22	EQIGKCSTRGRKCCRRKKEAAA	EEQ	AAK	0.00854	70.56
197	219	23	IAVYYTVLNDLWGNKKKTERMQE	FIA	QEK	0.00854	70.6
5	25	21	ALEAAAKGIINTLQKYYCRVR	HAL	VRG	0.00852	70.64
139	158	20	QEKWKVKLAAYRETTEAAII	MQE	IIA	0.00849	70.68
174	187	14	PGAFIAVYYTVLND	GPG	NDL	0.00848	70.72
50	62	13	KCCRRKKEAAAKA	RKC	KAK	0.00848	70.75
204	224	21	LNDLWGNKKKTERMQEKWKVK	VLN	VKK	0.00848	70.79
81	101	21	GVSLGIAAYRMQEKWKVKAAY	GGV	AYI	0.00846	70.83
217	237	21	MQEKWKVKKKAKFVAAWTLKA	RMQ	KAA	0.00845	70.87
172	187	16	PGPGAFIAVYYTVLND	GPG	NDL	0.00842	70.91
95	114	20	WKVKAAYIQLRWFFVFAAYS	KWK	YSY	0.00842	70.95
129	149	21	FFVFSTAAYMQEKWKVKLAAY	WFF	AYR	0.0084	70.99
102	116	15	IQLRWFFVFAAYSYC	YIQ	YCL	0.00839	71.03
96	115	20	KVKAAYIQLRWFFVFAAYSY	WKV	SYC	0.00835	71.06
47	68	22	RGRKCCRRKKEAAAKAKFVAAW	TRG	AWT	0.00835	71.1
214	234	21	TERMQEKWKVKKKAKFVAAWT	KTE	WTL	0.00834	71.14
124	142	19	AYLRWFFVFSTAAYMQEKW	AAY	KWK	0.00831	71.18
177	196	20	FIAVYYTVLNDLWGGPGPGF	AFI	GFI	0.0083	71.22
19	40	22	KYYCRVRGGRCAVLSCLPKEEQ	QKY	EQI	0.00829	71.26
64	85	22	FVAAWTLKAAAAAYFIGGVSLG	KFV	LGI	0.00829	71.3
180	199	20	VYYTVLNDLWGGPGPGFIAV	AVY	AVY	0.00825	71.33
23	41	19	RVRGGRCAVLSCLPKEEQI	CRV	QIG	0.00821	71.37
129	150	22	FFVFSTAAYMQEKWKVKLAAYR	WFF	YRE	0.00821	71.41
190	211	22	GGPGPGFIAVYYTVLNDLWGNK	WGG	NKK	0.00818	71.45
134	155	22	TAAYMQEKWKVKLAAYRETTEA	STA	EAA	0.00816	71.49
224	244	21	KKKAKFVAAWTLKAAAKKTGA	VKK	GAL	0.00816	71.53
195	216	22	GFIAVYYTVLNDLWGNKKKTER	PGF	ERM	0.00808	71.57
67	87	21	AWTLKAAAAAYFIGGVSLGIA	AAW	IAA	0.00807	71.61
89	110	22	YRMQEKWKVKAAYIQLRWFFVF	AYR	VFA	0.00805	71.64
227	248	22	AKFVAAWTLKAAAKKTGALLAA	KAK	AAG	0.00804	71.68
179	200	22	AVYYTVLNDLWGGPGPGFIAVY	IAV	VYY	0.00803	71.72
11	31	21	KGIINTLQKYYCRVRGGRCAV	AKG	AVL	0.00802	71.76
172	185	14	PGPGAFIAVYYTVL	GPG	VLN	0.00802	71.8
65	84	20	VAAWTLKAAAAAYFIGGVSL	FVA	SLG	0.00799	71.84

159	178	20	AAYIGAAFIAVYGPGPGAFI	IAA	FIA	0.00799	71.88
69	89	21	TLKAAAAAYFIGGVSLGIAAY	WTL	AYR	0.00798	71.91
9	22	14	AAKGIINTLQKYYC	AAA	YCR	0.00798	71.95
127	148	22	RWFFVFSTAAYMQEKWKVKLAA	LRW	AAY	0.00795	71.99
90	111	22	RMQEKWKVKAAYIQLRWFFVFA	YRM	FAA	0.00795	72.03
7	27	21	EAAAKGIINTLQKYYCRVRGG	LEA	GGR	0.00788	72.07
49	71	23	RKCCRRKKEAAAKAKFVAAWTLK	GRK	LKA	0.00785	72.11
90	110	21	RMQEKWKVKAAYIQLRWFFVF	YRM	VFA	0.00782	72.15
211	232	22	KKKTERMQEKWKVKKKAKFVAA	NKK	AAW	0.00781	72.19
144	165	22	VKLAAYRETTEAAIIAAYIGAA	KVK	AAF	0.0078	72.22
105	125	21	RWFFVFAAYSYCLYWLFVAAY	LRW	AYL	0.00775	72.26
50	65	16	KCCRRKKEAAAKAKFV	RKC	FVA	0.00775	72.3
68	90	23	WTLKAAAAAYFIGGVSLGIAAYR	AWT	YRM	0.00775	72.34
157	177	21	IIAAYIGAAFIAVYGPGPGAF	AII	AFI	0.00774	72.38
157	176	20	IIAAYIGAAFIAVYGPGPGA	AII	GAF	0.00773	72.42
8	22	15	AAAKGIINTLQKYYC	EAA	YCR	0.00773	72.46
105	127	23	RWFFVFAAYSYCLYWLFVAAYLR	LRW	LRW	0.00772	72.5
150	169	20	RETTEAAIIAAYIGAAFIAV	YRE	AVY	0.00772	72.53
4	21	18	HALEAAAKGIINTLQKYY	PHA	YYC	0.00771	72.57
111	132	22	AAYSYCLYWLFVAAYLRWFFVF	FAA	VFS	0.00769	72.61
225	247	23	KKAKFVAAWTLKAAAKKTGALLA	KKK	LAA	0.00768	72.65
202	224	23	TVLNDLWGNKKKTERMQEKWKVK	YTV	VKK	0.00768	72.69
204	221	18	LNDLWGNKKKTERMQEKW	VLN	KWK	0.00768	72.73
38	51	14	EEQIGKCSTRGRKC	KEE	KCC	0.00768	72.77
76	98	23	AYFIGGVSLGIAAYRMQEKWKVK	AAY	VKA	0.00766	72.8
123	145	23	AAYLRWFFVFSTAAYMQEKWKVK	VAA	VKL	0.00766	72.8
101	123	23	YIQLRWFFVFAAYSYCLYWLFVA	AYI	VAA	0.00765	72.88
188	207	20	LWGGPGPGFIAVYYTVLNDL	DLW	DLW	0.00765	72.92
184	203	20	VLNDLWGGPGPGFIAVYYTV	TVL	TVL	0.00763	72.96
148	167	20	AYRETTEAAIIAAYIGAAFI	AAY	FIA	0.00763	73.0
173	194	22	GPGAFIAVYYTVLNDLWGGPGP	PGP	GPG	0.00755	73.04
101	121	21	YIQLRWFFVFAAYSYCLYWLF	AYI	LFV	0.00753	73.08
69	86	18	TLKAAAAAYFIGGVSLGI	WTL	GIA	0.0075	73.11
14	30	17	INTLQKYYCRVRGGRCA	IIN	CAV	0.00748	73.15
132	152	21	FSTAAYMQEKWKVKLAAYRET	VFS	ETT	0.00747	73.19
45	66	22	STRGRKCCRRKKEAAAKAKFVA	CST	VAA	0.00746	73.23
72	94	23	AAAAAYFIGGVSLGIAAYRMQEK	KAA	EKW	0.00746	73.27
124	144	21	AYLRWFFVFSTAAYMQEKWKV	AAY	KVK	0.00745	73.31
130	151	22	FVFSTAAYMQEKWKVKLAAYRE	FFV	RET	0.00744	73.35
198	219	22	AVYYTVLNDLWGNKKKTERMQE	IAV	QEK	0.00744	73.38
7	28	22	EAAAKGIINTLQKYYCRVRGGR	LEA	GRC	0.00741	73.42
195	214	20	GFIAVYYTVLNDLWGNKKKT	PGF	KTE	0.0074	73.46
94	113	20	KWKVKAAYIQLRWFFVFAAY	EKW	AYS	0.00739	73.5
151	172	22	ETTEAAIIAAYIGAAFIAVYGP	RET	GPG	0.00736	73.54
142	161	20	WKVKLAAYRETTEAAIIAAY	KWK	AYI	0.00734	73.58
128	148	21	WFFVFSTAAYMQEKWKVKLAA	RWF	AAY	0.00734	73.62
212	233	22	KKTERMQEKWKVKKKAKFVAAW	KKK	AWT	0.0073	73.66
38	60	23	EEQIGKCSTRGRKCCRRKKEAAA	KEE	AAK	0.00729	73.69
49	69	21	RKCCRRKKEAAAKAKFVAAWT	GRK	WTL	0.00729	73.73

83	101	19	SLGIAAYRMQEKWKVKAAY	VSL	AYI	0.00728	73.77
88	108	21	AYRMQEKWKVKAAYIQLRWFF	AAY	FFV	0.00726	73.81
71	92	22	KAAAAAYFIGGVSLGIAAYRMQ	LKA	MQE	0.00723	73.85
46	67	22	TRGRKCCRRKKEAAAKAKFVAA	STR	AAW	0.00718	73.89
102	124	23	IQLRWFFVFAAYSYCLYWLFVAA	YIQ	AAY	0.00718	73.93
118	137	20	YWLFVAAYLRWFFVFSTAAY	LYW	AYM	0.00717	73.97
229	251	23	FVAAWTLKAAAKKTGALLAAGAA	KFV	AAA	0.00715	74.0
220	241	22	KWKVKKKAKFVAAWTLKAAAKK	EKW	KKT	0.00713	74.04
226	246	21	KAKFVAAWTLKAAAKKTGALL	KKA	LLA	0.00713	74.08
145	164	20	KLAAYRETTEAAIIAAYIGA	VKL	GAA	0.00712	74.12
10	31	22	AKGIINTLQKYYCRVRGGRCAV	AAK	AVL	0.00711	74.16
53	74	22	RRKKEAAAKAKFVAAWTLKAAA	CRR	AAA	0.00711	74.2
217	238	22	MQEKWKVKKKAKFVAAWTLKAA	RMQ	AAA	0.00711	74.2
78	99	22	FIGGVSLGIAAYRMQEKWKVKA	YFI	KAA	0.00711	74.27
57	78	22	EAAAKAKFVAAWTLKAAAAAYF	KEA	YFI	0.0071	74.31
157	179	23	IIAAYIGAAFIAVYGPGPGAFIA	AII	IAV	0.00709	74.35
182	201	20	YTVLNDLWGGPGPGFIAVYY	YYT	YYT	0.00706	74.39
202	221	20	TVLNDLWGNKKKTERMQEKW	YTV	KWK	0.00706	74.39
156	176	21	AIIAAYIGAAFIAVYGPGPGA	AAI	GAF	0.00701	74.47
153	171	19	TEAAIIAAYIGAAFIAVYG	TTE	YGP	0.007	74.51
17	38	22	LQKYYCRVRGGRCAVLSCLPKE	TLQ	KEE	0.00699	74.55
59	81	23	AAKAKFVAAWTLKAAAAAYFIGG	AAA	GGV	0.00698	74.58
174	186	13	PGAFIAVYYTVLN	GPG	LND	0.00696	74.62
186	207	22	NDLWGGPGPGFIAVYYTVLNDL	LND	DLW	0.00696	74.66
3	16	14	PHALEAAAKGIINT	PPH	NTL	0.00688	74.7
215	234	20	ERMQEKWKVKKKAKFVAAWT	TER	WTL	0.00684	74.74
215	236	22	ERMQEKWKVKKKAKFVAAWTLK	TER	LKA	0.00683	74.78
200	220	21	YYTVLNDLWGNKKKTERMQEK	VYY	EKW	0.00683	74.82
176	198	23	AFIAVYYTVLNDLWGGPGPGFIA	GAF	IAV	0.00682	74.85
36	54	19	PKEEQIGKCSTRGRKCCRR	LPK	RRK	0.00678	74.89
210	230	21	NKKKTERMQEKWKVKKKAKFV	GNK	FVA	0.00677	74.93
144	162	19	VKLAAYRETTEAAIIAAYI	KVK	YIG	0.00676	74.97
195	215	21	GFIAVYYTVLNDLWGNKKKTE	PGF	TER	0.00674	75.01
216	238	23	RMQEKWKVKKKAKFVAAWTLKAA	ERM	AAA	0.00673	75.05
101	117	17	YIQLRWFFVFAAYSYCL	AYI	CLY	0.0067	75.09
17	39	23	LQKYYCRVRGGRCAVLSCLPKEE	TLQ	EEQ	0.00669	75.13
216	237	22	RMQEKWKVKKKAKFVAAWTLKA	ERM	KAA	0.00668	75.16
108	128	21	FVFAAYSYCLYWLFVAAYLRW	FFV	RWF	0.00668	75.2
22	36	15	CRVRGGRCAVLSCLP	YCR	LPK	0.00664	75.24
125	142	18	YLRWFFVFSTAAYMQEKW	AYL	KWK	0.00661	75.28
101	116	16	YIQLRWFFVFAAYSYC	AYI	YCL	0.00661	75.32
218	239	22	QEKWKVKKKAKFVAAWTLKAAA	MQE	AAK	0.00661	75.36
13	30	18	IINTLQKYYCRVRGGRCA	GII	CAV	0.00659	75.4
204	223	20	LNDLWGNKKKTERMQEKWKV	VLN	KVK	0.00659	75.44
223	244	22	VKKKAKFVAAWTLKAAAKKTGA	KVK	GAL	0.00658	75.47
160	181	22	AYIGAAFIAVYGPGPGAFIAVY	AAY	VYY	0.00657	75.51
134	156	23	TAAYMQEKWKVKLAAYRETTEAA	STA	AAI	0.00656	75.55
99	119	21	AAYIQLRWFFVFAAYSYCLYW	KAA	YWL	0.00655	75.59
139	160	22	QEKWKVKLAAYRETTEAAIIAA	MQE	AAY	0.00653	75.63

4	25	22	HALEAAAKGIINTLQKYYCRVR	PHA	VRG	0.00647	75.67
106	125	20	WFFVFAAYSYCLYWLFVAAY	RWF	AYL	0.00644	75.71
179	199	21	AVYYTVLNDLWGGPGPGFIAV	IAV	AVY	0.00641	75.74
184	205	22	VLNDLWGGPGPGFIAVYYTVLN	TVL	LND	0.00641	75.78
141	163	23	KWKVKLAAYRETTEAAIIAAYIG	EKW	IGA	0.00641	75.82
30	45	16	AVLSCLPKEEQIGKCS	CAV	CST	0.00635	75.86
165	185	21	AFIAVYGPGPGAFIAVYYTVL	AAF	VLN	0.00634	75.9
156	177	22	AIIAAYIGAAFIAVYGPGPGAF	AAI	AFI	0.00632	75.94
31	45	15	VLSCLPKEEQIGKCS	AVL	CST	0.00631	75.98
172	188	17	PGPGAFIAVYYTVLNDL	GPG	DLW	0.0063	76.02
194	210	17	PGFIAVYYTVLNDLWGN	GPG	GNK	0.0063	76.02
6	28	23	LEAAAKGIINTLQKYYCRVRGGR	ALE	GRC	0.0063	76.09
82	102	21	VSLGIAAYRMQEKWKVKAAYI	GVS	YIQ	0.00625	76.13
129	151	23	FFVFSTAAYMQEKWKVKLAAYRE	WFF	RET	0.00624	76.17
113	134	22	YSYCLYWLFVAAYLRWFFVFST	AYS	STA	0.00624	76.21
170	190	21	YGPGPGAFIAVYYTVLNDLWG	VYG	WGG	0.0062	76.25
75	95	21	AAYFIGGVSLGIAAYRMQEKW	AAA	KWK	0.0062	76.29
177	197	21	FIAVYYTVLNDLWGGPGPGFI	AFI	FIA	0.0062	76.32
231	252	22	AAWTLKAAAKKTGALLAAGAAA	VAA	AAK	0.00616	76.36
178	199	22	IAVYYTVLNDLWGGPGPGFIAV	FIA	AVY	0.00614	76.4
104	117	14	LRWFFVFAAYSYCL	QLR	CLY	0.00614	76.44
27	49	23	GRCAVLSCLPKEEQIGKCSTRGR	GGR	GRK	0.00614	76.48
56	78	23	KEAAAKAKFVAAWTLKAAAAAYF	KKE	YFI	0.00613	76.52
61	83	23	KAKFVAAWTLKAAAAAYFIGGVS	AKA	VSL	0.00612	76.56
10	22	13	AKGIINTLQKYYC	AAK	YCR	0.00612	76.6
211	233	23	KKKTERMQEKWKVKKKAKFVAAW	NKK	AWT	0.00611	76.63
158	178	21	IAAYIGAAFIAVYGPGPGAFI	IIA	FIA	0.00611	76.67
232	253	22	AWTLKAAAKKTGALLAAGAAAK	AAW	AKK	0.0061	76.71
88	107	20	AYRMQEKWKVKAAYIQLRWF	AAY	WFF	0.00608	76.75
105	117	13	RWFFVFAAYSYCL	LRW	CLY	0.00607	76.79
174	190	17	PGAFIAVYYTVLNDLWG	GPG	WGG	0.00606	76.83
190	212	23	GGPGPGFIAVYYTVLNDLWGNKK	WGG	KKK	0.00603	76.87
187	209	23	DLWGGPGPGFIAVYYTVLNDLWG	NDL	WGN	0.00602	76.91
97	118	22	VKAAYIQLRWFFVFAAYSYCLY	KVK	LYW	0.00602	76.94
126	147	22	LRWFFVFSTAAYMQEKWKVKLA	YLR	LAA	0.00597	76.98
196	217	22	FIAVYYTVLNDLWGNKKKTERM	GFI	RMQ	0.00596	77.02
106	127	22	WFFVFAAYSYCLYWLFVAAYLR	RWF	LRW	0.00595	77.06
153	174	22	TEAAIIAAYIGAAFIAVYGPGP	TTE	GPG	0.00594	77.1
71	93	23	KAAAAAYFIGGVSLGIAAYRMQE	LKA	QEK	0.00594	77.14
64	82	19	FVAAWTLKAAAAAYFIGGV	KFV	GVS	0.00593	77.18
193	214	22	GPGFIAVYYTVLNDLWGNKKKT	PGP	KTE	0.00592	77.21
194	206	13	PGFIAVYYTVLND	GPG	NDL	0.00589	77.25
3	17	15	PHALEAAAKGIINTL	PPH	TLQ	0.00587	77.29
112	133	22	AYSYCLYWLFVAAYLRWFFVFS	AAY	FST	0.00586	77.33
145	165	21	KLAAYRETTEAAIIAAYIGAA	VKL	AAF	0.00586	77.37
85	106	22	GIAAYRMQEKWKVKAAYIQLRW	LGI	RWF	0.00583	77.41
193	215	23	GPGFIAVYYTVLNDLWGNKKKTE	PGP	TER	0.00581	77.45
126	146	21	LRWFFVFSTAAYMQEKWKVKL	YLR	KLA	0.0058	77.49
78	100	23	FIGGVSLGIAAYRMQEKWKVKAA	YFI	AAY	0.0058	77.52

147	167	21	AAYRETTEAAIIAAYIGAAFI	LAA	FIA	0.00578	77.56
58	80	23	AAAKAKFVAAWTLKAAAAAYFIG	EAA	IGG	0.00578	77.6
59	79	21	AAKAKFVAAWTLKAAAAAYFI	AAA	FIG	0.00577	77.64
207	227	21	LWGNKKKTERMQEKWKVKKKA	DLW	KAK	0.00576	77.68
131	152	22	VFSTAAYMQEKWKVKLAAYRET	FVF	ETT	0.00576	77.72
43	60	18	KCSTRGRKCCRRKKEAAA	GKC	AAK	0.00576	77.76
91	112	22	MQEKWKVKAAYIQLRWFFVFAA	RMQ	AAY	0.00576	77.79
82	104	23	VSLGIAAYRMQEKWKVKAAYIQL	GVS	QLR	0.00575	77.83
199	220	22	VYYTVLNDLWGNKKKTERMQEK	AVY	EKW	0.00574	77.87
163	183	21	GAAFIAVYGPGPGAFIAVYYT	IGA	YTV	0.00571	77.91
55	76	22	KKEAAAKAKFVAAWTLKAAAAA	RKK	AAY	0.0057	77.95
43	61	19	KCSTRGRKCCRRKKEAAAK	GKC	AKA	0.00569	77.99
45	65	21	STRGRKCCRRKKEAAAKAKFV	CST	FVA	0.00568	78.03
125	144	20	YLRWFFVFSTAAYMQEKWKV	AYL	KVK	0.00567	78.07
37	53	17	KEEQIGKCSTRGRKCCR	PKE	CRR	0.00566	78.1
205	227	23	NDLWGNKKKTERMQEKWKVKKKA	LND	KAK	0.00565	78.14
185	204	20	LNDLWGGPGPGFIAVYYTVL	VLN	VLN	0.00564	78.18
46	68	23	TRGRKCCRRKKEAAAKAKFVAAW	STR	AWT	0.00562	78.22
105	126	22	RWFFVFAAYSYCLYWLFVAAYL	LRW	YLR	0.0056	78.26
207	228	22	LWGNKKKTERMQEKWKVKKKAK	DLW	AKF	0.0056	78.3
6	27	22	LEAAAKGIINTLQKYYCRVRGG	ALE	GGR	0.0056	78.34
209	230	22	GNKKKTERMQEKWKVKKKAKFV	WGN	FVA	0.00559	78.38
10	32	23	AKGIINTLQKYYCRVRGGRCAVL	AAK	VLS	0.00559	78.41
93	114	22	EKWKVKAAYIQLRWFFVFAAYS	QEK	YSY	0.00558	78.45
192	204	13	PGPGFIAVYYTVL	GPG	VLN	0.00557	78.49
230	252	23	VAAWTLKAAAKKTGALLAAGAAA	FVA	AAK	0.00555	78.53
202	223	22	TVLNDLWGNKKKTERMQEKWKV	YTV	KVK	0.00554	78.57
67	88	22	AWTLKAAAAAYFIGGVSLGIAA	AAW	AAY	0.00552	78.61
176	196	21	AFIAVYYTVLNDLWGGPGPGF	GAF	GFI	0.00551	78.65
19	34	16	KYYCRVRGGRCAVLSC	QKY	SCL	0.00544	78.68
76	95	20	AYFIGGVSLGIAAYRMQEKW	AAY	KWK	0.00543	78.72
100	119	20	AYIQLRWFFVFAAYSYCLYW	AAY	YWL	0.00543	78.72
123	142	20	AAYLRWFFVFSTAAYMQEKW	VAA	KWK	0.00543	78.72
165	184	20	AFIAVYGPGPGAFIAVYYTV	AAF	TVL	0.0054	78.84
64	84	21	FVAAWTLKAAAAAYFIGGVSL	KFV	SLG	0.00537	78.88
120	141	22	LFVAAYLRWFFVFSTAAYMQEK	WLF	EKW	0.00536	78.92
114	135	22	SYCLYWLFVAAYLRWFFVFSTA	YSY	TAA	0.00531	78.96
149	170	22	YRETTEAAIIAAYIGAAFIAVY	AYR	VYG	0.0053	78.99
229	250	22	FVAAWTLKAAAKKTGALLAAGA	KFV	GAA	0.00529	79.03
111	133	23	AAYSYCLYWLFVAAYLRWFFVFS	FAA	FST	0.00527	79.07
11	32	22	KGIINTLQKYYCRVRGGRCAVL	AKG	VLS	0.00527	79.11
160	180	21	AYIGAAFIAVYGPGPGAFIAV	AAY	AVY	0.00525	79.15
75	97	23	AAYFIGGVSLGIAAYRMQEKWKV	AAA	KVK	0.00524	79.19
15	36	22	NTLQKYYCRVRGGRCAVLSCLP	INT	LPK	0.0052	79.23
106	126	21	WFFVFAAYSYCLYWLFVAAYL	RWF	YLR	0.00517	79.26
146	166	21	LAAYRETTEAAIIAAYIGAAF	KLA	AFI	0.00517	79.3
224	245	22	KKKAKFVAAWTLKAAAKKTGAL	VKK	ALL	0.00513	79.34
11	33	23	KGIINTLQKYYCRVRGGRCAVLS	AKG	LSC	0.00513	79.38
164	185	22	AAFIAVYGPGPGAFIAVYYTVL	GAA	VLN	0.00511	79.42

66	87	22	AAWTLKAAAAAYFIGGVSLGIA	VAA	IAA	0.0051	79.46
143	164	22	KVKLAAYRETTEAAIIAAYIGA	WKV	GAA	0.00508	79.5
2	21	20	PPHALEAAAKGIINTLQKYY	APP	YYC	0.00508	79.54
7	22	16	EAAAKGIINTLQKYYC	LEA	YCR	0.00506	79.57
225	246	22	KKAKFVAAWTLKAAAKKTGALL	KKK	LLA	0.00502	79.61
94	114	21	KWKVKAAYIQLRWFFVFAAYS	EKW	YSY	0.005	79.65
110	130	21	FAAYSYCLYWLFVAAYLRWFF	VFA	FFV	0.00499	79.69
118	139	22	YWLFVAAYLRWFFVFSTAAYMQ	LYW	MQE	0.00499	79.73
223	245	23	VKKKAKFVAAWTLKAAAKKTGAL	KVK	ALL	0.00495	79.77
20	42	23	YYCRVRGGRCAVLSCLPKEEQIG	KYY	IGK	0.00495	79.81
218	240	23	QEKWKVKKKAKFVAAWTLKAAAK	MQE	AKK	0.00494	79.85
36	55	20	PKEEQIGKCSTRGRKCCRRK	LPK	RKK	0.00494	79.88
166	187	22	FIAVYGPGPGAFIAVYYTVLND	AFI	NDL	0.00493	79.92
150	170	21	RETTEAAIIAAYIGAAFIAVY	YRE	VYG	0.00491	79.96
203	225	23	VLNDLWGNKKKTERMQEKWKVKK	TVL	KKK	0.00491	80.0
99	120	22	AAYIQLRWFFVFAAYSYCLYWL	KAA	WLF	0.0049	80.04
86	108	23	IAAYRMQEKWKVKAAYIQLRWFF	GIA	FFV	0.0049	80.08
121	143	23	FVAAYLRWFFVFSTAAYMQEKWK	LFV	WKV	0.00487	80.12
53	75	23	RRKKEAAAKAKFVAAWTLKAAAA	CRR	AAA	0.00487	80.15
101	122	22	YIQLRWFFVFAAYSYCLYWLFV	AYI	FVA	0.00487	80.19
214	235	22	TERMQEKWKVKKKAKFVAAWTL	KTE	TLK	0.00487	80.23
140	161	22	EKWKVKLAAYRETTEAAIIAAY	QEK	AYI	0.00486	80.27
196	218	23	FIAVYYTVLNDLWGNKKKTERMQ	GFI	MQE	0.00484	80.31
164	184	21	AAFIAVYGPGPGAFIAVYYTV	GAA	TVL	0.00483	80.35
18	38	21	QKYYCRVRGGRCAVLSCLPKE	LQK	KEE	0.00483	80.39
103	115	13	QLRWFFVFAAYSY	IQL	SYC	0.00481	80.43
198	220	23	AVYYTVLNDLWGNKKKTERMQEK	IAV	EKW	0.00481	80.46
189	210	22	WGGPGPGFIAVYYTVLNDLWGN	LWG	GNK	0.0048	80.5
54	76	23	RKKEAAAKAKFVAAWTLKAAAAA	RRK	AAY	0.00479	80.54
145	162	18	KLAAYRETTEAAIIAAYI	VKL	YIG	0.00478	80.58
183	204	22	TVLNDLWGGPGPGFIAVYYTVL	YTV	VLN	0.00474	80.62
146	168	23	LAAYRETTEAAIIAAYIGAAFIA	KLA	IAV	0.00473	80.66
159	181	23	AAYIGAAFIAVYGPGPGAFIAVY	IAA	VYY	0.00471	80.7
107	128	22	FFVFAAYSYCLYWLFVAAYLRW	WFF	RWF	0.00467	80.74
185	203	19	LNDLWGGPGPGFIAVYYTV	VLN	TVL	0.00467	80.77
88	109	22	AYRMQEKWKVKAAYIQLRWFFV	AAY	FVF	0.00465	80.81
118	138	21	YWLFVAAYLRWFFVFSTAAYM	LYW	YMQ	0.00465	80.85
231	253	23	AAWTLKAAAKKTGALLAAGAAAK	VAA	AKK	0.00461	80.89
65	87	23	VAAWTLKAAAAAYFIGGVSLGIA	FVA	IAA	0.00459	80.93
48	69	22	GRKCCRRKKEAAAKAKFVAAWT	RGR	WTL	0.00454	80.97
163	182	20	GAAFIAVYGPGPGAFIAVYY	IGA	YYT	0.00454	81.01
122	142	21	VAAYLRWFFVFSTAAYMQEKW	FVA	KWK	0.00454	81.04
165	186	22	AFIAVYGPGPGAFIAVYYTVLN	AAF	LND	0.00454	81.08
100	120	21	AYIQLRWFFVFAAYSYCLYWL	AAY	WLF	0.00451	81.12
143	165	23	KVKLAAYRETTEAAIIAAYIGAA	WKV	AAF	0.0045	81.16
119	137	19	WLFVAAYLRWFFVFSTAAY	YWL	AYM	0.00449	81.2
183	203	21	TVLNDLWGGPGPGFIAVYYTV	YTV	TVL	0.00449	81.24
201	221	21	YTVLNDLWGNKKKTERMQEKW	YYT	KWK	0.00449	81.24
185	205	21	LNDLWGGPGPGFIAVYYTVLN	VLN	LND	0.00448	81.32

221	243	23	WKVKKKAKFVAAWTLKAAAKKTG	KWK	TGA	0.00445	81.35
226	248	23	KAKFVAAWTLKAAAKKTGALLAA	KKA	AAG	0.00445	81.39
215	235	21	ERMQEKWKVKKKAKFVAAWTL	TER	TLK	0.00444	81.43
9	31	23	AAKGIINTLQKYYCRVRGGRCAV	AAA	AVL	0.00442	81.47
164	186	23	AAFIAVYGPGPGAFIAVYYTVLN	GAA	LND	0.00437	81.51
141	161	21	KWKVKLAAYRETTEAAIIAAY	EKW	AYI	0.00436	81.55
87	108	22	AAYRMQEKWKVKAAYIQLRWFF	IAA	FFV	0.00435	81.59
39	51	13	EQIGKCSTRGRKC	EEQ	KCC	0.00429	81.62
77	99	23	YFIGGVSLGIAAYRMQEKWKVKA	AYF	KAA	0.00428	81.66
63	85	23	KFVAAWTLKAAAAAYFIGGVSLG	AKF	LGI	0.00428	81.7
221	242	22	WKVKKKAKFVAAWTLKAAAKKT	KWK	KTG	0.00428	81.74
113	135	23	YSYCLYWLFVAAYLRWFFVFSTA	AYS	TAA	0.00427	81.78
76	97	22	AYFIGGVSLGIAAYRMQEKWKV	AAY	KVK	0.00427	81.82
123	144	22	AAYLRWFFVFSTAAYMQEKWKV	VAA	KVK	0.00427	81.82
151	173	23	ETTEAAIIAAYIGAAFIAVYGPG	RET	PGP	0.00426	81.9
70	91	22	LKAAAAAYFIGGVSLGIAAYRM	TLK	RMQ	0.00426	81.93
208	229	22	WGNKKKTERMQEKWKVKKKAKF	LWG	KFV	0.00426	81.97
49	70	22	RKCCRRKKEAAAKAKFVAAWTL	GRK	TLK	0.00425	82.01
6	22	17	LEAAAKGIINTLQKYYC	ALE	YCR	0.00424	82.05
21	37	17	YCRVRGGRCAVLSCLPK	YYC	PKE	0.00423	82.09
152	173	22	TTEAAIIAAYIGAAFIAVYGPG	ETT	PGP	0.00422	82.13
20	34	15	YYCRVRGGRCAVLSC	KYY	SCL	0.0042	82.17
38	52	15	EEQIGKCSTRGRKCC	KEE	CCR	0.0042	82.21
110	129	20	FAAYSYCLYWLFVAAYLRWF	VFA	WFF	0.00418	82.24
66	88	23	AAWTLKAAAAAYFIGGVSLGIAA	VAA	AAY	0.00417	82.28
169	190	22	VYGPGPGAFIAVYYTVLNDLWG	AVY	WGG	0.00413	82.32
90	112	23	RMQEKWKVKAAYIQLRWFFVFAA	YRM	AAY	0.0041	82.36
118	140	23	YWLFVAAYLRWFFVFSTAAYMQE	LYW	QEK	0.0041	82.36
18	30	13	QKYYCRVRGGRCA	LQK	CAV	0.00408	82.44
28	42	15	RCAVLSCLPKEEQIG	GRC	IGK	0.00407	82.48
183	205	23	TVLNDLWGGPGPGFIAVYYTVLN	YTV	LND	0.00406	82.51
192	209	18	PGPGFIAVYYTVLNDLWG	GPG	WGN	0.00405	82.55
194	211	18	PGFIAVYYTVLNDLWGNK	GPG	NKK	0.00405	82.55
87	107	21	AAYRMQEKWKVKAAYIQLRWF	IAA	WFF	0.00405	82.63
14	36	23	INTLQKYYCRVRGGRCAVLSCLP	IIN	LPK	0.00404	82.67
103	119	17	QLRWFFVFAAYSYCLYW	IQL	YWL	0.00402	82.71
189	208	20	WGGPGPGFIAVYYTVLNDLW	LWG	LWG	0.00401	82.75
161	183	23	YIGAAFIAVYGPGPGAFIAVYYT	AYI	YTV	0.00401	82.79
12	30	19	GIINTLQKYYCRVRGGRCA	KGI	CAV	0.00401	82.82
68	89	22	WTLKAAAAAYFIGGVSLGIAAY	AWT	AYR	0.00399	82.86
217	239	23	MQEKWKVKKKAKFVAAWTLKAAA	RMQ	AAK	0.00399	82.9
68	86	19	WTLKAAAAAYFIGGVSLGI	AWT	GIA	0.00398	82.94
95	115	21	WKVKAAYIQLRWFFVFAAYSY	KWK	SYC	0.00398	82.98
112	134	23	AYSYCLYWLFVAAYLRWFFVFST	AAY	STA	0.00396	83.02
130	152	23	FVFSTAAYMQEKWKVKLAAYRET	FFV	ETT	0.00392	83.06
40	62	23	QIGKCSTRGRKCCRRKKEAAAKA	EQI	KAK	0.0039	83.09
18	39	22	QKYYCRVRGGRCAVLSCLPKEE	LQK	EEQ	0.00386	83.13
157	178	22	IIAAYIGAAFIAVYGPGPGAFI	AII	FIA	0.00385	83.17
109	130	22	VFAAYSYCLYWLFVAAYLRWFF	FVF	FFV	0.00385	83.21

152	171	20	TTEAAIIAAYIGAAFIAVYG	ETT	YGP	0.00384	83.25
122	144	23	VAAYLRWFFVFSTAAYMQEKWKV	FVA	KVK	0.00384	83.29
83	105	23	SLGIAAYRMQEKWKVKAAYIQLR	VSL	LRW	0.00384	83.33
114	136	23	SYCLYWLFVAAYLRWFFVFSTAA	YSY	AAY	0.00384	83.37
127	149	23	RWFFVFSTAAYMQEKWKVKLAAY	LRW	AYR	0.00383	83.4
50	67	18	KCCRRKKEAAAKAKFVAA	RKC	AAW	0.00381	83.44
86	107	22	IAAYRMQEKWKVKAAYIQLRWF	GIA	WFF	0.00381	83.48
222	244	23	KVKKKAKFVAAWTLKAAAKKTGA	WKV	GAL	0.0038	83.52
201	223	23	YTVLNDLWGNKKKTERMQEKWKV	YYT	KVK	0.0038	83.56
81	102	22	GVSLGIAAYRMQEKWKVKAAYI	GGV	YIQ	0.0038	83.6
162	184	23	IGAAFIAVYGPGPGAFIAVYYTV	YIG	TVL	0.00378	83.64
156	178	23	AIIAAYIGAAFIAVYGPGPGAFI	AAI	FIA	0.00377	83.68
79	101	23	IGGVSLGIAAYRMQEKWKVKAAY	FIG	AYI	0.00375	83.71
166	188	23	FIAVYGPGPGAFIAVYYTVLNDL	AFI	DLW	0.00375	83.75
99	121	23	AAYIQLRWFFVFAAYSYCLYWLF	KAA	LFV	0.00374	83.79
83	102	20	SLGIAAYRMQEKWKVKAAYI	VSL	YIQ	0.00373	83.83
213	234	22	KTERMQEKWKVKKKAKFVAAWT	KKT	WTL	0.00373	83.87
143	162	20	KVKLAAYRETTEAAIIAAYI	WKV	YIG	0.00373	83.91
184	206	23	VLNDLWGGPGPGFIAVYYTVLND	TVL	NDL	0.00372	83.95
24	46	23	VRGGRCAVLSCLPKEEQIGKCST	RVR	STR	0.00372	83.98
176	197	22	AFIAVYYTVLNDLWGGPGPGFI	GAF	FIA	0.00371	84.02
25	47	23	RGGRCAVLSCLPKEEQIGKCSTR	VRG	TRG	0.00371	84.06
42	64	23	GKCSTRGRKCCRRKKEAAAKAKF	IGK	KFV	0.00367	84.1
200	222	23	YYTVLNDLWGNKKKTERMQEKWK	VYY	WKV	0.00366	84.14
189	211	23	WGGPGPGFIAVYYTVLNDLWGNK	LWG	NKK	0.00364	84.18
22	38	17	CRVRGGRCAVLSCLPKE	YCR	KEE	0.00361	84.22
151	171	21	ETTEAAIIAAYIGAAFIAVYG	RET	YGP	0.00361	84.26
109	129	21	VFAAYSYCLYWLFVAAYLRWF	FVF	WFF	0.00358	84.29
119	139	21	WLFVAAYLRWFFVFSTAAYMQ	YWL	MQE	0.00357	84.33
74	95	22	AAAYFIGGVSLGIAAYRMQEKW	AAA	KWK	0.00355	84.37
25	46	22	RGGRCAVLSCLPKEEQIGKCST	VRG	STR	0.00355	84.41
37	58	22	KEEQIGKCSTRGRKCCRRKKEA	PKE	EAA	0.00354	84.45
26	45	20	GGRCAVLSCLPKEEQIGKCS	RGG	CST	0.00352	84.49
35	52	18	LPKEEQIGKCSTRGRKCC	CLP	CCR	0.00351	84.53
126	148	23	LRWFFVFSTAAYMQEKWKVKLAA	YLR	AAY	0.00349	84.56
96	118	23	KVKAAYIQLRWFFVFAAYSYCLY	WKV	LYW	0.00347	84.6
144	166	23	VKLAAYRETTEAAIIAAYIGAAF	KVK	AFI	0.00347	84.64
155	177	23	AAIIAAYIGAAFIAVYGPGPGAF	EAA	AFI	0.00347	84.64
70	92	23	LKAAAAAYFIGGVSLGIAAYRMQ	TLK	MQE	0.00346	84.72
168	190	23	AVYGPGPGAFIAVYYTVLNDLWG	IAV	WGG	0.00345	84.76
128	150	23	WFFVFSTAAYMQEKWKVKLAAYR	RWF	YRE	0.00345	84.8
152	174	23	TTEAAIIAAYIGAAFIAVYGPGP	ETT	GPG	0.00342	84.84
87	109	23	AAYRMQEKWKVKAAYIQLRWFFV	IAA	FVF	0.00334	84.87
210	232	23	NKKKTERMQEKWKVKKKAKFVAA	GNK	AAW	0.00333	84.91
16	38	23	TLQKYYCRVRGGRCAVLSCLPKE	NTL	KEE	0.00333	84.95
99	116	18	AAYIQLRWFFVFAAYSYC	KAA	YCL	0.00328	84.99
215	237	23	ERMQEKWKVKKKAKFVAAWTLKA	TER	KAA	0.00328	85.03
33	48	16	SCLPKEEQIGKCSTRG	LSC	RGR	0.00327	85.07
47	69	23	RGRKCCRRKKEAAAKAKFVAAWT	TRG	WTL	0.00327	85.11

32	45	14	LSCLPKEEQIGKCS	VLS	CST	0.00326	85.15
182	204	23	YTVLNDLWGGPGPGFIAVYYTVL	YYT	VLN	0.00325	85.18
155	176	22	AAIIAAYIGAAFIAVYGPGPGA	EAA	GAF	0.00321	85.22
110	131	22	FAAYSYCLYWLFVAAYLRWFFV	VFA	FVF	0.0032	85.26
83	104	22	SLGIAAYRMQEKWKVKAAYIQL	VSL	QLR	0.00319	85.3
48	70	23	GRKCCRRKKEAAAKAKFVAAWTL	RGR	TLK	0.00317	85.34
117	137	21	LYWLFVAAYLRWFFVFSTAAY	CLY	AYM	0.00316	85.38
159	180	22	AAYIGAAFIAVYGPGPGAFIAV	IAA	AVY	0.00314	85.42
194	208	15	PGFIAVYYTVLNDLW	GPG	LWG	0.00314	85.45
204	226	23	LNDLWGNKKKTERMQEKWKVKKK	VLN	KKA	0.00314	85.49
69	91	23	TLKAAAAAYFIGGVSLGIAAYRM	WTL	RMQ	0.00314	85.53
88	110	23	AYRMQEKWKVKAAYIQLRWFFVF	AAY	VFA	0.00313	85.57
104	125	22	LRWFFVFAAYSYCLYWLFVAAY	QLR	AYL	0.00312	85.61
92	113	22	QEKWKVKAAYIQLRWFFVFAAY	MQE	AYS	0.00309	85.65
170	189	20	YGPGPGAFIAVYYTVLNDLW	VYG	LWG	0.00308	85.69
84	106	23	LGIAAYRMQEKWKVKAAYIQLRW	SLG	RWF	0.00308	85.73
188	209	22	LWGGPGPGFIAVYYTVLNDLWG	DLW	WGN	0.00308	85.76
39	52	14	EQIGKCSTRGRKCC	EEQ	CCR	0.00307	85.8
138	157	20	MQEKWKVKLAAYRETTEAAI	YMQ	AII	0.00307	85.84
103	120	18	QLRWFFVFAAYSYCLYWL	IQL	WLF	0.00306	85.88
172	184	13	PGPGAFIAVYYTV	GPG	TVL	0.00302	85.92
148	169	22	AYRETTEAAIIAAYIGAAFIAV	AAY	AVY	0.003	85.96
99	117	19	AAYIQLRWFFVFAAYSYCL	KAA	CLY	0.003	86.0
119	138	20	WLFVAAYLRWFFVFSTAAYM	YWL	YMQ	0.003	86.03
5	24	20	ALEAAAKGIINTLQKYYCRV	HAL	RVR	0.00299	86.07
150	172	23	RETTEAAIIAAYIGAAFIAVYGP	YRE	GPG	0.00298	86.11
100	116	17	AYIQLRWFFVFAAYSYC	AAY	YCL	0.00298	86.15
161	182	22	YIGAAFIAVYGPGPGAFIAVYY	AYI	YYT	0.00296	86.19
109	131	23	VFAAYSYCLYWLFVAAYLRWFFV	FVF	FVF	0.00295	86.23
128	149	22	WFFVFSTAAYMQEKWKVKLAAY	RWF	AYR	0.00295	86.27
22	39	18	CRVRGGRCAVLSCLPKEE	YCR	EEQ	0.00294	86.31
63	82	20	KFVAAWTLKAAAAAYFIGGV	AKF	GVS	0.00292	86.34
58	79	22	AAAKAKFVAAWTLKAAAAAYFI	EAA	FIG	0.00291	86.38
33	47	15	SCLPKEEQIGKCSTR	LSC	TRG	0.00289	86.42
100	121	22	AYIQLRWFFVFAAYSYCLYWLF	AAY	LFV	0.00288	86.46
98	119	22	KAAYIQLRWFFVFAAYSYCLYW	VKA	YWL	0.00287	86.5
204	225	22	LNDLWGNKKKTERMQEKWKVKK	VLN	KKK	0.00287	86.54
80	101	22	GGVSLGIAAYRMQEKWKVKAAY	IGG	AYI	0.00286	86.58
212	234	23	KKTERMQEKWKVKKKAKFVAAWT	KKK	WTL	0.00286	86.62
174	189	16	PGAFIAVYYTVLNDLW	GPG	LWG	0.00284	86.65
104	116	13	LRWFFVFAAYSYC	QLR	YCL	0.00284	86.69
93	115	23	EKWKVKAAYIQLRWFFVFAAYSY	QEK	SYC	0.00284	86.73
62	82	21	AKFVAAWTLKAAAAAYFIGGV	KAK	GVS	0.00281	86.77
32	54	23	LSCLPKEEQIGKCSTRGRKCCRR	VLS	RRK	0.00281	86.81
45	67	23	STRGRKCCRRKKEAAAKAKFVAA	CST	AAW	0.00279	86.85
110	132	23	FAAYSYCLYWLFVAAYLRWFFVF	VFA	VFS	0.00276	86.89
170	192	23	YGPGPGAFIAVYYTVLNDLWGGP	VYG	GPG	0.00274	86.92
55	77	23	KKEAAAKAKFVAAWTLKAAAAAY	RKK	AYF	0.00274	86.96
220	242	23	KWKVKKKAKFVAAWTLKAAAKKT	EKW	KTG	0.00274	87.0

228	250	23	KFVAAWTLKAAAKKTGALLAAGA	AKF	GAA	0.00273	87.04
124	146	23	AYLRWFFVFSTAAYMQEKWKVKL	AAY	KLA	0.00272	87.08
104	126	23	LRWFFVFAAYSYCLYWLFVAAYL	QLR	YLR	0.0027	87.12
135	157	23	AAYMQEKWKVKLAAYRETTEAAI	TAA	AII	0.0027	87.16
180	202	23	VYYTVLNDLWGGPGPGFIAVYYT	AVY	YTV	0.00268	87.2
181	202	22	YYTVLNDLWGGPGPGFIAVYYT	VYY	YTV	0.00266	87.23
67	89	23	AWTLKAAAAAYFIGGVSLGIAAY	AAW	AYR	0.00266	87.27
132	153	22	FSTAAYMQEKWKVKLAAYRETT	VFS	TTE	0.00265	87.31
194	212	19	PGFIAVYYTVLNDLWGNKK	GPG	KKK	0.00265	87.35
115	127	13	YCLYWLFVAAYLR	SYC	LRW	0.00265	87.39
50	68	19	KCCRRKKEAAAKAKFVAAW	RKC	AWT	0.00265	87.43
165	187	23	AFIAVYGPGPGAFIAVYYTVLND	AAF	NDL	0.00263	87.47
177	199	23	FIAVYYTVLNDLWGGPGPGFIAV	AFI	AVY	0.00263	87.5
108	130	23	FVFAAYSYCLYWLFVAAYLRWFF	FFV	FFV	0.00262	87.54
142	164	23	WKVKLAAYRETTEAAIIAAYIGA	KWK	GAA	0.00261	87.58
213	235	23	KTERMQEKWKVKKKAKFVAAWTL	KKT	TLK	0.00261	87.62
100	117	18	AYIQLRWFFVFAAYSYCL	AAY	CLY	0.0026	87.66
194	213	20	PGFIAVYYTVLNDLWGNKKK	GPG	KKT	0.0026	87.7
18	40	23	QKYYCRVRGGRCAVLSCLPKEEQ	LQK	EQI	0.00259	87.74
158	180	23	IAAYIGAAFIAVYGPGPGAFIAV	IIA	AVY	0.00259	87.78
146	167	22	LAAYRETTEAAIIAAYIGAAFI	KLA	FIA	0.00257	87.81
182	203	22	YTVLNDLWGGPGPGFIAVYYTV	YYT	TVL	0.00257	87.85
98	120	23	KAAYIQLRWFFVFAAYSYCLYWL	VKA	WLF	0.00257	87.89
67	86	20	AWTLKAAAAAYFIGGVSLGI	AAW	GIA	0.00253	87.93
4	24	21	HALEAAAKGIINTLQKYYCRV	PHA	RVR	0.00252	87.97
154	176	23	EAAIIAAYIGAAFIAVYGPGPGA	TEA	GAF	0.00252	88.01
36	56	21	PKEEQIGKCSTRGRKCCRRKK	LPK	KKE	0.0025	88.05
208	230	23	WGNKKKTERMQEKWKVKKKAKFV	LWG	FVA	0.0025	88.09
28	43	16	RCAVLSCLPKEEQIGK	GRC	GKC	0.00249	88.12
138	159	22	MQEKWKVKLAAYRETTEAAIIA	YMQ	IAA	0.00247	88.16
17	34	18	LQKYYCRVRGGRCAVLSC	TLQ	SCL	0.00247	88.2
224	246	23	KKKAKFVAAWTLKAAAKKTGALL	VKK	LLA	0.00246	88.24
147	169	23	AAYRETTEAAIIAAYIGAAFIAV	LAA	AVY	0.00245	88.28
131	153	23	VFSTAAYMQEKWKVKLAAYRETT	FVF	TTE	0.00245	88.32
119	140	22	WLFVAAYLRWFFVFSTAAYMQE	YWL	QEK	0.00245	88.36
138	158	21	MQEKWKVKLAAYRETTEAAII	YMQ	IIA	0.00244	88.39
5	26	22	ALEAAAKGIINTLQKYYCRVRG	HAL	RGG	0.00243	88.43
73	95	23	AAAAYFIGGVSLGIAAYRMQEKW	AAA	KWK	0.00243	88.47
174	192	19	PGAFIAVYYTVLNDLWGGP	GPG	GPG	0.00242	88.51
187	208	22	DLWGGPGPGFIAVYYTVLNDLW	NDL	LWG	0.00242	88.55
37	59	23	KEEQIGKCSTRGRKCCRRKKEAA	PKE	AAA	0.00242	88.59
3	20	18	PHALEAAAKGIINTLQKY	PPH	KYY	0.00241	88.63
21	36	16	YCRVRGGRCAVLSCLP	YYC	LPK	0.00241	88.67
132	154	23	FSTAAYMQEKWKVKLAAYRETTE	VFS	TEA	0.00241	88.7
13	29	17	IINTLQKYYCRVRGGRC	GII	RCA	0.00241	88.74
62	84	23	AKFVAAWTLKAAAAAYFIGGVSL	KAK	SLG	0.0024	88.78
117	139	23	LYWLFVAAYLRWFFVFSTAAYMQ	CLY	MQE	0.00237	88.82
125	147	23	YLRWFFVFSTAAYMQEKWKVKLA	AYL	LAA	0.00237	88.86
14	29	16	INTLQKYYCRVRGGRC	IIN	RCA	0.00236	88.9

181	201	21	YYTVLNDLWGGPGPGFIAVYY	VYY	YYT	0.00235	88.94
30	44	15	AVLSCLPKEEQIGKC	CAV	KCS	0.00233	88.97
63	84	22	KFVAAWTLKAAAAAYFIGGVSL	AKF	SLG	0.00232	89.01
136	157	22	AYMQEKWKVKLAAYRETTEAAI	AAY	AII	0.00231	89.05
15	29	15	NTLQKYYCRVRGGRC	INT	RCA	0.0023	89.09
37	51	15	KEEQIGKCSTRGRKC	PKE	KCC	0.00229	89.13
169	189	21	VYGPGPGAFIAVYYTVLNDLW	AVY	LWG	0.00228	89.17
92	114	23	QEKWKVKAAYIQLRWFFVFAAYS	MQE	YSY	0.00225	89.21
103	123	21	QLRWFFVFAAYSYCLYWLFVA	IQL	VAA	0.00223	89.25
192	210	19	PGPGFIAVYYTVLNDLWGN	GPG	GNK	0.00223	89.28
100	122	23	AYIQLRWFFVFAAYSYCLYWLFV	AAY	FVA	0.00223	89.32
4	26	23	HALEAAAKGIINTLQKYYCRVRG	PHA	RGG	0.00221	89.36
36	57	22	PKEEQIGKCSTRGRKCCRRKKE	LPK	KEA	0.00221	89.4
27	45	19	GRCAVLSCLPKEEQIGKCS	GGR	CST	0.0022	89.44
17	29	13	LQKYYCRVRGGRC	TLQ	RCA	0.00219	89.48
16	29	14	TLQKYYCRVRGGRC	NTL	RCA	0.00219	89.52
145	166	22	KLAAYRETTEAAIIAAYIGAAF	VKL	AFI	0.00217	89.56
185	206	22	LNDLWGGPGPGFIAVYYTVLND	VLN	NDL	0.00217	89.59
195	217	23	GFIAVYYTVLNDLWGNKKKTERM	PGF	RMQ	0.00217	89.63
57	79	23	EAAAKAKFVAAWTLKAAAAAYFI	KEA	FIG	0.00215	89.67
172	190	19	PGPGAFIAVYYTVLNDLWG	GPG	WGG	0.00215	89.71
97	119	23	VKAAYIQLRWFFVFAAYSYCLYW	KVK	YWL	0.00215	89.75
85	107	23	GIAAYRMQEKWKVKAAYIQLRWF	LGI	WFF	0.00213	89.79
94	115	22	KWKVKAAYIQLRWFFVFAAYSY	EKW	SYC	0.00213	89.83
163	185	23	GAAFIAVYGPGPGAFIAVYYTVL	IGA	VLN	0.00209	89.86
5	23	19	ALEAAAKGIINTLQKYYCR	HAL	CRV	0.00208	89.9
103	121	19	QLRWFFVFAAYSYCLYWLF	IQL	LFV	0.00207	89.94
28	40	13	RCAVLSCLPKEEQ	GRC	EQI	0.00207	89.98
11	30	20	KGIINTLQKYYCRVRGGRCA	AKG	CAV	0.00206	90.02
148	170	23	AYRETTEAAIIAAYIGAAFIAVY	AAY	VYG	0.00206	90.06
43	63	21	KCSTRGRKCCRRKKEAAAKAK	GKC	AKF	0.00204	90.1
108	129	22	FVFAAYSYCLYWLFVAAYLRWF	FFV	WFF	0.00204	90.14
10	30	21	AKGIINTLQKYYCRVRGGRCA	AAK	CAV	0.00203	90.17
138	160	23	MQEKWKVKLAAYRETTEAAIIAA	YMQ	AAY	0.00202	90.21
180	201	22	VYYTVLNDLWGGPGPGFIAVYY	AVY	YYT	0.00198	90.25
136	158	23	AYMQEKWKVKLAAYRETTEAAII	AAY	IIA	0.00197	90.29
106	128	23	WFFVFAAYSYCLYWLFVAAYLRW	RWF	RWF	0.00197	90.33
139	161	23	QEKWKVKLAAYRETTEAAIIAAY	MQE	AYI	0.00196	90.37
125	146	22	YLRWFFVFSTAAYMQEKWKVKL	AYL	KLA	0.00192	90.41
192	208	17	PGPGFIAVYYTVLNDLW	GPG	LWG	0.00192	90.44
33	49	17	SCLPKEEQIGKCSTRGR	LSC	GRK	0.00191	90.48
188	210	23	LWGGPGPGFIAVYYTVLNDLWGN	DLW	GNK	0.00191	90.52
31	44	14	VLSCLPKEEQIGKC	AVL	KCS	0.00191	90.56
20	35	16	YYCRVRGGRCAVLSCL	KYY	CLP	0.00189	90.6
43	62	20	KCSTRGRKCCRRKKEAAAKA	GKC	KAK	0.00189	90.64
91	113	23	MQEKWKVKAAYIQLRWFFVFAAY	RMQ	AYS	0.00187	90.68
117	138	22	LYWLFVAAYLRWFFVFSTAAYM	CLY	YMQ	0.00185	90.72
115	128	14	YCLYWLFVAAYLRW	SYC	RWF	0.00184	90.75
167	189	23	IAVYGPGPGAFIAVYYTVLNDLW	FIA	LWG	0.00183	90.79

19	35	17	KYYCRVRGGRCAVLSCL	QKY	CLP	0.00183	90.83
115	130	16	YCLYWLFVAAYLRWFF	SYC	FFV	0.00182	90.87
4	23	20	HALEAAAKGIINTLQKYYCR	PHA	CRV	0.00181	90.91
20	41	22	YYCRVRGGRCAVLSCLPKEEQI	KYY	QIG	0.0018	90.95
21	33	13	YCRVRGGRCAVLS	YYC	LSC	0.0018	90.99
121	142	22	FVAAYLRWFFVFSTAAYMQEKW	LFV	KWK	0.00179	91.03
34	48	15	CLPKEEQIGKCSTRG	SCL	RGR	0.00179	91.06
52	66	15	CRRKKEAAAKAKFVA	CCR	VAA	0.00179	91.06
26	44	19	GGRCAVLSCLPKEEQIGKC	RGG	KCS	0.00178	91.14
66	86	21	AAWTLKAAAAAYFIGGVSLGI	VAA	GIA	0.00178	91.18
142	162	21	WKVKLAAYRETTEAAIIAAYI	KWK	YIG	0.00178	91.18
174	195	22	PGAFIAVYYTVLNDLWGGPGPG	GPG	PGF	0.00177	91.26
19	41	23	KYYCRVRGGRCAVLSCLPKEEQI	QKY	QIG	0.00177	91.3
22	40	19	CRVRGGRCAVLSCLPKEEQ	YCR	EQI	0.00175	91.33
33	46	14	SCLPKEEQIGKCST	LSC	STR	0.00173	91.37
40	52	13	QIGKCSTRGRKCC	EQI	CCR	0.00173	91.41
107	129	23	FFVFAAYSYCLYWLFVAAYLRWF	WFF	WFF	0.00171	91.45
207	229	23	LWGNKKKTERMQEKWKVKKKAKF	DLW	KFV	0.00169	91.49
18	34	17	QKYYCRVRGGRCAVLSC	LQK	SCL	0.00167	91.53
179	201	23	AVYYYTVLNDLWGGPGPGFIAVYY	IAV	YYT	0.00166	91.57
185	207	23	LNDLWGGPGPGFIAVYYTVLNDL	VLN	DLW	0.00165	91.61
163	184	22	GAAFIAVYGPGPGAFIAVYYTV	IGA	TVL	0.00165	91.64
2	24	23	PPHALEAAAKGIINTLQKYYCRV	APP	RVR	0.00164	91.68
51	66	16	CCRRKKEAAAKAKFVA	KCC	VAA	0.00162	91.72
137	157	21	YMQEKWKVKLAAYRETTEAAI	AYM	AII	0.00162	91.76
192	211	20	PGPGFIAVYYTVLNDLWGNK	GPG	NKK	0.00161	91.8
33	50	18	SCLPKEEQIGKCSTRGRK	LSC	RKC	0.00161	91.84
175	196	22	GAFIAVYYTVLNDLWGGPGPGF	PGA	GFI	0.00161	91.88
168	189	22	AVYGPGPGAFIAVYYTVLNDLW	IAV	LWG	0.00159	91.91
119	141	23	WLFVAAYLRWFFVFSTAAYMQEK	YWL	EKW	0.00158	91.95
149	171	23	YRETTEAAIIAAYIGAAFIAVYG	AYR	YGP	0.00158	91.99
80	102	23	GGVSLGIAAYRMQEKWKVKAAYI	IGG	YIQ	0.00154	92.03
103	122	20	QLRWFFVFAAYSYCLYWLFV	IQL	FVA	0.00153	92.07
98	116	19	KAAYIQLRWFFVFAAYSYC	VKA	YCL	0.00153	92.11
98	117	20	KAAYIQLRWFFVFAAYSYCL	VKA	CLY	0.0015	92.15
61	82	22	KAKFVAAWTLKAAAAAYFIGGV	AKA	GVS	0.00149	92.19
188	208	21	LWGGPGPGFIAVYYTVLNDLW	DLW	LWG	0.00148	92.22
186	208	23	NDLWGGPGPGFIAVYYTVLNDLW	LND	LWG	0.00145	92.26
23	35	13	RVRGGRCAVLSCL	CRV	CLP	0.00144	92.3
137	159	23	YMQEKWKVKLAAYRETTEAAIIA	AYM	IAA	0.0014	92.34
34	49	16	CLPKEEQIGKCSTRGR	SCL	GRK	0.0014	92.38
12	29	18	GIINTLQKYYCRVRGGRC	KGI	RCA	0.0014	92.42
170	191	22	YGPGPGAFIAVYYTVLNDLWGG	VYG	GGP	0.00139	92.46
174	191	18	PGAFIAVYYTVLNDLWGG	GPG	GGP	0.00138	92.5
60	82	23	AKAKFVAAWTLKAAAAAYFIGGV	AAK	GVS	0.00138	92.53
115	131	17	YCLYWLFVAAYLRWFFV	SYC	FVF	0.00138	92.57
34	50	17	CLPKEEQIGKCSTRGRK	SCL	RKC	0.00137	92.61
160	182	23	AYIGAAFIAVYGPGPGAFIAVYY	AAY	YYT	0.00136	92.65
199	221	23	VYYTVLNDLWGNKKKTERMQEKW	AVY	KWK	0.00136	92.65

36	53	18	PKEEQIGKCSTRGRKCCR	LPK	CRR	0.00135	92.73
200	221	22	YYTVLNDLWGNKKKTERMQEKW	VYY	KWK	0.00135	92.77
174	193	20	PGAFIAVYYTVLNDLWGGPG	GPG	PGP	0.00134	92.8
65	86	22	VAAWTLKAAAAAYFIGGVSLGI	FVA	GIA	0.00134	92.84
5	27	23	ALEAAAKGIINTLQKYYCRVRGG	HAL	GGR	0.00132	92.88
51	63	13	CCRRKKEAAAKAK	KCC	AKF	0.00131	92.92
34	47	14	CLPKEEQIGKCSTR	SCL	TRG	0.00131	92.96
145	167	23	KLAAYRETTEAAIIAAYIGAAFI	VKL	FIA	0.0013	93.0
175	197	23	GAFIAVYYTVLNDLWGGPGPGFI	PGA	FIA	0.0013	93.0
140	162	23	EKWKVKLAAYRETTEAAIIAAYI	QEK	YIG	0.00127	93.08
120	142	23	LFVAAYLRWFFVFSTAAYMQEKW	WLF	KWK	0.00127	93.11
103	117	15	QLRWFFVFAAYSYCL	IQL	CLY	0.00126	93.15
150	171	22	RETTEAAIIAAYIGAAFIAVYG	YRE	YGP	0.00122	93.19
27	44	18	GRCAVLSCLPKEEQIGKC	GGR	KCS	0.0012	93.23
137	158	22	YMQEKWKVKLAAYRETTEAAII	AYM	IIA	0.00116	93.27
172	189	18	PGPGAFIAVYYTVLNDLW	GPG	LWG	0.00112	93.31
169	191	23	VYGPGPGAFIAVYYTVLNDLWGG	AVY	GGP	0.00111	93.35
192	212	21	PGPGFIAVYYTVLNDLWGNKK	GPG	KKK	0.0011	93.38
97	116	20	VKAAYIQLRWFFVFAAYSYC	KVK	YCL	0.00109	93.42
2	23	22	PPHALEAAAKGIINTLQKYYCR	APP	CRV	0.00109	93.46
115	129	15	YCLYWLFVAAYLRWF	SYC	WFF	0.00107	93.5
9	30	22	AAKGIINTLQKYYCRVRGGRCA	AAA	CAV	0.00106	93.54
103	124	22	QLRWFFVFAAYSYCLYWLFVAA	IQL	AAY	0.00104	93.58
97	117	21	VKAAYIQLRWFFVFAAYSYCL	KVK	CLY	0.00104	93.62
16	34	19	TLQKYYCRVRGGRCAVLSC	NTL	SCL	0.00104	93.66
37	52	16	KEEQIGKCSTRGRKCC	PKE	CCR	0.00103	93.69
116	131	16	CLYWLFVAAYLRWFFV	YCL	FVF	0.00101	93.73
115	132	18	YCLYWLFVAAYLRWFFVF	SYC	VFS	0.00101	93.77
172	192	21	PGPGAFIAVYYTVLNDLWGGP	GPG	GPG	0.00101	93.81
174	194	21	PGAFIAVYYTVLNDLWGGPGP	GPG	GPG	0.00101	93.81
116	130	15	CLYWLFVAAYLRWFF	YCL	FFV	0.001	93.89
36	58	23	PKEEQIGKCSTRGRKCCRRKKEA	LPK	EAA	0.001	93.93
50	69	20	KCCRRKKEAAAKAKFVAAWT	RKC	WTL	0.00099	93.97
50	71	22	KCCRRKKEAAAKAKFVAAWTLK	RKC	LKA	0.00099	94.0
3	21	19	PHALEAAAKGIINTLQKYY	PPH	YYC	0.00098	94.04
64	86	23	FVAAWTLKAAAAAYFIGGVSLGI	KFV	GIA	0.00097	94.08
141	162	22	KWKVKLAAYRETTEAAIIAAYI	EKW	YIG	0.00095	94.12
192	213	22	PGPGFIAVYYTVLNDLWGNKKK	GPG	KKT	0.00095	94.16
194	216	23	PGFIAVYYTVLNDLWGNKKKTER	GPG	ERM	0.00093	94.2
3	25	23	PHALEAAAKGIINTLQKYYCRVR	PPH	VRG	0.00093	94.24
28	41	14	RCAVLSCLPKEEQI	GRC	QIG	0.00093	94.27
181	203	23	YYTVLNDLWGGPGPGFIAVYYTV	VYY	TVL	0.00092	94.31
22	42	21	CRVRGGRCAVLSCLPKEEQIG	YCR	IGK	0.00091	94.35
52	67	16	CRRKKEAAAKAKFVAA	CCR	AAW	0.00089	94.39
116	132	17	CLYWLFVAAYLRWFFVF	YCL	VFS	0.00086	94.43
36	51	16	PKEEQIGKCSTRGRKC	LPK	KCC	0.00085	94.47
5	22	18	ALEAAAKGIINTLQKYYC	HAL	YCR	0.00085	94.51
21	38	18	YCRVRGGRCAVLSCLPKE	YYC	KEE	0.00085	94.55
51	64	14	CCRRKKEAAAKAKF	KCC	KFV	0.00083	94.58

194	214	21	PGFIAVYYTVLNDLWGNKKKT	GPG	KTE	0.00079	94.62
51	65	15	CCRRKKEAAAKAKFV	KCC	FVA	0.00078	94.66
103	116	14	QLRWFFVFAAYSYC	IQL	YCL	0.00076	94.7
32	44	13	LSCLPKEEQIGKC	VLS	KCS	0.00075	94.74
17	35	19	LQKYYCRVRGGRCAVLSCL	TLQ	CLP	0.00075	94.78
21	39	19	YCRVRGGRCAVLSCLPKEE	YYC	EEQ	0.00072	94.82
52	65	14	CRRKKEAAAKAKFV	CCR	FVA	0.00071	94.85
4	22	19	HALEAAAKGIINTLQKYYC	PHA	YCR	0.00068	94.89
52	68	17	CRRKKEAAAKAKFVAAW	CCR	AWT	0.00068	94.93
10	29	20	AKGIINTLQKYYCRVRGGRC	AAK	RCA	0.00068	94.97
11	29	19	KGIINTLQKYYCRVRGGRC	AKG	RCA	0.00067	95.01
15	34	20	NTLQKYYCRVRGGRCAVLSC	INT	SCL	0.00065	95.05
194	215	22	PGFIAVYYTVLNDLWGNKKKTE	GPG	TER	0.00065	95.09
50	70	21	KCCRRKKEAAAKAKFVAAWTL	RKC	TLK	0.00064	95.13
8	30	23	AAAKGIINTLQKYYCRVRGGRCA	EAA	CAV	0.00064	95.16
116	128	13	CLYWLFVAAYLRW	YCL	RWF	0.00063	95.2
115	133	19	YCLYWLFVAAYLRWFFVFS	SYC	FST	0.00061	95.24
31	53	23	VLSCLPKEEQIGKCSTRGRKCCR	AVL	CRR	0.0006	95.28
51	67	17	CCRRKKEAAAKAKFVAA	KCC	AAW	0.0006	95.32
25	45	21	RGGRCAVLSCLPKEEQIGKCS	VRG	CST	0.0006	95.36
34	46	13	CLPKEEQIGKCST	SCL	STR	0.0006	95.4
96	116	21	KVKAAYIQLRWFFVFAAYSYC	WKV	YCL	0.00058	95.44
52	64	13	CRRKKEAAAKAKF	CCR	KFV	0.00058	95.47
172	191	20	PGPGAFIAVYYTVLNDLWGG	GPG	GGP	0.00055	95.51
24	45	22	VRGGRCAVLSCLPKEEQIGKCS	RVR	CST	0.00052	95.55
43	64	22	KCSTRGRKCCRRKKEAAAKAKF	GKC	KFV	0.00052	95.59
96	117	22	KVKAAYIQLRWFFVFAAYSYCL	WKV	CLY	0.0005	95.63
116	133	18	CLYWLFVAAYLRWFFVFS	YCL	FST	0.0005	95.67
32	53	22	LSCLPKEEQIGKCSTRGRKCCR	VLS	CRR	0.0005	95.71
172	193	22	PGPGAFIAVYYTVLNDLWGGPG	GPG	PGP	0.00049	95.74
18	35	18	QKYYCRVRGGRCAVLSCL	LQK	CLP	0.00049	95.78
116	129	14	CLYWLFVAAYLRWF	YCL	WFF	0.00048	95.82
50	72	23	KCCRRKKEAAAKAKFVAAWTLKA	RKC	KAA	0.00047	95.86
2	22	21	PPHALEAAAKGIINTLQKYYC	APP	YCR	0.00047	95.9
14	34	21	INTLQKYYCRVRGGRCAVLSC	IIN	SCL	0.00047	95.9
21	40	20	YCRVRGGRCAVLSCLPKEEQ	YYC	EQI	0.00046	95.98
28	46	19	RCAVLSCLPKEEQIGKCST	GRC	STR	0.00041	96.02
28	47	20	RCAVLSCLPKEEQIGKCSTR	GRC	TRG	0.00041	96.05
13	34	22	IINTLQKYYCRVRGGRCAVLSC	GII	SCL	0.00041	96.09
103	125	23	QLRWFFVFAAYSYCLYWLFVAAY	IQL	AYL	0.0004	96.13
51	68	18	CCRRKKEAAAKAKFVAAW	KCC	AWT	0.0004	96.17
115	134	20	YCLYWLFVAAYLRWFFVFST	SYC	STA	0.0004	96.21
172	194	23	PGPGAFIAVYYTVLNDLWGGPGP	GPG	GPG	0.00039	96.25
9	29	21	AAKGIINTLQKYYCRVRGGRC	AAA	RCA	0.00039	96.29
31	51	21	VLSCLPKEEQIGKCSTRGRKC	AVL	KCC	0.00038	96.32
22	41	20	CRVRGGRCAVLSCLPKEEQI	YCR	QIG	0.00036	96.36
22	43	22	CRVRGGRCAVLSCLPKEEQIGK	YCR	GKC	0.00035	96.4
16	35	20	TLQKYYCRVRGGRCAVLSCL	NTL	CLP	0.00034	96.44
32	51	20	LSCLPKEEQIGKCSTRGRKC	VLS	KCC	0.00034	96.48

28	48	21	RCAVLSCLPKEEQIGKCSTRG	GRC	RGR	0.00032	96.52
25	44	20	RGGRCAVLSCLPKEEQIGKC	VRG	KCS	0.00031	96.56
192	214	23	PGPGFIAVYYTVLNDLWGNKKKT	GPG	KTE	0.00031	96.6
24	44	21	VRGGRCAVLSCLPKEEQIGKC	RVR	KCS	0.0003	96.63
43	65	23	KCSTRGRKCCRRKKEAAAKAKFV	GKC	FVA	0.0003	96.67
3	24	22	PHALEAAAKGIINTLQKYYCRV	PPH	RVR	0.0003	96.71
116	134	19	CLYWLFVAAYLRWFFVFST	YCL	STA	0.0003	96.75
36	52	17	PKEEQIGKCSTRGRKCC	LPK	CCR	0.00029	96.79
12	34	23	GIINTLQKYYCRVRGGRCAVLSC	KGI	SCL	0.00028	96.83
95	117	23	WKVKAAYIQLRWFFVFAAYSYCL	KWK	CLY	0.00026	96.87
115	135	21	YCLYWLFVAAYLRWFFVFSTA	SYC	TAA	0.00025	96.91
95	116	22	WKVKAAYIQLRWFFVFAAYSYC	KWK	YCL	0.00025	96.94
52	71	20	CRRKKEAAAKAKFVAAWTLK	CCR	LKA	0.00025	96.98
23	45	23	RVRGGRCAVLSCLPKEEQIGKCS	CRV	CST	0.00024	97.02
3	23	21	PHALEAAAKGIINTLQKYYCR	PPH	CRV	0.00024	97.06
30	51	22	AVLSCLPKEEQIGKCSTRGRKC	CAV	KCC	0.00024	97.1
52	69	18	CRRKKEAAAKAKFVAAWT	CCR	WTL	0.00023	97.14
21	42	22	YCRVRGGRCAVLSCLPKEEQIG	YYC	IGK	0.00021	97.18
33	54	22	SCLPKEEQIGKCSTRGRKCCRR	LSC	RRK	0.00021	97.18
15	35	21	NTLQKYYCRVRGGRCAVLSCL	INT	CLP	0.00021	97.25
174	196	23	PGAFIAVYYTVLNDLWGGPGPGF	GPG	GFI	0.0002	97.29
8	29	22	AAAKGIINTLQKYYCRVRGGRC	EAA	RCA	0.0002	97.33
116	135	20	CLYWLFVAAYLRWFFVFSTA	YCL	TAA	0.0002	97.37
22	34	13	CRVRGGRCAVLSC	YCR	SCL	0.00019	97.41
34	54	21	CLPKEEQIGKCSTRGRKCCRR	SCL	RRK	0.00018	97.45
28	49	22	RCAVLSCLPKEEQIGKCSTRGR	GRC	GRK	0.00016	97.49
94	116	23	KWKVKAAYIQLRWFFVFAAYSYC	EKW	YCL	0.00016	97.52
28	50	23	RCAVLSCLPKEEQIGKCSTRGRK	GRC	RKC	0.00016	97.56
33	55	23	SCLPKEEQIGKCSTRGRKCCRRK	LSC	RKK	0.00016	97.56
51	71	21	CCRRKKEAAAKAKFVAAWTLK	KCC	LKA	0.00016	97.64
115	136	22	YCLYWLFVAAYLRWFFVFSTAA	SYC	AAY	0.00015	97.68
52	70	19	CRRKKEAAAKAKFVAAWTL	CCR	TLK	0.00014	97.72
13	35	23	IINTLQKYYCRVRGGRCAVLSCL	GII	CLP	0.00014	97.76
51	69	19	CCRRKKEAAAKAKFVAAWT	KCC	WTL	0.00014	97.79
22	35	14	CRVRGGRCAVLSCL	YCR	CLP	0.00014	97.83
14	35	22	INTLQKYYCRVRGGRCAVLSCL	IIN	CLP	0.00013	97.87
116	136	21	CLYWLFVAAYLRWFFVFSTAA	YCL	AAY	0.00013	97.91
23	44	22	RVRGGRCAVLSCLPKEEQIGKC	CRV	KCS	0.00012	97.95
33	45	13	SCLPKEEQIGKCS	LSC	CST	0.00012	97.99
34	55	22	CLPKEEQIGKCSTRGRKCCRRK	SCL	RKK	0.00012	98.03
21	34	14	YCRVRGGRCAVLSC	YYC	SCL	0.00011	98.07
52	72	21	CRRKKEAAAKAKFVAAWTLKA	CCR	KAA	0.00011	98.1
31	52	22	VLSCLPKEEQIGKCSTRGRKCC	AVL	CCR	0.00011	98.14
32	52	21	LSCLPKEEQIGKCSTRGRKCC	VLS	CCR	0.00011	98.18
7	29	23	EAAAKGIINTLQKYYCRVRGGRC	LEA	RCA	0.0001	98.22
21	43	23	YCRVRGGRCAVLSCLPKEEQIGK	YYC	GKC	0.0001	98.26
3	22	20	PHALEAAAKGIINTLQKYYC	PPH	YCR	9e-05	98.3
52	73	22	CRRKKEAAAKAKFVAAWTLKAA	CCR	AAA	9e-05	98.34
51	70	20	CCRRKKEAAAKAKFVAAWTL	KCC	TLK	9e-05	98.38

21	41	21	YCRVRGGRCAVLSCLPKEEQI	YYC	QIG	9e-05	98.41
30	52	23	AVLSCLPKEEQIGKCSTRGRKCC	CAV	CCR	8e-05	98.45
28	45	18	RCAVLSCLPKEEQIGKCS	GRC	CST	6e-05	98.49
51	73	23	CCRRKKEAAAKAKFVAAWTLKAA	KCC	AAA	6e-05	98.53
52	74	23	CRRKKEAAAKAKFVAAWTLKAAA	CCR	AAA	6e-05	98.53
51	72	22	CCRRKKEAAAKAKFVAAWTLKA	KCC	KAA	6e-05	98.61
34	56	23	CLPKEEQIGKCSTRGRKCCRRKK	SCL	KKE	6e-05	98.65
21	35	15	YCRVRGGRCAVLSCL	YYC	CLP	6e-05	98.68
115	137	23	YCLYWLFVAAYLRWFFVFSTAAY	SYC	AYM	5e-05	98.72
33	53	21	SCLPKEEQIGKCSTRGRKCCR	LSC	CRR	4e-05	98.76
28	44	17	RCAVLSCLPKEEQIGKC	GRC	KCS	4e-05	98.8
34	53	20	CLPKEEQIGKCSTRGRKCCR	SCL	CRR	3e-05	98.84
116	137	22	CLYWLFVAAYLRWFFVFSTAAY	YCL	AYM	3e-05	98.88
33	51	19	SCLPKEEQIGKCSTRGRKC	LSC	KCC	3e-05	98.92
116	138	23	CLYWLFVAAYLRWFFVFSTAAYM	YCL	YMQ	2e-05	98.96
34	51	18	CLPKEEQIGKCSTRGRKC	SCL	KCC	2e-05	98.99
33	52	20	SCLPKEEQIGKCSTRGRKCC	LSC	CCR	1e-05	99.03
34	52	19	CLPKEEQIGKCSTRGRKCC	SCL	CCR	1e-05	99.07
22	44	23	CRVRGGRCAVLSCLPKEEQIGKC	YCR	KCS	1e-05	99.11
29	41	13	CAVLSCLPKEEQI	RCA	QIG	0.0	99.15
44	56	13	CSTRGRKCCRRKK	KCS	KKE	0.0	99.15
29	42	14	CAVLSCLPKEEQIG	RCA	IGK	0.0	99.15
44	57	14	CSTRGRKCCRRKKE	KCS	KEA	0.0	99.15
29	43	15	CAVLSCLPKEEQIGK	RCA	GKC	0.0	99.15
44	58	15	CSTRGRKCCRRKKEA	KCS	EAA	0.0	99.15
29	44	16	CAVLSCLPKEEQIGKC	RCA	KCS	0.0	99.15
44	59	16	CSTRGRKCCRRKKEAA	KCS	AAA	0.0	99.15
29	45	17	CAVLSCLPKEEQIGKCS	RCA	CST	0.0	99.15
44	60	17	CSTRGRKCCRRKKEAAA	KCS	AAK	0.0	99.15
29	46	18	CAVLSCLPKEEQIGKCST	RCA	STR	0.0	99.15
44	61	18	CSTRGRKCCRRKKEAAAK	KCS	AKA	0.0	99.15
29	47	19	CAVLSCLPKEEQIGKCSTR	RCA	TRG	0.0	99.15
44	62	19	CSTRGRKCCRRKKEAAAKA	KCS	KAK	0.0	99.15
29	48	20	CAVLSCLPKEEQIGKCSTRG	RCA	RGR	0.0	99.15
44	63	20	CSTRGRKCCRRKKEAAAKAK	KCS	AKF	0.0	99.15
29	49	21	CAVLSCLPKEEQIGKCSTRGR	RCA	GRK	0.0	99.15
44	64	21	CSTRGRKCCRRKKEAAAKAKF	KCS	KFV	0.0	99.15
29	50	22	CAVLSCLPKEEQIGKCSTRGRK	RCA	RKC	0.0	99.15
44	65	22	CSTRGRKCCRRKKEAAAKAKFV	KCS	FVA	0.0	99.15
29	51	23	CAVLSCLPKEEQIGKCSTRGRKC	RCA	KCC	0.0	99.15
44	66	23	CSTRGRKCCRRKKEAAAKAKFVA	KCS	VAA	0.0	99.15
