# Supplementary material for: Immunoinformatic Design of a Multivalent Peptide Vaccine Against Mucormycosis: Targeting FTR1 Protein of Major Causative Fungi
Source: Front Immunol. 2022 May 26;13:863234. doi: 10.3389/fimmu.2022.863234 (PMC9204303; doi:10.3389/fimmu.2022.863234)
Supplement: Supplementary file 13 [file Table_7.pdf]

Table S7. The proteasomal cleavage analysis of BFV using NetChop3.1 server.

Position	AA	Score
1	A	0.071878
2	P	0.029435
3	P	0.030615
4	H	0.087373
5	A	0.367544
6	L	0.938775
7	E	0.091489
8	A	0.653481
9	A	0.288792
10	A	0.119621
11	K	0.068105
12	G	0.034161
13	I	0.075430
14	I	0.061336
15	N	0.023037
16	T	0.026648
17	L	0.973778
18	Q	0.033868
19	K	0.838980
20	Y	0.973811
21	Y	0.935643
22	C	0.027445
23	R	0.255031
24	V	0.819229
25	R	0.064459
26	G	0.031110
27	G	0.046675
28	R	0.202127
29	C	0.024837
30	A	0.264823
31	V	0.924106
32	L	0.976550
33	S	0.038700
34	C	0.026511
35	L	0.852203
36	P	0.029256
37	K	0.603841
38	E	0.025129
39	E	0.044170
40	Q	0.024525
41	I	0.405644
42	G	0.066268
43	K	0.961205
44	C	0.048070
45	S	0.029299
46	T	0.047160
47	R	0.491540
48	G	0.057264
49	R	0.195221
50	K	0.472570
51	C	0.029904

52	C	0.029176
53	R	0.158588
54	R	0.339527
55	K	0.116702
56	K	0.555187
57	E	0.023207
58	A	0.031236
59	A	0.026863
60	A	0.061747
61	K	0.340550
62	A	0.290573
63	K	0.071526
64	F	0.306480
65	V	0.086420
66	A	0.160237
67	A	0.114474
68	W	0.659507
69	T	0.027265
70	L	0.930341
71	K	0.520393
72	A	0.305182
73	A	0.093138
74	A	0.402330
75	A	0.074385
76	A	0.037643
77	Y	0.945238
78	F	0.133452
79	I	0.099314
80	G	0.030515
81	G	0.028598
82	V	0.266933
83	S	0.024113
84	L	0.965359
85	G	0.041539
86	I	0.586986
87	A	0.045698
88	A	0.049401
89	Y	0.970894
90	R	0.239010
91	M	0.888978
92	Q	0.024900
93	E	0.023401
94	K	0.086497
95	W	0.270068
96	K	0.048705
97	V	0.781047
98	K	0.261014
99	A	0.079333
100	A	0.078647
101	Y	0.699319
102	I	0.293840
103	Q	0.024301
104	L	0.633672
105	R	0.046658

106	W	0.477237
107	F	0.063168
108	F	0.130424
109	V	0.129243
110	F	0.744407
111	A	0.323635
112	A	0.625293
113	Y	0.959150
114	S	0.029716
115	Y	0.945029
116	C	0.024673
117	L	0.356297
118	Y	0.212343
119	W	0.644326
120	L	0.097477
121	F	0.211632
122	V	0.323980
123	A	0.045328
124	A	0.274418
125	Y	0.965157
126	L	0.468117
127	R	0.260128
128	W	0.559107
129	F	0.063116
130	F	0.055278
131	V	0.226962
132	F	0.944519
133	S	0.061536
134	T	0.594812
135	A	0.772362
136	A	0.420902
137	Y	0.888950
138	M	0.723875
139	Q	0.042069
140	E	0.023249
141	K	0.250383
142	W	0.175776
143	K	0.044147
144	V	0.730994
145	K	0.760204
146	L	0.953891
147	A	0.318548
148	A	0.429885
149	Y	0.953925
150	R	0.125439
151	E	0.024935
152	T	0.026478
153	T	0.060707
154	E	0.023171
155	A	0.180299
156	A	0.122909
157	I	0.452268
158	I	0.144759
159	A	0.067277

160	A	0.052309
161	Y	0.773130
162	I	0.185146
163	G	0.023958
164	A	0.050848
165	A	0.086726
166	F	0.520330
167	I	0.766965
168	A	0.659837
169	V	0.829167
170	Y	0.941164
171	G	0.029327
172	P	0.027461
173	G	0.024978
174	P	0.022256
175	G	0.024560
176	A	0.140381
177	F	0.314337
178	I	0.108654
179	A	0.285695
180	V	0.526398
181	Y	0.968188
182	Y	0.692883
183	T	0.154359
184	V	0.968843
185	L	0.936273
186	N	0.321845
187	D	0.059637
188	L	0.976196
189	W	0.866170
190	G	0.027908
191	G	0.031674
192	P	0.024603
193	G	0.023861
194	P	0.023594
195	G	0.099858
196	F	0.084114
197	I	0.055425
198	A	0.061084
199	V	0.950889
200	Y	0.954840
201	Y	0.959904
202	T	0.074305
203	V	0.940435
204	L	0.758826
205	N	0.044004
206	D	0.025654
207	L	0.960282
208	W	0.681481
209	G	0.025030
210	N	0.028432
211	K	0.769383
212	K	0.813224
213	K	0.591145

214	T	0.484626
215	E	0.026278
216	R	0.079838
217	M	0.600097
218	Q	0.024827
219	E	0.022584
220	K	0.324193
221	W	0.465894
222	K	0.044293
223	V	0.665734
224	K	0.574585
225	K	0.349222
226	K	0.377774
227	A	0.317980
228	K	0.077148
229	F	0.225260
230	V	0.233949
231	A	0.155320
232	A	0.081588
233	W	0.629979
234	T	0.024852
235	L	0.962614
236	K	0.917892
237	A	0.757311
238	A	0.069091
239	A	0.356628
240	K	0.544729
241	K	0.665048
242	T	0.055457
243	G	0.026727
244	A	0.107647
245	L	0.803796
246	L	0.910491
247	A	0.282653
248	A	0.232763
249	G	0.044462
250	A	0.101609
251	A	0.284938
252	A	0.542139
253	K	0.877494
254	K	0.861939
