# Supplementary material for: Immunoinformatic Design of a Multivalent Peptide Vaccine Against Mucormycosis: Targeting FTR1 Protein of Major Causative Fungi
Source: Front Immunol. 2022 May 26;13:863234. doi: 10.3389/fimmu.2022.863234 (PMC9204303; doi:10.3389/fimmu.2022.863234)
Supplement: Supplementary file 14 [file Table_8.pdf]

Table S8. Results of the molecular docking analyses of BFV with different TLRs.

Target TLRs (with PDB IDs)	ClusPro energy score	Global energy (PatchDock server)	MM-GBSA (binding free energy, in kcal mol^{-1})
TLR-1 (6NIH)	-1097.7	3.2	-58.9
TLR-2 (3A7C)	-1116.9	3.2	-61.3
TLR-4 (4G8A)	-1214.9	4.1	-68.4
