# Supplementary material for: Immunoinformatic Design of a Multivalent Peptide Vaccine Against Mucormycosis: Targeting FTR1 Protein of Major Causative Fungi
Source: Front Immunol. 2022 May 26;13:863234. doi: 10.3389/fimmu.2022.863234 (PMC9204303; doi:10.3389/fimmu.2022.863234)
Supplement: Supplementary file 15 [file Table_9.pdf]

Table S9. The list of the predicted conformational B-cell epitopes of BFV, with the number of residues and their scores.

No.	Residues	Number of residues	Score
1	A:A227, A:K228, A:F229, A:V230, A:A231, A:A232, A:W233, A:T234, A:L235, A:K236, A:A237, A:A238, A:A239, A:K240, A:K241, A:T242, A:G243, A:A244, A:L245, A:L246, A:A247, A:A248, A:G249, A:A250, A:A251, A:A252, A:K253, A:K254	28	0.913
2	A:F132, A:S133, A:T134, A:A135, A:A136, A:Y137, A:M138, A:Q139, A:E140, A:K141, A:W142, A:K143, A:K145, A:L146, A:Y149	15	0.739
3	A:G12, A:N15, A:T16, A:L17, A:Q18, A:K19, A:Y20, A:Y21, A:C22, A:R23, A:V24, A:R25, A:V31, A:L32, A:S33, A:C34, A:L35, A:P36, A:K37, A:E38, A:E39, A:Q40, A:I41, A:G42, A:K43, A:C44, A:S45, A:T46, A:R47, A:G48, A:R49, A:K50, A:C51, A:C52, A:R53, A:R54, A:K55, A:K56, A:E57, A:A58, A:A59, A:A60, A:K61, A:A62, A:F64	45	0.721
4	A:W221, A:V223, A:K224, A:K226	4	0.67
5	A:K213, A:T214, A:E215, A:Q218, A:E219, A:K222	6	0.649
6	A:S83, A:G85, A:I86, A:A88, A:Y89, A:R90, A:E93, A:K94, A:W95, A:K96, A:V97, A:K98	12	0.647
7	A:G190, A:G191, A:P192, A:G193, A:P194, A:G195, A:A198, A:Y201, A:T202, A:N205	10	0.566
